# Origin of Suppressed Chain Transfer in Phosphinephenolato Ni(II)-Catalyzed Ethylene Polymerization

**DOI:** 10.1021/jacs.3c06597

**Published:** 2023-12-16

**Authors:** Fei Lin, Maria Voccia, Lukas Odenwald, Inigo Göttker-Schnetmann, Laura Falivene, Lucia Caporaso, Stefan Mecking

**Affiliations:** † Chair of Chemical Materials Science, Department of Chemistry, University of Konstanz, 78457 Konstanz, Germany; ‡ Dipartimento di Chimica e Biologia, Università di Salerno, Via Papa Paolo Giovanni II, I-84084 Fisciano, Italy

## Abstract

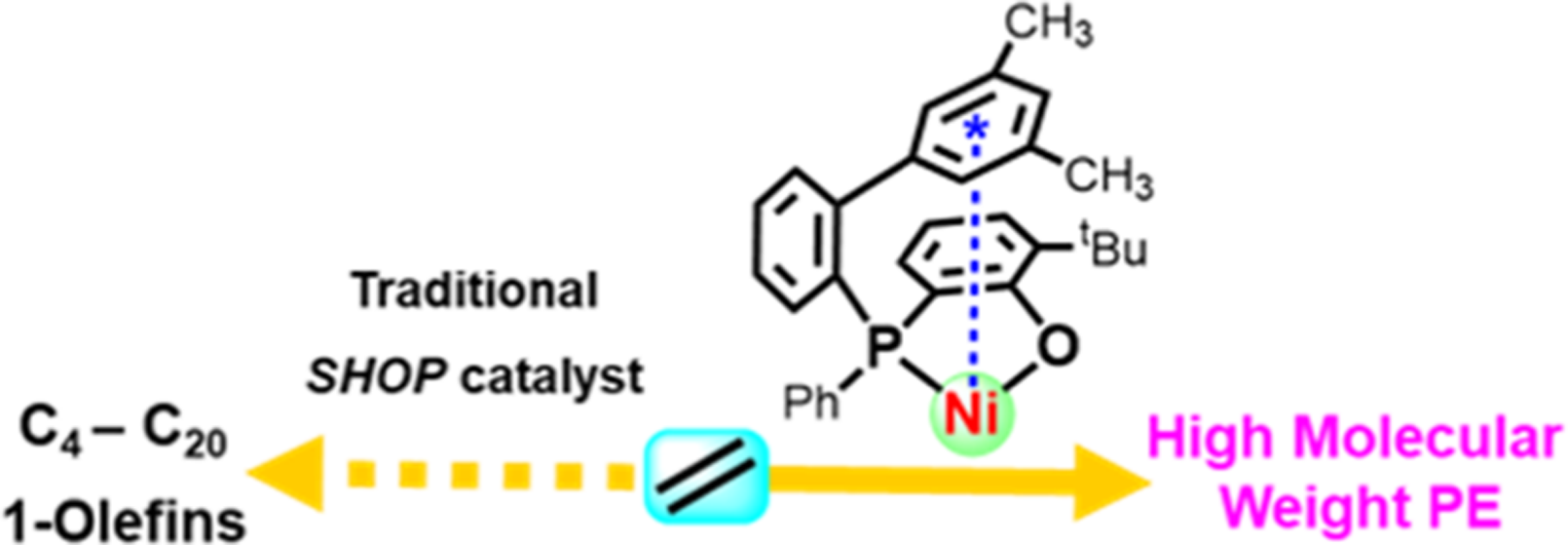

Recent breakthroughs in the generation of polar-functionalized and more sustainable degradable polyethylenes have been enabled by advanced phosphinephenolato Ni(II) catalysts. A key has been to overcome this type of catalysts’ propensity for extensive chain transfer to enable formation of high-molecular-weight polyethylene chains. We elucidate the mechanistic origin of this paradigm shift by a combined experimental and theoretical study. Single-crystal X-ray structural analysis and cyclic voltammetry of a set of six different catalysts with variable electronics and sterics, combined with extensive pressure reactor polymerization studies, suggest that an attractive Ni–aryl interaction of a *P*-[2-(aryl)phenyl] is responsible for the suppression of chain transfer. This differs from the established picture of steric shielding found for other prominent late transition metal catalysts. Extensive density functional theory studies identify the relevant pathways of chain growth and chain transfer and show how this attractive interaction suppresses chain transfer.

## Introduction

Polyolefins are among the largest-scale synthetic man-made materials. Their myriad of applications in virtually all fields of modern technologies is essentially enabled by microstructure control during their catalytic synthesis, which in turn determines the materials and processing properties.^1−3^ A desirable feature that has long been lacking is the ability to introduce in-chain functional groups during olefin polymerization. Traditional olefin polymerization catalysts based on early transition d^0^-metal active sites are highly oxophilic and extremely sensitive to heteroatom-containing reagents or reaction media, a limitation that can be overcome by less oxophilic late transition metal catalysts.^4−14^ Most recently, state-of-the-art Ni(II) phosphinephenolate catalysts have been found to enable the long-sought nonalternating copolymerization^15,16^ of ethylene and carbon monoxide to high-molecular-weight keto-polyethylenes with largely isolated in-chain keto groups.^17,18^ These do not compromise the desirable processing and materials properties of polyethylene while enabling photolytic degradation that could alleviate the problematic environmental persistency of polyethylene litter.

A key to this advance is catalysts capable of polyethylene chain growth to high molecular weights. This is notable all the more as Ni(II) phosphinophenolate catalysts are the textbook example of late transition metal catalysts’ propensity to undergo chain transfer via β-hydride elimination (BHE). The Shell higher olefin process for ethylene oligomerization to 1-olefins is based on these catalysts’ ability of effectively competitive chain transfer, with typically the growth rate being only five- to 10-fold higher than the elimination rate.^19−21^ Before this background, the finding of Shimizu et al.^22^ and Li et al.^23^ of catalysts with *P*-[2-(aryl)phenyl] substituents that promote ethylene polymerization to high-molecular-weight polymer (*M*_n_ > 10^5^ g mol^–1^) was an unexpected breakthrough. Further development^24,25^ recently enabled polymerizations with Ni(II) phosphinephenolate catalysts in which chain transfer is virtually completely suppressed, resulting in living chain growth into the ultrahigh-molecular-weight regime, in aqueous polymerizations.^26,27^

Considering the origin of the capability to form high-molecular-weight polymers, for Brookhart’s prototypical cationic Ni(II) diimine catalysts,^28^ it is understood that a steric shielding of axial sites hinders chain transfer from a detailed mechanistic understanding of the mechanism of polymerization.^29−31^ In the development of late transition metal olefin polymerization catalysts, in general, the utilization of sterically bulky substituents has proven to be a successful practical guideline. A series of *ortho*-phosphinebenzenesulfonato Pd(II) and Ni(II) catalysts were synthesized to quantify their correlation between the steric bulk of the ligands and molecular weight of (co)polymers, indicating the imperative of blocking of the axial position on the inhibition of chain transfer and the production of high-molecular-weight polymers.^32−34^ Studies of chelated phosphineenolate catalysts with an aliphatic backbone highlighted the importance of bulky substituents for catalyst activity, thermal stability, and polyethylene molecular weight.^35^ The rationale derived from this overall picture does not account for the actual origin of the aforementioned unique and useful Ni(II) phosphinephenolate catalysts’ ability to suppress chain transfer, which has remained unclear so far.

We now report a combined experimental and theoretical study that reveals the relevant parameters of chain growth and chain transfer and shows that a weak attractive interaction contributes to the suppression of chain transfer in the uniquely versatile phosphinephenolato Ni(II) complexes.

## Results and Discussion

### Synthesis and Structure of Phosphinephenolato Ni(II) Complexes

**Figure 1:**
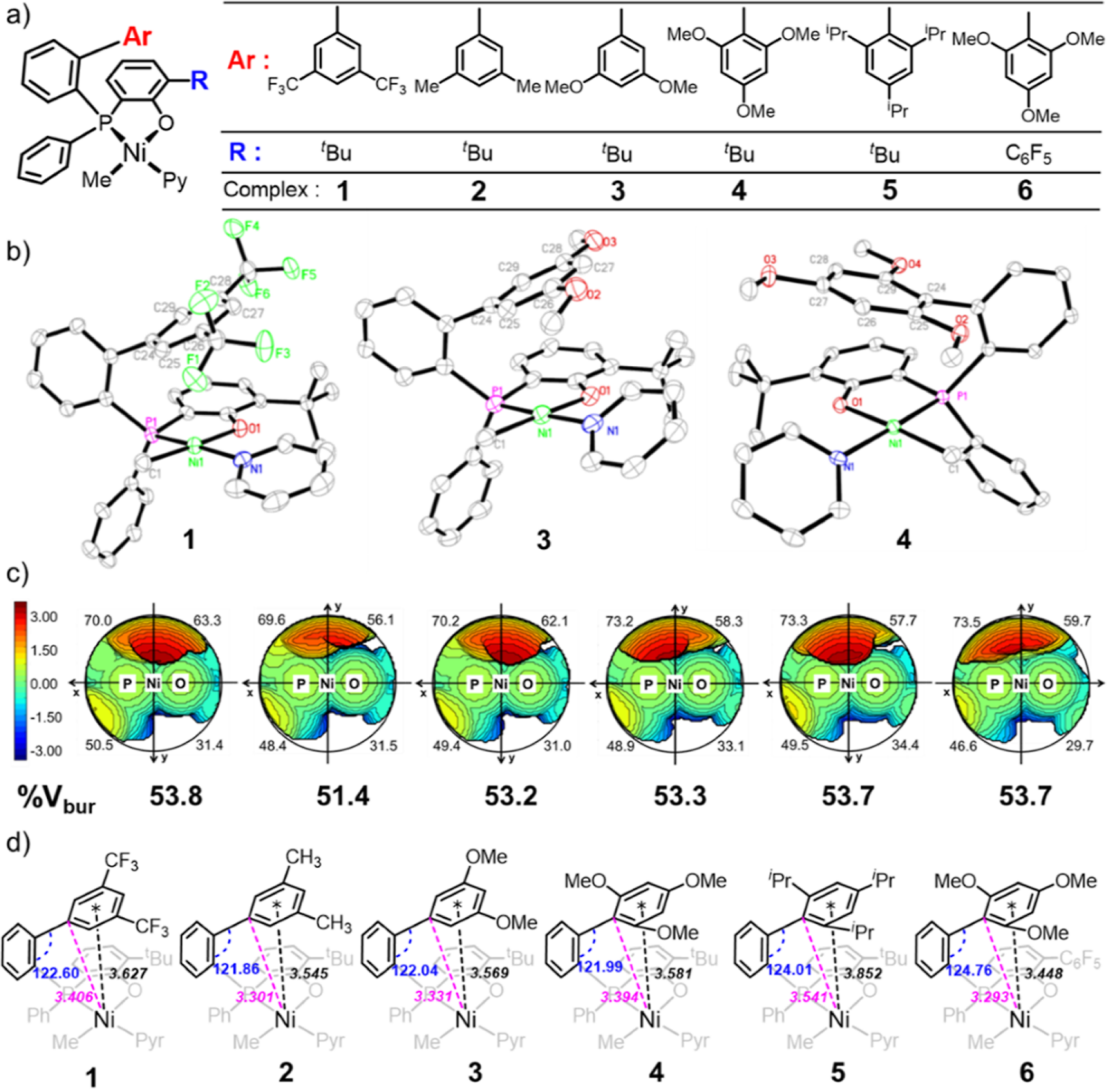
(a) Structures of phosphinephenolato Ni(II) complexes **1–6** studied. (b) Crystal structures of complexes **1**, **3**, **4**, hydrogen atoms are excluded for clarity. (c) Topographical steric maps with % *V*_bur_ in total and divided by quadrants for complexes **1–6** (from left to right), distance in the color scale is in Å, H atoms were included in the calculations. (d) Angle of C24 with the adjacent phenyl, the distance [Å] of the Ni center to the aryl *ipso*-carbon C24 and the center of the aryl (*) for complexes **1–6**.

To allow for the fine-tuning of the electronic and steric environment of the nickel catalysts, diaryl halides were prepared according to established procedures and used in the following syntheses. Phosphinephenolate ligands were obtained in good yield through successive reaction of Cl_2_PPh with different lithiated aryl compounds (cf. the Supporting Information for experimental details and characterization data for all compounds). All six different ligands were reacted with [(tmeda)NiMe_2_] in the presence of excess pyridine to give the corresponding Ni complexes **1–6** in virtually quantitative yield (>95%) (Figure 1a).

Characterization by single-crystal X-ray diffraction revealed a square planar geometry with the Ni–Me group coordinated trans to the oxygen atoms of the chelate (Figures 1b and S27–S32 and Tables S2–S7 in the Supporting Information). Analysis by SambVca 2.1^36^ provided topographical steric maps and percent buried volume data (%*V*_bur_) related to the chelating ligands’ space filling properties (Figure 1c). The most sterically hindered areas for all six complexes are in one axial position of the nickel center; this originates from the *ortho*-aryl groups (C24 to C29). The comparable %*V*_bur_ in total and quadrants for complexes **1–6** reveals that the variation of the substitution patterns on the *ortho*-aryl groups has little impact on the steric environment of the nickel center. Further analysis of the crystal structure data shows that the differences of Ni–C1, Ni–N1, Ni–O1, and Ni–P1 bond lengths of these complexes are negligible (Table S8). The *ortho*-aryl groups are located in one axial position of the nickel center, with the closest distance between the oxygen atoms of an -OMe aryl substituent and the Ni atom being 3.584 Å in complex **6**, which is larger than the sum of the van der Waals radii (*r*_w_) determined by Bondi (3.15 Å),^37^ indicating that the interaction between the -OMe group and the nickel center is negligible. Remarkably, the distance between the aryl *ipso*-carbon C24 and the nickel center in the complexes with electron-donating substituted axial aryl groups (3.301, 3.331, 3.394, and 3.293 Å for **2**, **3**, **4**, and **6**, respectively) is smaller than or close to the sum of van der Waals radii (*r*_w_, 3.33 Å) of a carbon and a nickel atom,^37^ indicating a weak attractive interaction between the *ipso*-carbon C24 and the nickel center (Figure 1d). This weak interaction can be obstructed by electron-withdrawing or sterically bulky substituents on the axial aryl groups, as evidenced by the larger C24–Ni distances in complexes **1** (3.406 Å) and **5** (3.541 Å).

Such a scenario is also underlined by the smaller distance between the center of the axial aryl group and the nickel atom in complexes **2** (3.545 Å), **3** (3.569 Å), **4** (3.581 Å), and **6** (3.448 Å) compared to that in complexes **1** (3.627 Å) and **5** (3.852 Å), in which the low electron density of the 3,5-bis(trifluoromethyl)phenyl in **1** and bulky nature of the 2,4,6-tri(isopropyl)phenyl in **5**, respectively, may obstruct the interaction between this phenyl group and the nickel center.

The ligand basicity is reflected by the phosphorus nuclear magnetic resonance ^31^P NMR resonance, which is low-field-shifted with increasing electron deficiency.^38^ Complex **1** bearing the electron-withdrawing 3,5-bis(trifluoromethyl)phenyl group features the highest ^31^P NMR chemical shift (δ 27.38 ppm), while complexes’ **4** and **6** resonate at the lowest chemical shift (δ 20.75 and 19.52, respectively) in the series, owing to the strongly electron-donating 2,4,6-trimethoxyphenyl substitution (Table 1). The different electronic environments generated by these phosphinephenolato ligands result in different electron densities at the Ni center, as observed by cyclic voltammetry (CV). The forward peak potentials of the Ni(II)/Ni(III) pair of the complexes are highly dependent on the P-phenyl group’s aryl substituent. The more electron-rich the substituent is, the higher is the electron density at the Ni atom (*E*_1/2_ = 174, 45, 28, 9, and −79 mV for **1**, **2**, **5**, **3**, and **4**, respectively). In addition, the electron-withdrawing group in the ortho-position to the phenolate in **6** decreases the electron density at the Ni atom (*E*_1/2_ = −79 mV for **4** vs *E*_1/2_ = 88 mV for **6**).

**Table 1:** ^31^P NMR Shifts and Forward Peak Potentials from CV of Complexes **1**–**6**

entry	complex	δ *P* [ppm]	*E*_1/2_ [mV]^a^
1	**1**	27.38	174
2	**2**	23.54	45
3	**3**	23.41	9
4	**4**	20.75	–79
5	**5**	23.63	28
6	**6**	19.52	88

a Determined from CV against [Cp*_2_Fe]/[Cp*_2_Fe]^+^ (Cp* = pentamethyl cyclopentadienyl) in dichloromethane (DCM), NBu_4_PF_6_ as electrolyte, and a sweep rate of 100 mV s^–1^.

### Catalytic Properties under Pressure Reactor Conditions

**Figure 2:**
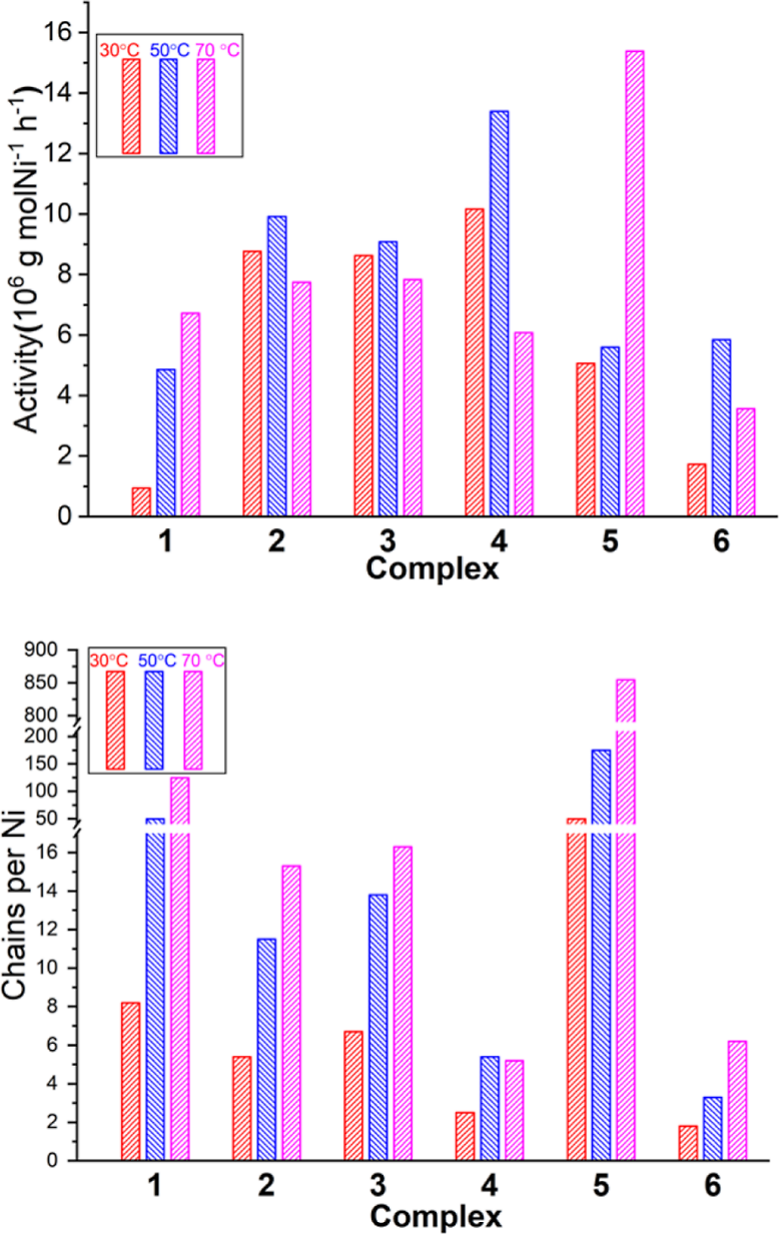
Catalytic activity (top) and chains per Ni (bottom) of the different catalysts in ethylene polymerization (1 μmol catalyst in 100 mL toluene, *P*_ethylene_ = 10 bar, 30 min).

The catalytic properties of all complexes toward ethylene were investigated at different polymerization temperatures (Table 2). Monitoring of the polymerization via the mass flow of ethylene uptake revealed a reasonable stability for all catalysts in the entire temperature regime studied (up to 70 °C. Figures S34–S47 in the Supporting Information). The polymerization activity and particularly the propensity for chain transfer vary significantly for the different catalyst precursors (Figure 2).

**Table 2:** Ethylene Polymerization by Complexes **1–6**^a^

entry	complex	*T* (°C)	yield (g)	activity^b^	*M*_n_^c^ (×10^3^)	*M*_w_/*M*_n_^c^	chains per nickel	*T*_m_^d^ (°C)	cryst.^d^ (%)
1	**1**	30	0.47	0.94	57	2.0	8	135	66
2	**1**	50	2.43	4.86	49	1.7	50	136	76
3	**1**	70	3.36	6.72	27	1.8	124	135	76
4	**2**	30	4.38	8.76	816	2.0	5	139	50
5	**2**	50	4.96	9.92	430	2.0	12	141	54
6	**2**	70	2.87	5.74	187	2.1	15	138	55
7	**3**	30	4.31	8.62	646	1.9	7	137	54
8	**3**	50	4.54	9.08	329	2.2	14	139	57
9	**3**	70	3.69	7.83	226	2.0	16	137	66
10	**4**	30	5.08	10.16	2062	1.6	3	138	47
11	**4**	50	6.70	13.40	1235	1.9	5	139	49
12	**4**	70	3.04	6.08	485	1.7	5	141	50
13	**5**	30	2.53	5.06	51	1.8	50	135	68
14	**5**	50	2.80	5.60	16	1.8	175	133	76
15	**5**	70	7.69	15.38	9	1.7	854	131	78
16	**6**	30	0.86	1.72	479	1.5	2	140	51
17	**6**	50	2.92	5.84	888	1.7	3	140	47
18	**6**	70	1.78	3.56	287	2.0	6	135	53
19	**1-tmeda**	30	3.13	6.26	93	1.9	34	139	69
20	**1-tmeda**	50	2.69	5.38	45	1.7	60	136	75
21	**1-tmeda**	70	2.66	5.32	32	1.6	83	135	82

a Polymerization conditions: 1 μmol of catalyst precursor in 100 mL of toluene, 10 bar ethylene pressure, 30 min polymerization time, 1000 rpm stirring rate.

b Given in 10^6^ g_PE_ mol_Ni_^–1^ h^–1^.

c Determined via gel permeation chromatography (GPC) at 160 °C in 1,2-dichlorobenzene.

d Determined by differential scanning calorimetry (DSC) with 10 K min^–1^ heating/cooling rate (data from the second heating cycle), crystallinities were determined assuming a melt enthalpy of 293 J g^–1^ for 100% crystalline polyethylene.

Complex **1** bearing an electron-withdrawing 3,5-bis(trifluoromethyl)phenyl substituent showed the lowest catalytic activity (9.4 × 10^5^ g mol_Ni_^–1^ h^–1^) for ethylene polymerization and gave linear polyethylene with comparatively low molecular weight (*M*_n_ 57 kg mol^–1^) at 30 °C. The activity increased to 4.86 × 10^6^ and 6.72 × 10^6^ g mol_Ni_^–1^ h^–1^at 50 and 70 °C, but as expected the molecular weight *M*_*n*_ decreased to 49 and 27 kg mol^–1^, respectively (Table 2, entries 1–3). The relatively high propensity for chain transfer during polymerization also reflects in a high number of chains per nickel (up to 124 at 70 °C). Complexes **2** and **3** are significantly more active than complex **1** at 30 °C (8.76 × 10^6^ g mol_Ni_^–1^ h^–1^ for **2** and 8.62 × 10^6^ g mol_Ni_^–1^ h^–1^ for **3**) and 50 °C (9.92 × 10^6^ g mol_Ni_^–1^ h^–1^ for **2** and 9.08 × 10^6^ g mol_Ni_^–1^ h^–1^ for **3**) and display a comparable polymerization activity at 70 °C (Table 2, entries 4–9). Remarkably, the electron-donating 3,5-bis(methyl)phenyl and 3,5-bis(methoxy)phenyl, respectively, substituents in **2** and **3** result in efficiently suppressed chain transfer in all cases, with less than 20 chains generated per nickel even at 70 °C. With the yet more electron-donating 2,4,6-tri(methoxy)phenyl substitution in **4**, the polymerization activities at 30 and 50 °C were further enhanced to 1.02 × 10^7^ g mol_Ni_^–1^ h^–1^ and 1.34 × 10^7^ g mol_Ni_^–1^ h^–1^, respectively (Table 2, entries 10–12). Notably, chain transfer is efficiently suppressed, as evidenced by the small number of chains per nickel formed (3, 5, and 5 at 30, 50, and 70 °C, respectively); consequently, ultrahigh-molecular-weight polyethylene with a rather narrow molecular weight distribution was obtained (*M*_n_ = 2062 kg mol^–1^, *M*_w_/*M*_n_ = 1.59 at 30 °C and *M*_n_ = 1235 kg mol^–1^, *M*_w_/*M*_n_ = 1.87 at 50 °C). Compared to catalyst precursors **2**, **3**, and **4**, complex **5** showed a similar polymerization activity but much higher propensity for chain transfer (chains per nickel 50, 175, and 854 at 30, 50, and 70 °C, respectively. Table 2, entries 13–15), despite the strongly electron-donating substituted 2,4,6-tri(isopropyl)phenyl group. BHE by the catalyst from **5** also reflects in observable branch formation (0.6, 1.0, and 1.2 methyl branches per 1000C at 30, 50, and 70 °C, respectively. cf. Figures S49–S51 for representative ^13^C NMR spectra) and a slightly lower melting point *T*_m_ (entries 14 and 15), as determined by differential scanning calorimetry (DSC) (Figures S52–S65). Complex **6** with a C_6_F_5_-substitution in the ortho-position of the phenolate showed a lower catalytic activity than that of **4** but maintained the low propensity for chain transfer reactions (Table 2, entries 16 and 17).

**Figure 3:**
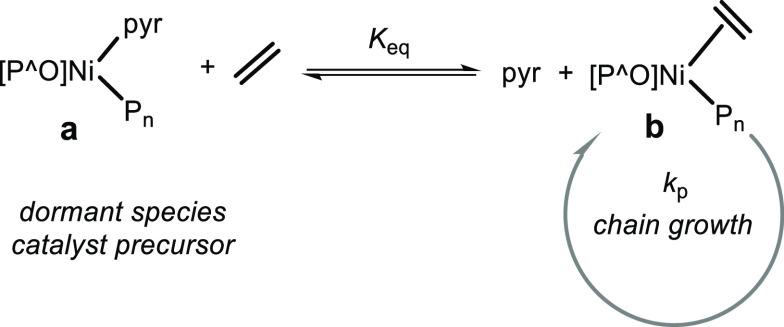
Pyridine vs ethylene binding during polymerization.

In the ethylene polymerization by such neutral Ni(II) catalysts, the pyridine liberated from the catalyst precursor upon the formation of active species can compete with ethylene for coordination to the Ni center, which results in a rapid reversible equilibrium (Figure 3). The relative binding of pyridine is much stronger than that for ethylene, and this effect is offset by the typically much higher ethylene concentration. The relative binding constants and actual chain growth rate for **1** and **2** were estimated from activities observed in a series of experiments with variable amounts of added pyridine by plotting the reciprocal activity vs the pyridine concentration (cf. the Supporting Information for details).^14,39^ This yielded *K*_eq_ (30 °C) = 5.5 × 10^–7^ and *k*_p_ (30 °C) = 1.9 × 10^5^ (**1**) and *K*_eq_ (30 °C) = 4.3 × 10^–6^ and *k*_p_ (30 °C) = 2.8 × 10^5^ (**2**). That is, the observed higher polymerization activity of **2** vs that of **1** in the above polymerizations (Table 2) is in part a result of a slightly higher chain growth rate (*k*_p_) but primarily originates from the more facile dissociation of pyridine. Relative binding of the olefin vs that of the harder N-donor is more favorable for the less electron-deficient nickel site. This becomes notable particularly in polymerizations at 30 °C (Table 2, entries 4, 7, 10, 13 vs 1). At higher temperatures, the equilibrium constants *K*_eq_ and the ratio of *K*_eq_s for different catalysts are expected to be lower, which accounts for the smaller differences in observed polymerization activities at 50 and 70 °C. To further corroborate the role of pyridine binding in these catalysts, **1-tmeda** (tmeda = *N*,*N*,*N*′,*N*′-tetramethylethylenediamine) with a more labile amine ligand^40,41^ was synthesized. The observed catalytic activity at 30 °C with this catalyst precursor was indeed significantly higher (6.26 × 10^6^ g mol_Ni_^–1^ h^–1^) than that for **1** and similar to that of **2** (Table 2, entries 19 to 21), although the molecular weight of the obtained polyethylene was still low owing to the extensive chain transfer reaction.

### Mechanism of Chain Transfer

The mechanism of chain transfer and the origin of the advantageously low chain transfer with electron-rich substituted 2-(aryl)phenyls were elucidated by density functional theory (DFT) studies of catalysts **1** and **2**.

**Scheme 1:**
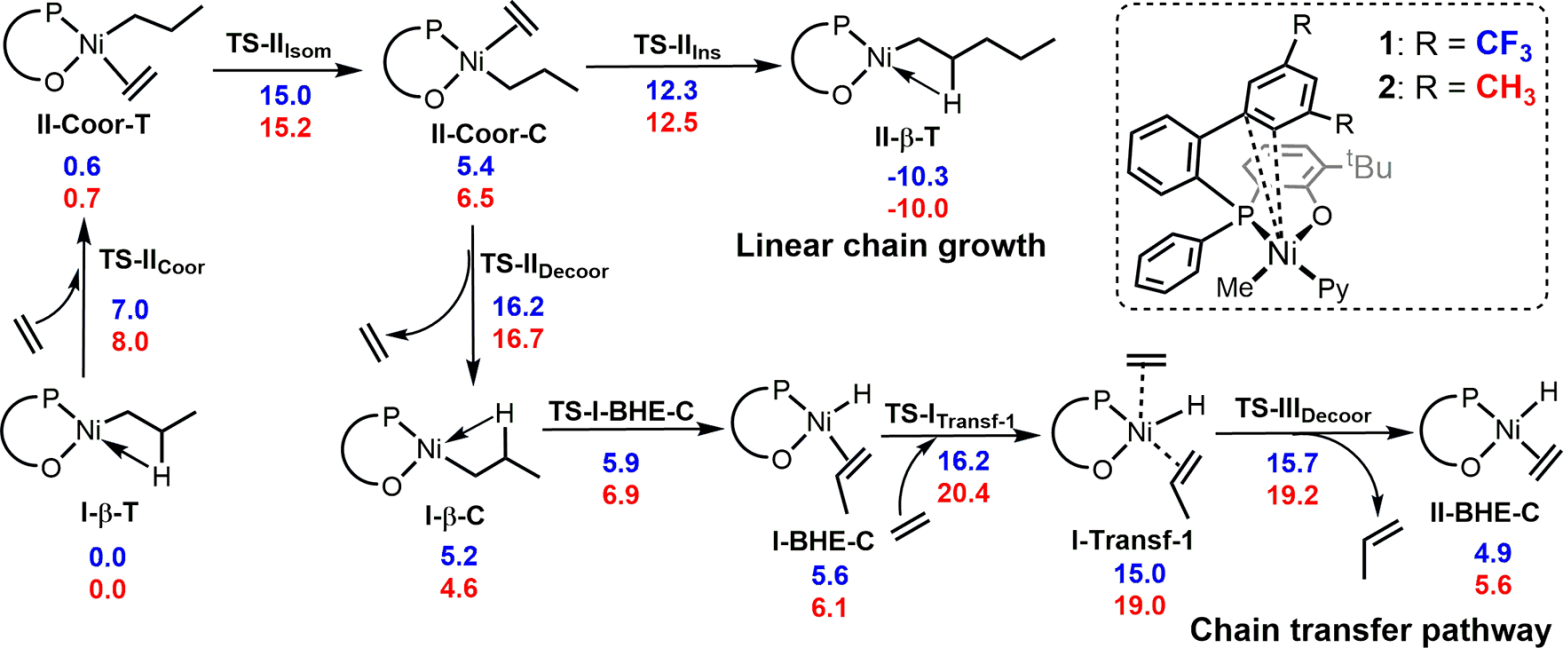
Gibbs Energies (Δ*G*_Tol_ in kcal/mol) of Key Species of Linear Chain Growth and Chain Transfer for Catalysts **1** and **2**

In the starting point **I-β-T** (referenced as the zero-point energy, Scheme 1), with the growing alkyl chain *trans* to the oxygen atom, a stabilizing η^2^ interaction of the aryl ring with the metal center is also found. The optimized geometry (see the Supporting Information) shows the arene to be planar and placed in the apical position of the metal with the whole ring interacting with Ni (see short distances between Ni and C_ipso_, C_ortho_, and C_meta_ in Figure S78). A more detailed description of this Ni–aryl ring interaction is reported in the Supporting Information.

From **I-β-T**, the coordination of ethylene to form **II-Coor-T** occurs by opening the β-agostic interaction through a transition state **TS-II**_**Coor**_ with a similar free energy barrier for both **1** and **2** (7.0 and 8.0 kcal/mol, respectively). The following intermediate is slightly higher in energy relative to that of **I-β-T** + C_2_H_4_ and is isoenergetic for both catalysts. From **II-Coor-T**, the favored linear chain growth pathway comprises the isomerization via **TS-II**_**Isom**_ to the less stable π-complex **II-Coor-C**, followed by monomer insertion via **TS-II**_**Ins**_ to form **II-β-T**. The two catalysts behave similarly along the entire linear chain growth pathway, with the decisive isomerization energy barrier amounting to ca. 15 kcal/mol, in agreement with the comparable experimentally observed chain growth rates.

From the key intermediate **II-Coor-C** (also cf. Figure S77), monomer decoordination competes with the aforementioned monomer insertion reaction. In the product of monomer decoordination (Scheme 1), the β-agostic intermediate **I-β-C**, the Ni–aryl interaction is still maintained. Ethylene dissociation takes place via a single-step (**TS-II**_**Decoor**_) process with a similar energy barrier for the two catalysts (16.2 and 16.7 kcal/mol for **1** and **2**, respectively). As a consequence, the ethylene decoordination step is not the decisive step^42,43^ for the microstructure and molecular weights of the polyethylene obtained.

From **I-β-C**, BHE occurs with an energy barrier of about 6 kcal/mol for both catalysts, leading to the Ni–H intermediate **I-BHE-C**. Starting from **I-BHE-C**, the favored chain-transfer pathway consists of the coordination of new monomer via **TS-I**_**Transf-1**_ to form a pentacoordinate intermediate **II-Transf-1** that quickly releases propene, via **TS-III**_**Decoor**_, affording the hydride ethylene complex **II-BHE-C**. The first step is rate determination along the chain-transfer pathway for both catalysts, with an energy barrier higher by 4.2 kcal/mol for **2** compared to that for **1** (Scheme 1). In **TS-I**_**Transf-1**_, the incoming monomer coordinates in the apical position above the metal, displacing the Ni–aryl interaction present in all of the major species along the pathways considered. This interaction is stronger in **2** than in **1** due to the presence of electron-donating substituents on the ring (as evidenced also by the shorter Ni–aryl distances in **2** than those in **1**, Figure 1), and thus, it is more difficult to break, causing an increase in the energy of **TS-1**_**Transf-1**_. This agrees with the experimentally observed higher polyethylene molecular weight obtained with **2**.^44^

**Scheme 2:**
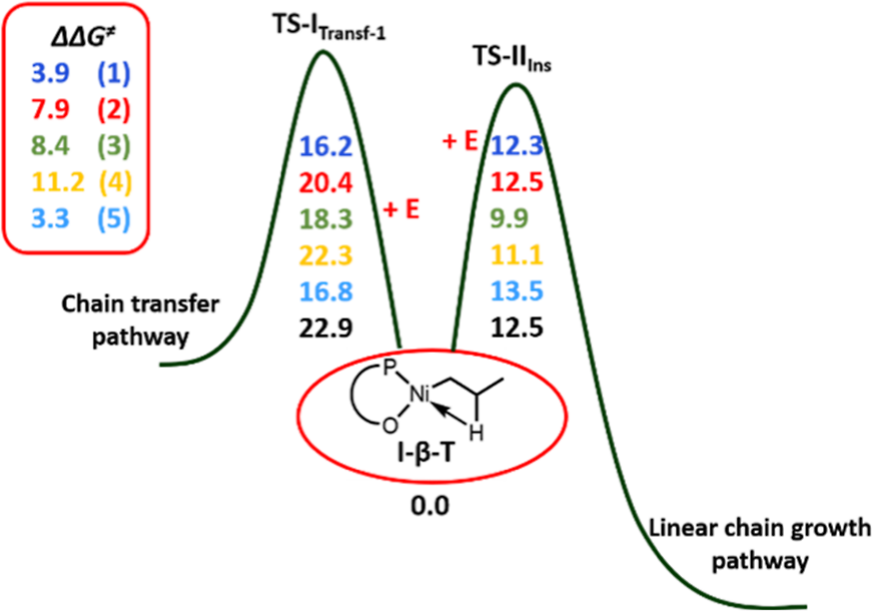
ΔΔ*G*^⧧^ as a Measure for the Relative Propensity for the Chain Growth Pathway versus Access to the Chain-Transfer Pathway for Catalysts **1**, **2**, **3**, **4**, and **5**

Considering the entire series of catalysts, we expect that the strength of the Ni–aryl interaction increases with the electron density on the aryl ring in the order **1** < **2** < **3** < **4** ≈ **6** with a consequent suppression of the chain transfer reaction to a greater extent. The calculated ΔΔ*G*^⧧^ between the determining steps of the linear chain growth and the chain transfer follows this trend (see Schemes 2 and S1), in agreement with the experimental results (see Table S11).

The only exception is found for complex **5**, for which the steric hindrance of the three bulky isopropyl groups on the distal ring overrules this electronic effect, weakening the Ni–aryl interaction (cf. the long Ni–aryl distance and large angle of C24 with the adjacent phenyl in **5**, Figure 1). As a consequence, for catalyst **5**, the Gibbs energy barrier to the chain transfer resembles that for catalyst **1** (Scheme 2).

## Conclusions

The combined in-depth experimental and theoretical study reveals the origin of the ability of the privileged phosphinephenolato Ni(II) catalyst structure to suppress chain transfer, this being the key to their unique polymerization properties.^17^ Different from the prevailing view established for other catalyst systems of axial steric shielding being instrumental to suppress chain transfer, the electronic nature of an axially arranged aryl and its ability for an attractive Ni–aryl interaction with the metal center are decisive here. The latter is dependent on the nature of the substituents on this aryl group in the 2-position of the *P*-phenyl. Electron-donating substituents promote suppression of chain transfer and formation of a high-molecular-weight polymer. This role of substituents is also underlined by the demonstration of this desirable effect by electron-donating but extremely bulky isopropyl substituents, which hinder the Ni–aryl interaction.

This understanding and rationale will promote the further development of this unique class of catalysts and may enable the finding of novel even superior catalyst systems capable of copolymerization of polar monomers, generation of in-chain functional groups, and aqueous polymerizations.

## Supporting Information Available

General considerations, experimental details, synthesis, characterization, CV, and X-ray structure data of ligands and complexes; polymerization procedure; NMR spectra, DSC traces, and GPC data of the obtained polymers; mass flow of ethylene consumed in polymerizations; results of ethylene polymerization by the Ni catalyst with additional pyridine; and calculation details and Cartesian coordinates (PDF)

## Accession Codes

CCDC 2264071–2264076 contain the supplementary crystallographic data for this paper. These data can be obtained free of charge via www.ccdc.cam.ac.uk/data_request/cif, or by emailing data_request@ccdc.cam.ac.uk, or by contacting The Cambridge Crystallographic Data Centre, 12 Union Road, Cambridge CB2 1EZ, UK; fax: +44 1223 336033.
