# Supplementary material for: Origin of Suppressed Chain Transfer in Phosphinephenolato Ni(II)-Catalyzed Ethylene Polymerization
Source: J Am Chem Soc. 2023 Dec 16;145(51):27950–7. doi: 10.1021/jacs.3c06597 (PMC10755696; doi:10.1021/jacs.3c06597)
Supplement: Supplementary file 1 — ja3c06597_si_001.pdf [file ja3c06597_si_001.pdf]

Supporting Information

Origin of Suppressed Chain Transfer in Phosphinephenolato Ni(II) Catalyzed Ethylene Polymerization

Fei Lin[†a], Maria Voccia[‡a], Lukas Odenwald[†], Inigo Göttker-Schnetmann[†], Laura Falivene[‡], Lucia Caporaso[‡*] and Stefan Mecking[†*]

[†] Chair of Chemical Materials Science, Department of Chemistry, University of Konstanz, 78457 Konstanz, Germany
[‡] Dipartimento di Chimica e Biologia, Università di Salerno, Via Papa Paolo Giovanni II, I-84084 Fisciano, Italy
[a] contributed equally
[*]Corresponding Authors
Email: stefan.mecking@uni-konstanz.de, lcaporaso@unisa.it

Contents

1 General considerations 4

1.1 Solvents and reagents 4

1.2 Analytical methods and techniques 4

2 Synthesis and characterization of phosphinephenols and complexes 6

2.1 Synthesis and NMR spectra of phosphinephenols **1a-5a** and complexes **1-5**. 6

2.2 Synthesis and NMR spectra of phosphinephenol **6a** and complex **6**. 32

2.3 Crystallographic Details 38

2.4 Cyclic Voltammetry 47

3 Ethylene Polymerization 48

3.1 Ethylene Polymerization Procedure 48

3.2 Mass Flow Profiles of Ethylene Polymerizations 49

4 Polymer Characterization 56

4.1 NMR Analysis 56

4.2 DSC traces 58

4.3 GPC Traces 65

5 Estimation of pyridine binding equilibria and chain growth rate 70

5.1 Polymerizations with added pyridine 70

5.2 Data analysis 71

6 Computational Details 72

6.1 Selected Intermediates and Transition States 72

6.2 General method 73

6.3 Gibbs energies of competitive species for catalyst **2** with different computational protocols. 74

6.4 Gibbs energies of key species for catalysts **1-5**. 74

6.5 Supplementary tables 74

6.6 Non-covalent interaction analysis. 77

6.7 Cartesian Coordinates 80
7 Supplementary References... 187

1 General considerations

Unless noted otherwise, all manipulations of air and moisture sensitive materials were carried out under inert gas atmosphere using standard glovebox and Schlenk techniques.

1.1 Solvents and reagents

Solvents were dried and degassed using standard laboratory techniques. THF was distilled from sodium benzophenone ketyl, benzene from sodium, dichloromethane was distilled from CaH_2 and pentane and toluene were dried and degassed by passing through columns equipped with alumina and BASF R3-11 catalyst. *n*-Butyllithium (*n*-BuLi; 2.5 M solution in *n*-hexane), 2-tert-butylphenol (≥99%), dichlorophenylphosphine, 3,4-dihydro-2H-pyran (≥97%), and hexafluorobenzene were purchased from Sigma-Aldrich or ABCR. Ethylene (grade 3.5) and hydrogen (grade 5.0) was supplied by Air Liquide and used as received. [(tmeda)$NiMe_2$],[1] 2-bromo-3',5'-bis(trifluoromethyl)-1,1'-biphenyl, 2-bromo-3',5'-dimethyl-1,1'-biphenyl,[2] 2-bromo-3',5'-dimethoxy-1,1'-biphenyl,[3] 2'-bromo-2,4,6-trimethoxy-1,1'-biphenyl,[4] 2'-iodo-2,4,6-triisopropyl-1,1'-biphenyl,[5] 2-phenoxytetrahydro-2H-pyran[6] and 2-(2-(tert-butyl)phenoxy)tetrahydro-2H-pyran[7] were synthesized following reported procedures. All other commercially available reagents and starting materials were supplied by Sigma Aldrich, Acros, ABCR or Activate Scientific. $CDCl_3$ was supplied by Sigma Aldrich, all other deuterated solvents by Eurisotop.

1.2 Analytical methods and techniques

NMR spectra were recorded on a Bruker Avance III HD 400 (^1H: 400.1 MHz, ^{13}C: 100.6 MHz, ^{19}F: 376.1 MHz), a Bruker Avance III 400 or a Bruker Avance III 600 spectrometer (^1H: 600 MHz, ^{13}C: 151 MHz). ^1H chemical shifts were referenced to the solvent's residual proton signals ($CDCl_3$: 7.26 ppm, $C_2D_2Cl_4$: 5.91 ppm, C_6D_6: 7.16 ppm). ^{13}C chemical shifts were referenced to the carbon signal of the deuterated solvent ($CDCl_3$: 77.16 ppm, $C_2D_2Cl_4$: 74.30 ppm, C_6D_6: 128.06 ppm). ^{19}F chemical shifts were referenced to external $BF_3 \cdot OEt_2$. NMR spectra of polyethylenes were recorded with addition of 5 mg mL^{-1} of $Cr(acac)_3$ as paramagnetic relaxation agent.

DSC measurements were carried out on a Netzsch DSC 204 F1 instrument (software: Netzsch Proteus Thermal Analysis, version 6.1.0) on ca. 5 mg of polymer with heating and cooling rates of

10 K min^{-1} under a nitrogen atmosphere. Crystallinities were determined assuming a melt enthalpy of 293 J g^{-1} for 100% crystalline polyethylene.

GPC was performed on a PolymerChar GPC-IR instrument equipped with PSS Polefin Linear XL columns (3 × 30 cm, additional guard column), an infrared detector (IR5 MCT) and a four-capillary viscometer at 160 °C in 1,2-dichlorobenzene at 1.0 mL min^{-1} flow rate. A reduced flow rate of only 0.5 mL min^{-1} was used for samples with $M_n > 10^5$ g mol^{-1} to avoid sample shearing on the column. Sample molecular weights were analyzed via universal calibration employing narrow polystyrene standards (PSS Polymer Standards). The raw data was evaluated with PSS WinGPC UniChrom software.

X-ray diffraction analysis were performed at 100 K on a STOE IPDS-2T diffractometer, equipped with a graphite-monochromated radiation source (λ = 0.71073 Å) and an image plate detection system. Crystals were placed on a fine glass fiber with grease or oil. The selection, integration and averaging procedure of the measured reflex intensities, the determination of the unit cell dimensions and a least-squares fit of the 2Θ values as well as data reduction, LP-correction and space group determination were performed using the X-Area software package delivered with the diffractometer.[8] Using Olex2,[9] the structure was solved with the ShelXT[10] structure solution program using intrinsic phasing and refined with the olex2[11] refine refinement package using Gauss-Newton minimization. Graphical representations were created by the ORTEP-3 V2.02. for Windows XP software package.[12]

Topographical maps of complexes **1**, **2**, **3**, **4**, **5** and **6**, as well as corresponding percent buried volume data (%V_{bur}) were generated by Cavallo's SambVca 2.1 (Salerno molecular buried volume calculation) program.[13-15] In detail, for %Vbur calculation and in steric maps: 1) The nickel atom defines the center of the xyz coordinate system; 2) the Ni(pyr)Me fragment was excluded; 3) Bondi radii were scaled by 1.17; 4) The sphere radius was set to 3.5 Å; 5) The distance of the coordination point from the center of the sphere was set to 0.0 Å; 6) Mesh spacing for numerical integration was 0.10; 6) H atoms were included in the calculations. For all complexes, xz-plane and the z-axis were defined following the methods for the Unsymmetric complex in SambVca 2.1-Library. The x- or y-axis is flipped so the larger axial shielding locates on the top and the substitute *ᵗ*Bu- or C_6F_5

on the ortho-position of phenolate locates on the right. Note that for a specific complex, the %Vbur remained the same with Ni in the origin even if the xyz coordination system rotated or flipped.

Cyclic voltammetry (CV) measurements were performed in a custom-built (Rainer Winter group at the University of Konstanz) one-compartment cell. A spiral-shaped Pt wire and an Ag wire as the counter and reference electrodes are sealed into glass capillaries that are introduced via Quickfit screws at opposite sides of the cell. A Pt electrode is introduced as the working electrode through the top port. It is polished with first 1 μm and then 0.25 μm diamond paste (Buehler-Wirtz) just before measurements. The experiments were performed with approximately 5 mL of dichloromethane. $^{n}Bu_4NPF_6$ was used as supporting electrolyte. Referencing was performed with addition of $[(Me_5Cp)_2Fe]$ as an internal standard after all data have been acquired. The scans were repeated with internal standard. Final referencing was performed against ferrocene/ferrocenium ($Cp_2Fe^{0/+}$) with $E_{1/2}(Cp_2Fe^{0/+})$ = -550 mV vs. $Cp_2Fe^{0/+}$. Electrochemical data were acquired with a computer-controlled BASi potentiostat.

2 Synthesis and characterization of phosphinephenols and complexes

2.1 Synthesis and NMR spectra of phosphinephenols **1a-5a** and complexes **1-5**.

The synthesis of compounds **1a-5a** employed a modification of a reported procedure.[16] At room temperature n-BuLi (1.3 mL, 2.5 M in hexane, 3.3 mmol, 1.1 equiv) was added dropwise to a solution of 2-(2-(tert-butyl)phenoxy)tetrahydro-2H-pyran (0.7 g, 3 mmol, 1.0 equiv.) in diethyl ether (10 mL) at 0 °C. The reaction mixture was stirred for 2 hours to give a white suspension. The obtained suspension was added dropwise to a solution of $PhPCl_2$ (0.59 g, 3.3 mmol, 1.1 equiv.) in diethyl ether (10 mL) at -78 °C. The mixture was warmed to room temperature slowly over 8 hours to obtain a white suspension **A**. In another Schlenk flask, n-BuLi (1.3 mL, 2.5 M in hexane, 3.3 mmol, 1.1 equiv) was added dropwise to a solution of the corresponding diaryl bromide or iodide (3.3 mmol, 1.1 equiv.) in THF (20 mL) at -78 °C. The reaction mixture was stirred for two hours, to yield a suspension. Then, the suspension **A** was added to suspension **B** at -78 °C, and the reaction mixture was warmed to room temperature slowly over 8 hours. The mixture was concentrated in vacuo, the residue was dissolved in 10 mL of degassed ethyl acetate, and 1 mL of conc. HCl was

injected. The reaction mixture was stirred at room temperature overnight, added slowly into a solution of 5 g $NaHCO_3$ in 30 mL H_2O, and stirred for 30 minutes. The organic phase was separated, and the aqueous phase was extracted with ethyl acetate (2 × 20 mL). The combined organic phases were concentrated in vacuo, and the residue was subjected to column chromatography on silica using a petrol ether/ethylene acetate mixture as eluent to give the pure phosphinephenol.

General procedure for the synthesis of complexes **1** to **5**: To [(tmeda)$NiMe_2$] (1.05 equiv.) and the phosphinephenol (100 μmol, 1 equiv.), a solution of pyridine (25 equiv.) in 5 mL of benzene was added. Gas evolution (methane) was observed. After the reactants were stirred for 4 hours at room temperature, volatiles were removed in vacuo. The residue was dispersed with benzene again, and the formed nickel black formed during the reaction was removed via centrifugation. The yellow solution was frozen in liquid nitrogen und the solvent removed by freeze drying to give the desired product as a yellow powder.

Procedure for the synthesis of **1-tmeda**: To [(tmeda)$NiMe_2$] (1.05 equiv.) and the phosphinephenole **1a** (100 μmol, 1 equiv.), 5 mL of benzene was added. Gas evolution (methane) was observed. After stirring for 4 hours at room temperature, volatiles were removed in vacuo. The residue was dispersed with benzene again, and nickel black formed during the reaction was removed via centrifugation. The yellow solution was frozen in liquid nitrogen und the solvent removed by freeze drying to give the desired product as a yellow powder.

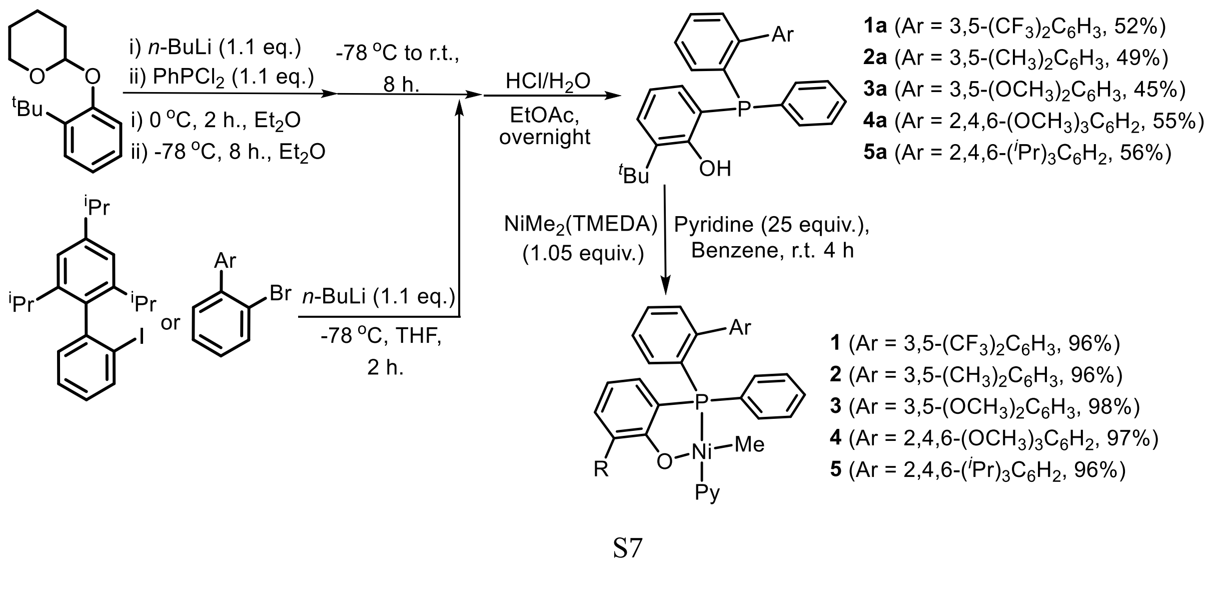

2-(2-(3',5'-(CF_3)$_2C_6H_3$)C_6H_4)Ph-6-^{t}Bu-C_6H_3OH (1a) was obtained following the general procedure, employing 2-bromo-3',5'-bis(trifluoromethyl)-1,1'-biphenyl. Yield: 0.852 g, 1.56 mmol, 52%

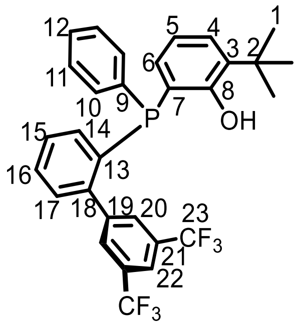

^{1}H NMR (400 MHz, Benzene-d_6) δ 7.65 (s, 1H, 22-H), 7.51 (s, 2H, 20-H), 7.22-7.16 (m, 3H, 10- and 4-H), 7.15 – 7.09 (m, 1H, 16-H), 7.04 – 6.89 (m, 5H, 11-, 12-, 14-H and OH), 6.79 (ddd, *J* = 7.6, 4.7, 1.6 Hz, 1H, 6-H), 6.71 (ddd, *J* = 7.4, 4.5, 1.1 Hz, 1H, 15-H), 6.67 (d, *J* = 11.4 Hz, 1H, 17-H), 6.60 (t, *J* = 7.7 Hz, 1H, 5-H), 1.38 (s, 9H, 1-H).

^{13}C{^{1}H} NMR (101 MHz, Benzene-d_6) δ 158.16 (d, *J* = 21.2 Hz, C8), 143.36 (d, *J* = 22.0 Hz, C18), 143.36 (d, *J* = 4.6 Hz, C19), 136.24 (d, *J* = 1.3 Hz, C16), 135.32 (d, *J* = 6.7 Hz, C3), 134.18 (d, *J* = 19.8 Hz, C10), 133.00 (d, *J* = 1.7 Hz, C4), 132.87 (d, *J* = 2.3 Hz, C15), 132.79 (C7), 130.91 (q, *J* = 33.2 Hz, C21), 130.03 (d, *J* = 4.0 Hz, C17), 129.46 – 129.32 (m, C20), 129.39 (d, *J* = 22.7 Hz, C6), 128.85 (C9), 128.67 (d, *J* = 7.9 Hz, C11), 128.37 (d, *J* = 1.3 Hz, C12), 123.47 (q, *J* = 273.0 Hz, C23), 121.11 – 120.77 (m, C22), 120.54 (d, *J* = 2.3 Hz, C5), 119.15 (d, *J* = 2.1 Hz, C9), 34.71 (C2), 29.24 (C1).

^{19}F{^{1}H} NMR (376 MHz, Benzene-d_6) δ -62.54.

^{31}P{^{1}H} NMR (162 MHz, Benzene-d_6) δ -37.97.

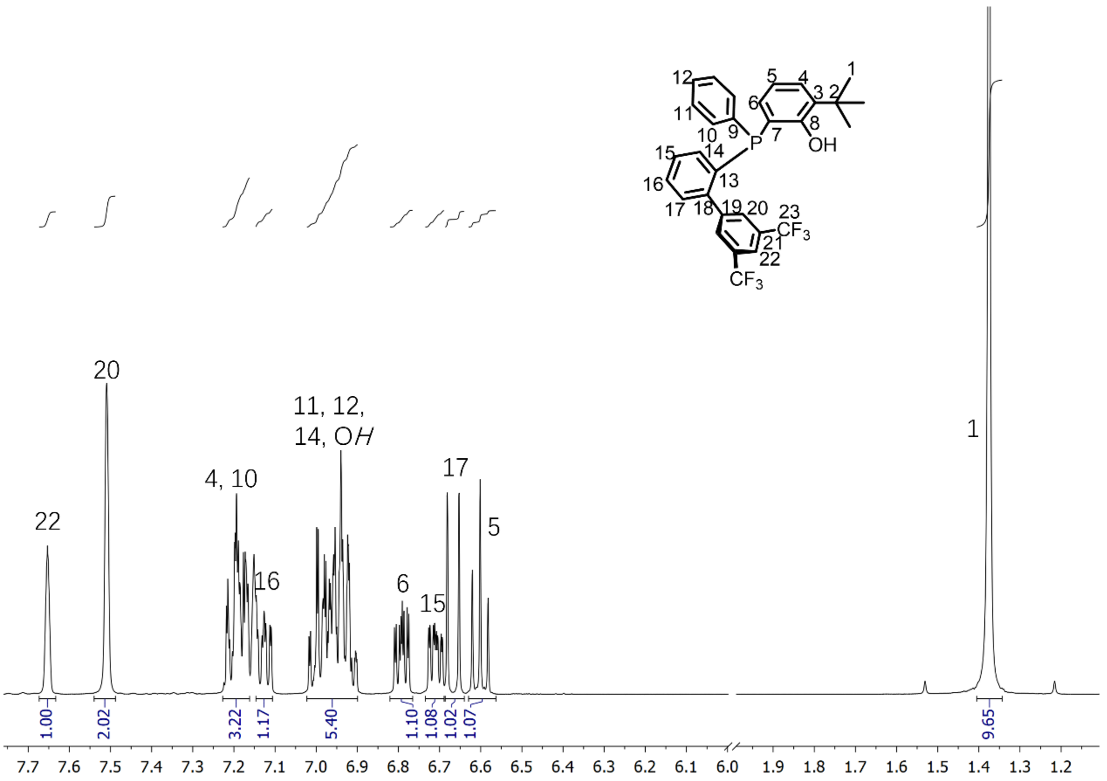

Figure S1. ^{1}H NMR spectrum of phosphinephenol **1a** in C_6D_6 at 300 K.

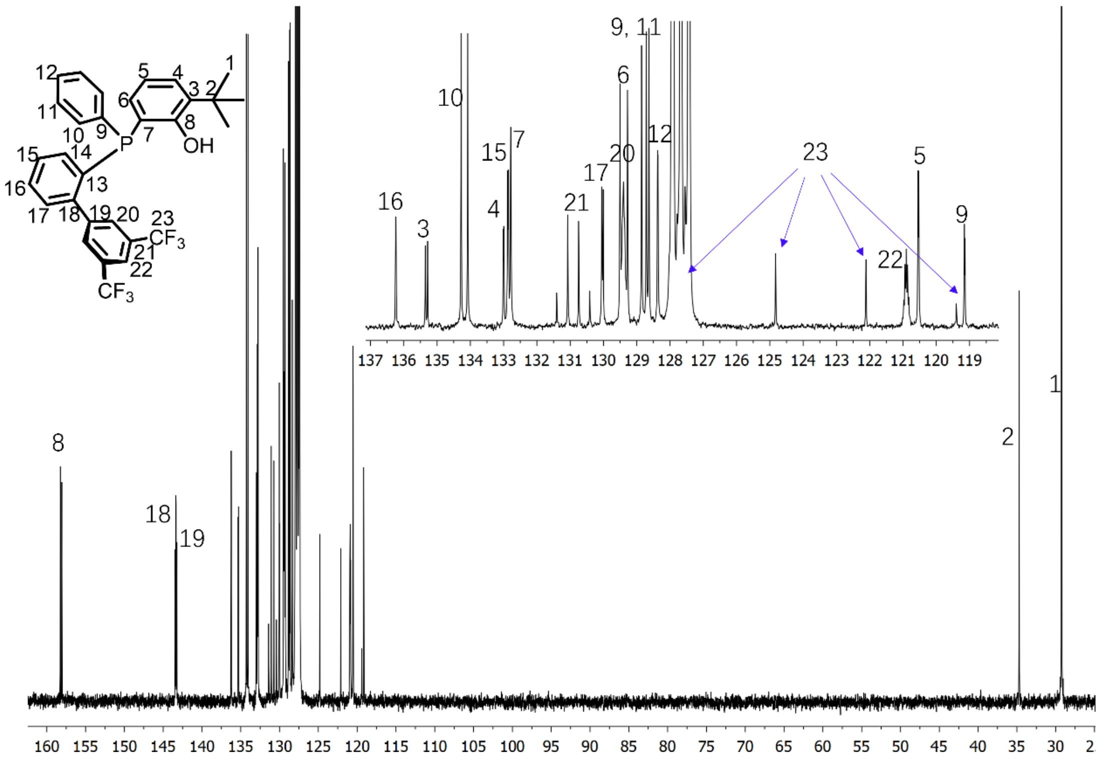

Figure S2. $^{13}C\{^{1}H\}$ NMR spectrum of phosphinephenol **1a** in C_6D_6 at 300 K.

2-(2-(3',5'-$(CH_3)_2C_6H_3)C_6H_4$)Ph-6-^{t}Bu-C_6H_3OH (2a) was obtained following the general procedure, employing 2-bromo-3',5'-dimethyl-1,1'-biphenyl. Yield: 0.63 g, 1.47 mmol, 49%

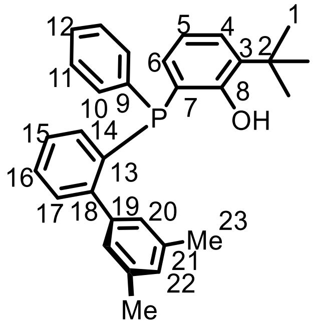

^{1}H NMR (400 MHz, Benzene-d_6) δ 7.34 (ddd, J = 7.7, 4.8, 1.1 Hz, 1H, 14-H), 7.30 – 7.23 (m, 4H, 4-H, 10-H, 15-H), 7.14 – 7.09 (m, 2H, 6-H, 17-H), 7.01 – 6.96 (m, 5H, 11-, 12-H, 16-H, OH), 6.87 (s, 2H, 20-H), 6.77 (s, 1H, 22-H), 6.73 (t, J = 7.6 Hz, 1H, 5-H), 2.12 (s, 6H, 23-H), 1.46 (s, 9H, 1-H).

^{13}C{^{1}H} NMR (101 MHz, Benzene-d_6) δ 158.22 (d, J = 21.5 Hz, C8), 148.36 (d, J = 26.9 Hz, C18), 141.61 (d, J = 6.2 Hz, C9), 136.97 (C20), 135.91 (d, J = 1.2 Hz, C21), 135.82 (d, J = 3.5 Hz, C3), 134.62 (d, J = 4.8 Hz, C14), 133.74 (C16), 133.66 (d, J = 22.1 Hz, C10), 133.03 (C17), 130.15 (d, J = 5.3 Hz, C15), 128.97 (C22), 128.84 (C4), 128.45 (d, J = 7.2 Hz, C11), 128.41 (C12), 127.21(d, J = 0.7 Hz, C19), 121.39 (C7), 120.47 (d, J = 1.8 Hz, C5), 34.78 (C2), 29.52 (C1), 21.03 (C23). C6 and C13 are overlapped by C_6D_6.

^{31}P{^{1}H} NMR (162 MHz, Benzene-d_6) δ -41.77.

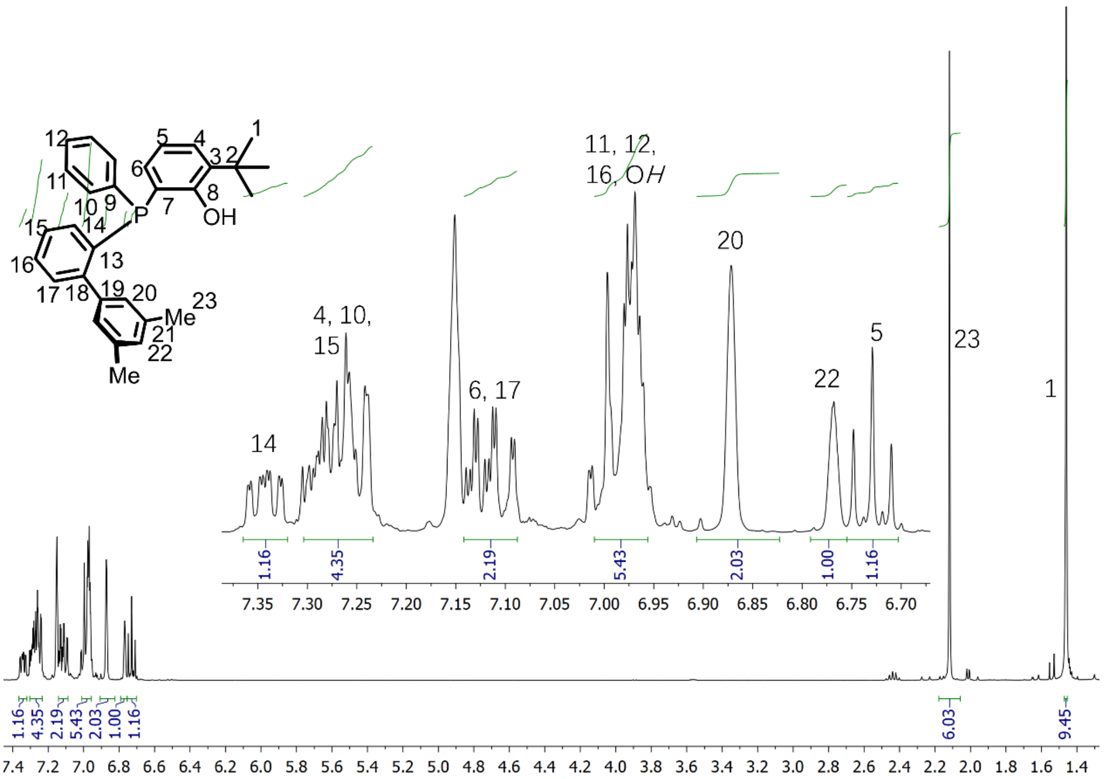

Figure S3. ^{1}H NMR spectrum of phosphinephenol **2a** in C_6D_6 at 300 K.

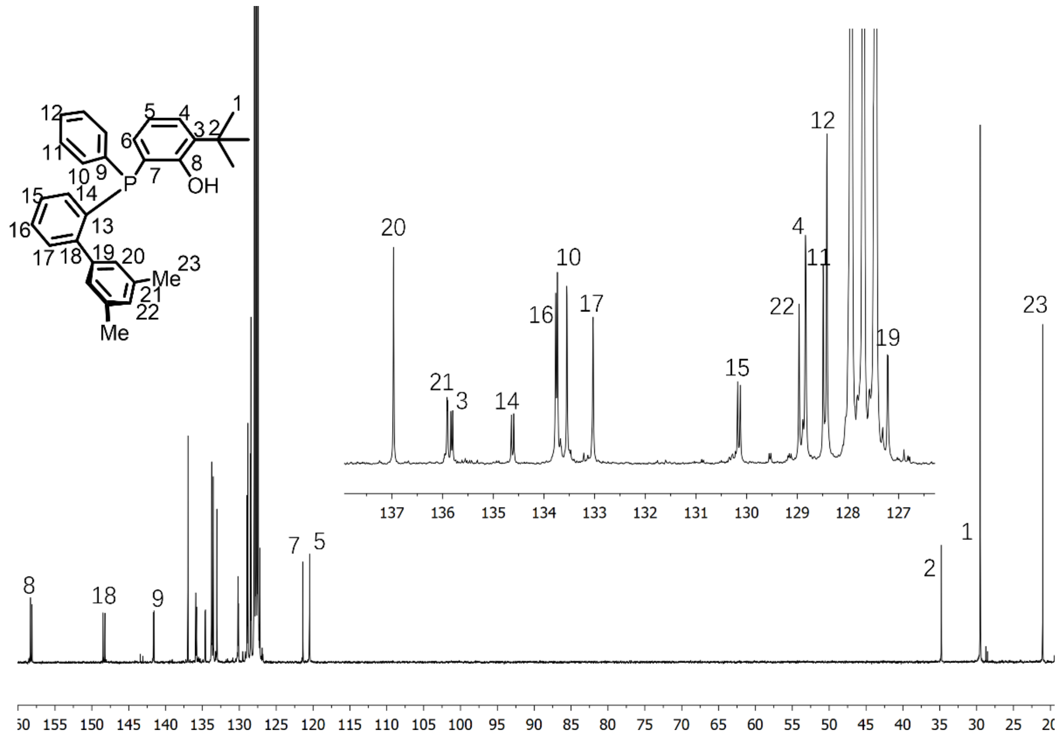

Figure S4. $^{13}C\{^{1}H\}$ NMR spectrum of phosphinephenol **2a** in C_6D_6 at 300 K.

2-(2-(3’,5’-(OCH$_3$)$_2$C$_6$H$_3$)C$_6$H$_4$)Ph-6-^{t}Bu-C$_6$H$_3$OH (3a) was obtained following the general procedure employing 2-bromo-3',5'-dimethoxy-1,1'-biphenyl. Yield: 0.63 g, 1.35 mmol, 45%

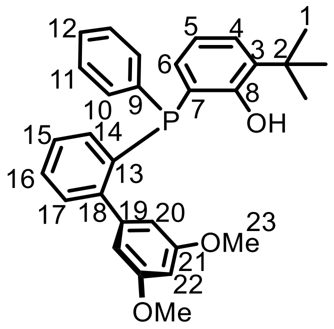

^{1}H NMR (400 MHz, Benzene-d_6) δ 7.38 – 7.22 (m, 5H, 4-, 10-, 14-, 17-H), 7.12-7.05 (m, 2H, 5- and 15-H), 7.02-6.89 (m, 5H, 6-, 11-, 12-H and OH), 6.71 (t, J = 7.5 Hz, 1H, 16-H), 6.57 (s, 1H, 22-H), 6.50 (s, 2H, 20-H), 3.24 (s, 6H, 23-H), 1.46 (s, 9H, 1-H).

^{13}C{^{1}H} NMR (101 MHz, Benzene-d_6) δ 160.60 (C21), 158.26 (d, J = 21.4 Hz, C8), 148.28 (d, $^3J_{CP}$ = 27.4 Hz, C18), 143.61 (d, $^3J_{CP}$ = 6.4 Hz, C17), 136.11 (d, $^4J_{CP}$ = 0.6 Hz, C19), 135.70 (d, $^3J_{CP}$ = 3.8 Hz, C3), 134.29 (d, J = 5.3 Hz, C5), 133.74 (C4), 133.67 (d, $^2J_{CP}$ = 24.2 Hz, C10), 132.98 (C6), 129.97 (d, J = 5.3 Hz, C15), 128.85 (d, $^3J_{CP}$ = 5.6 Hz, C11), 128.52 (C12), 128.46 (d, J = 1.5 Hz, C7), 121.74 (C13), 120.48 (d, J = 1.4 Hz, 16-C), 107.64 (d, $^4J_{CP}$ = 3.1 Hz, C20), 100.41 (C22), 54.45 (C23), 34.79 (C2), 29.45 (C1). The signals for C9 and C14 are overlapped by C$_6$D$_6$.

^{31}P NMR (162 MHz, Benzene-d_6) δ -41.18.

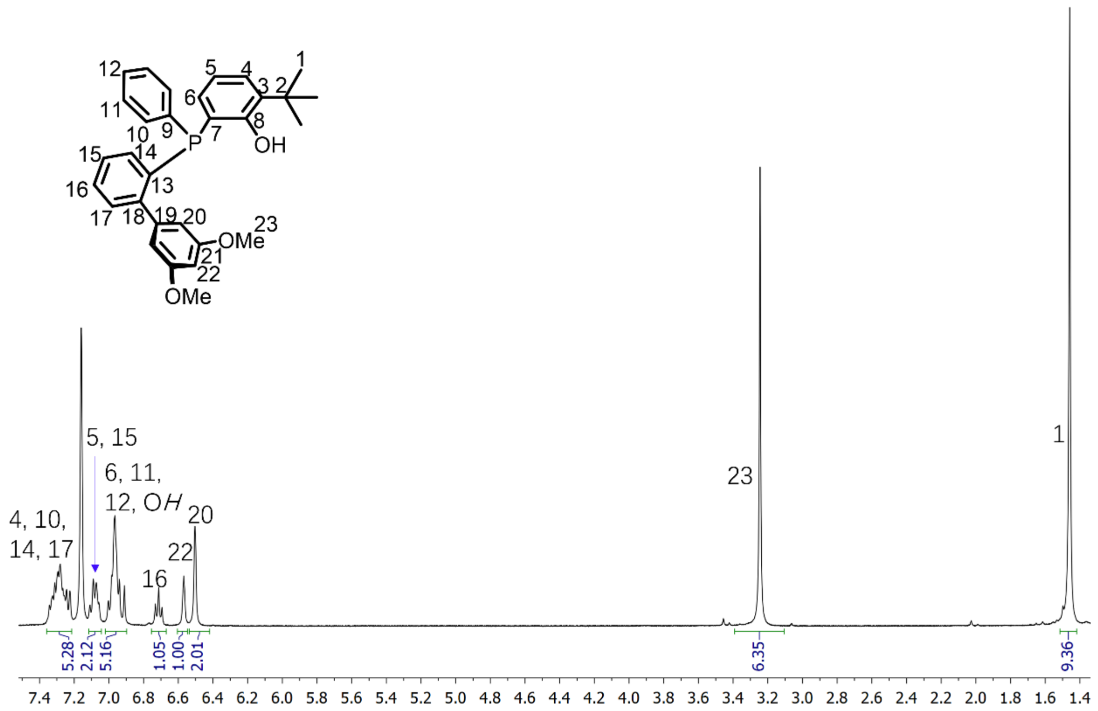

Figure S5. ^{1}H NMR spectrum of phosphinephenol **3a** in C_6D_6 at 300 K.

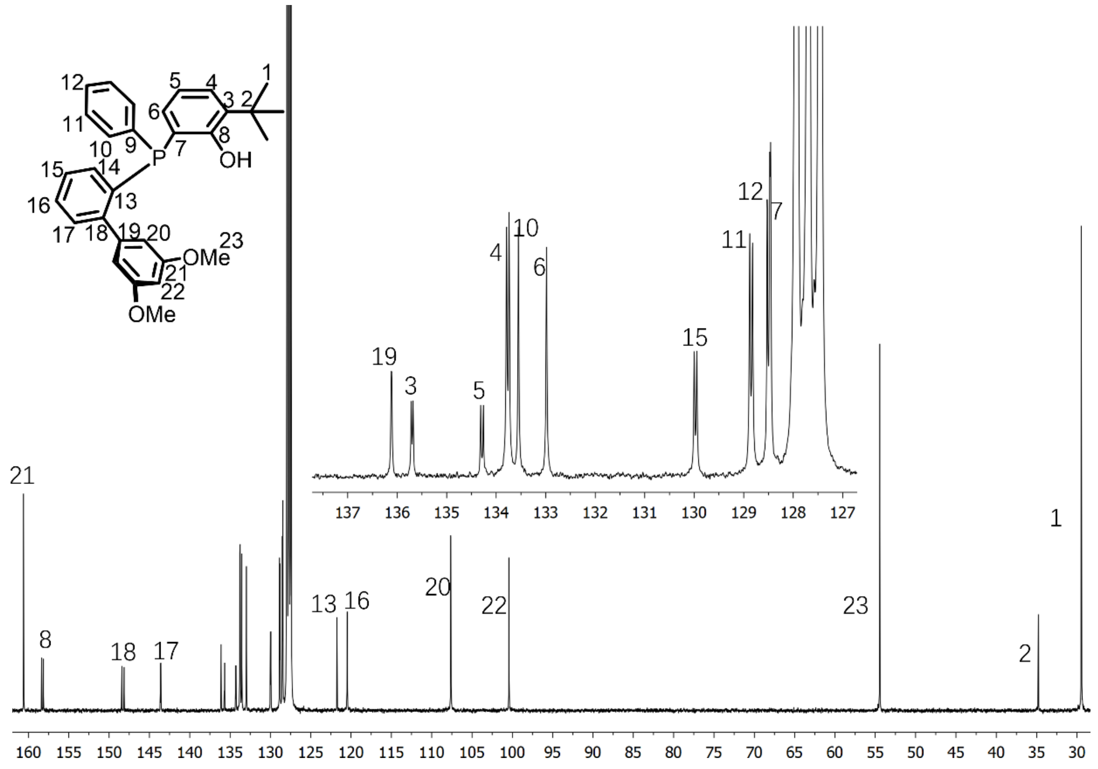

Figure S6. $^{13}C\{^{1}H\}$ NMR spectrum of phosphinephenol **3a** in C_6D_6 at 300 K.

2-(2-(2’,4’,6’-(OCH_3)$_3$$C_6H_2$)$C_6H_4$)Ph-6-^{t}Bu-$C_6H_3$OH (4a) was obtained following the general procedure, employing 2'-bromo-2,4,6-trimethoxy-1,1'-biphenyl. Yield: 0.825 g, 1.65 mmol, 55%

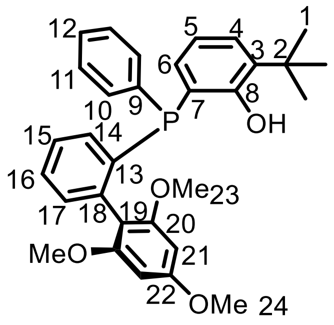

^{1}H NMR (400 MHz, Benzene-d_6) δ 7.48 (ddd, J = 7.6, 4.5, 0.8 Hz,, 1H, 14-H), 7.39 (ddd, J = 7.5, 4.4, 0.8 Hz, 1H, 6-H), 7.36 – 7.28 (m, 2H, 10-H), 7.25 (dd, J = 7.5, 1.0 Hz, 1H, 5-H), 7.19 (m, 2H, 15- and 16-H), 7.11 (ddd, J = 7.5, 4.1, 1.5 Hz, 1H, 5-H), 7.05 – 6.94 (m, 4H, 11-, 17- and OH), 6.74 (t, J = 7.6 Hz, 1H, 6-H), 6.09 (s, 2H, 21-H), 3.39 (s, 3H, 24-H), 3.14 (s, 6H, 23-H), 1.50 (s, 9H, 1-H).

^{13}C{^{1}H} NMR (101 MHz, Benzene-d_6) δ 161.60 (C22), 158.65 (d, J = 0.4 Hz, C20), 158.531 (d, J = 0.5 Hz, C20), 158.40 (d, J = 21.6 Hz, C8), 141.88 (d, J = 32.5 Hz, C18), 136.39 (C4), 136.04 (d, J = 5.7 Hz, C15), 135.44 (d, J = 0.9 Hz, C16), 133.50 (d, J = 18.4 Hz, C10), 133.47 (d, J = 0.8 Hz, C12), 132.70 (C7), 131.97 (d, J = 6.4 Hz, C3), 129.23 (C9), 128.28 (C5), 128.10 (d, J = 6.7 Hz, C11), 122.38 (d, J = 2.9 Hz, C17), 120.07 (d, J = 1.1 Hz, C6), 112.08 (d, J = 8.1 Hz, C19), 90.80 (C21), 90.50 (C21), 54.72 (C23), 54.70 (C23), 54.48 (C24), 34.78 (C2), 29.57 (C1). The signals for C-13 and C-14 are overlapped with C_6D_6.

^{31}P{^{1}H} NMR (162 MHz, Benzene-d_6) δ -39.78.

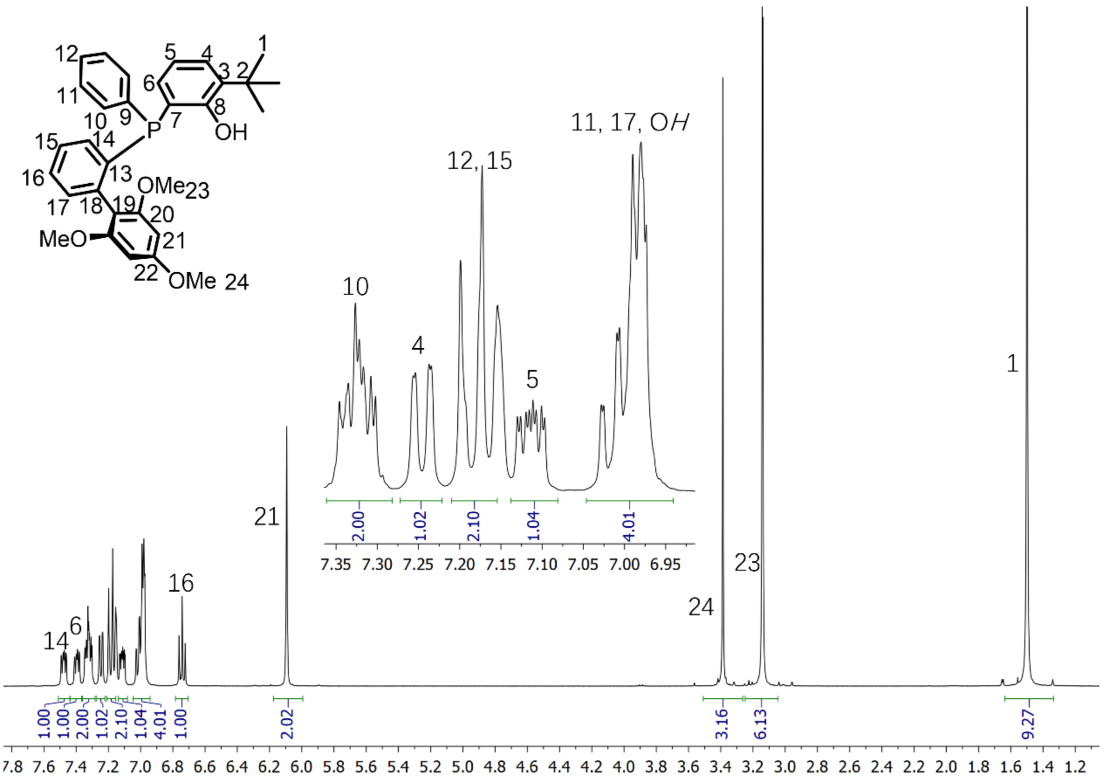

Figure S7. ^{1}H NMR spectrum of phosphinephenol **4a** in C_6D_6 at 300 K.

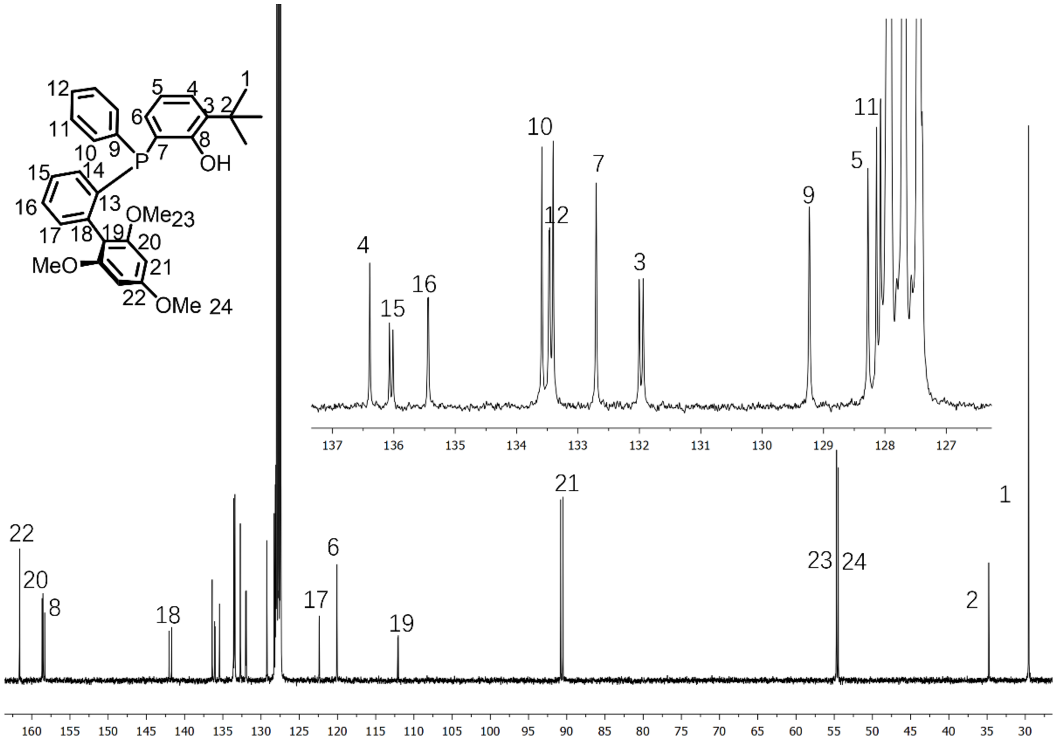

Figure S8. $^{13}C\{^{1}H\}$ NMR spectrum of phosphinephenol **4a** in C_6D_6 at 300 K.

2-(2-(2’,4’,6’-(^{i}Pr)$_3$C$_6$H$_2$)C$_6$H$_4$)Ph-6-^{t}Bu-C$_6$H$_3$OH (5a) was obtain following the general procedure with 2'-iodo-2,4,6-triisopropyl-1,1'-biphenyl. Yield: 0.90 g, 1.68 mmol, 56%

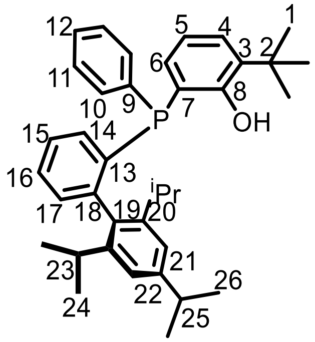

^{1}H NMR (400 MHz, Benzene-d_6) δ 7.41 (ddd, J = 7.7, 4.5, 1.4 Hz, 1H, 5-H), 7.30 – 7.22 (m, 5H, 21-H, 10-H, 14-H and OH), 7.14-7.09 (m, 3H, 12-H, 6-H 15-H), 7.04 – 6.95 (m, 4H, 21-H, 11-H, 4-H), 6.75 (t, J = 7.6 Hz, 1H, 16-H), 6.72 (d, J = 11.9 Hz, 1H, 17-H), 2.97 (h, J = 6.9 Hz,, 1H, 23-H), 2.87 (h, J = 6.9 Hz, 1H, 25-H), 2.13 (h, J = 6.9 Hz, 1H, 23-H), 1.44 (s, 9H, 1-H), 1.40 (d, J = 6.9 Hz, 3H, 24-H), 1.31 (d, J = 3.8 Hz, 3H, 26-H), 1.29 (d, J = 3.8 Hz, 3H, 26-H), 1.14 (d, J = 6.8 Hz, 3H, 24-H), 0.98 (d, J = 6.8 Hz, 3H, 24-H), 0.56 (d, J = 6.8 Hz, 3H, 24-H).

^{13}C{^{1}H} NMR (101 MHz, Benzene-d_6) δ 159.25 (d, J = 22.7 Hz, C8), 148.96 (C22), 147.15 (d, J = 1.1 Hz, C20), 146.98 (C3), 146.66 (C18), 146.17 (d, J = 0.8 Hz, C20), 136.26 (d, J = 3.7 Hz, C7), 136.11 (C4), 136.05 (d, J = 1.2 Hz, C17), 135.62 (d, J = 7.9 Hz, C19), 134.52 (d, J = 1.8 Hz, C5), 133.08 (C6), 132.81 (d, J = 17.8 Hz, C10), 130.73 (d, J = 6.5 Hz, C14), 129.02, 128.73 (C12), 128.44 (d, J = 6.3 Hz, C11), 128.05 (C9), 127.34 (C13), 120.46 (d, J = 1.4 Hz, C21), 120.40 (d, J = 1.5 Hz, C16), 118.93 (C15), 34.74 (d, J = 2.1 Hz, C2), 34.55 (C23), 31.22 (d, J = 2.7 Hz, C25), 30.84 (C23), 29.37 (C1), 26.33 (C26), 25.52 (C24), 24.13 (C24), 22.52 (C24), 22.49 (C24), 21.20 (C26).

^{31}P{^{1}H} NMR (162 MHz, Benzene-d_6) δ -47.48.

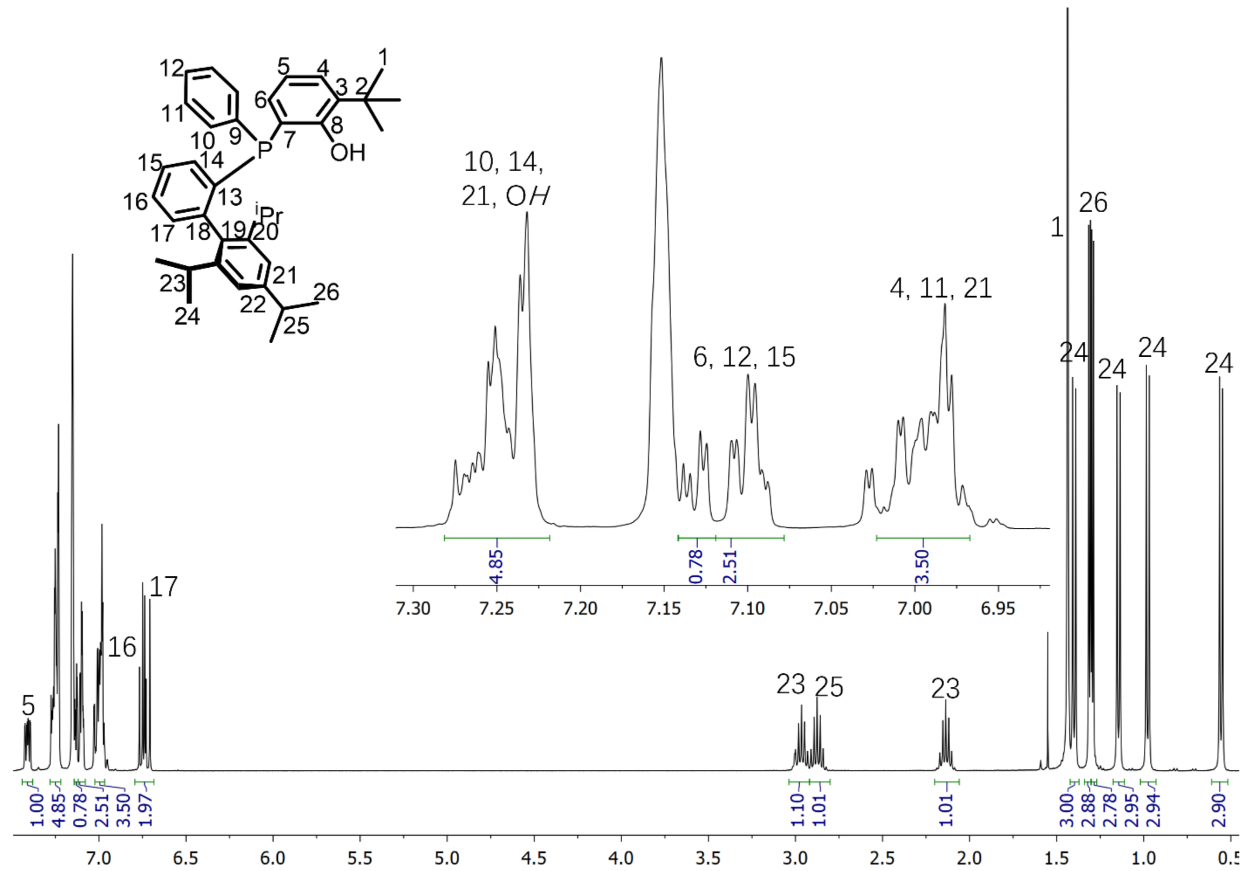

Figure S9. ^{1}H NMR spectrum of phosphinephenol **5a** in C_6D_6 at 300 K.

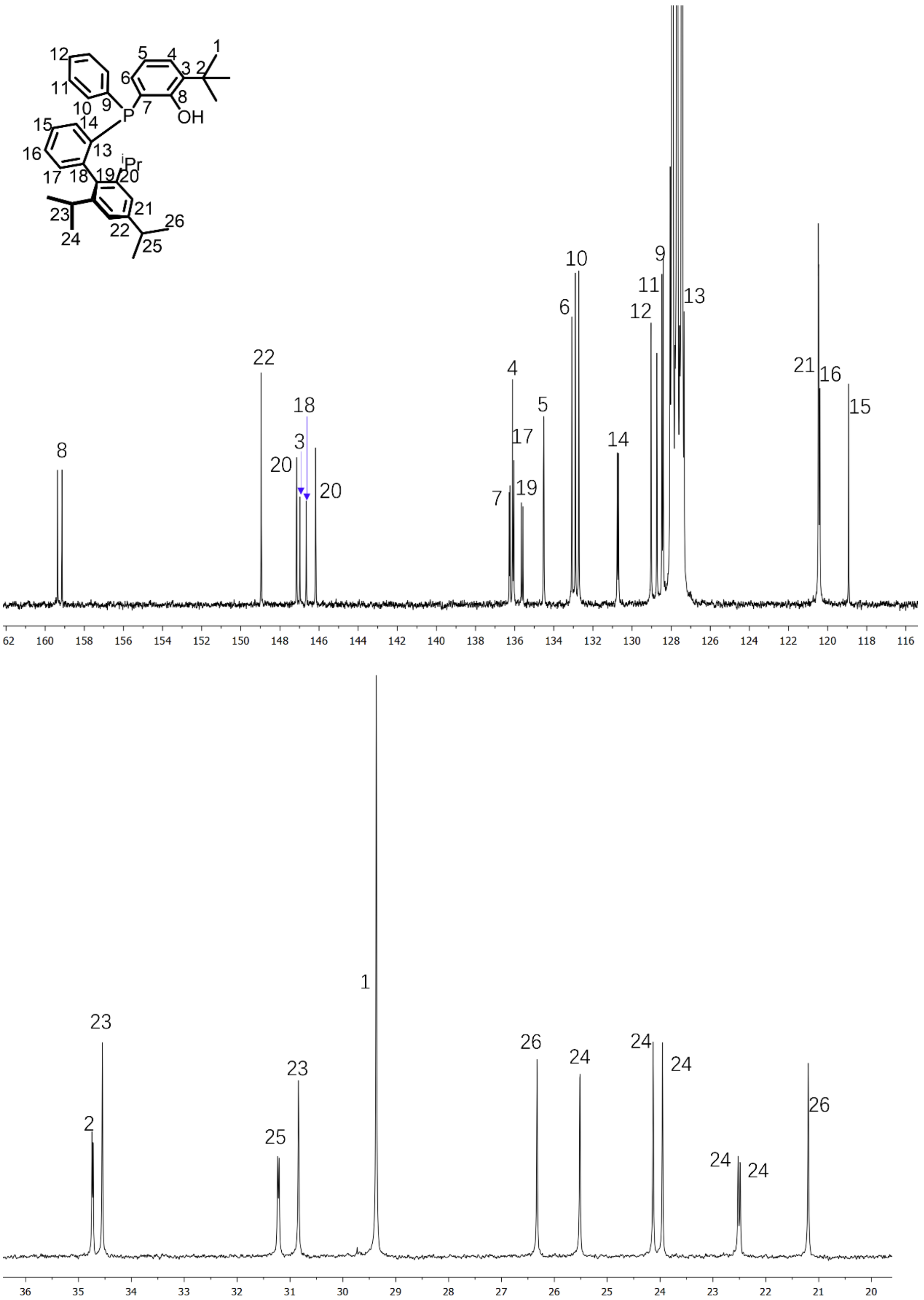

Figure S10. $^{13}C\{^1H\}$ NMR spectrum of phosphinephenol **5a** in C_6D_6 at 300 K, the aromatic region (top) and the aliphatic region (bottom).

2-(2-(3’,5’-$(CF_3)_2C_6H_3)C_6H_4$)Ph-6-^{t}Bu-C_6H_3ONi(II)Me(Pyrdine) (1) was synthesized following the general procedure. Yield: 96 %, 96 μmol, 66 mg.

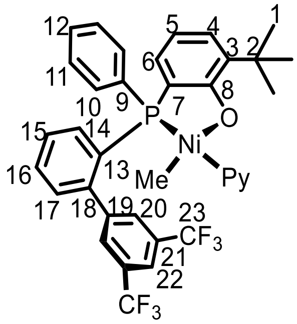

^{1}H NMR (400 MHz, Benzene-d_6) δ 8.51 (s, 2H, *o*-pyr), 7.89 (ddd, *J* = 9.9, 6.6, 3.0 Hz, 2H, 10-H), 7.81 (s, 1H, 22-H), 7.34 (ddd, *J* = 9.8, 7.9, 1.4 Hz, 1H, 14-H), 7.26 (dd, *J* = 7.4, 1.7 Hz, 1H, 4-H), 7.15 (s, 2H, 20-H), 7.11 – 6.99 (m, 4H, 11- , 12- and 16-H), 6.91 (t, *J* = 7.6 Hz, 1H, 15-H), 6.85 (ddd, *J* = 9.2, 7.4, 1.5 Hz, 1H, 6-H), 6.81-6.75 (br, 1H, *m*-pyr), 6.70 (ddd, *J* = 7.6, 4.2, 1.3 Hz, 1H, 17-H), 6.56-6.46 (m, 3H, *p*-pyr and 5-H), 1.41 (s, 9H, 1-H), -0.50 (d, *J* = 4.8 Hz, 3H, Ni-CH_3).

^{13}C{^{1}H} NMR (101 MHz, Benzene-d_6) δ 174.50 (d, *J* = 20.4 Hz, C8), 150.41 (*o*-pyr), 144.69 (d, *J* = 13.5 Hz, C18), 143.65 (d, *J* = 5.1 Hz, C19), 138.75 (d, *J* = 8.9 Hz, C3), 136.94 (*m*-pyr), 134.54 (d, *J* = 3.9 Hz, C14), 133.68 (d, *J* = 10.3 Hz, C10), 132.72 (d, *J* = 48.1 Hz, C9), 131.91 (d, *J* = 8.0 Hz, C17), 131.67 (d, J = 48.5 Hz, C13), 130.77 (q, *J* = 33.1 Hz, C21), 130.38 – 130.20 (C15, C16 and C20), 130.18 (d, J = 12.1 Hz, C6), 129.96 (C4), 128.90 (d, *J* = 9.7 Hz, C11), 128.59 (C12), 124.14 (q, *J* = 273.1 Hz, C23), 123.46 (*p*-pyr), 122.08 – 121.26 (m, C22), 118.96 (d, *J* = 52.8 Hz, C7), 114.27 (d, *J* = 8.0 Hz, C5), 34.99 (d, *J* = 2.1 Hz, C2), 29.62 (C1), -13.64 (d, *J* = 37.3 Hz, Ni-*C*H_3).

^{19}F{^{1}H} NMR (376 MHz, Benzene-d_6) δ -62.02.

^{31}P{^{1}H} NMR (162 MHz, Benzene-d_6) δ 27.38.

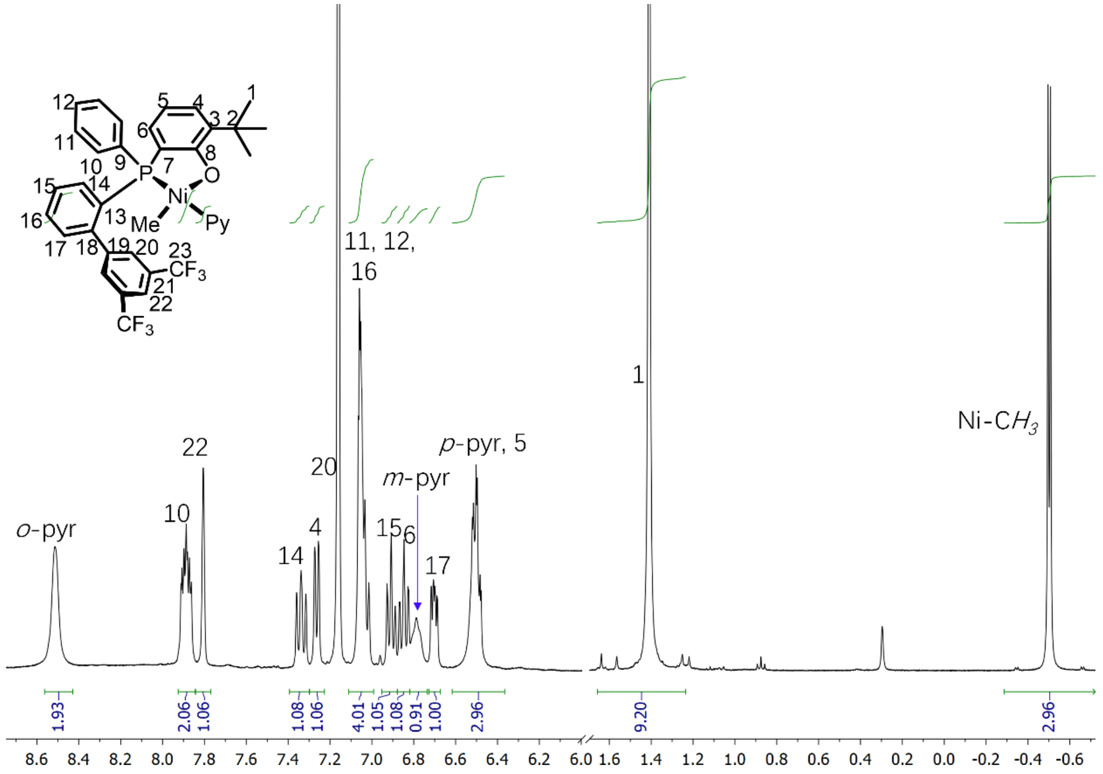

Figure S11. ^{1}H NMR spectrum of complex **1** in C_6D_6 at 300 K.

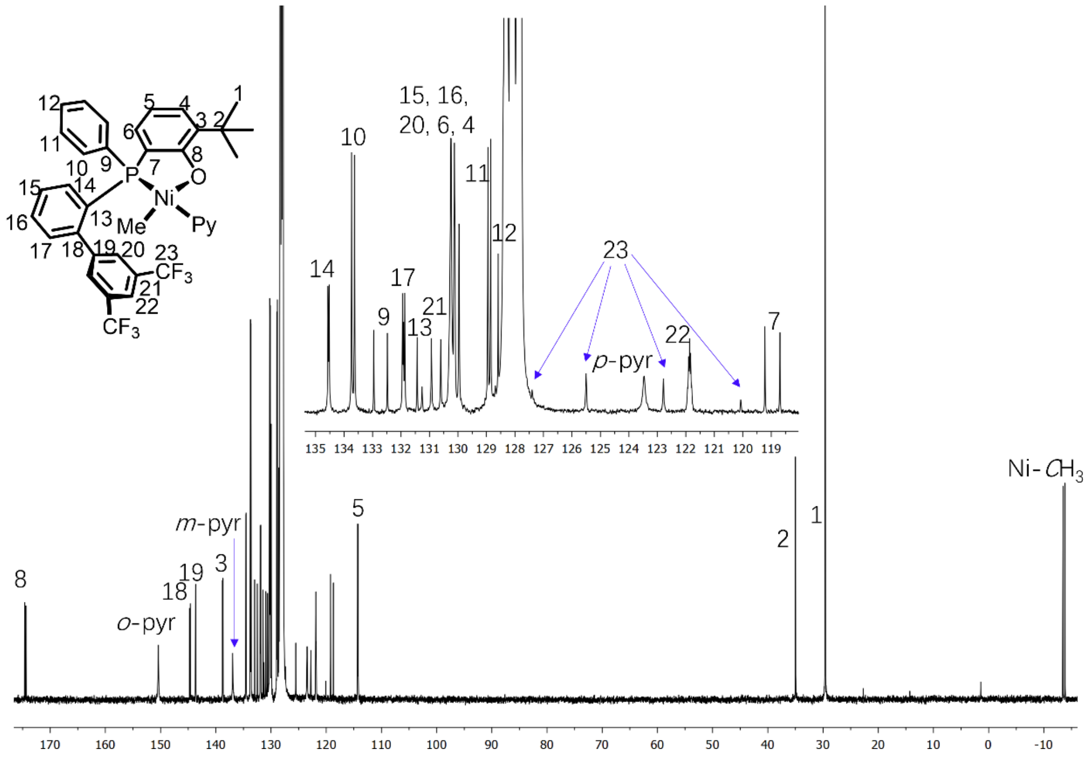

Figure S12. $^{13}C\{^{1}H\}$ NMR spectrum of complex **1** in C_6D_6 at 300 K.

2-(2-(3',5'-$(CF_3)_2C_6H_3)C_6H_4$)Ph-6-^{t}Bu-C_6H_3ONi(II)Me(tmeda) (1-tmeda) was synthesized following the above procedure. Yield: 95 %, 95 µmol, 70 mg.

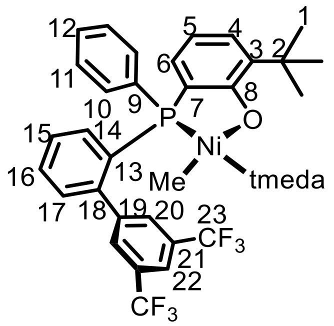

Due to multiple isomers[17] occurring for **1-tmeda** in C_6D_6, NMR characterization was performed in pyridine-d_6 giving the complex **1-pyr-d6**.

^{1}H NMR (400 MHz, Pyridine-d_5) δ 8.13-8.04 (m, 4H, 10-H and 20-H), 8.01 and 7.87 (1H, 22-H), 7.64 (d, J = 9.2 Hz, 14-H), 7.62 (d, J = 7.8 Hz, 4-H), 7.00 (ddd, J = 9.2, 7.6, 1.6 Hz, 1H, 6-H), 6.56 – 6.51 (m, 1H, 5-H), 6.49-6.40(m, 17-H), 2.39 (s, 3.58 H, tmeda-CH_2), 2.17 (s, 10.76 H, tmeda-CH_3), 1.32 (s, 9H), -0.47 (d, J = 4.9 Hz, 3H). Because tmeda can coordinated with two Ni center,the integral area of tmeda-CH_2 is less than 4H. Other peaks for 1-temda are overlapped by pyridine-d_5

^{19}F{^{1}H} NMR (376 MHz, Benzene-d_6) δ -61.71

^{31}P{^{1}H} NMR (162 MHz, Benzene-d_6) δ 28.29.

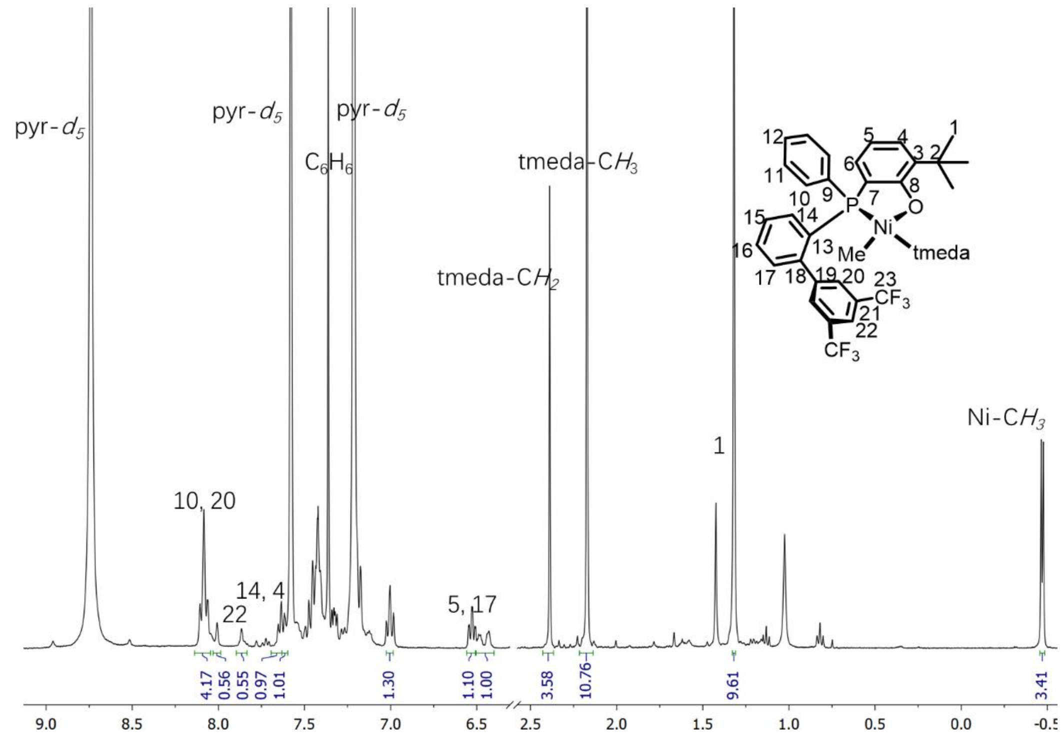

Figure S13. ^{1}H NMR spectrum of complex **1-tmeda** in pyridine-d_5 at 300 K.

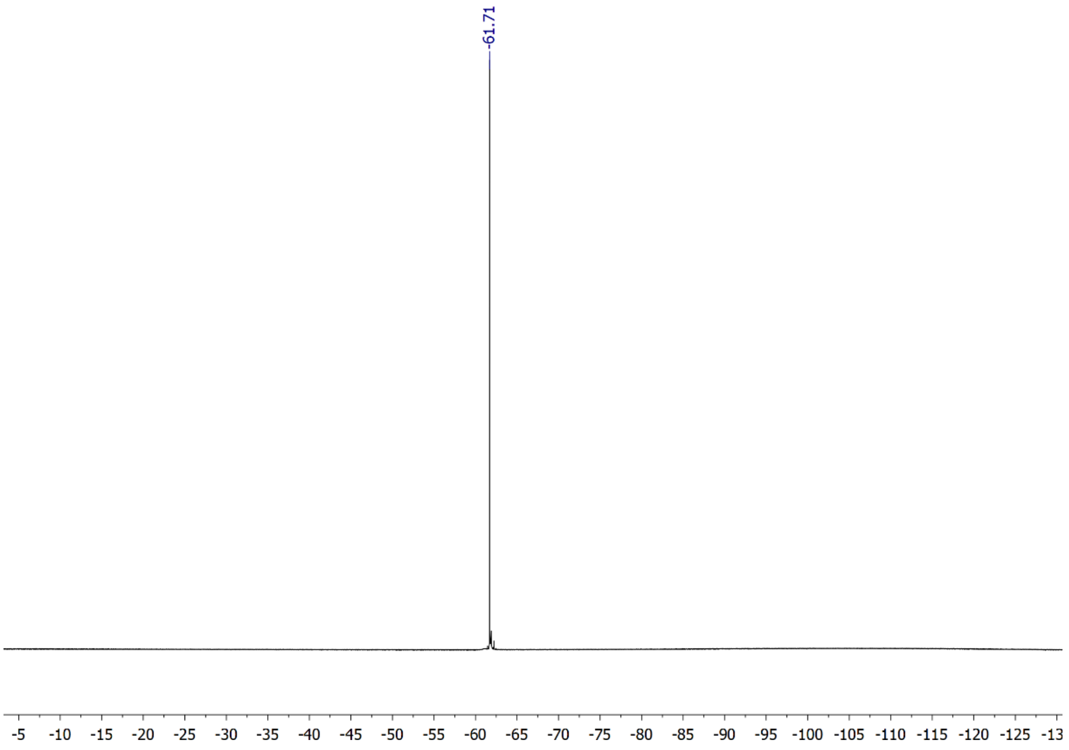

Figure S14. ^{19}F{^{1}H} NMR spectrum of complex **1-tmeda** in pyridine-d_5 at 300 K.

2-(2-(3',5'-$(CH_3)_2C_6H_3)C_6H_4$)Ph-6-^{t}Bu-C_6H_3ONi(II)Me(Pyrdine) (2) was synthesized following the general procedure. Yield: 96 %, 95 μmol, 57 mg.

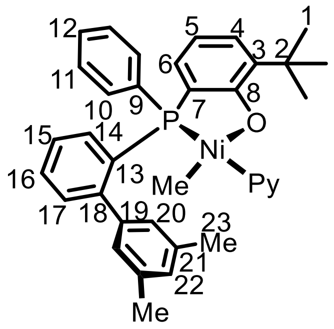

^{1}H NMR (400 MHz, Benzene-d_6) δ 8.43 (d, J = 5.5 Hz, 2H, *o*-pyr), 7.77 – 7.67 (m, 2H, 10-H), 7.59 – 7.50 (m, 1H, 16-H), 7.44 – 7.34 (m, 4H, 6-, 17- and 20-H), 7.22 (ddd, J = 9.2, 7.6, 1.6 Hz, 1H, 14-H), 7.20-7.17 (m, 1H, 4-H), 7.05 – 7.00 (m, 3H, 11- and 12-H), 6.97 (t, J = 7.6 Hz, 1H, 15-H), 6.91 (s, 1H, 22-H), 6.77 (t, J = 7.4 Hz, 1H, *p*-pyr), 6.62 (td, J = 7.4, 2.1 Hz, 1H, 5-H), 6.46 (t, J = 6.6 Hz, 2H, *m*-pyr), 2.20 (s, 6H, 23-H), 1.58 (s, 9H, 1-H), -0.75 (d, J = 4.8 Hz, 3H, Ni-CH_3).

^{13}C{^{1}H} NMR (101 MHz, Benzene-d_6) δ 174.57 (d, J = 20.4 Hz, C8), 150.65 (*o*-pyr), 148.78 (d, J = 15.3 Hz, C18), 142.29 (d, J = 4.9 Hz, C14), 138.56 (d, J = 8.7 Hz, C3), 137.21 (*p*-pyr), 136.35 (C17), 135.70 (d, J = 45.9 Hz, C7), 135.30 (d, J = 2.0 Hz, C16), 132.69 (d, J = 9.8 Hz, C10), 131.89 (d, J = 8.5 Hz, C6), 130.80 (d, J = 49.6 Hz, C9), 130.23 (d, J = 2.3 Hz, C19), 129.64 (d, J = 1.4 Hz, C4), 129.28 (C22), 129.23 (d, J = 2.1 Hz, C12), 128.91 (C21), 128.77 (C20), 128.63 (d, J = 8.4 Hz, C11), 126.60 (d, J = 6.9 Hz, C15), 123.08 (*m*-pyr), 120.41 (d, J = 51.4 Hz, C13), 113.82 (d, J = 7.7 Hz, C5), 35.22 (C2), 30.03 (C1), 21.78 (C23), -14.57 (d, J = 37.6 Hz, Ni-*C*H_3).

^{31}P{^{1}H} NMR (162 MHz, Benzene-d_6) δ 23.54.

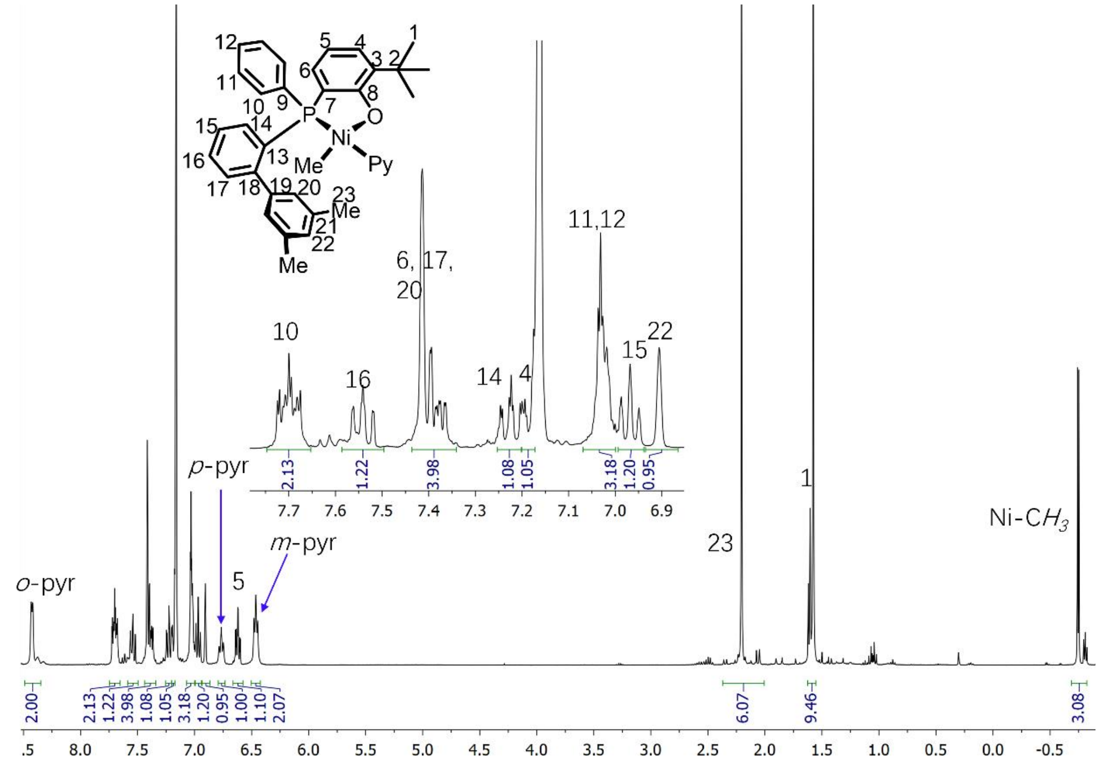

Figure S15. ^{1}H NMR spectrum of complex **2** in C_6D_6 at 300 K.

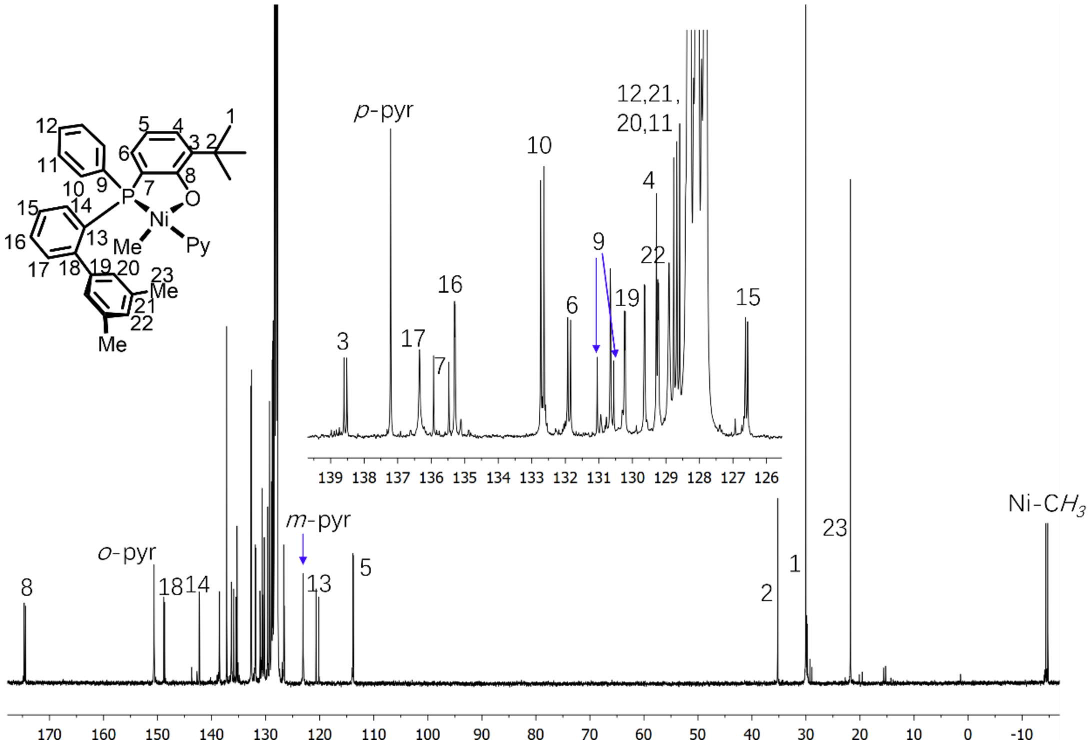

Figure S16. ^{13}C{^{1}H} NMR spectrum of complex **2** in C_6D_6 at 300 K.

2-(2-(3',5'-(OCH_3)$_2$$C_6H_3$)$C_6H_4$)Ph-6-^{t}Bu-$C_6H_3$ONi(II)Me(Pyrdine) (3) was synthesized following the general procedure. Yield: 98 %, 98 μmol, 61 mg.

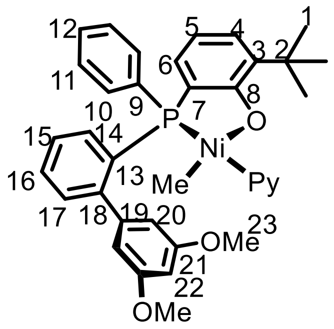

^{1}H NMR (400 MHz, Benzene-d_6) δ 8.50 (d, J = 5.0 Hz, 2H, *o*-pyr), 7.72 (t, J = 8.1 Hz, 2H, 10-H), 7.51 (t, J = 9.0 Hz, 1H, 16-H), 7.41 – 7.32 (m, 2H, 4-H, 17-H), 7.14-7.11 (m, 2H, 12-H, 6-H), 7.08 – 7.01 (m, 3H, 11- and 14-H), 6.96 (t, J = 7.6 Hz, 1H, 15-H), 6.92 – 6.81 (br, 2H, 20-H), 6.76 (t, J = 8.0 Hz, 1H, p-pyr), 6.72 (s, 1H, 22-H), 6.57 (td, J = 7.3, 1.8 Hz, 1H, 5-H), 6.48 (t, J = 6.6 Hz, 2H, m-pyr), 3.30 (s, 6H, 23-H), 1.54 (s, 9H, 1-H), -0.59 (d, J = 4.9 Hz, 3H, Ni-CH_3).

^{13}C{^{1}H} NMR (101 MHz, Benzene-d_6) δ 174.89 (d, J = 20.6 Hz, C8), 160.68 (C21), 150.81 (*o*-pyr), 148.96 (d, J = 15.7 Hz, C6), 144.09 (d, J = 5.4 Hz, C18), 138.80 (d, J = 8.6 Hz, C3), 136.26 (p-pyr), 134.88 (d, J = 45.6 Hz, C9), 134.81 (d, J = 2.3 Hz, C16), 132.77 (d, J = 9.7 Hz, C10), 131.62 (d, J = 8.5 Hz, C14), 130.99 (d, J = 49.4 Hz, C13), 130.63 (C12), 130.16 (d, J = 2.2 Hz, C19), 129.58 (d, J = 1.3 Hz, C17), 129.34 (d, J = 2.0 Hz, C4), 128.78 (d, J = 9.2 Hz, C11), 126.85 (d, J = 7.0 Hz, C15), 123.11 (m-pyr), 120.36 (d, J = 51.6 Hz, C7), 113.57 (d, J = 7.7 Hz, C5), 108.79 (C20), 101.41 (C22), 54.98 (C23), 35.18 (C2), 29.82 (C1), -14.14 (d, J = 37.1 Hz, Ni-CH$_3$).

^{31}P{^{1}H} NMR (162 MHz, Benzene-d_6) δ 23.41.

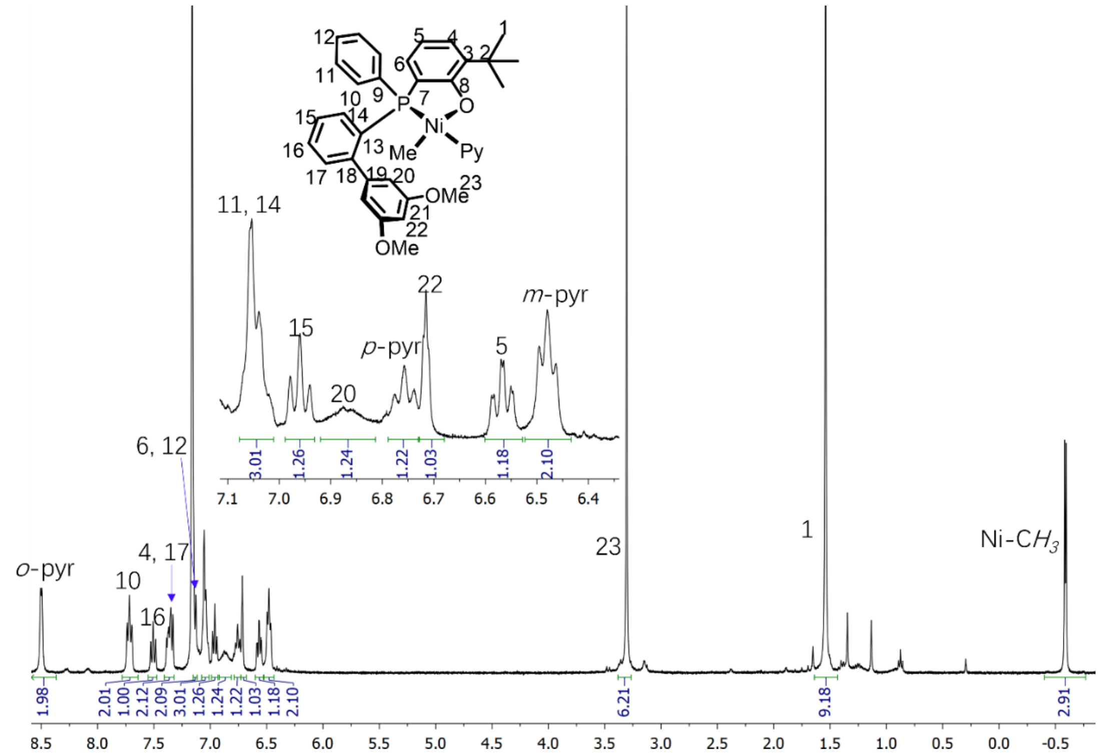

Figure S17. ^{1}H NMR spectrum of complex **3** in C_6D_6 at 300 K.

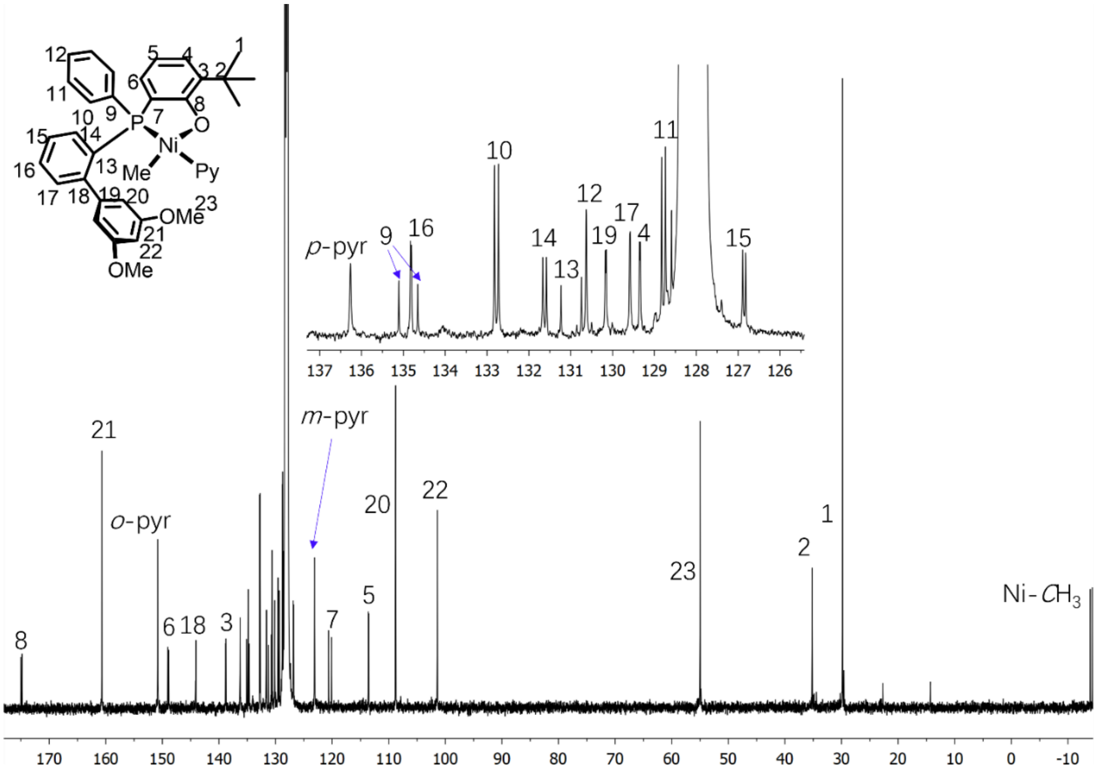

Figure S18. $^{13}C\{^{1}H\}$ NMR spectrum of complex **3** in C_6D_6 at 300 K.

2-(2-(2',4',6'-$(OCH_3)_3C_6H_2)C_6H_4$)Ph-6-^{t}Bu-C_6H_3ONi(II)Me(Pyrdine) (4) was synthesized following the general procedure. Yield: 97 %, 97 μmol, 63 mg.

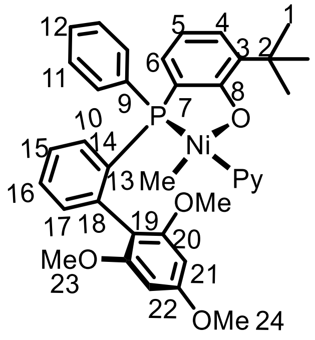

^{1}H NMR (400 MHz, Benzene-d_6) δ 8.44 (dd, J = 4.8, 1.8 Hz, 2H, *o*-pyr), 7.68-7.60 (m, 3H, 10- and 16-H), 7.54 (dd, J = 6.8, 4.6 Hz, 1H, 6-H), 7.30 (d, J = 7.1 Hz, 1H, 4-H), 7.28 – 7.20 (m, 2H, 14- and 17-H), 7.10 – 6.92 (m, 4H, 11-, 12-H, 15-H), 6.83 (tt, J = 7.5, 1.7 Hz, 1H, *p*-pyr), 6.60 (td, J = 7.4, 2.1 Hz, 1H, 5-H), 6.56 (t, J = 6.7 Hz, 2H, *m*-pyr), 6.29 (d, J = 2.2 Hz, 1H, 21-H), 6.16 (d, J = 2.2 Hz, 1H, 21-H), 3.43 (s, 3H, 23-H), 3.34 (s, 3H, 23-H), 2.96 (s, 3H, 24-H), 1.55 (s, 9H, 1-H), -0.65 (d, J = 4.9 Hz, 3H, Ni-CH_3).

^{13}C{^{1}H} NMR (101 MHz, Benzene-d_6) δ 175.08 (d, J = 20.9 Hz, C8), 162.04 (C22), 160.44 (C20), 158.98 (C20), 150.83 (*o*-pyr), 141.99 (d, J = 17.0 Hz, C18), 137.52 (d, J = 8.7 Hz, C3), 136.92 (d, J = 47.4 Hz, C13), 136.11 (*p*-pyr), 134.93 (d, J = 1.7 Hz, C16), 133.87 (d, J = 9.1 Hz, C6), 133.09 (d, J = 51.3 Hz, C9), 132.63 (d, J = 9.3 Hz, C10), 130.71 (C15), 130.04 (d, J = 2.2 Hz, C12), 128.87 (d, J = 1.3 Hz, C4), 128.77 (d, J = 2.0 Hz, C17), 128.46 (d, J = 9.2 Hz, C11), 126.54 (d, J = 6.8 Hz, C14), 122.93 (d, J = 1.5 Hz, *m*-pyr), 120.19 (d, J = 51.0 Hz, C7), 113.32 (d, J = 7.9 Hz, C5), 113.05 (d, J = 5.6 Hz, C19), 90.40 (C21), 89.70 (C21), 54.74 (d, J = 4.3 Hz, C23), 53.96 (C24), 35.17 (C2), 29.83 (C1), -14.40 (d, J = 36.1 Hz, Ni-CH_3).

^{31}P{^{1}H} NMR (162 MHz, Benzene-d_6) δ 20.75.

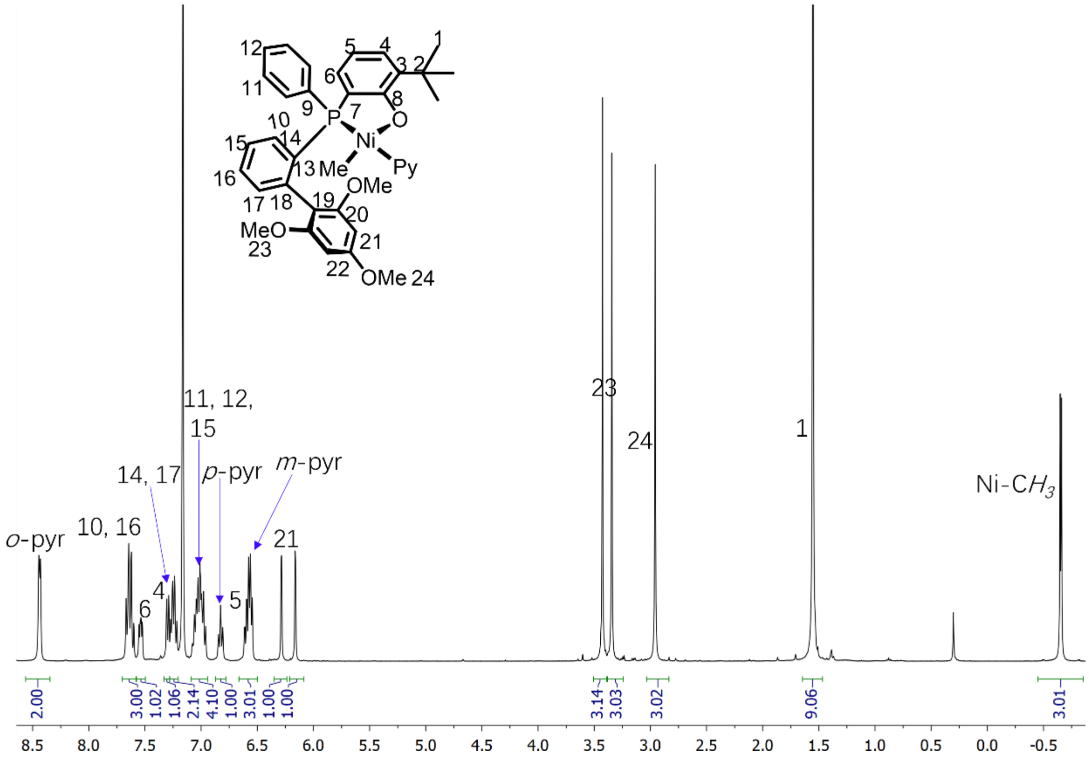

Figure S19. ^{1}H NMR spectrum of complex **4** in C_6D_6 at 300 K.

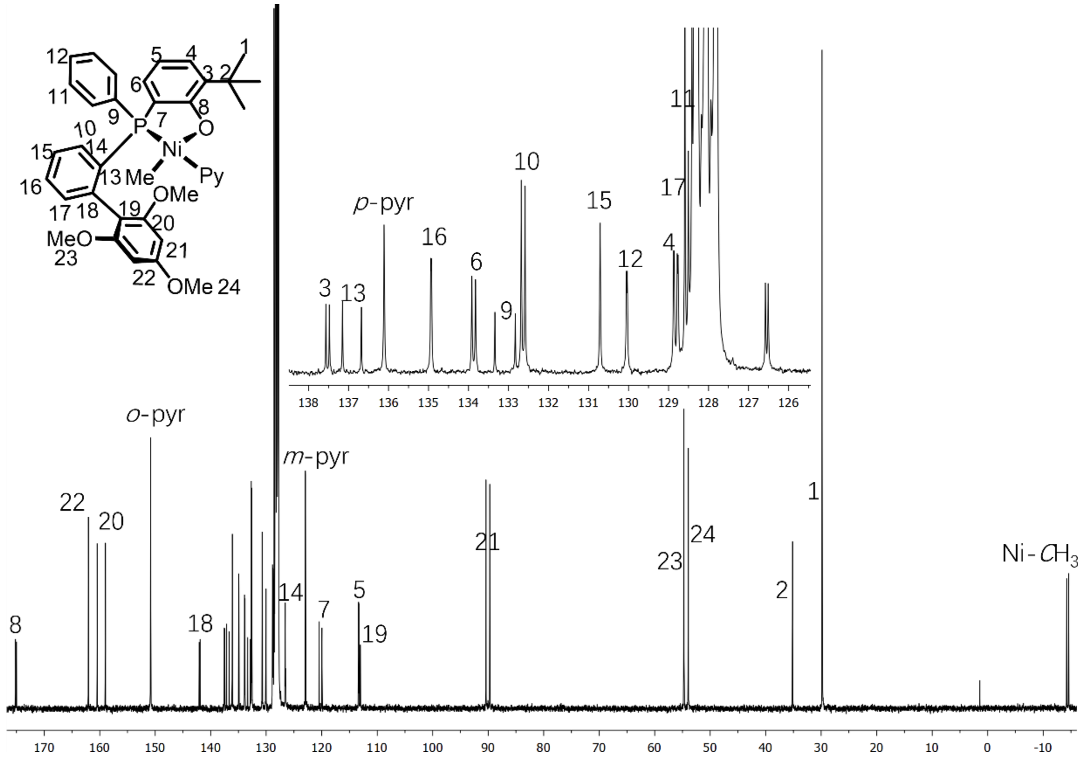

Figure S20. $^{13}C\{^{1}H\}$ NMR spectrum of complex **4** in C_6D_6 at 300 K.

2-(2-(2',4',6'-(^{i}Pr)$_3$C$_6$H$_2$)C$_6$H$_4$)Ph-6-^{t}Bu-C$_6$H$_3$ONi(II)Me(Pyrdine) (5) was synthesized following the general procedure. Yield: 96 %, 96 μmol, 66 mg.

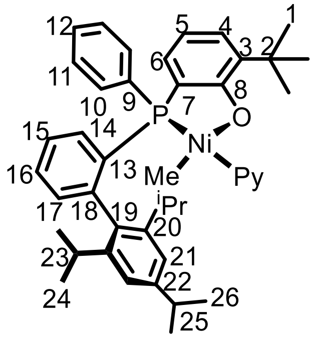

^{1}H NMR (400 MHz, Benzene-d_6) δ 8.41 (br, 2H, *o*-pyr), 7.87 (t, *J* = 8.7 Hz, 1H, 14-H), 7.63 (td, *J* = 8.1, 7.5, 3.8 Hz, 2H, 10-H), 7.45 – 7.28 (m, 3H, 6-, 12- and 16-H), 7.20 (s, 2H, 21-H), 7.12 (t, *J* = 7.5 Hz, 1H, 17-H), 7.08 – 6.94 (m, 5H, 4-, 11- and 15-H), 6.76 (br, 1H, *p*-pyr), 6.55 (t, *J* = 7.6, 1H, 5-H), 6.48 (br, 2H, *m*-pyr), 2.90 (dp, *J* = 13.8, 6.8 Hz, 2H, 23-H), 2.74 (p, *J* = 6.7 Hz, 1H, 25-H), 1.53 (s, 9H, 1-H), 1.40 (d, *J* = 6.8 Hz, 3H, 24-H), 1.27 (d, *J* = 6.0 Hz, 3H, 24-H), 1.25 (d, *J* = 6.6 Hz, 3H, 26-H), 1.23 (d, *J* = 7.4 Hz, 3H, 24-H), 1.21 (d, *J* = 6.8 Hz, 3H, 24-H), 0.96(d, *J* = 6.6 Hz, 3H, 26-H), -0.83 (d, *J* = 5.0 Hz, 3H, Ni-CH_3).

^{13}C{^{1}H} NMR (101 MHz, Benzene-d_6) δ 175.81 (d, *J* = 21.0 Hz, C8), 150.57 (*o*-pyr), 148.55 (C20), 148.45 (C20), 147.41 (C22), 145.91 (d, *J* = 15.3 Hz, C18), 138.39 (d, *J* = 8.4 Hz, C3), 137.73 (d, *J* = 3.3 Hz, C19), 137.04 (d, *J* = 47.6 Hz, C13), 136.51 (d, *J* = 3.2 Hz, C14), 136.27 (*p*-pyr), 134.18 (d, *J* = 8.9 Hz, C6), 132.46 (d, *J* = 9.7 Hz, C10), 131.32 (d, *J* = 45.9 Hz, C9), 130.24 (C4), 129.61 (C12), 129.27 (C16), 129.94 (C17), 128.54 (d, *J* = 9.5 Hz, C11), 126.61 (d, *J* = 7.0 Hz, C15), 123.18 (*m*-pyr), 121.08 (C21), 120.45 (C21), 119.47 (d, *J* = 49.1 Hz, C7), 113.88 (d, *J* = 7.6 Hz, C5), 35.20 (C23), 34.85 (C2), 31.77 (C23), 31.46 (C25), 29.84 (C1), 27.44 (C24), 26.60 (C26), 24.55 (C24), 24.27 (C24), 23.04 (C26), 22.06 (C24), -11.35 (d, *J* = 34.9 Hz, Ni-*C*H_3).

^{31}P{^{1}H} NMR (162 MHz, Benzene-d_6) δ 23.63.

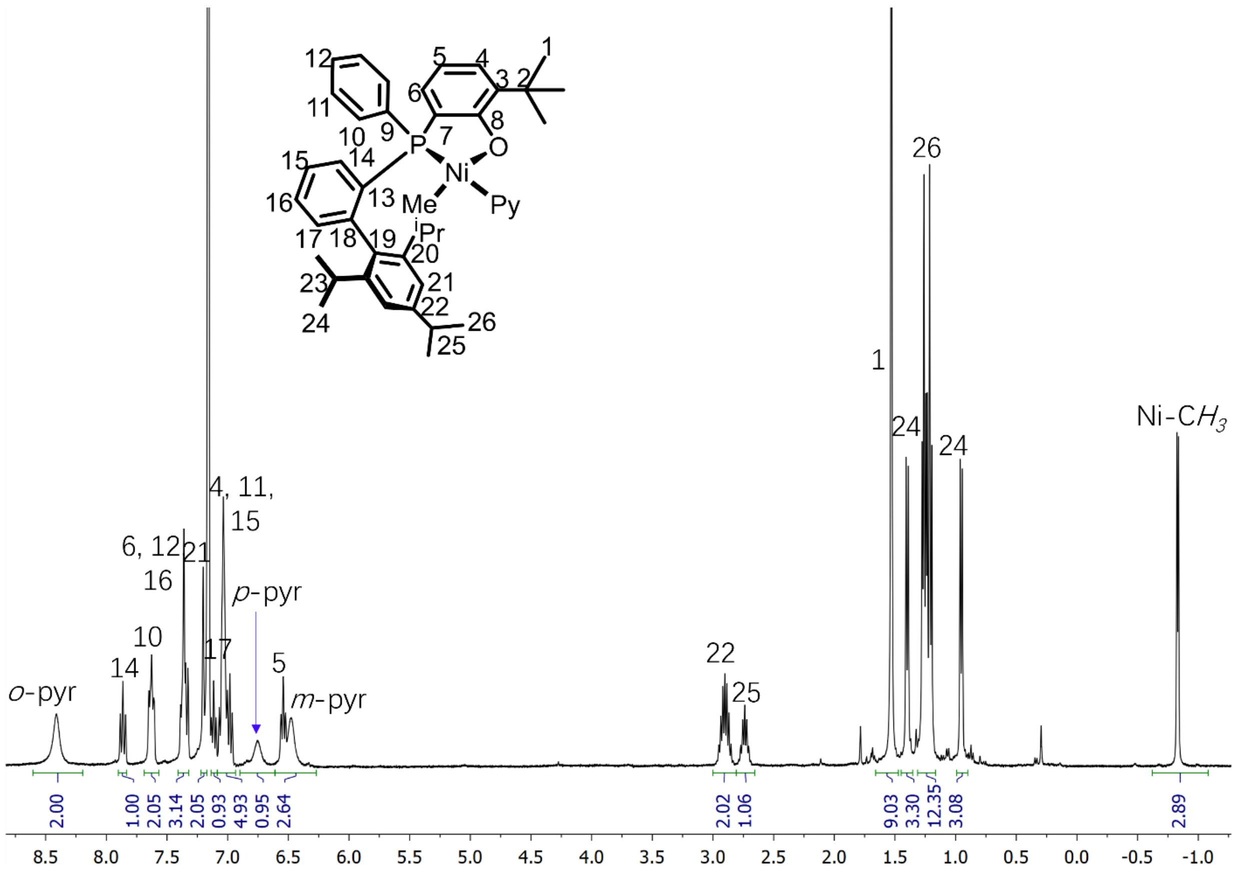

Figure S21. ^{1}H NMR spectrum of complex **5** in C_6D_6 at 300 K.

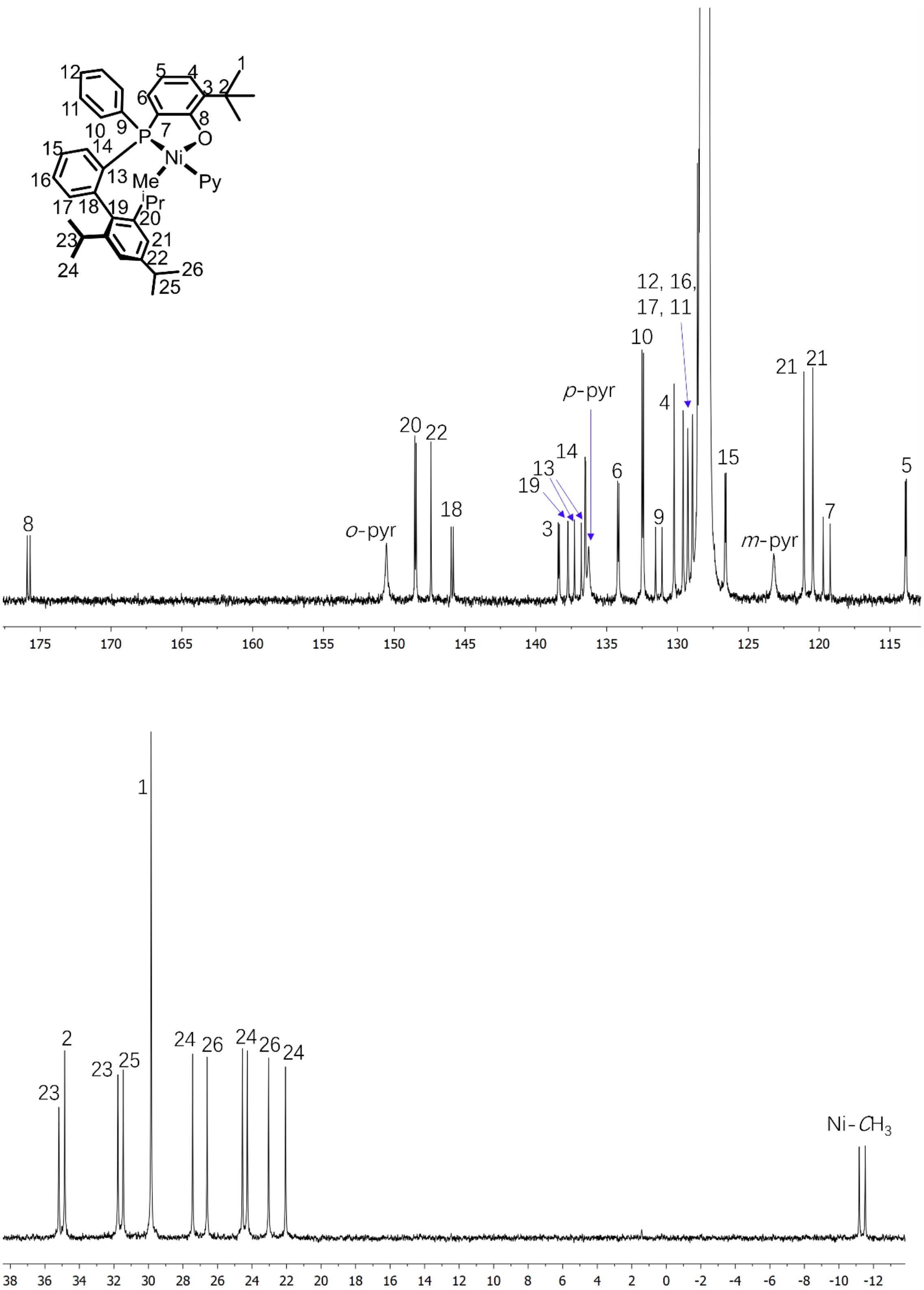

Figure S22. $^{13}C\{^1H\}$ NMR spectrum of complex **5** in C_6D_6 at 300 K, details of aromatic region (top) and of aliphatic region (bottom).

2.2 Synthesis and NMR spectra of phosphinephenol **6a** and complex **6**.

The phosphinephenol **6** were prepared by modification of a reported procedure.[18] At room temperature n-BuLi (2.6 mL, 2.5 M in hexane, 6.6 mmol, 1.1 equiv) was added dropwise to a solution of 2-phenoxytetrahydro-2H-pyran (1.1 g, 6 mmol, 1.0 equiv.) in diethyl ether (20 mL) at 0 ºC. The reaction mixture was stirred for 2 hours to give a white suspension. The obtained suspension was added dropwise to a solution of $PhPCl_2$ (1.17 g, 6.6 mmol, 1.1 equiv.) in diethyl ether (20 mL) at -78 ºC. The mixture was warmed to room temperature slowly over 8 hours to obtain a white suspension **A**. In another Schlenk flask, n-BuLi (2.6 mL, 2.5 M in hexane, 6.6 mmol, 1.1 equiv) was added dropwise to a solution of 2'-bromo-2,4,6-trimethoxy-1,1'-biphenyl (6 mmol, 1.0 equiv.) in THF (40 mL) at -78 ºC. The reaction mixture was stirred for two hours, to yield a suspension **B**. Then, the suspension **A** was added to suspension **B** at -78 ºC, and the reaction mixture was warmed to room temperature slowly over 8 hours. The mixture was concentrated in vacuo, the residue was dissolved in THF (30 mL) and cooled to 0 ºC. n-BuLi (2.6 mL, 2.5 M in hexane, 6.6 mmol, 1,1 equiv.) was added dropwise to the solution and stirred for 2 hours. Then, the mixture was cooled to -78 ºC and C_6F_6 (5.6 g, 30 mmol, 5.0 equiv.) was added to the above solution. The mixture was warmed to room temperature slowly and stirred for 16 hours. The mixture was concentrated in vacuo, and the residue was dissolved in 20 mL of degassed ethyl acetate, and 1 mL of conc. HCl was injected. The reaction mixture was stirred at room temperature overnight, added slowly into a solution of 5 g $NaHCO_3$ in 30 mL H_2O, and stirred for 30 minutes. The organic phase was separated, and the aqueous phase was extracted with ethyl acetate (2 × 40 mL). The combined organic phases were concentrated in vacuo, and the residue was subjected to column chromatography on silica using petrol ether/ethylene acetate mixture as eluent to give the pure phosphinephenol **6a** (1.4 g, 2.3mmol, 39%).

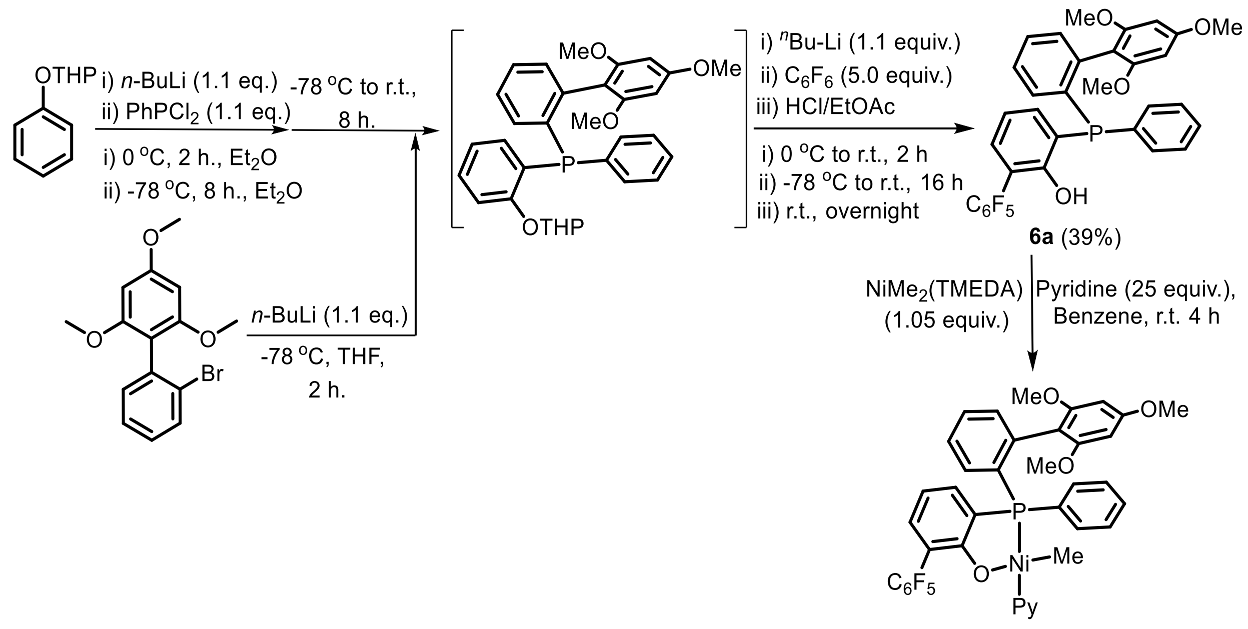

2-(2-(3’,5’-$(CF_3)_2C_6H_3$)C_6H_4)Ph-6-C_6F_5-C_6H_3OH (6a)

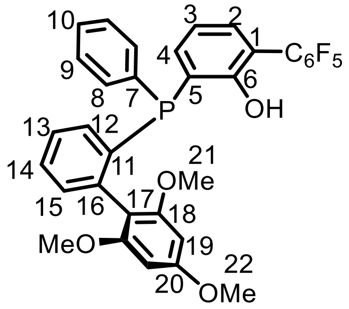

^{1}H NMR (400 MHz, Benzene-d_6) δ 7.46 (ddd, J = 7.8, 4.3, 1.4 Hz, 1H, 12-H), 7.43 – 7.36 (m, 1H, 4-H), 7.34 – 7.26 (m, 2H, 8-H), 7.24 – 7.18 (m, 2H, 3-H and 2-H), 7.08 – 6.96 (m, 6H, 9-, 10-, 13-, 15-H and O*H*), 6.73 (t, J = 7.6 Hz, 1H, 14-H), 6.09 (dd, J = 15.6, 2.2 Hz, 2H, 19-H), 3.35 (s, 3H, 22-H), 3.18 (s, 3H, 20-H), 3.06 (s, 3H, 20-H).

^{13}C{^{1}H} NMR (101 MHz, Benzene-d_6) δ 161.67 (C20), 158.70 (C18), 158.35 (C20), 156.92 (d, *J* = 20.6 Hz, C6), 142.28 (d, *J* = 34.0 Hz, C16), 135.80 (d, *J* = 2.4 Hz, C2), 135.31 (d, *J* = 1.0 Hz, C14), 134.85 (d, *J* = 8.1 Hz, C13), 133.57 (d, *J* = 19.4 Hz, C8), 133.51 (d, *J* = 1.6 Hz, C10), 132.62 (C5), 132.12 (d, *J* = 6.4 Hz, C11), 129.74 (C7), 128.33 (C3), 128.33 (d, *J* = 6.7 Hz, C9), 124.35 (d, *J* = 9.3 Hz, C15), 120.38(C4), 113.29(C11), 112.00 (d, *J* = 8.3 Hz, C17), 91.10 (C19), 90.56

(C19), 54.97 (C22), 54.69 (C21), 54.47 (C21). The signal for C12 is overlapped in C_6D_6, the peaks for -C_6F_5 are extremely broad due to multiple $^{x}J_{CF}$ couplings.

$^{19}F\{^{1}H\}$ NMR (376 MHz, Benzene-d_6) δ-140.32 (ddd, J = 86.1, 23.9, 8.0 Hz), -156.48 (t, J = 21.4 Hz), -163.55 (dtd, J = 90.8, 22.6, 7.8 Hz).

$^{31}P\{^{1}H\}$ NMR (162 MHz, Benzene-d_6) δ -36.31.

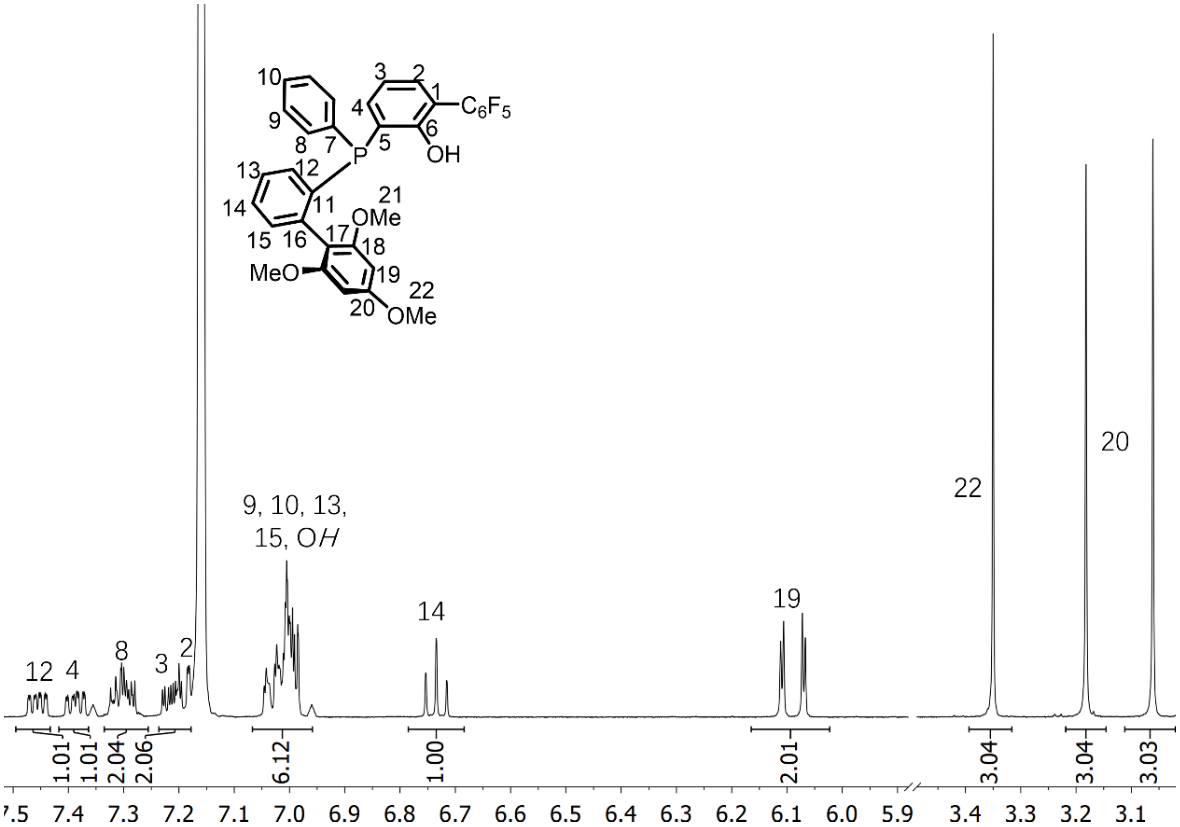

Figure S23. ^{1}H NMR spectrum of phosphinephenol **6a** in C_6D_6 at 300 K.

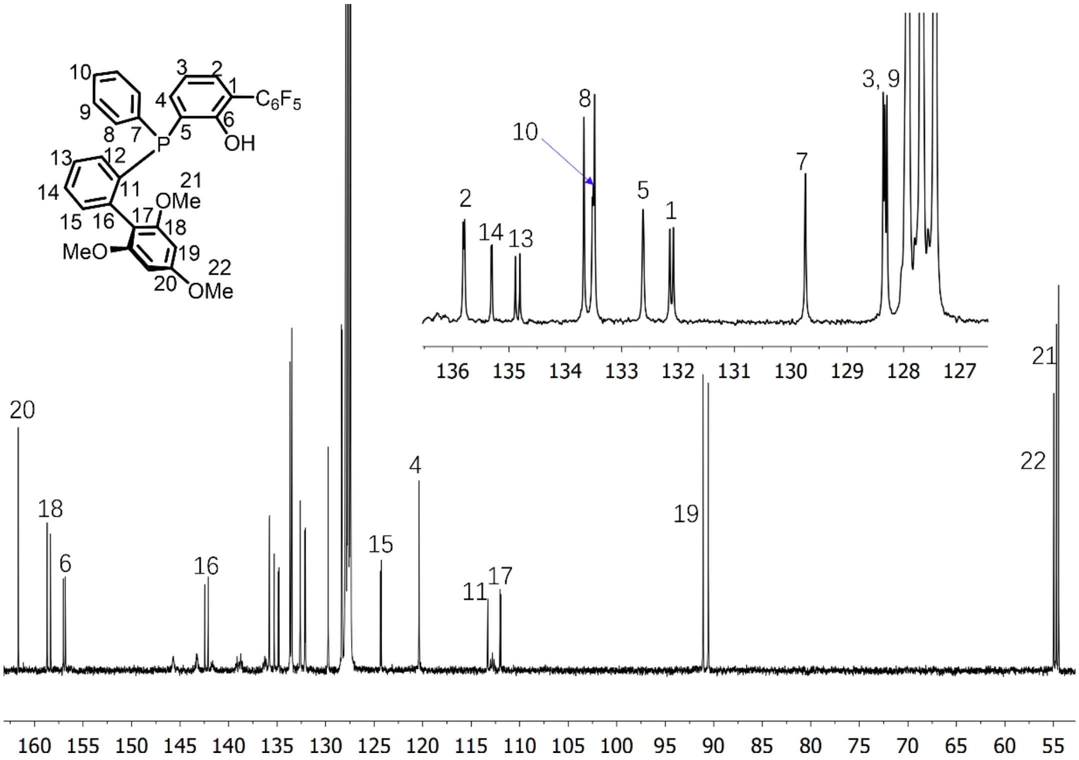

Figure S24. $^{13}C\{^1H\}$ NMR spectrum of phosphinephenol **6a** in C_6D_6 at 300 K.

2-(2-(3’,5’-$(CF_3)_2C_6H_3)C_6H_4$)Ph-6-C_6F_5-C_6H_3ONi(II)Me(Pyrdine) (6) was synthesized following the general procedure. Yield: 96 %, 96 μmol, 76 mg.

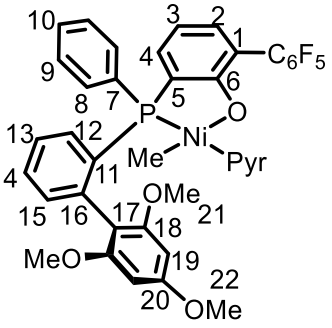

^{1}H NMR (400 MHz, Benzene-d_6) δ8.36 (d, J = 5.2 Hz, 2H, *o*-pyr), 7.60 (td, J = 8.5, 2.5 Hz, 3H, 8- and 14-H), 7.52 (dd, J = 7.1, 5.1 Hz, 1H, 4-H), 7.38 (ddd, J = 9.2, 7.8, 1.5 Hz, 1H, 12-H), 7.24 (t, J = 7.5 Hz, 1H, 15-H), 7.19 (d, J = 7.0 Hz, 1H, 2-H), 7.10 – 6.91 (m, 4H, 9-, 10- and 13-H), 6.77 (t, J = 7.8 Hz, 1H, *p*-pyr), 6.65 – 6.46 (m, 3H, 5-H and *m*-pyr), 6.26 (s, 1H, 19-H), 6.20 (s, 1H, 19-H), 3.32 (s, 3H, 21-H), 3.30 (s, 3H, 21-H), 3.11 (s, 3H, 22-H), -0.74 (d, J = 5.2 Hz, 3H, Ni-CH_3).

$^{13}C\{^1H\}$ NMR (101 MHz, Benzene-d_6) δ 173.14 (d, J = 21.7 Hz, C6), 161.82 (C20), 159.88 (C18), 158.65 (C18), 150.13 (*o*-pyr), 141.74 (d, J = 17.5 Hz, C16), 136.03(*p*-pyr), 135.93 (d, J = 48.9 Hz, C11), 134.41 (C14), 133.92 (d, J = 12.3 Hz, C4), 133.72 (d, J = 9.3 Hz, C1), 132.05 (d, J = 9.6 Hz, C8), 131.67 (d, J = 51.7 Hz, C7), 130.01 (d, J = 2.3 Hz, C13), 128.68 (d, J = 2.3 Hz, C10), 128.22 (C15), 128.21 (d, J = 9.3 Hz, C9), 126.31 (d, J = 6.8 Hz, C12), 122.76 (*m*-pyr), 122.43 (d, J = 48.5 Hz, C5), 112.88 (d, J = 7.2 Hz, C3), 112.38 (d, J = 5.6 Hz, C17), 90.65 (C19), 89.07 (C19), 54.46 (C21), 54.31 (C21), 53.59 (C22), 53.55 (C22), -14.67 (d, J = 36.6 Hz, Ni-CH_3). The peaks for -C_6F_5 are extremely broad due to multiple $^xJ_{CF}$ couplings.

$^{19}F\{^1H\}$ NMR (376 MHz, Benzene-d_6) δ -139.05 (ddd, J = 839.7, 24.4, 7.6 Hz), -159.92 (t, J = 21.5 Hz), -165.63 (dtd, J = 79.3, 22.2, 7.5 Hz).

$^{31}P\{^1H\}$ NMR (162 MHz, Benzene-d_6) δ 19.52.

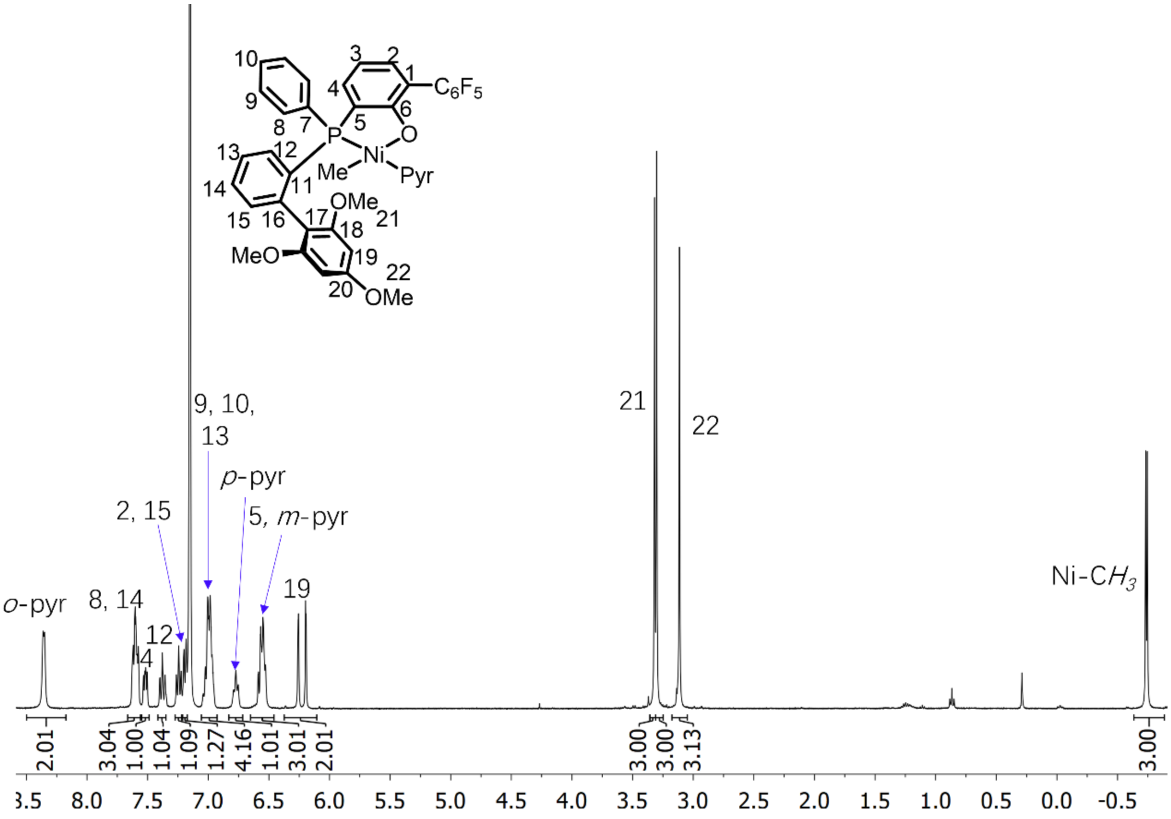

Figure S25. ^1H NMR spectrum of complex **6** in C_6D_6 at 300 K.

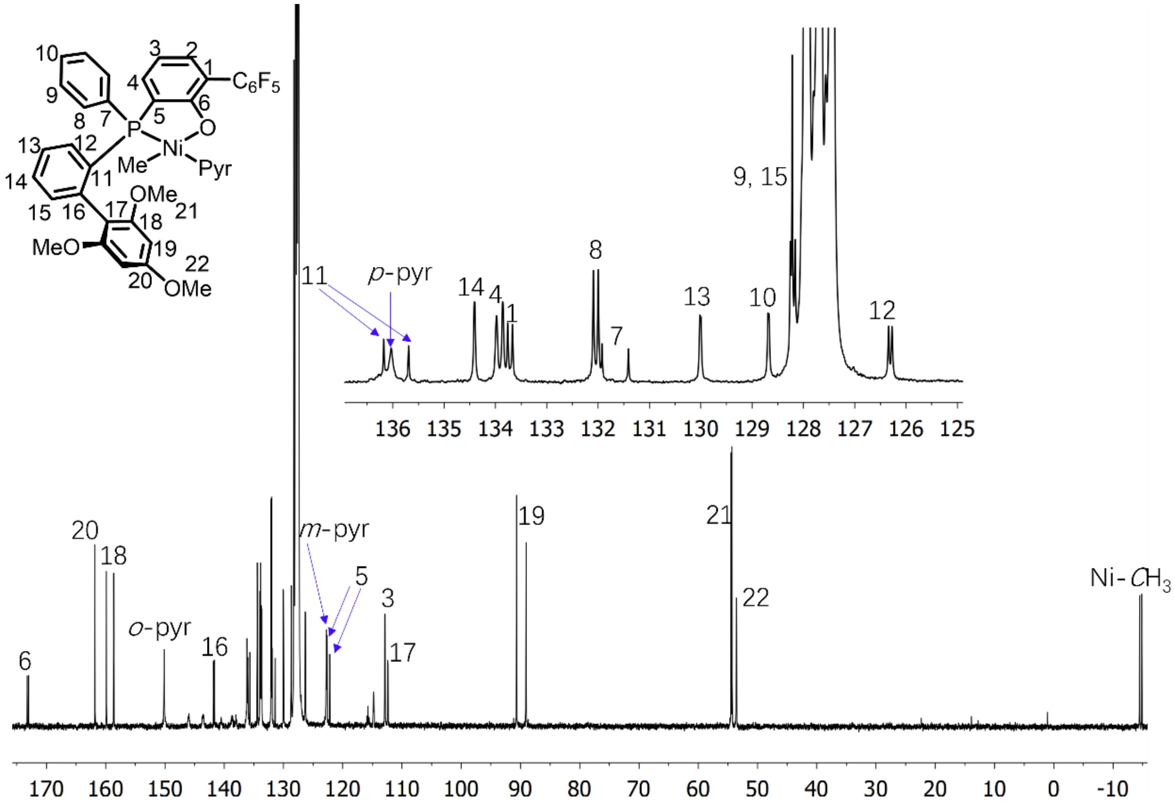

Figure S26. $^{13}C\{^1H\}$ NMR spectrum of complex **6** in C_6D_6 at 300 K.

Table S1. Selected NMR chemical shifts of complexes **1-6**.

Entry	Complex	δ Ni-CH_3 (ppm)	δ Ni-CH$_3$ (ppm)	δ Ni-P (ppm)
1	**1**	-0.50	-13.64	27.38
2	**2**	-0.75	-14.57	23.54
3	**3**	-0.59	-14.14	23.41
4	**4**	-0.65	-14.40	20.75
5	**5**	-0.83	-11.35	23.63
6	**6**	-0.73	-14.30	19.52

2.3 Crystallographic Details

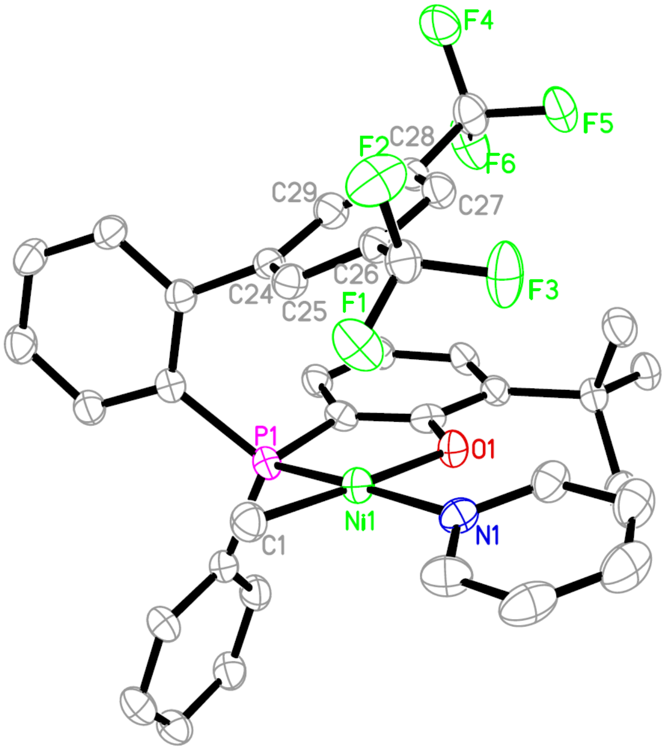

Figure S27. Crystal structure of complex **1**, hydrogen atoms are excluded for clarity.

Table S2. Crystal data and structure refinement of complex **1**.

Identification code	Complex **1**
CCDC number	2264071
Empirical formula	$C_{36}H_{32}F_6NNiOP$
Formula weight	698.30
Temperature/K	100.0
Crystal system	triclinic
Space group	P-1
a/Å	8.9333(6)
b/Å	12.8783(12)
c/Å	14.2065(9)
α/°	88.868(6)
β/°	84.276(5)
γ/°	78.132(6)
Volume/Å^3	1591.5(2)
Z	2
ρ_{calc}g/cm^3	1.457

μ/mm^{-1}	0.726
F(000)	720.0
Crystal size/mm^3	0.08 × 0.06 × 0.02
Radiation	Mo Kα (λ = 0.71073)
2Θ range for data collection/°	4.328 to 53.588
Index ranges	-11 ≤ h ≤ 11, -16 ≤ k ≤ 16, -18 ≤ l ≤ 16
Reflections collected	12448
Independent reflections	6696 [R_{int} = 0.0501, R_{sigma} = 0.0674]
Data/restraints/parameters	6696/0/419
Goodness-of-fit on F^2	1.041
Final R indexes [I>=2σ (I)]	R_1 = 0.0635, wR_2 = 0.1138
Final R indexes [all data]	R_1 = 0.1229, wR_2 = 0.1437
Largest diff. peak/hole / e Å^{-3}	0.61/-0.68

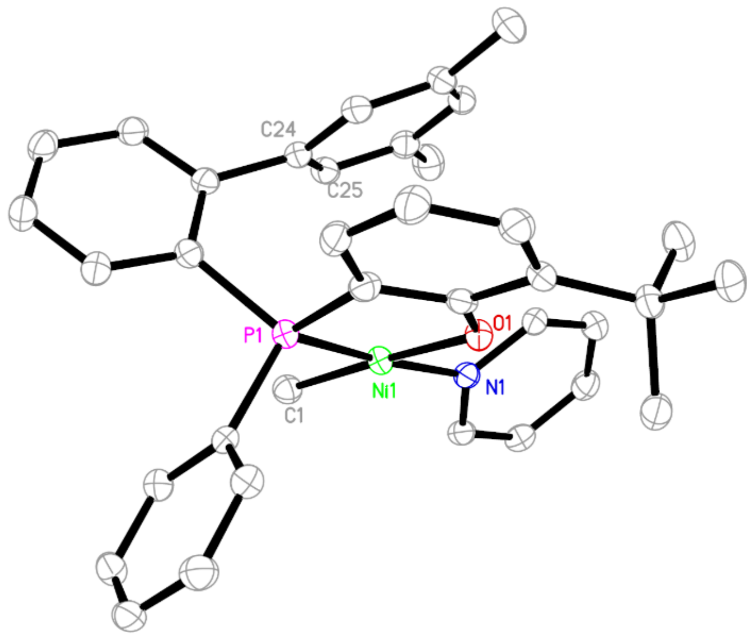

Figure S28. Crystal structure of complex **2**, hydrogen atoms are excluded for clarity.

Table S3. Crystal data and structure refinement of complex **2**.

Identification code	Complex **2**
CCDC number	2264073
Empirical formula	$C_{36}H_{38}NNiOP$
Formula weight	590.35
Temperature/K	100
Crystal system	triclinic
Space group	P-1

a/Å	10.9003(6)
b/Å	11.4299(7)
c/Å	13.8030(11)
α/°	99.326(6)
β/°	91.969(6)
γ/°	115.042(4)
Volume/$Å^3$	1527.23(18)
Z	2
ρ_{calc}g/cm^3	1.284
μ/mm^{-1}	0.716
F(000)	624.0
Crystal size/mm^3	0.3 × 0.163 × 0.04
Radiation	Mo Kα (λ = 0.71073)
2Θ range for data collection/°	4.01 to 53.652
Index ranges	-12 ≤ h ≤ 13, -14 ≤ k ≤ 14, -17 ≤ l ≤ 17
Reflections collected	16298
Independent reflections	6483 [R_{int} = 0.0266, R_{sigma} = 0.0245]
Data/restraints/parameters	6483/0/367
Goodness-of-fit on F^2	1.051
Final R indexes [I>=2σ (I)]	R_1 = 0.0418, wR_2 = 0.0895
Final R indexes [all data]	R_1 = 0.0574, wR_2 = 0.1035
Largest diff. peak/hole / e $Å^{-3}$	0.54/-0.66

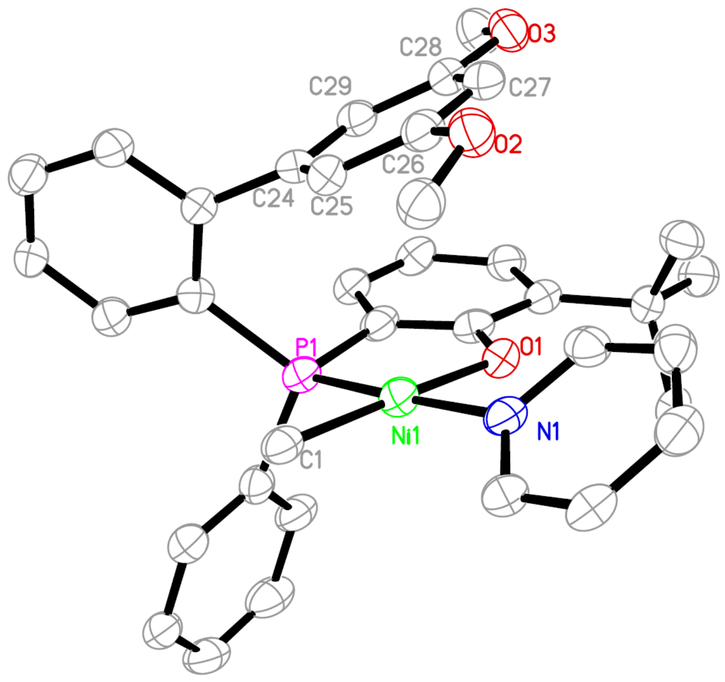

Figure S29. Crystal structure of complex **3**, hydrogen atoms are excluded for clarity.

Table S4. Crystal data and structure refinement of complex **3**.

Identification code	Complex **3**
CCDC number	2264072
Empirical formula	$C_{36}H_{38}NO_3PNi$
Formula weight	622.35
Temperature/K	100.0
Crystal system	triclinic
Space group	P-1
a/Å	10.7645(5)
b/Å	11.4292(5)
c/Å	14.2690(7)
α/°	82.674(4)
β/°	88.789(4)
γ/°	62.402(3)
Volume/$Å^3$	1541.63(13)
Z	2
$\rho_{calc}g/cm^3$	1.341
μ/mm^{-1}	0.718
F(000)	656.0
Crystal size/mm^3	0.15 × 0.09 × 0.02
Radiation	Mo Kα (λ = 0.71073)
2Θ range for data collection/ °	4.654 to 53.686
Index ranges	-13 ≤ h ≤ 13, -14 ≤ k ≤ 14, -18 ≤ l ≤ 16
Reflections collected	20378
Independent reflections	6497 [R_{int} = 0.1432, R_{sigma} = 0.1263]
Data/restraints/parameters	6497/0/386
Goodness-of-fit on F^2	0.966
Final R indexes [I>=2σ (I)]	R_1 = 0.0703, wR_2 = 0.1634
Final R indexes [all data]	R_1 = 0.1540, wR_2 = 0.2088
Largest diff. peak/hole / e $Å^{-3}$	0.55/-0.80

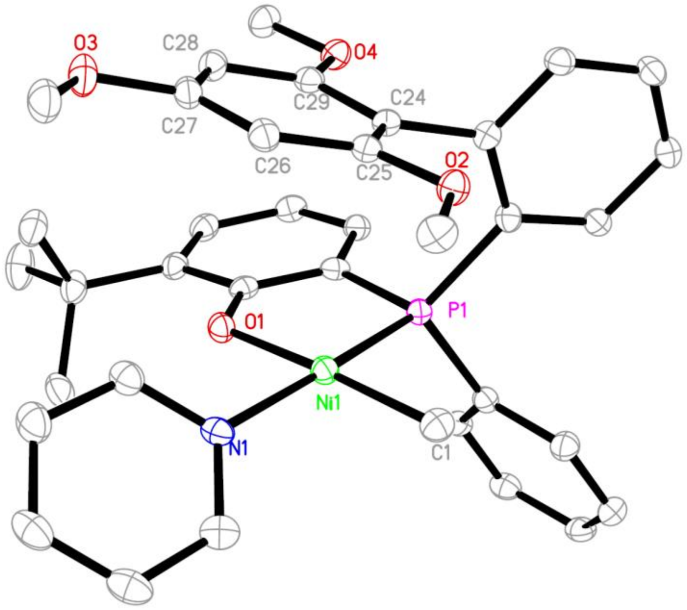

Figure S30. Crystal structure of complex **4**, hydrogen atoms are excluded for clarity.

Table S5. Crystal data and structure refinement of complex **4**.

Identification code	Complex **4**
CCDC number	2264076
Empirical formula	$C_{47.5}H_{51.5}NNiO_4P$
Formula weight	790.07
Temperature/K	100
Crystal system	triclinic
Space group	P-1
a/Å	11.0484(4)
b/Å	11.2489(4)
c/Å	16.5720(6)
α/°	77.252(3)
β/°	84.611(3)
γ/°	87.019(3)
Volume/Å^3	1998.92(13)
Z	2
ρ_{calc}g/cm^3	1.313
μ/mm^{-1}	0.571
F(000)	837.0
Crystal size/mm^3	0.45 × 0.217 × 0.05
Radiation	Mo Kα (λ = 0.71073)
2Θ range for data collection/°	3.704 to 53.704

Index ranges	$-13 \le h \le 13, -14 \le k \le 13, -20 \le l \le 20$
Reflections collected	24056
Independent reflections	8432 [R_{int} = 0.0239, R_{sigma} = 0.0203]
Data/restraints/parameters	8432/0/506
Goodness-of-fit on F^2	1.049
Final R indexes [I>=2σ (I)]	R_1 = 0.0397, wR_2 = 0.0881
Final R indexes [all data]	R_1 = 0.0506, wR_2 = 0.0986
Largest diff. peak/hole / e Å^{-3}	0.51/-0.52

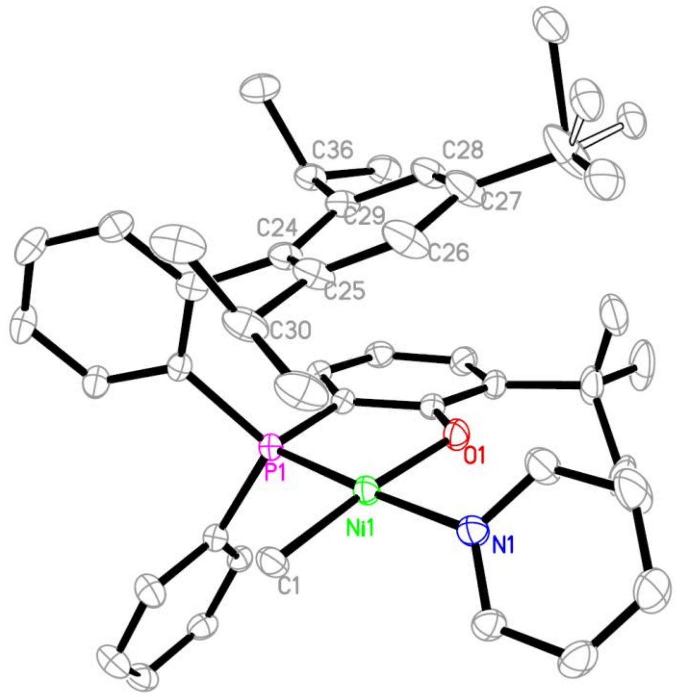

Figure S31. Crystal structure of complex **5**, hydrogen atoms are excluded for clarity.

Table S6. Crystal data and structure refinement of complex **5**.

Identification code	Complex **5**
CCDC number	2264074
Empirical formula	$C_{43}H_{51}NNiOP$
Formula weight	687.52
Temperature/K	100
Crystal system	triclinic
Space group	P-1
a/Å	10.9085(5)
b/Å	13.3205(7)
c/Å	14.4860(7)
α/°	79.806(4)

β/°	79.695(4)
γ/°	77.256(4)
Volume/Å^3	1999.09(18)
Z	2
ρ_{calc}g/cm^3	1.142
μ/mm^{-1}	0.556
F(000)	734.0
Crystal size/mm^3	0.42 × 0.313 × 0.15
Radiation	Mo Kα (λ = 0.71073)
2Θ range for data collection/°	3.87 to 53.616
Index ranges	-13 ≤ h ≤ 13, -16 ≤ k ≤ 16, -18 ≤ l ≤ 18
Reflections collected	21832
Independent reflections	8466 [R_{int} = 0.0263, R_{sigma} = 0.0268]
Data/restraints/parameters	8466/0/455
Goodness-of-fit on F^2	1.037
Final R indexes [I>=2σ (I)]	R_1 = 0.0411, wR_2 = 0.0901
Final R indexes [all data]	R_1 = 0.0561, wR_2 = 0.1008
Largest diff. peak/hole / e Å^{-3}	0.42/-0.47

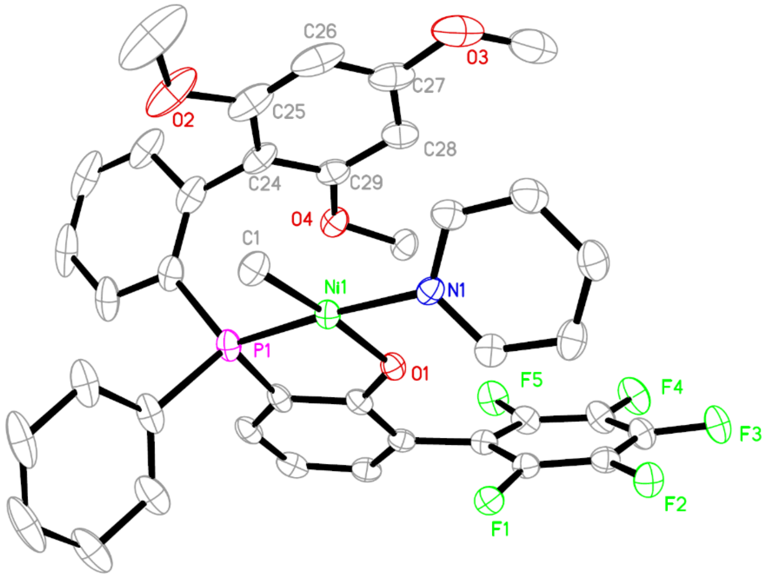

Figure S32. Crystal structure of complex **6**, hydrogen atoms are excluded for clarity.

Table S7. Crystal data and structure refinement of complex **6**.

Identification code	Complex **6**
CCDC number	2264075
Empirical formula	$C_{83}H_{74}F_{10}N_2Ni_2O_8P_2$
Formula weight	1596.80

Temperature/K	100
Crystal system	triclinic
Space group	P-1
a/Å	11.9660(3)
b/Å	16.7923(5)
c/Å	19.1982(5)
α/°	102.919(2)
β/°	105.241(2)
γ/°	92.280(2)
Volume/Å^3	3607.75(17)
Z	2
ρ_{calc}g/cm^3	1.470
μ/mm^{-1}	0.653
F(000)	1652.0
Crystal size/mm^3	0.15 × 0.103 × 0.04
Radiation	Mo Kα (λ = 0.71073)
2Θ range for data collection/°	3.638 to 53.794
Index ranges	-15 ≤ h ≤ 15, -21 ≤ k ≤ 21, -23 ≤ l ≤ 24
Reflections collected	41328
Independent reflections	15313 [R_{int} = 0.0396, R_{sigma} = 0.0569]
Data/restraints/parameters	15313/0/1060
Goodness-of-fit on F^2	1.054
Final R indexes [I>=2σ (I)]	R_1 = 0.0554, wR_2 = 0.1234
Final R indexes [all data]	R_1 = 0.1091, wR_2 = 0.1552
Largest diff. peak/hole / e Å^{-3}	1.37/-0.73

Refinement model description of Complex 6

Number of restraints - 0, number of constraints - unknown.

Details:

1. Fixed Uiso
 At 1.2 times of:
  All C(H) groups, All C(H,H) groups
 At 1.5 times of:
  All C(H,H,H) groups

2. Others
Sof(O9)=Sof(O10)=Sof(O11)=Sof(C72)=Sof(C73)=Sof(C74)=Sof(H74)=Sof(C75)=Sof(C76)=Sof(H76)=Sof(C77)=Sof(C78)=Sof(H78A)=Sof(H78B)=Sof(H78C)=Sof(C79)=Sof(H79A)=Sof(H79B)=Sof(H79C)=Sof(C80)=Sof(H80A)=Sof(H80B)=Sof(H80C)=1-FVAR(1)

Sof(O6)=Sof(O7)=Sof(O8)=Sof(C63)=Sof(C64)=Sof(C65)=Sof(H65)=Sof(C66)=Sof(C67)=Sof(H67)=Sof(C68)=Sof(C69)=Sof(H69A)=Sof(H69B)=Sof(H69C)=Sof(C70)=Sof(H70A)=Sof(H7

0B)=Sof(H70C)=Sof(C71)=Sof(H71A)=Sof(H71B)=Sof(H71C)=FVAR(1)

3.a Secondary CH2 refined with riding coordinates:
C81(H81A,H81B), C82(H82A,H82B), C87(H87A,H87B)

3.b Me refined with riding coordinates:
C71(H71A,H71B,H71C), C80(H80A,H80B,H80C)

3.c Aromatic/amide H refined with riding coordinates:
C4(H4), C5(H5), C6(H6), C13(H13), C14(H14), C15(H15), C16(H16), C17(H17), C19(H19), C20(H20), C21(H21), C22(H22), C26(H26), C28(H28), C33(H33), C34(H34), C35(H35), C36(H36), C37(H37), C43(H43), C44(H44), C45(H45), C52(H52), C53(H53), C54(H54), C55(H55), C56(H56), C58(H58), C59(H59), C60(H60), C61(H61), C65(H65), C67(H67), C74(H74), C76(H76), C84(H84), C86(H86), C88(H88), C90(H90), C92(H92)

3.d Fitted hexagon refined as free rotating group:
C63(C64,C65,C66,C67,C68), C72(C73,C74,C75,C76,C77)

3.e Idealised Me refined as rotating group:
C1(H1A,H1B,H1C), C30(H30A,H30B,H30C), C31(H31A,H31B,H31C), C32(H32A,H32B,H32C), C40(H40A,H40B,H40C), C69(H69A,H69B,H69C), C70(H70A,H70B,H70C), C78(H78A,H78B,H78C), C79(H79A,H79B,H79C), C83(H83A,H83B,H83C), C89(H89A,H89B,H89C)

2 x benzene with AFIX 66 (for disordered positions)

Table S8. Selected distances (Å) in crystal structure of complexes **1**-**6**.

Entry	Complex	Ni-C1 (Å)	Ni-N (Å)	Ni-P (Å)	Ni-O (Å)	Ni-C24 (Å)	Ni-Ar*[a] (Å)
1	**1**	1.941	1.991	2.105	1.918	3.406	3.627
2	**2**	1.939	1.949	2.115	1.925	3.301	3.545
3	**3**	1.970	1.954	2.110	1.924	3.331	3.569
4	**4**	1.931	1.947	2.123	1.919	3.394	3.581
5	**5**	1.940	1.940	2.120	1.916	3.541	3.852
6	**6**	1.946	1.961	2.137	1.928	3.293	3.448

[a] Ni-Ar*: distance between Ni and the center of the aromatic group (aryl) of the 2-(aryl)phenyl.

2.4 Cyclic Voltammetry

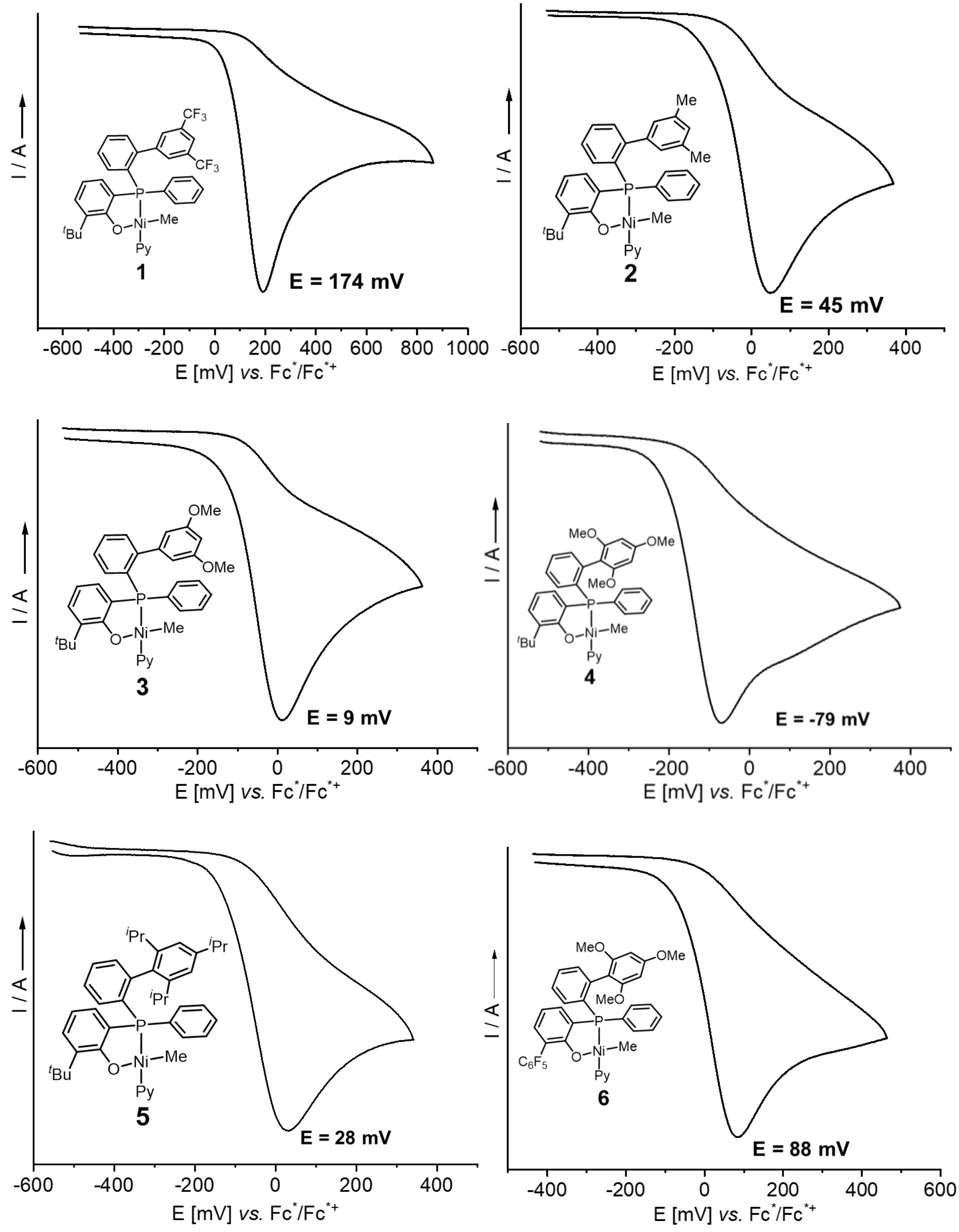

Figure S33. Cyclic voltammograms of complexes **1**-**6**.

3 Ethylene Polymerization

3.1 Ethylene Polymerization Procedure

Polymerizations were conducted in a Büchi miniclave reactor with a 200 mL vessel, equipped with a heating and cooling jacket connected to a thermostat, a mechanical stirrer, a nitrogen/vacuum supply and a thermocouple couple dipping into the reaction mixture. Ethylene was supplied via two Bronkhorst mass flow meters (up to 15 g h^{-1} and 150 g h^{-1} ethylene), the flow being controlled via a pressure meter and a compressed air-driven badger valve. Prior to all polymerization experiments, the reactor was evacuated and heated up (thermostat temperature: 90 °C). When the reactor temperature was > 60 °C, the reactor was flushed with nitrogen and evacuated three times. The reactor was brought 5 °C below the desired reaction temperature. The reactor was then filled with 100 mL of toluene via cannula transfer and the solution stirred with 500 rpm. 5 µmol of catalyst precursor was dissolved in 5 mL of toluene, and 1 mL of this solution was transferred into the reactor via syringe. Immediately after addition, the stirring rate was increased to 1000 rpm and the reactor was pressurized to the desired pressure. During the pressurization procedure the temperature was adjusted to the desired reaction temperature. A constant pressure was maintained over the entire polymerization experiment by feeding ethylene. After the desired reaction time, the ethylene flow was stopped, and the reactor carefully vented. The reactor content was poured into 300 mL of methanol and stirred for 30 minutes. The precipitated polymer was filtrated, washed with methanol and dried in a vacuum oven (60 °C, 30 mbar) overnight.

3.2 Mass Flow Profiles of Ethylene Polymerizations

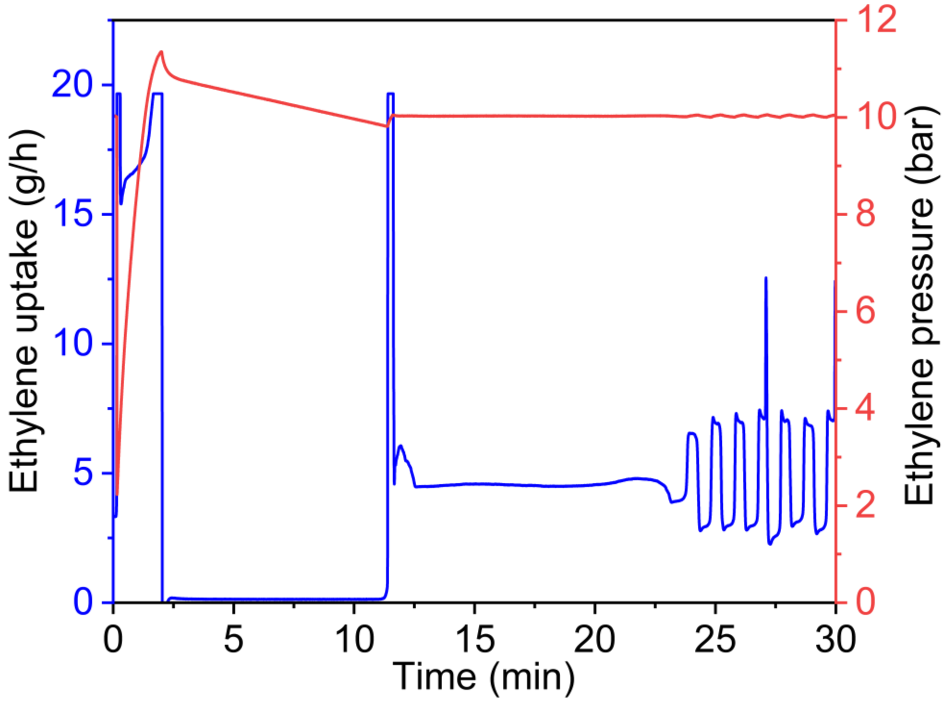

Figure S34. Ethylene uptake of ethylene polymerization by complex **1** at 30 ºC (Table 1, entry 1).

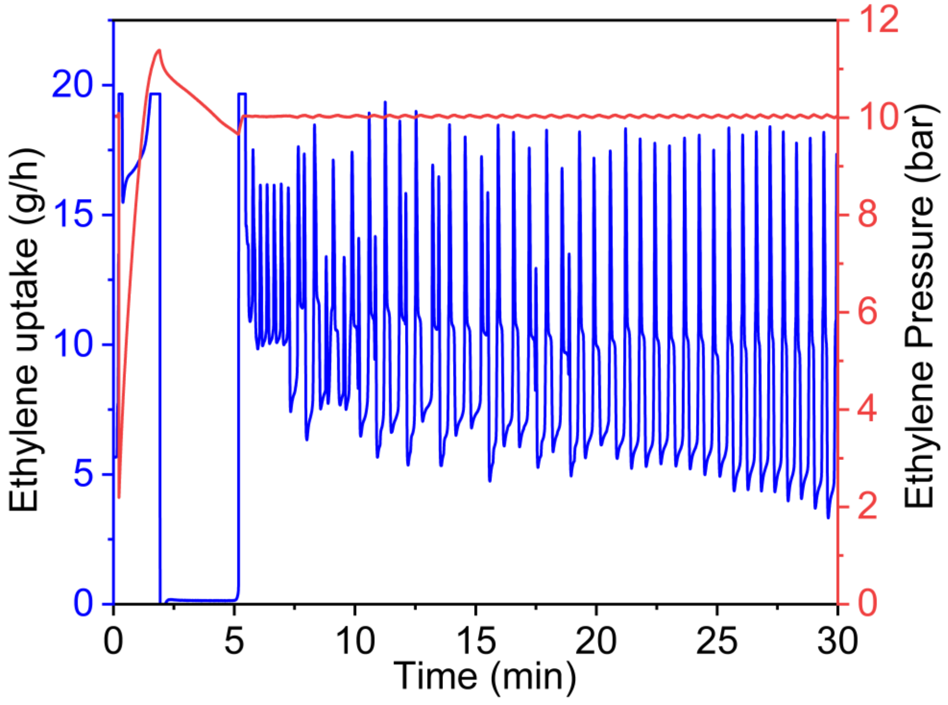

Figure S35. Ethylene uptake of ethylene polymerization by complex **1** at 50 ºC (Table 1, entry 2).

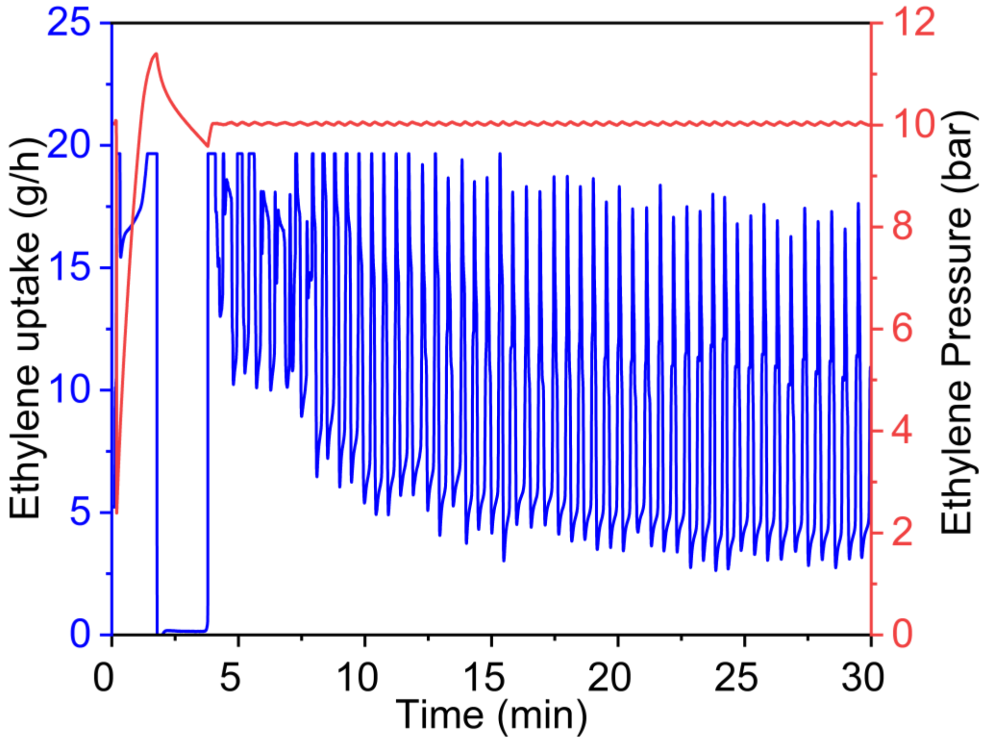

Figure S36. Ethylene uptake of ethylene polymerization by complex **1** at 70 ºC (Table 1, entry 3).

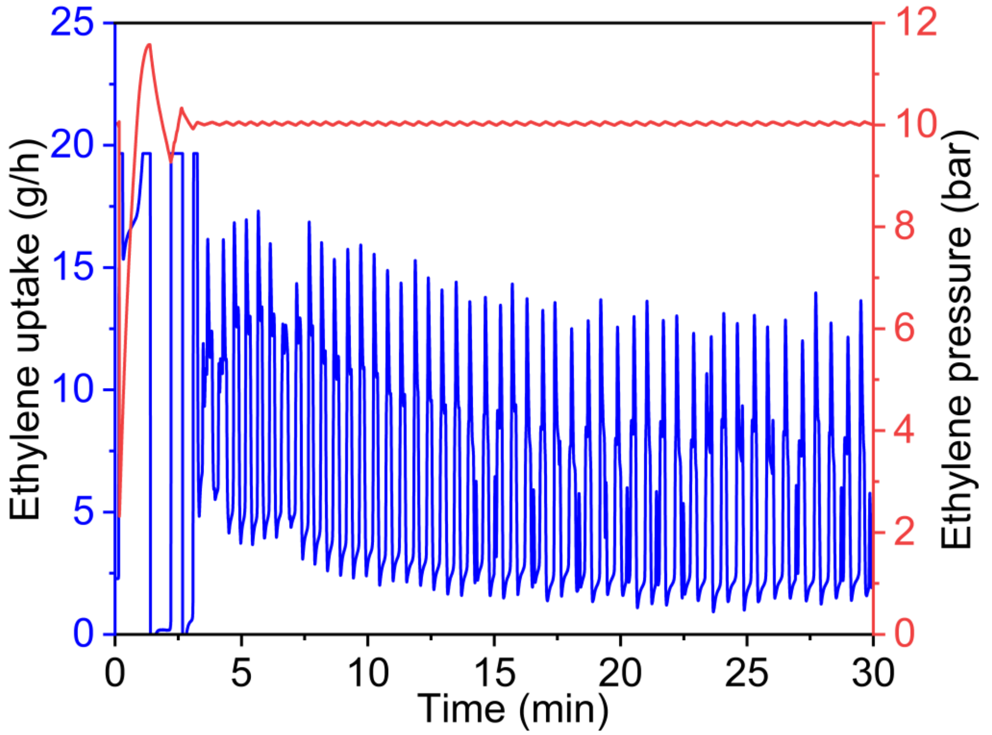

Figure S37. Ethylene uptake of ethylene polymerization by complex **2** at 70 ºC(Table 1, entry 6).

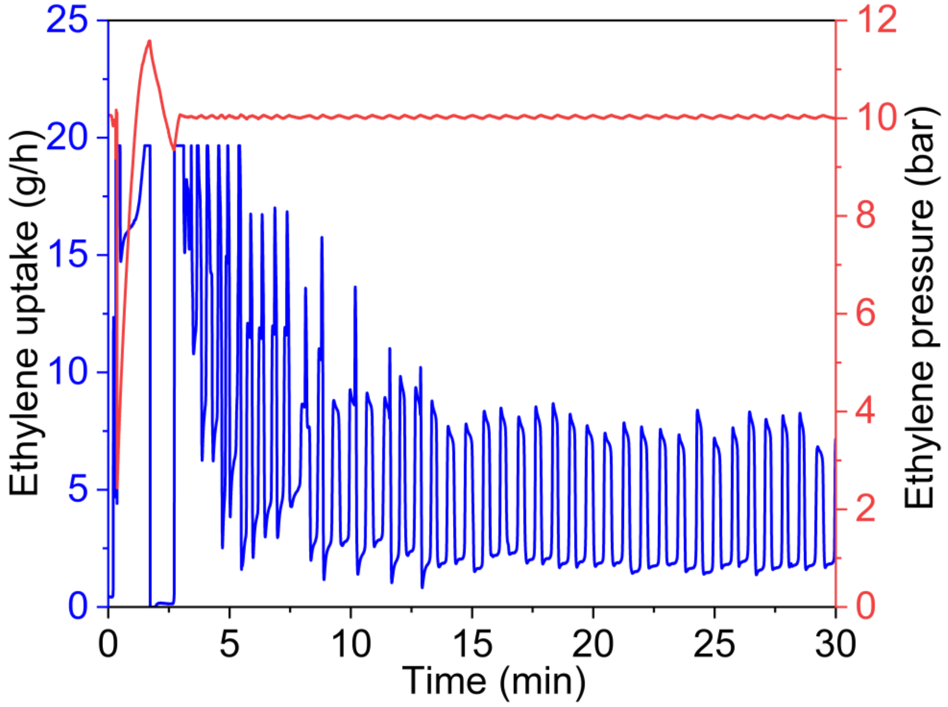

Figure S38. Ethylene uptake of ethylene polymerization by complex **3** at 70 ºC (Table 1, entry 9).

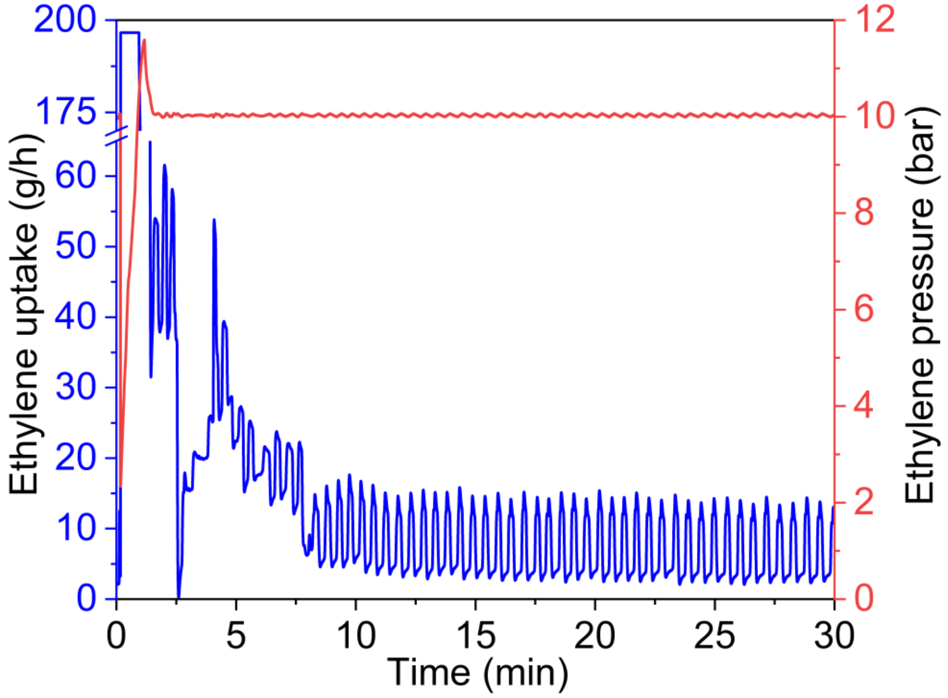

Figure S39. Ethylene uptake of ethylene polymerization by complex **4** at 30 ºC (Table 1, entry 10).

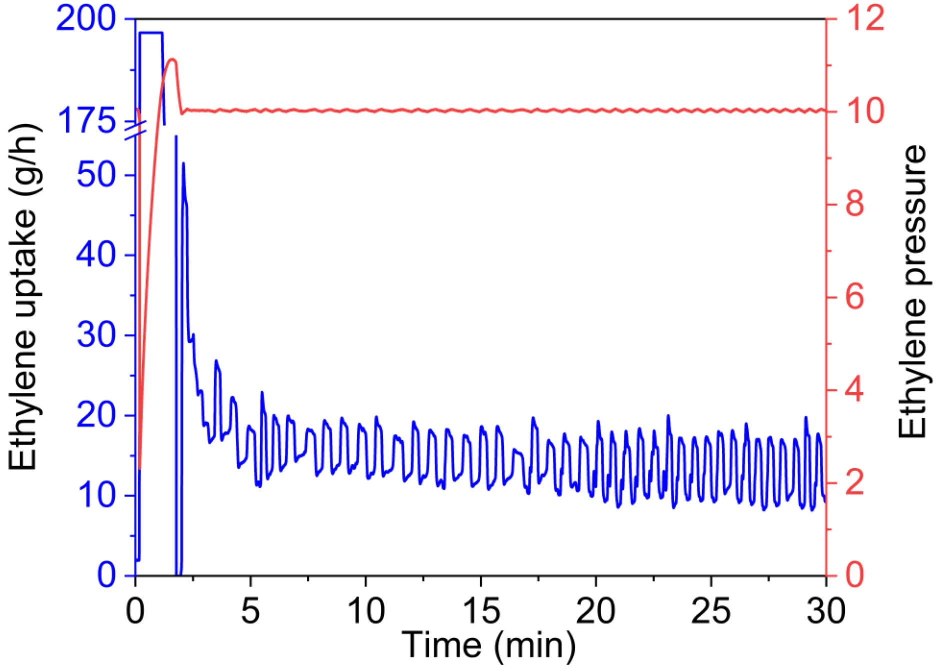

Figure S40. Ethylene uptake of ethylene polymerization by complex **4** at 50 ºC (Table 1, entry 11).

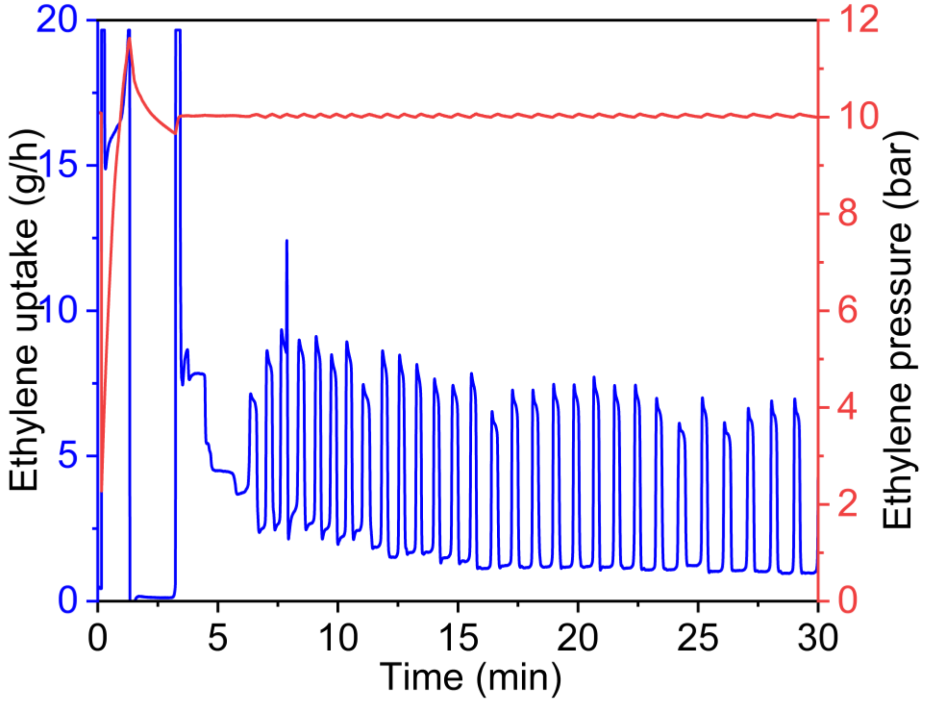

Figure S41. Ethylene uptake of ethylene polymerization by complex **4** at 70 ºC (Table 1, entry 12).

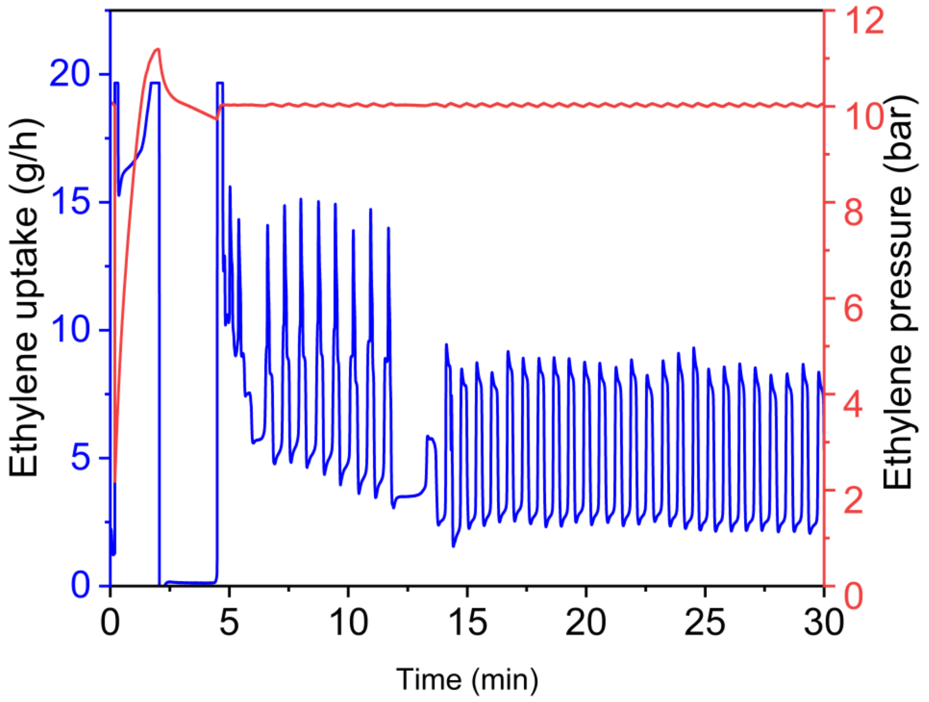

Figure S42. Ethylene uptake of ethylene polymerization by complex **5** at 30 ºC(Table 1, entry 13).

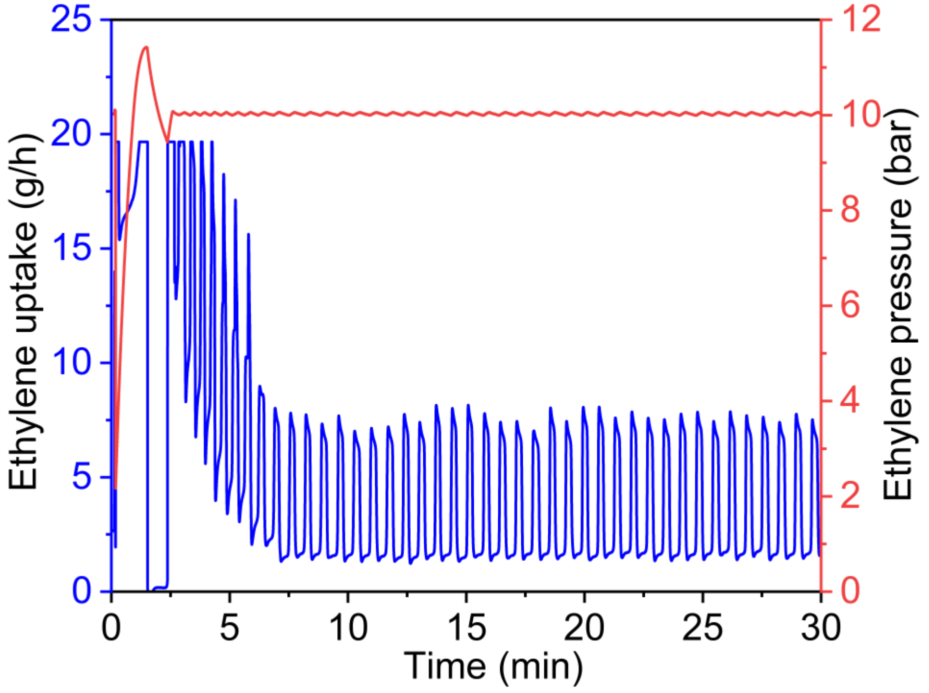

Figure S43. Ethylene uptake of ethylene polymerization by complex **5** at 50 ºC(Table 1, entry 14).

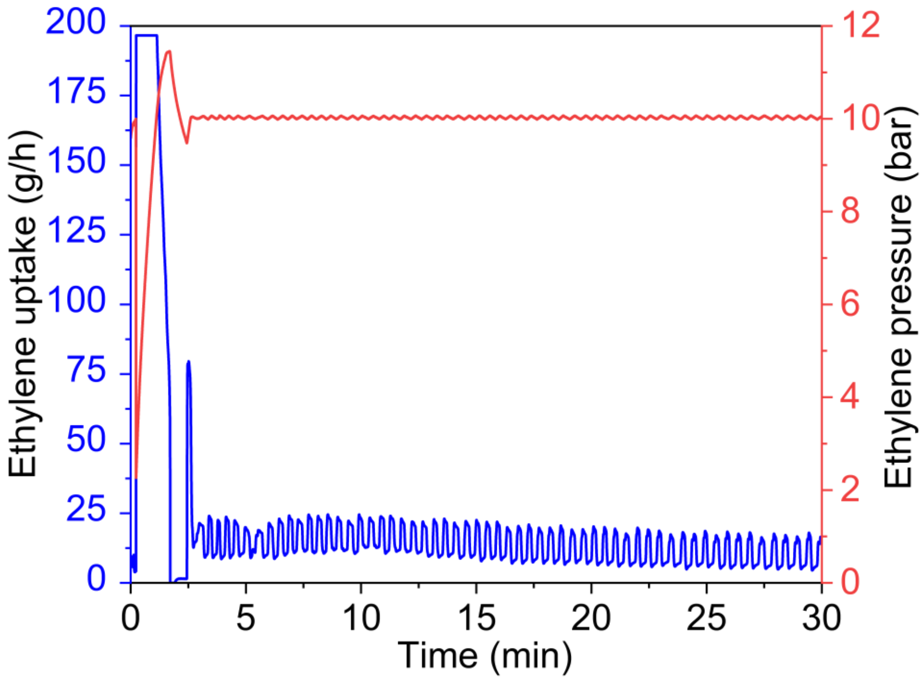

Figure S44. Ethylene uptake of ethylene polymerization by complex **5** at 70 ºC (Table 1, entry 15).

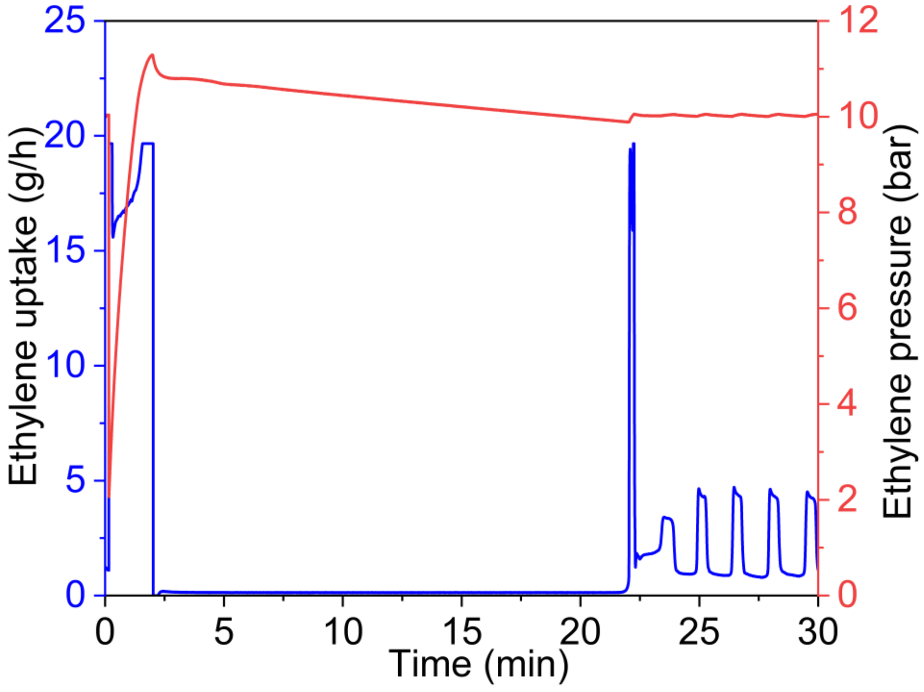

Figure S45. Ethylene uptake of ethylene polymerization by complex **6** at 30 ºC (Table 1, entry 16).

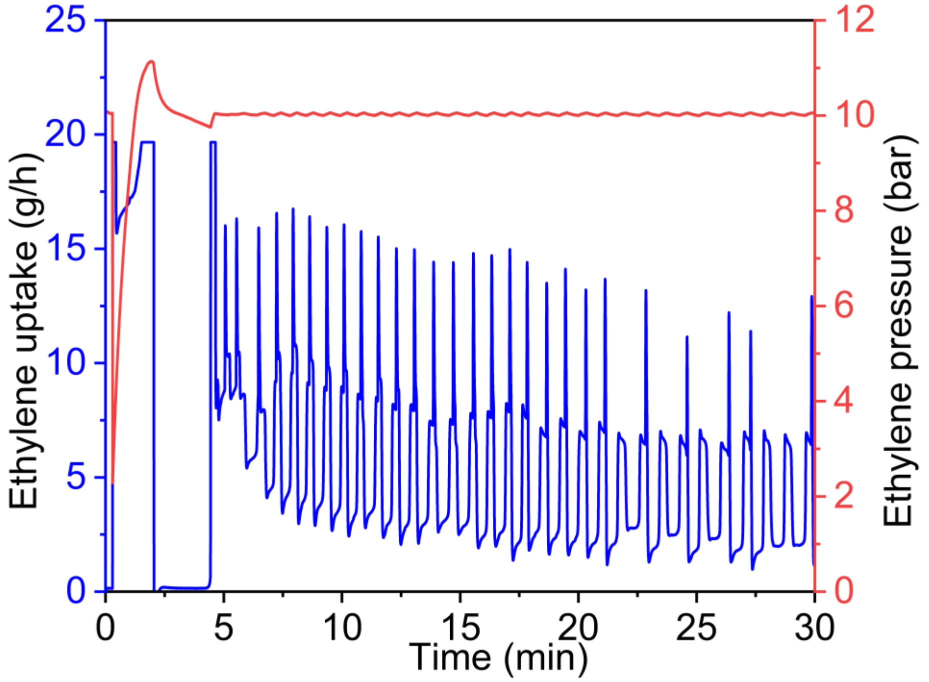

Figure S46. Ethylene uptake of ethylene polymerization by complex **6** at 50 ºC (Table 1, entry 17).

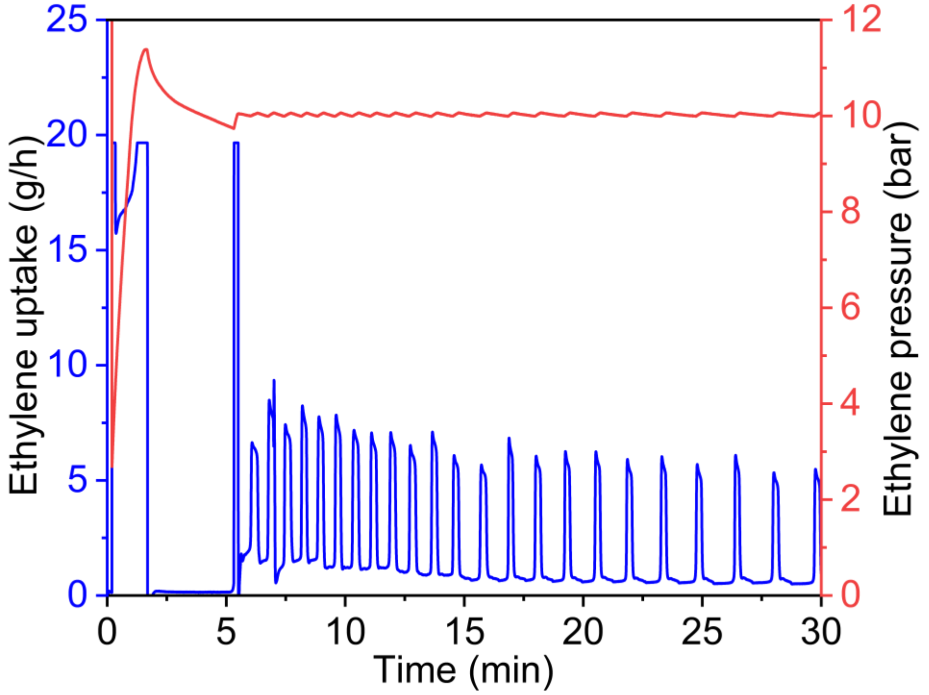

Figure S47. Ethylene uptake of ethylene polymerization by complex **6** at 70 ºC (Table 1, entry 18).

4 Polymer Characterization

4.1 NMR Analysis

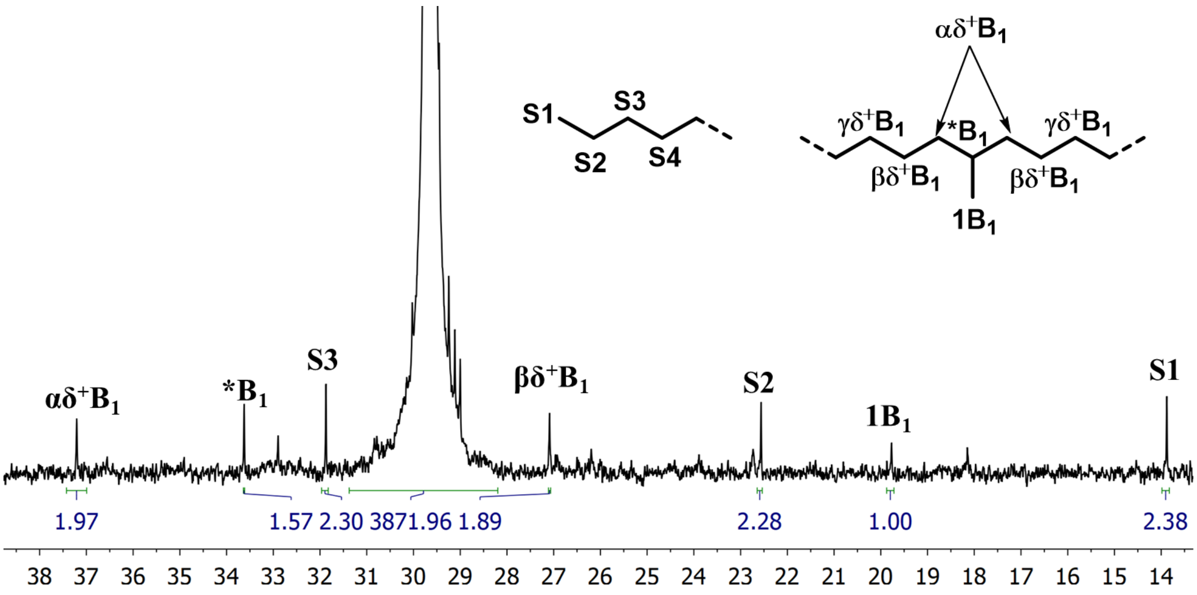

Figure S48. $^{13}C\{^{1}H\}$ NMR spectrum (101 MHz, $C_2D_2Cl_4$, 120 ºC) of polyethylene obtained (Table 1, entry 7, complex **3**), 0.2 branches per 1000 C atoms.

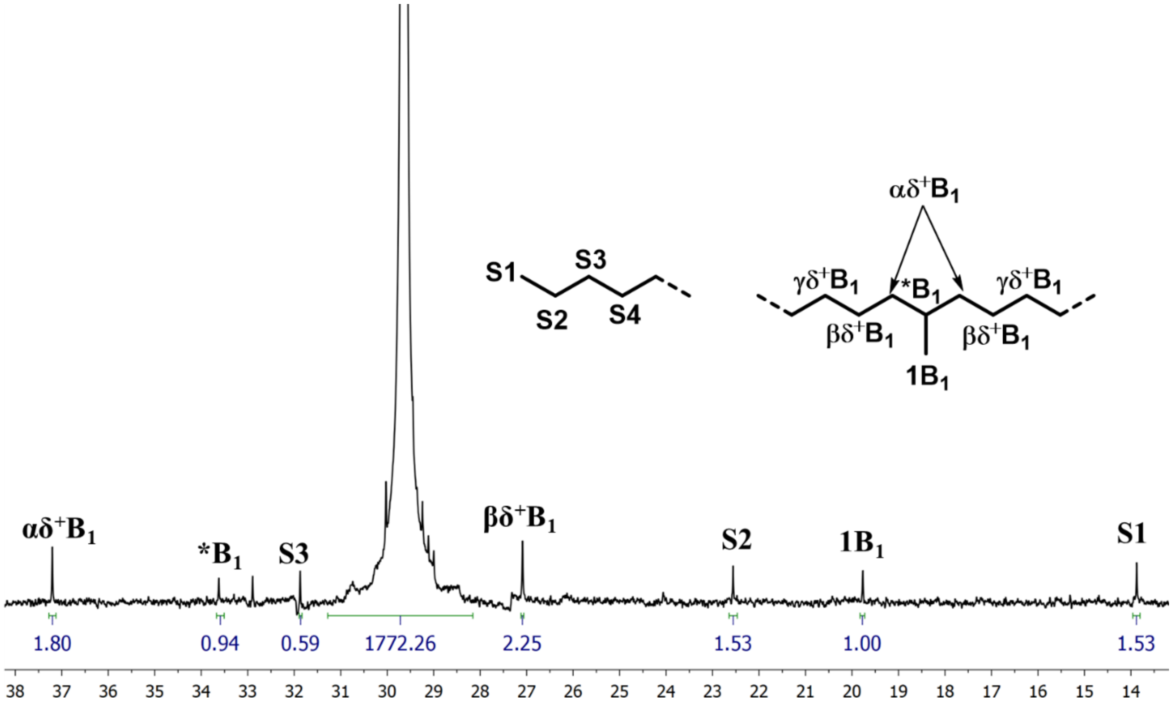

Figure S49. $^{13}C\{^{1}H\}$ NMR spectrum (101 MHz, $C_2D_2Cl_4$, 120 ºC) of polyethylene obtained (Table 1, entry 13, complex **5**), 0.6 branches per 1000 C atoms.

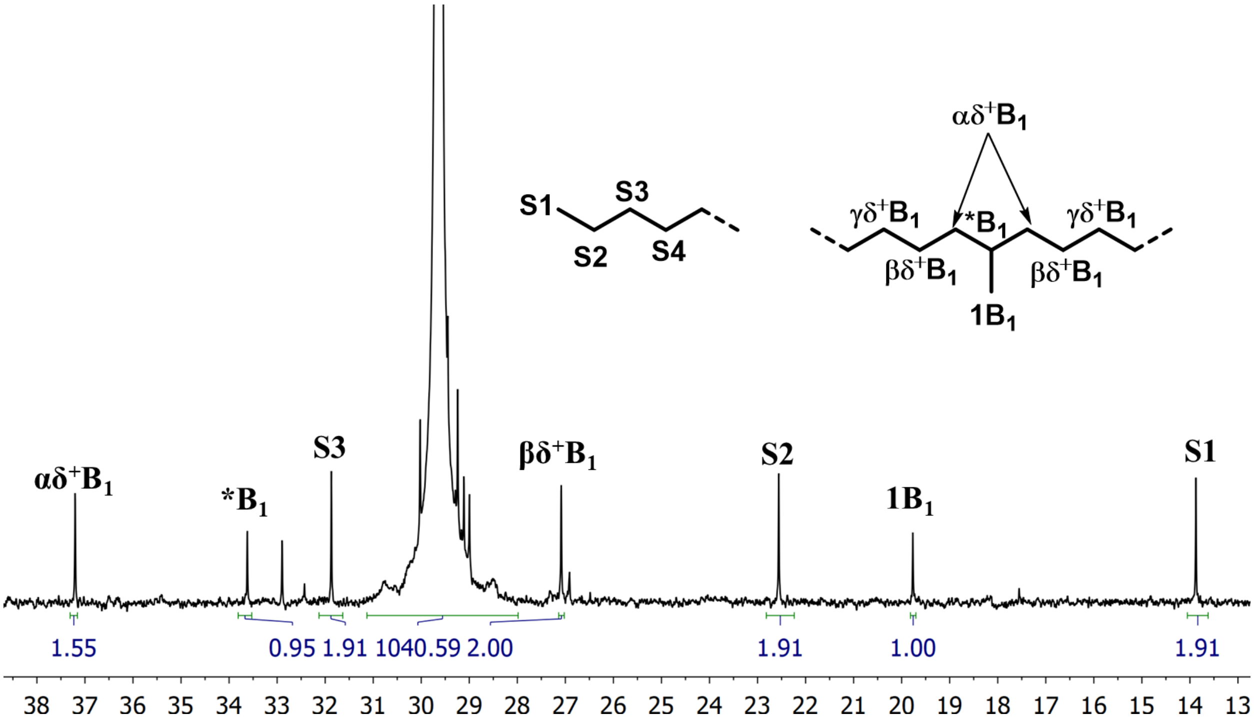

Figure S50. $^{13}C\{^{1}H\}$ NMR spectrum (101 MHz, $C_2D_2Cl_4$, 120 ºC) of polyethylene obtained (Table 1, entry 14, complex **5**), 1.0 branches per 1000 C atoms.

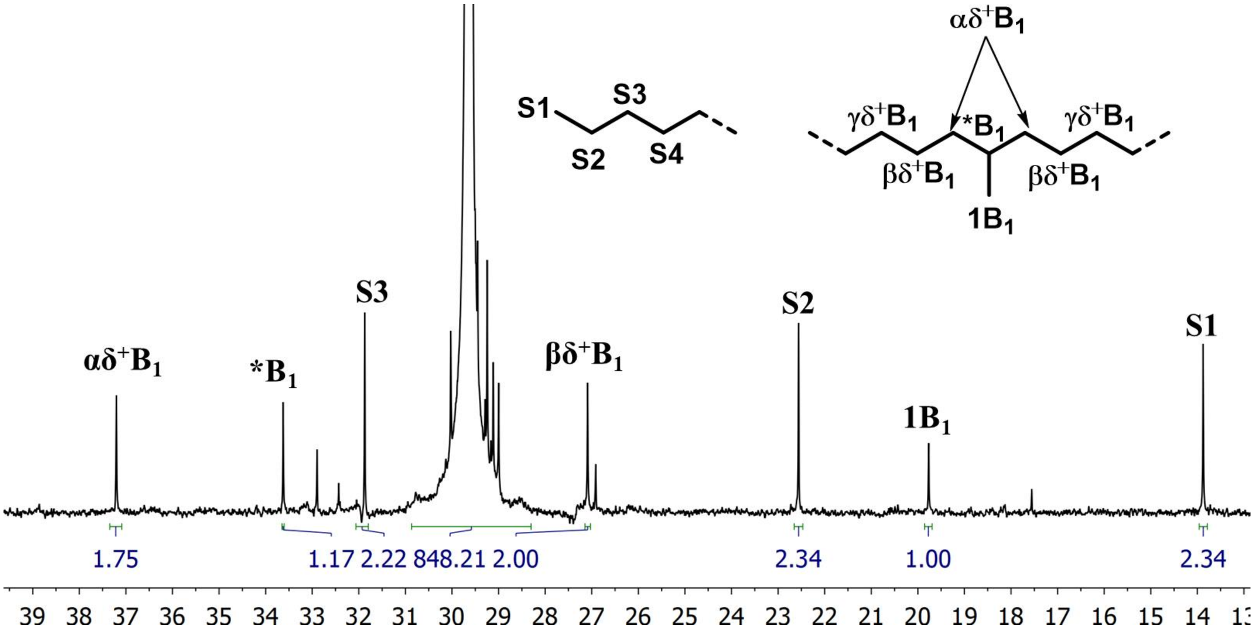

Figure S51. $^{13}C\{^{1}H\}$ NMR spectrum (101 MHz, $C_2D_2Cl_4$, 120 ºC) of polyethylene obtained (Table 1, entry 15, complex **5**), 1.2 branches per 1000 C atoms.

4.2 DSC traces

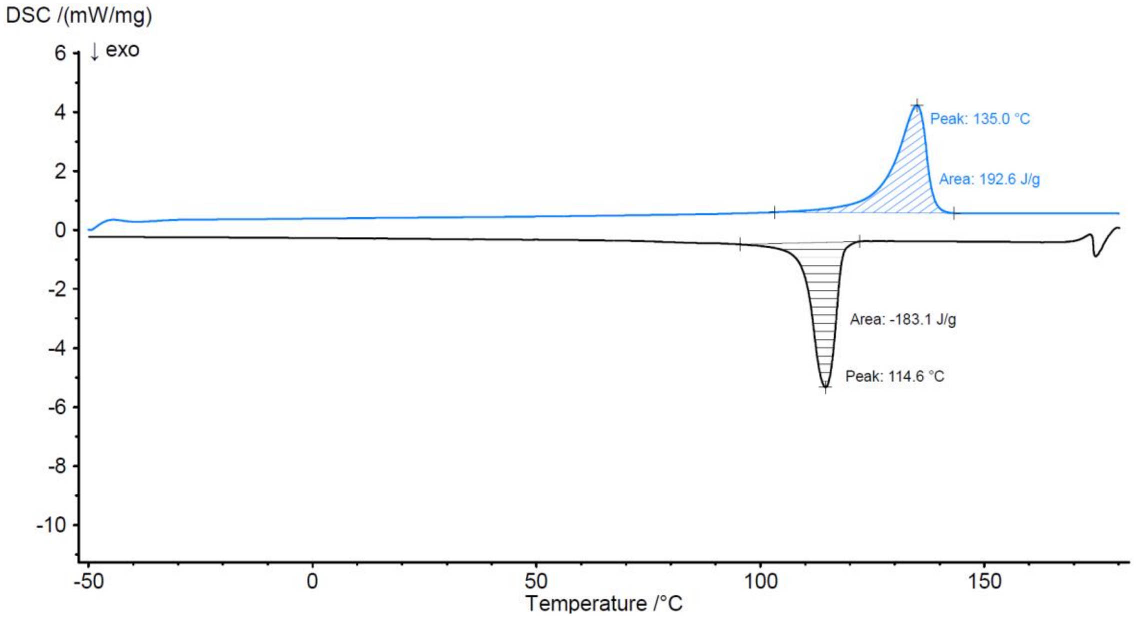

Figure S52. DSC traces of polyethylene obtained with complex **1** (Table 1, entry 1), measured with 10 K min^{-1} heating/cooling rate (black curve, 1st cooling; blue curve: 2nd heating).

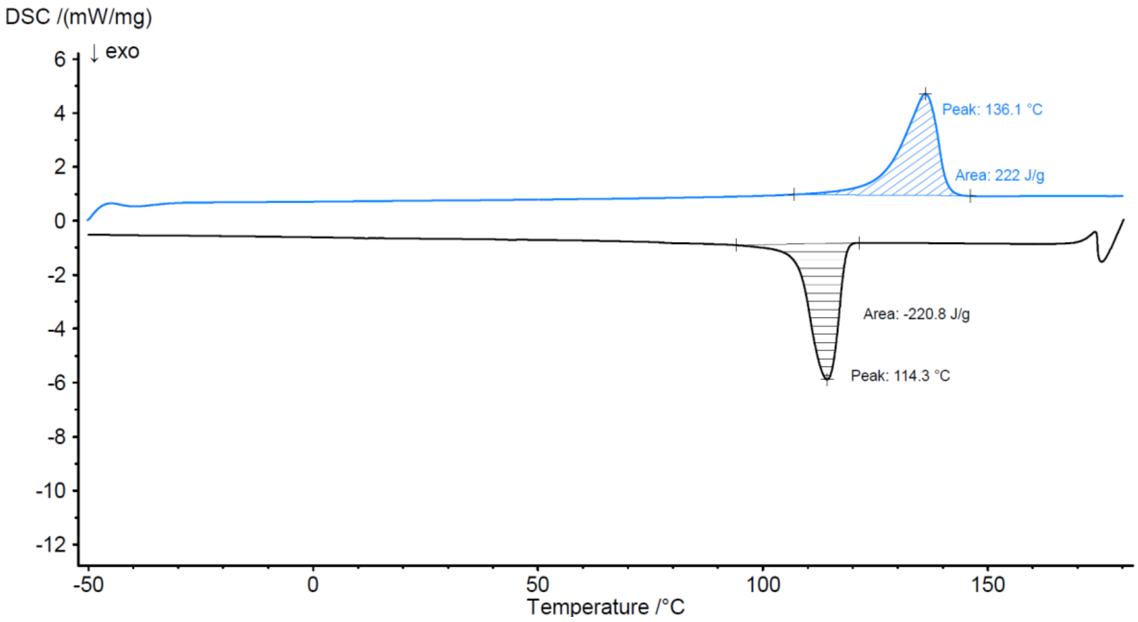

Figure S53. DSC traces of polyethylene obtained with complex **1** (Table 1, entry 2), measured with 10 K min^{-1} heating/cooling rate (black curve, 1st cooling; blue curve: 2nd heating).

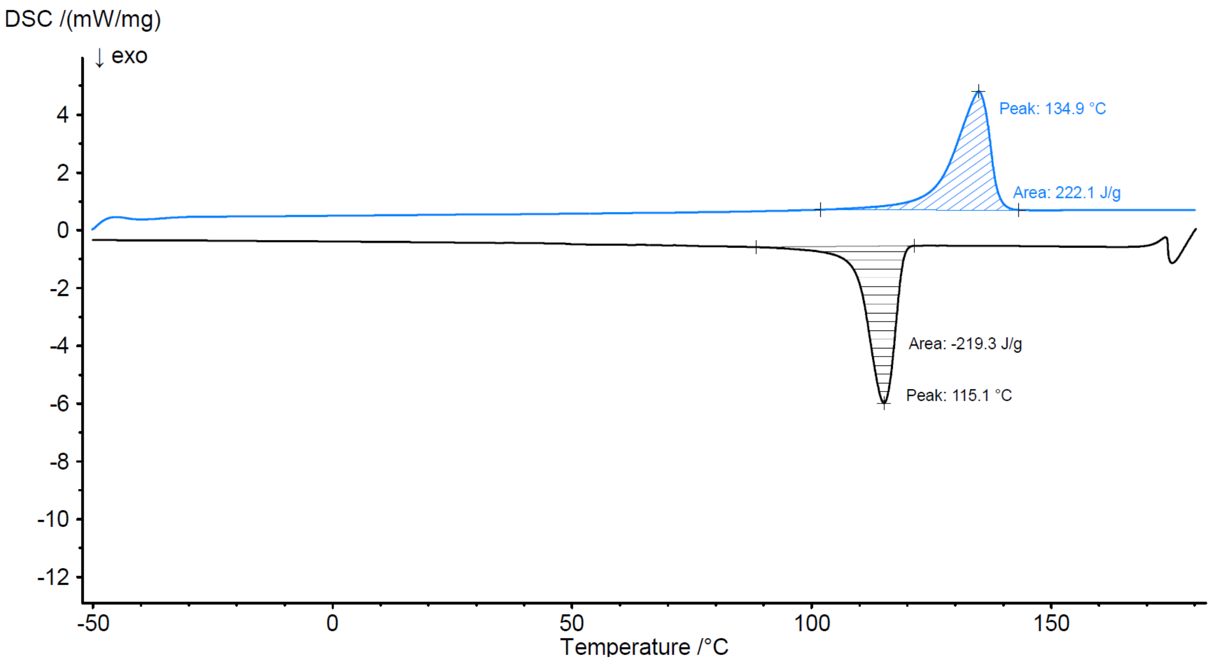

Figure S54. DSC traces of polyethylene obtained with complex **1** (Table 1, entry 3), measured with 10 K min^{-1} heating/cooling rate (black curve, 1st cooling; blue curve: 2nd heating).

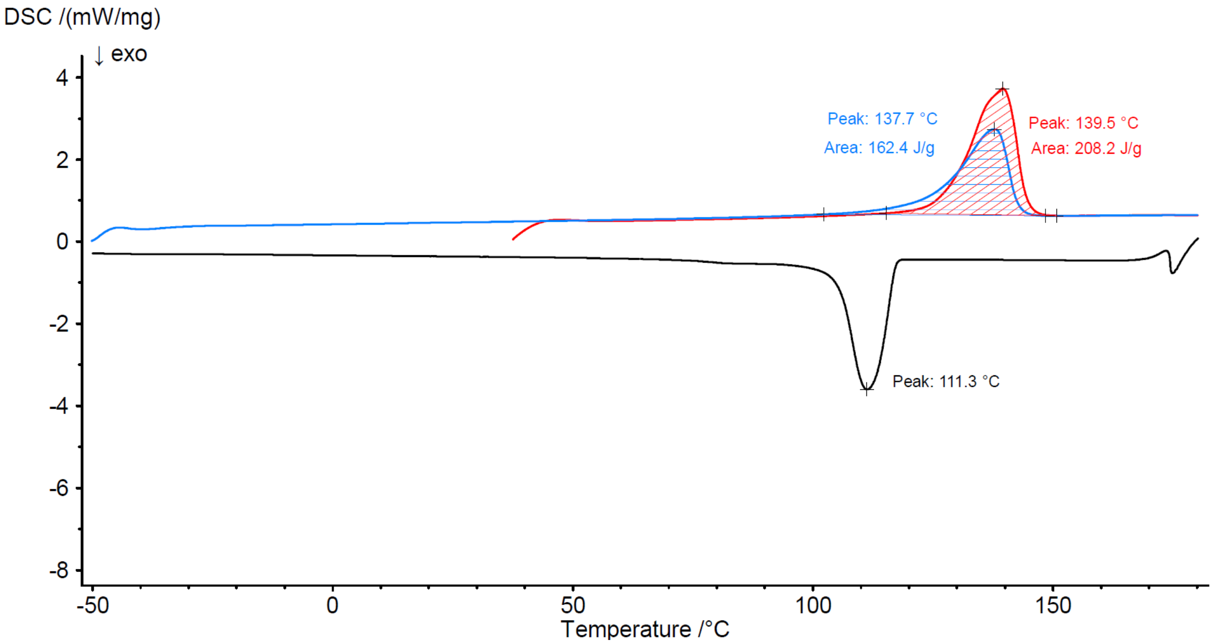

Figure S55. DSC traces of polyethylene obtained with complex **2** (Table 1, entry 6), measured with 10 K min^{-1} heating/cooling rate (red curve, 1st heating; black curve, 1st cooling; blue curve: 2nd heating).

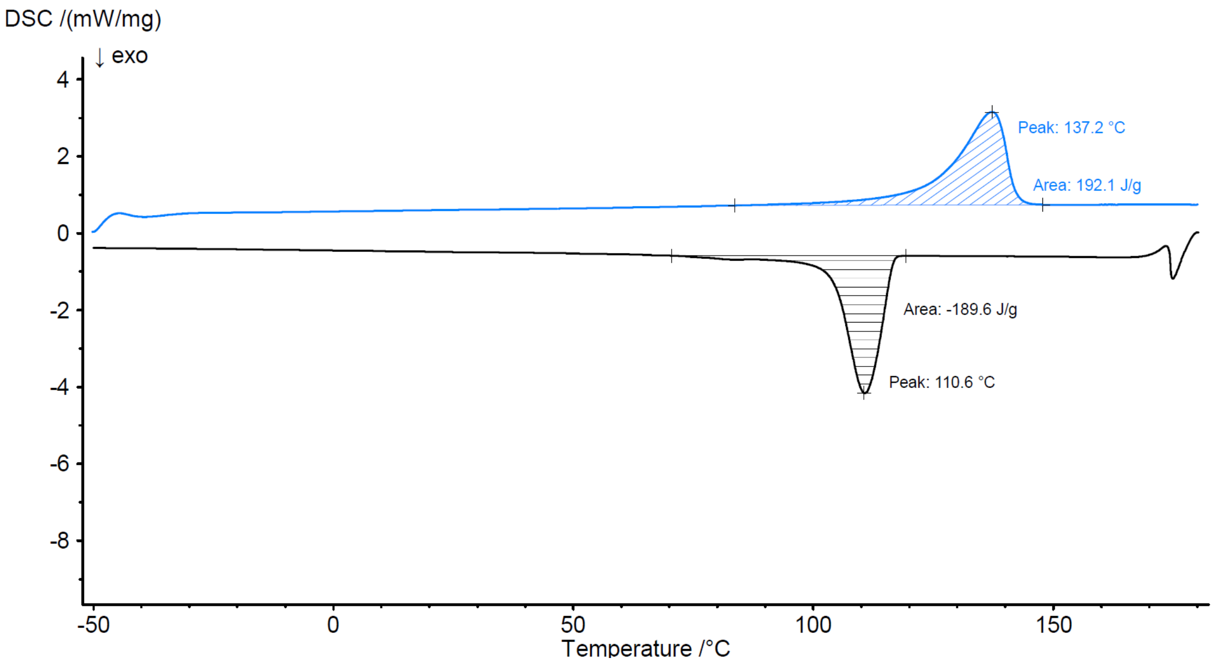

Figure S56. DSC traces of polyethylene obtained with complex **3** (Table 1, entry 9), measured with 10 K min^{-1} heating/cooling rate (black curve, 1st cooling; blue curve: 2nd heating).

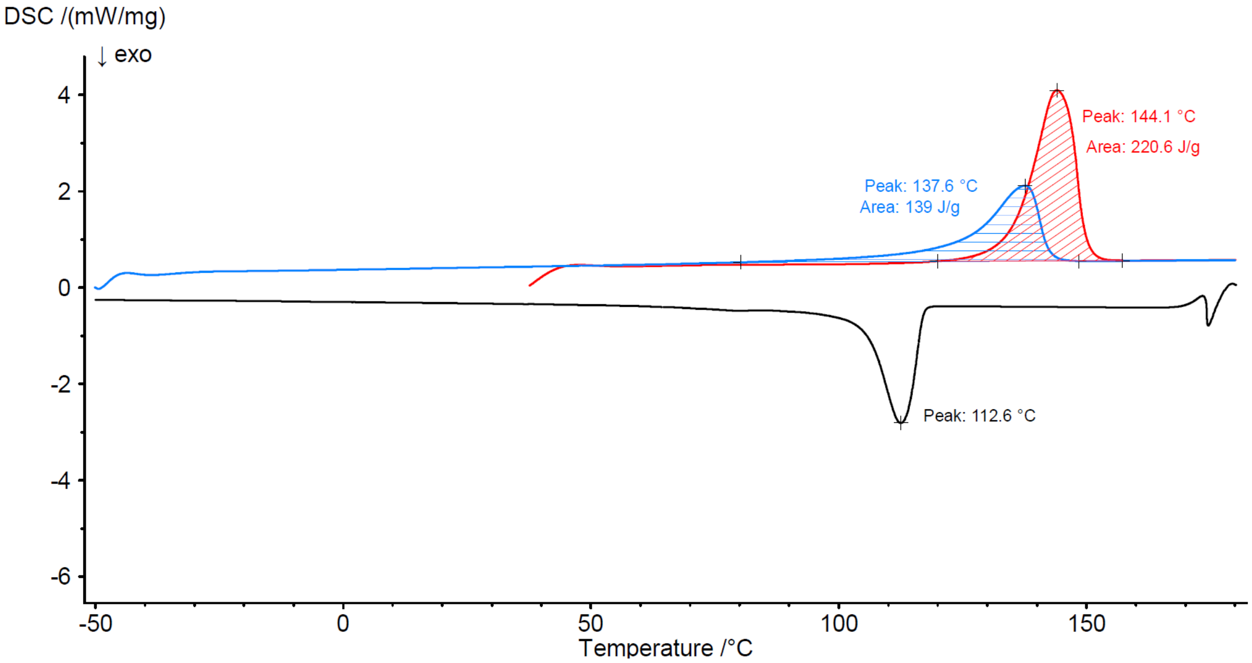

Figure S57. DSC traces of polyethylene obtained with complex **4** (Table 1, entry 10), measured with 10 K min^{-1} heating/cooling rate (red curve, 1st heating; black curve, 1st cooling; blue curve: 2nd heating).

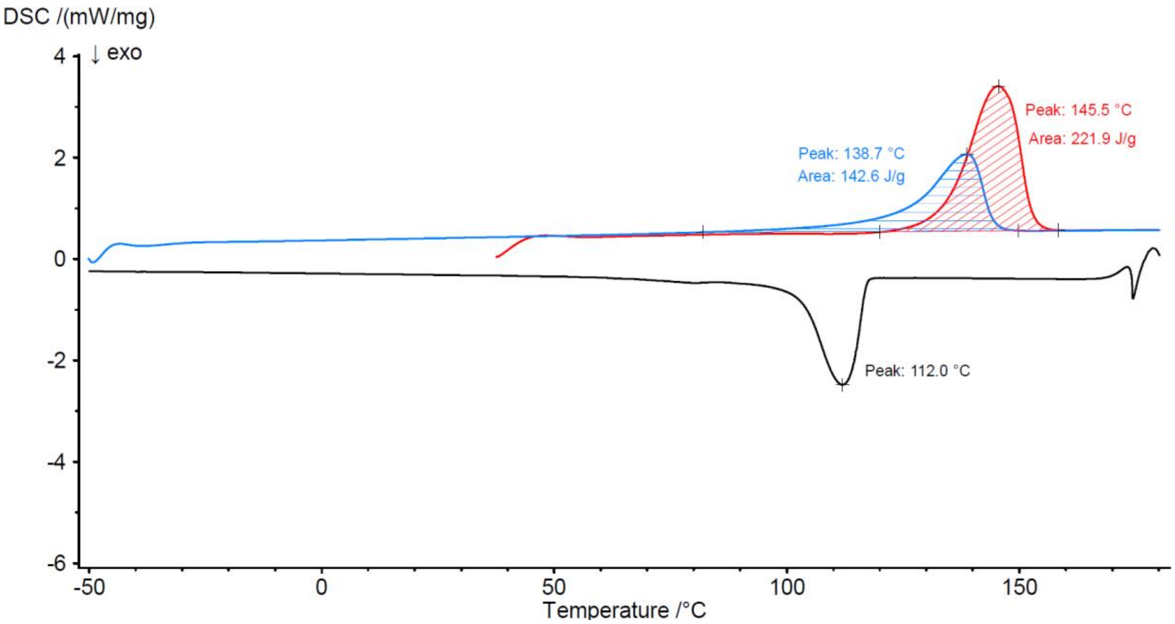

Figure S58. DSC traces of polyethylene obtained with complex **4** (Table 1, entry 11), measured with 10 K min^{-1} heating/cooling rate (red curve, 1st heating; black curve, 1st cooling; blue curve: 2nd heating).

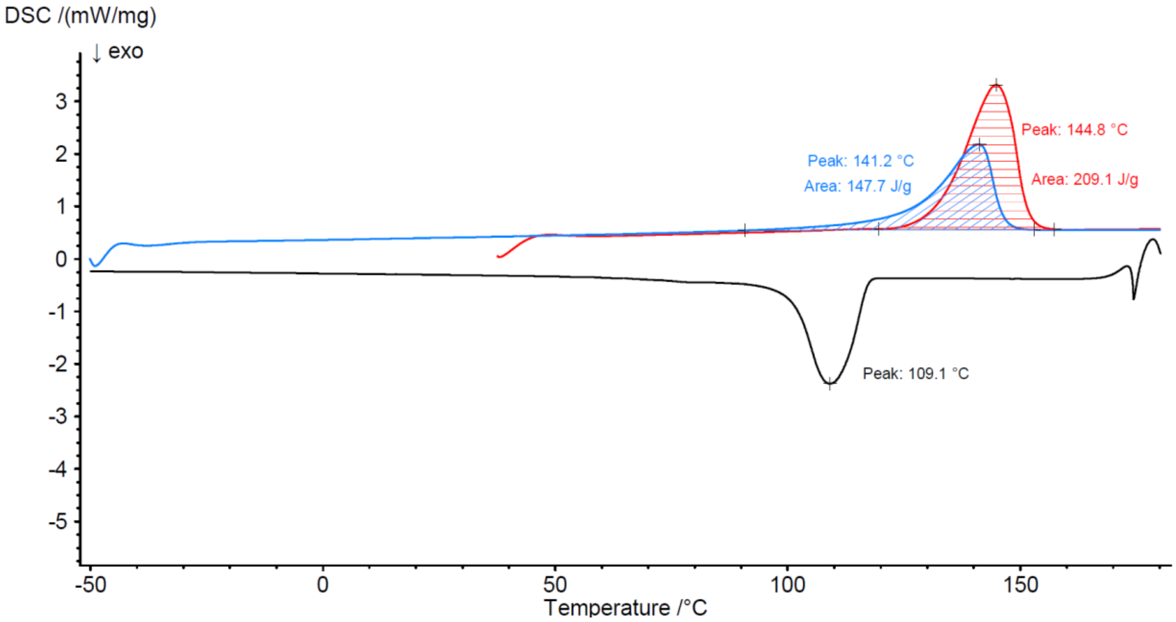

Figure S59. DSC traces of polyethylene obtained with complex **4** (Table 1, entry 12), measured with 10 K min^{-1} heating/cooling rate (red curve, 1st heating; black curve, 1st cooling; blue curve: 2nd heating).

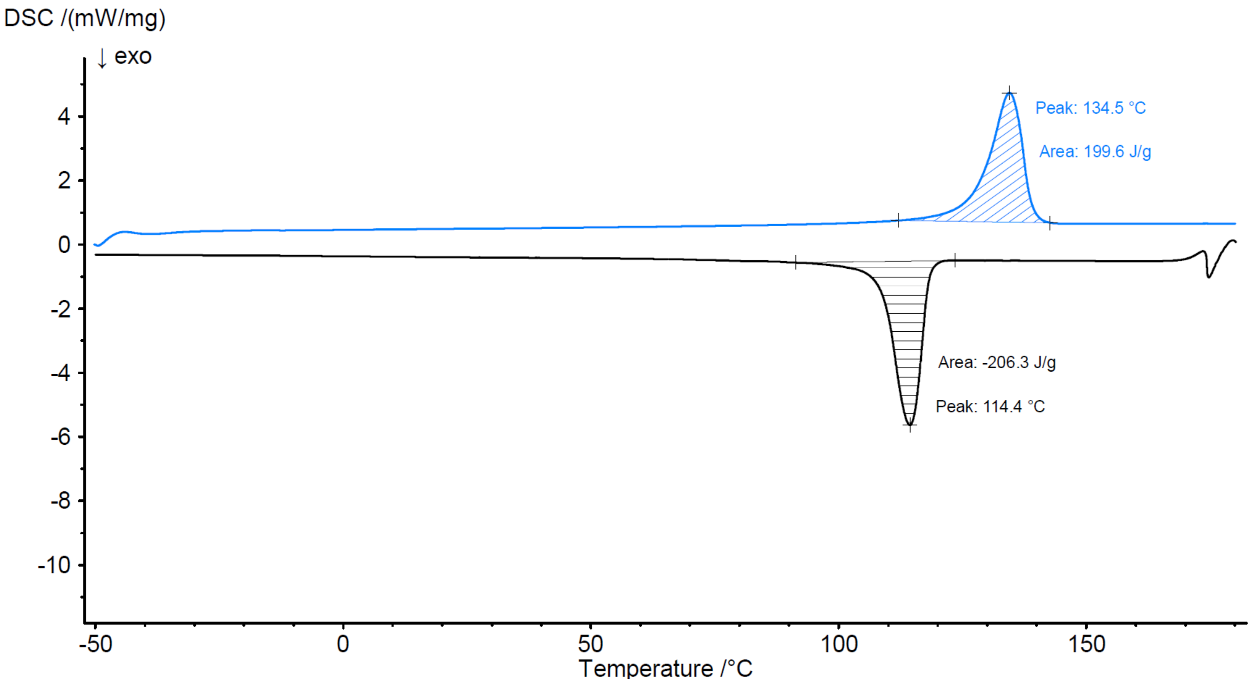

Figure S60. DSC traces of polyethylene obtained with complex **5** (Table 1, entry 13), measured with 10 K min^{-1} heating/cooling rate (black curve, 1st cooling; blue curve: 2nd heating).

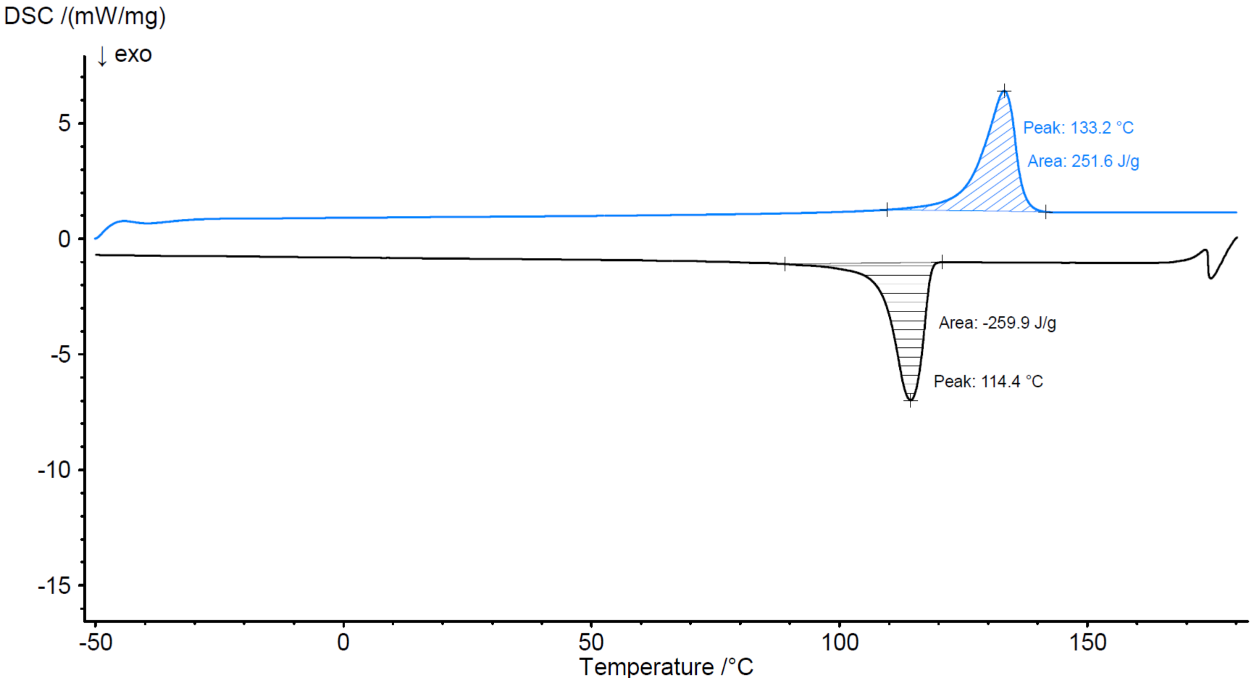

Figure S61. DSC traces of polyethylene obtained with complex **5** (Table 1, entry 14), measured with 10 K min^{-1} heating/cooling rate (black curve, 1st cooling; blue curve: 2nd heating).

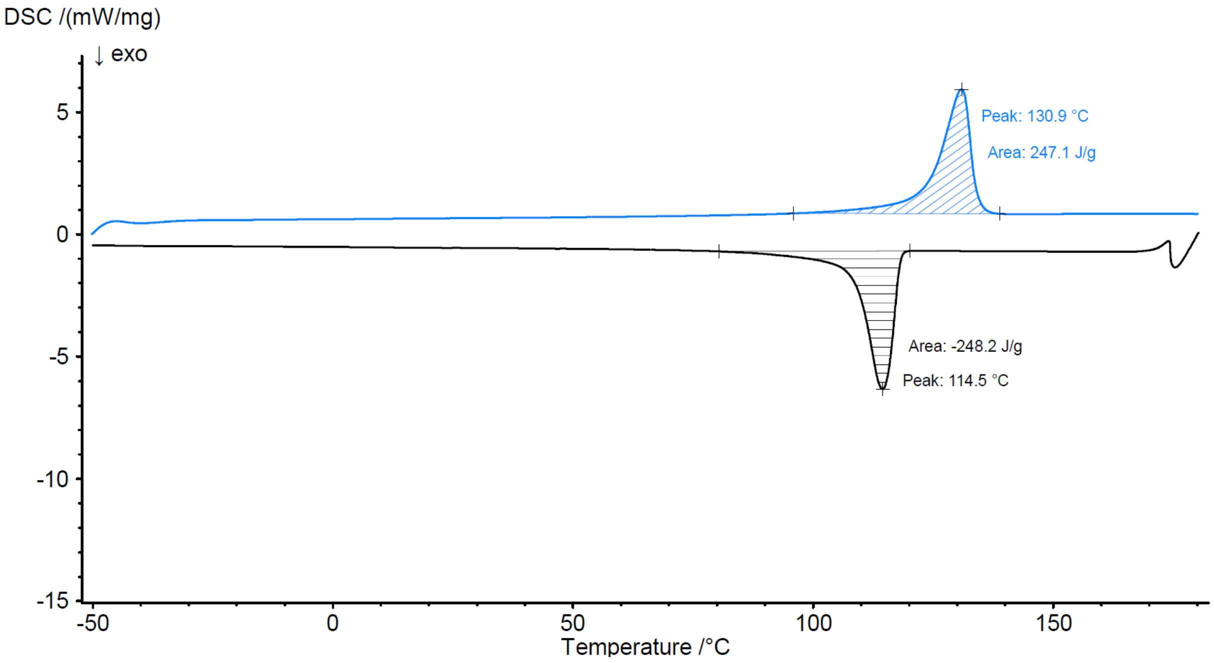

Figure S62. DSC traces of polyethylene obtained with complex **5** (Table 1, entry 15), measured with 10 K min^{-1} heating/cooling rate (black curve, 1st cooling; blue curve: 2nd heating).

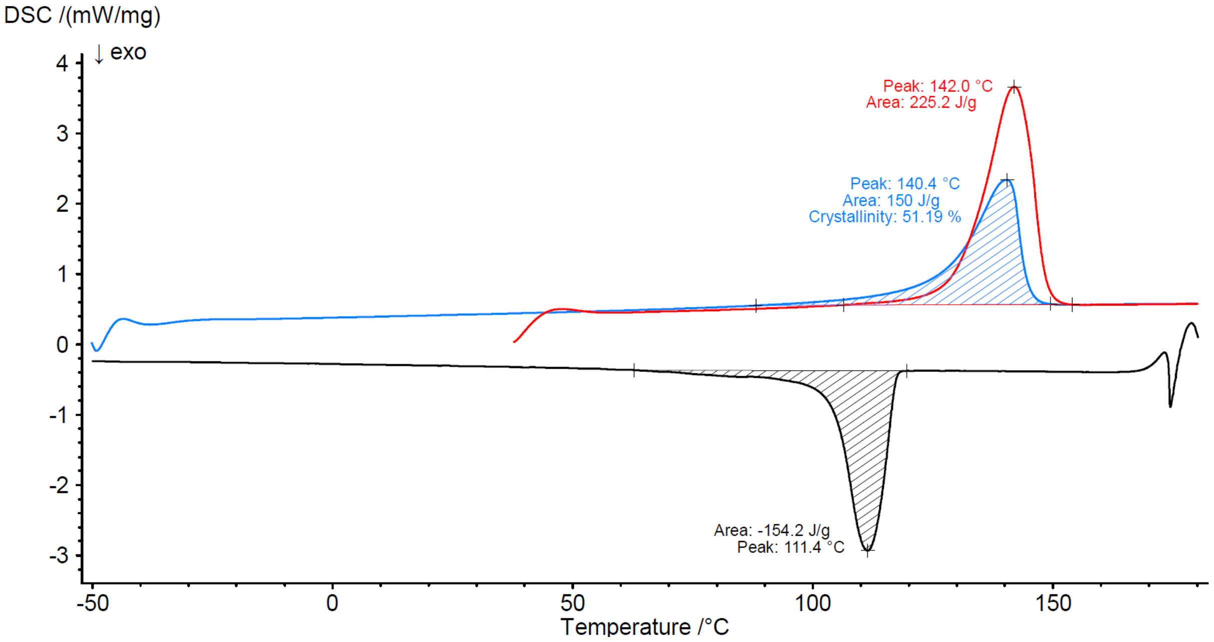

Figure S63. DSC traces of polyethylene obtained with complex **6** (Table 1, entry 16), measured with 10 K min^{-1} heating/cooling rate (red curve, 1st heating; black curve, 1st cooling; blue curve: 2nd heating).

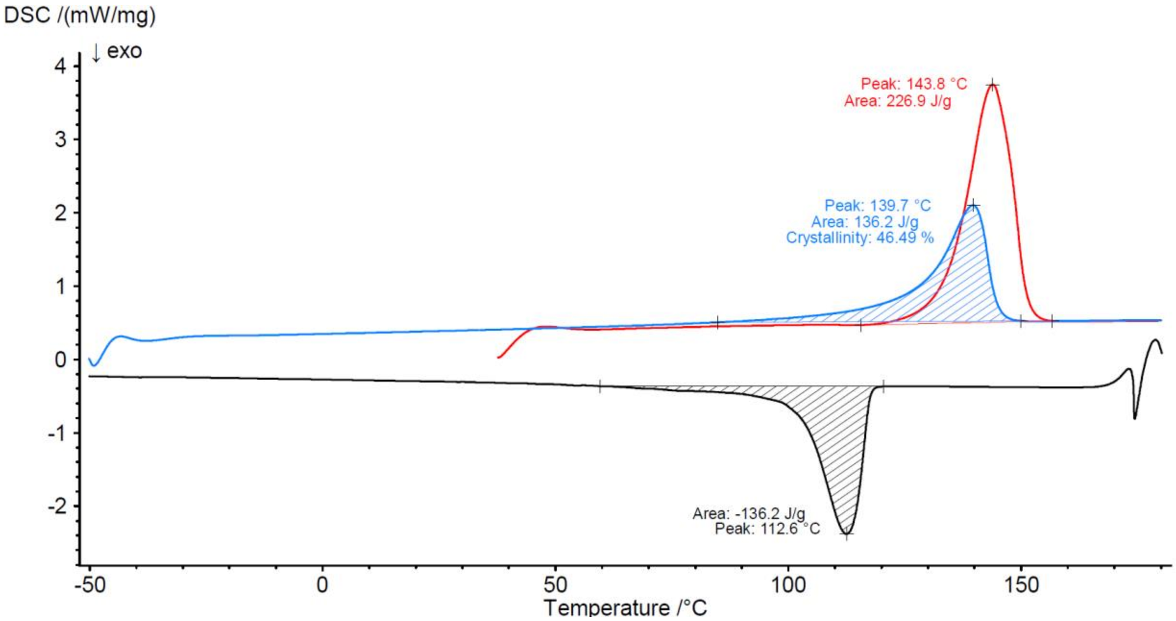

Figure S64. DSC traces of polyethylene obtained with complex **6** (Table 1, entry 17), measured with 10 K min^{-1} heating/cooling rate (red curve, 1st heating; black curve, 1st cooling; blue curve: 2nd heating).

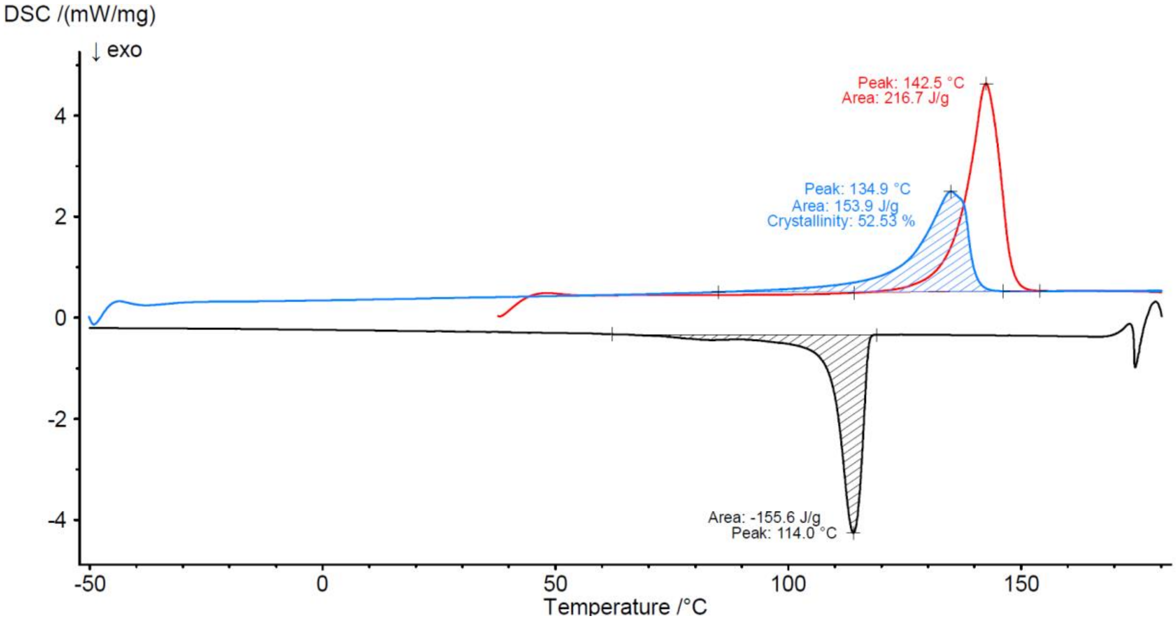

Figure S65. DSC traces of polyethylene obtained with complex **6** (Table 1, entry 18), measured with 10 K min^{-1} heating/cooling rate (red curve, 1st heating; black curve, 1st cooling; blue curve: 2nd heating).

4.3 GPC Traces

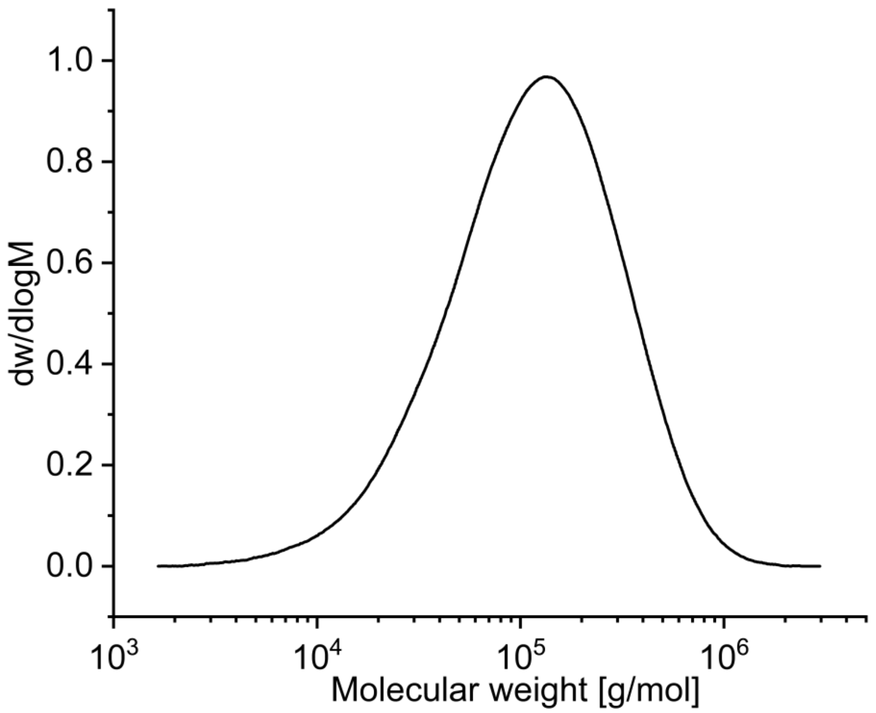

Figure S66. GPC trace of polyethylene obtained with complex **1** (Table 1, entry 1).

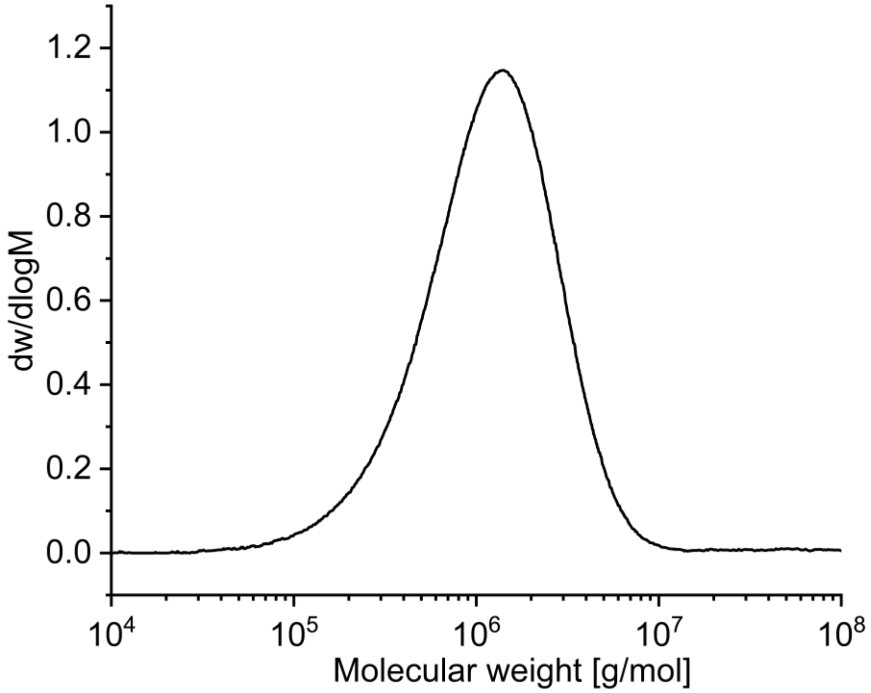

Figure S67. GPC trace of polyethylene obtained with complex **2** (Table 1, entry 4).

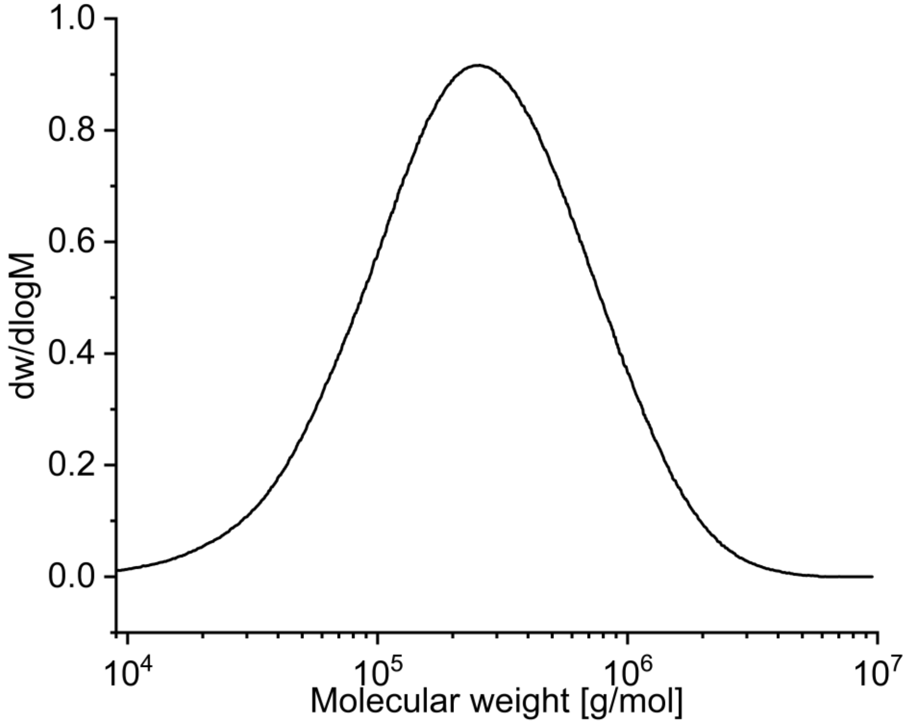

Figure S68. GPC trace of polyethylene obtained with complex **3** (Table 1, entry 9).

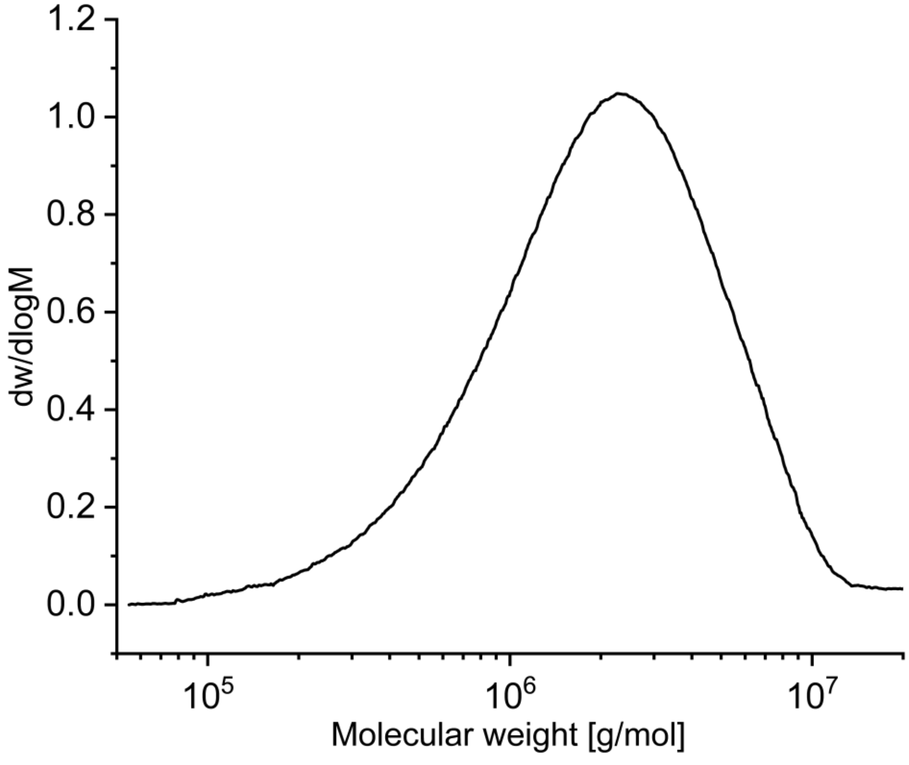

Figure S69. GPC trace of polyethylene obtained with complex **4** (Table 1, entry 10).

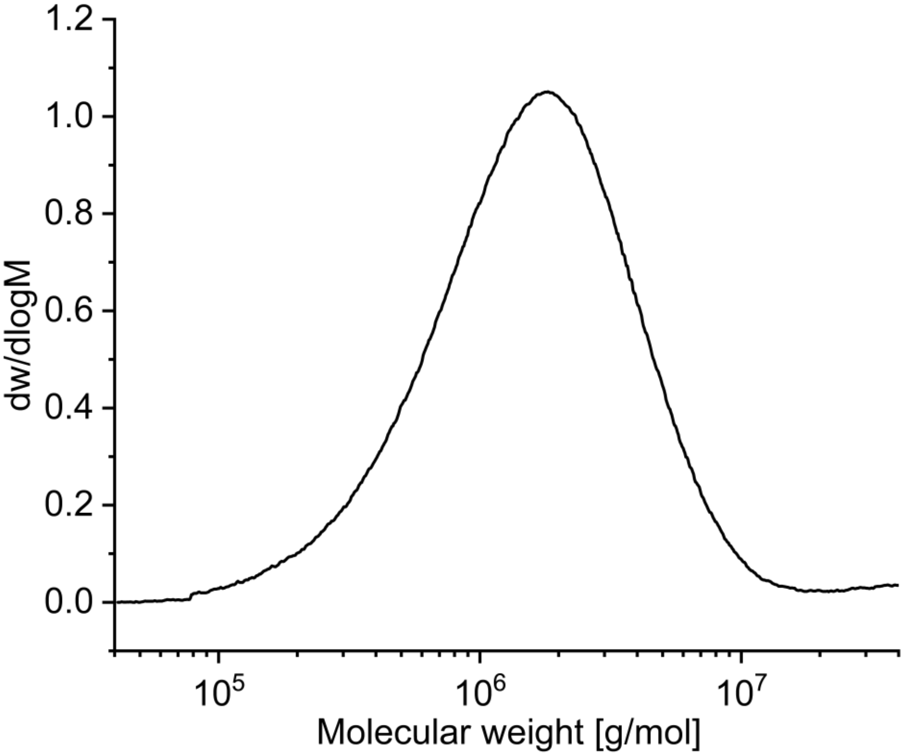

Figure S70. GPC trace of polyethylene obtained with complex **4** (Table 1, entry 11).

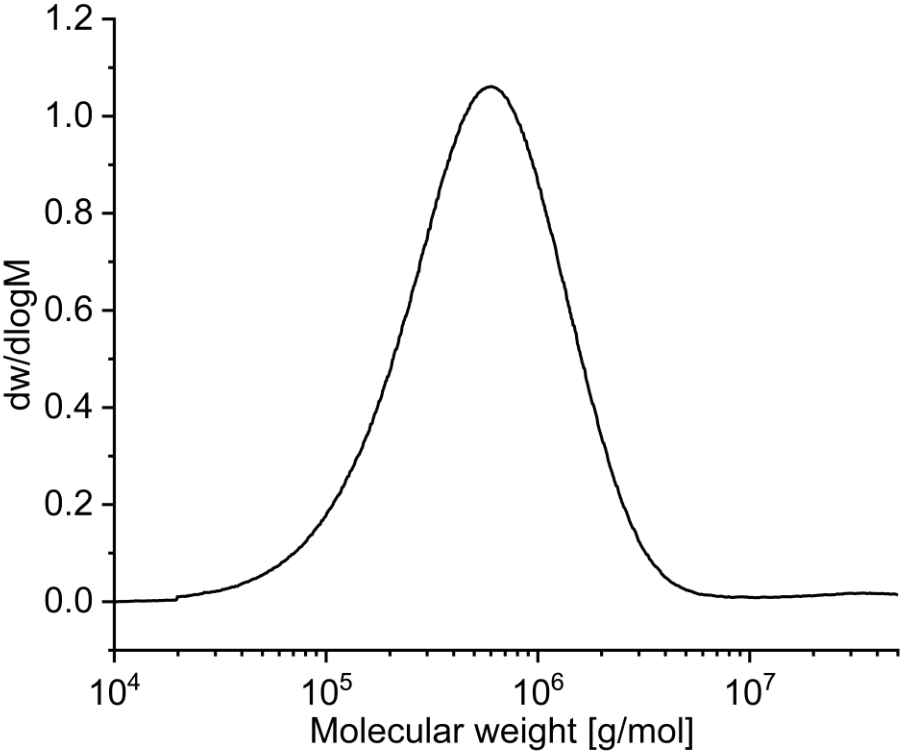

Figure S71. GPC trace of polyethylene obtained with complex **4** (Table 1, entry 12).

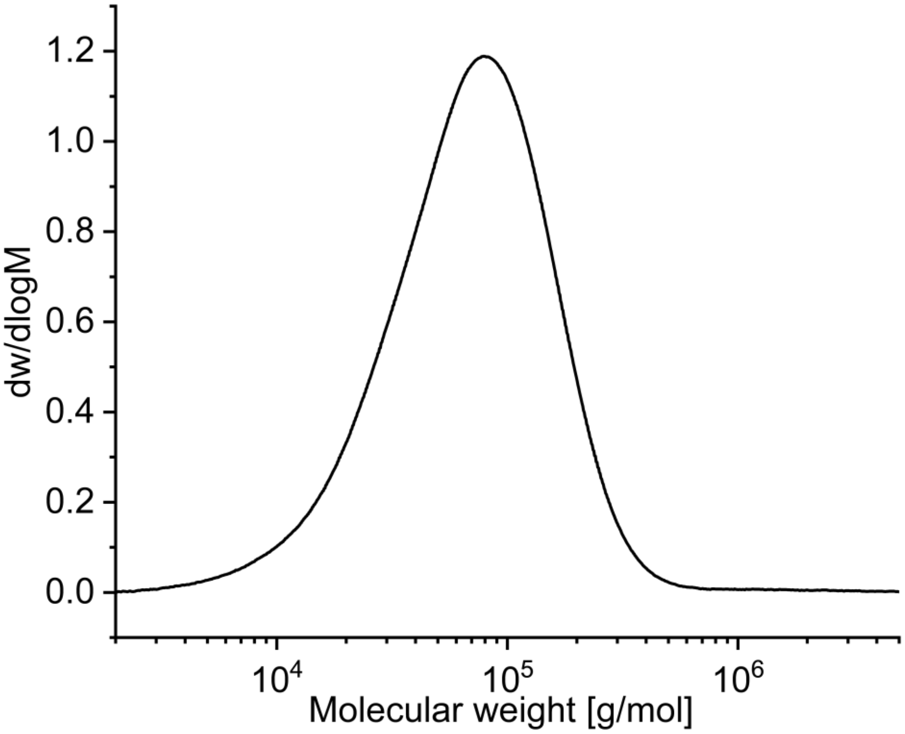

Figure S72. GPC trace of polyethylene obtained with complex **5** (Table 1, entry 13).

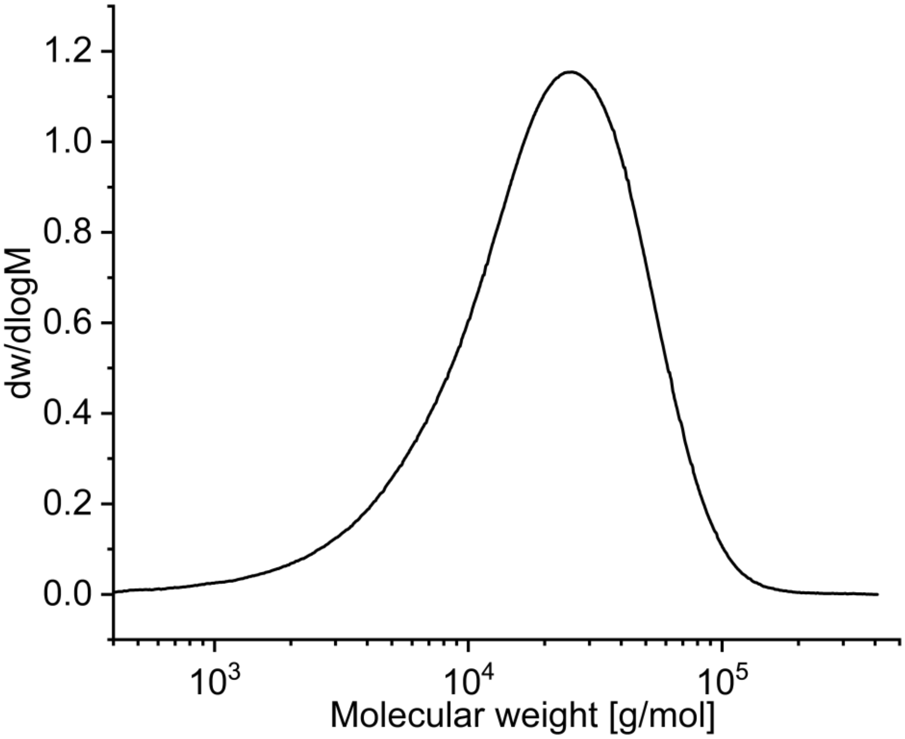

Figure S73. GPC trace of polyethylene obtained with complex **5** (Table 1, entry 14).

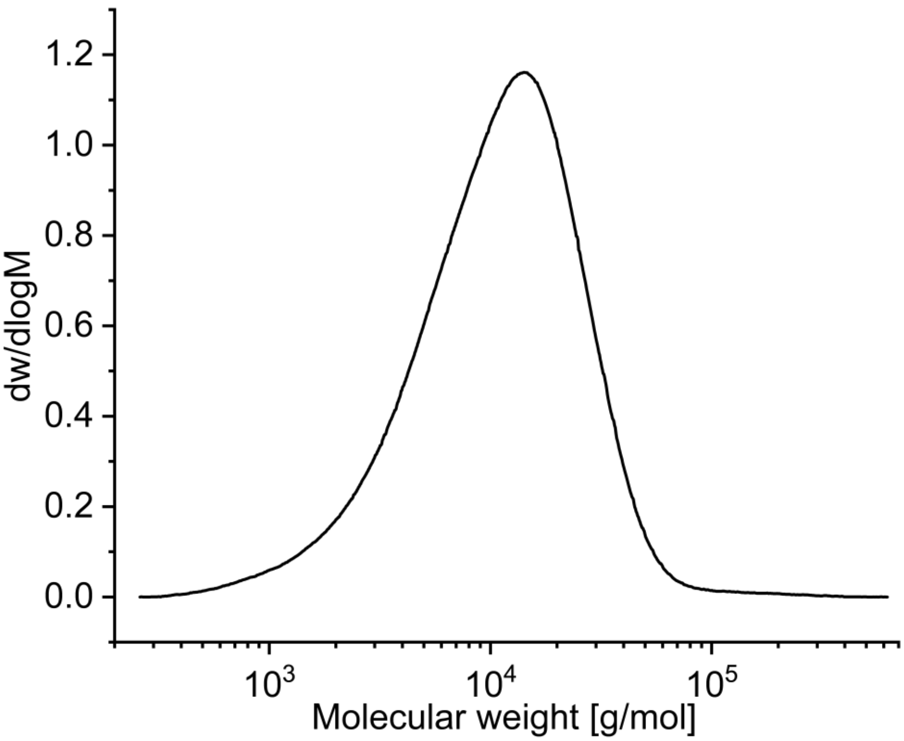

Figure S74. GPC trace of polyethylene obtained with complex **5** (Table 1, entry 15).

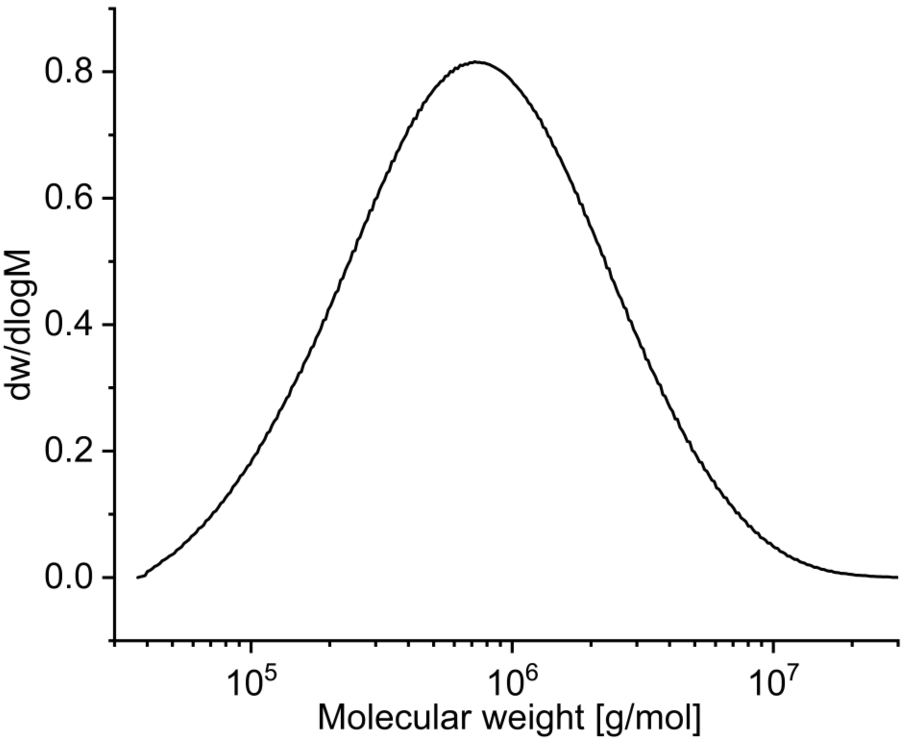

Figure S75. GPC trace of polyethylene obtained with complex **6** (Table 1, entry 18).

5 Estimation of pyridine binding equilibria and chain growth rate

5.1 Polymerizations with added pyridine

Polymerizations were performed by the general procedure outlined, with the following differences: 15 μmol catalyst precursor were dissolved in 7.5 mL of toluene. 31.6 mg (400 μmol) of pyridine were placed in a 8 mL vial, and toluene was added dropwise to form a pyridine solution (4 g in total). 1 mL of the catalyst solution was placed into a 8 mL vial, and the desired amount of pyridine solution was added (20 mg pyridine solution contained 0.158 mg, 2 μmol pyridine). The resulting solution was mixed well, and transferred into the reactor via syringe.

Table S9. Results of ethylene polymerization by complex **1** and **2** with additional pyridine.[a]

Entry	Complex	Additional pyridine (equiv.)	Yield (mg)	1/TOF (h)
1	**1**	1	60.3	1.5478E-04
2	**1**	2	36.8	2.5362E-04
3	**1**	3	25.3	3.6891E-04
4	**1**	4	17.8	5.2435E-04
5	**1**	5	14.0	6.6667E-04
6	**2**	1	717.4	1.3001E-05
7	**2**	3	258.9	3.6050E-05
8	**2**	5	147.3	6.3363E-05
9	**2**	7	116.5	8.0114E-05
10	**2**	9	91.9	1.0156E-04

[a] Polymerization conditions: 2 μmol of complex in 100 mL of toluene, 30 °C, 10 bar ethylene pressure, 10 min polymerization time, 1000 rpm.

5.2 Data analysis

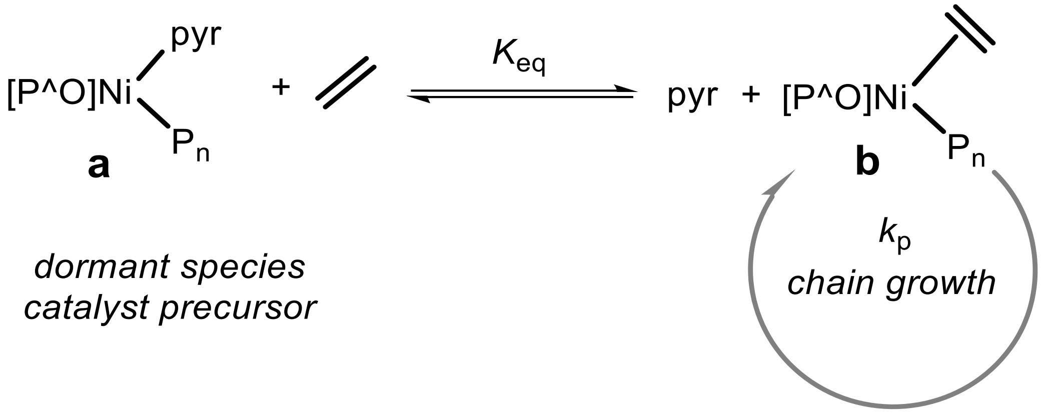

$$K_{eq} = \frac{[\mathbf{b}][pyr]}{[\mathbf{a}][\text{ethylene}]}$$

$$[\mathbf{b}] = \frac{K_{eq}\,[\mathbf{a}][\text{ethylene}]}{[pyr]}$$

$$R_p = k_p[\mathbf{b}]$$

$$R_p = K_{eq}\,k_p \frac{[\mathbf{a}][ethylene]}{[pyr]}$$

$$\text{TOF} = \frac{R_p}{[Ni]_0} = \frac{R_p}{[\boldsymbol{a}] + [\boldsymbol{b}]}$$

$$\frac{1}{\text{TOF}} = \frac{[\mathbf{a}] + [\mathbf{b}]}{R_p} = \frac{[\mathbf{a}]}{R_p} + \frac{[\mathbf{b}]}{R_p} = \frac{[\mathbf{a}]}{K_{eq}k_p[\mathbf{a}][ethylene]}[pyr] + \frac{[\mathbf{b}]}{k_p[\mathbf{b}]}$$

$$= \frac{1}{K_{eq}k_p[ethylene]}[pyr] + \frac{1}{k_p}$$

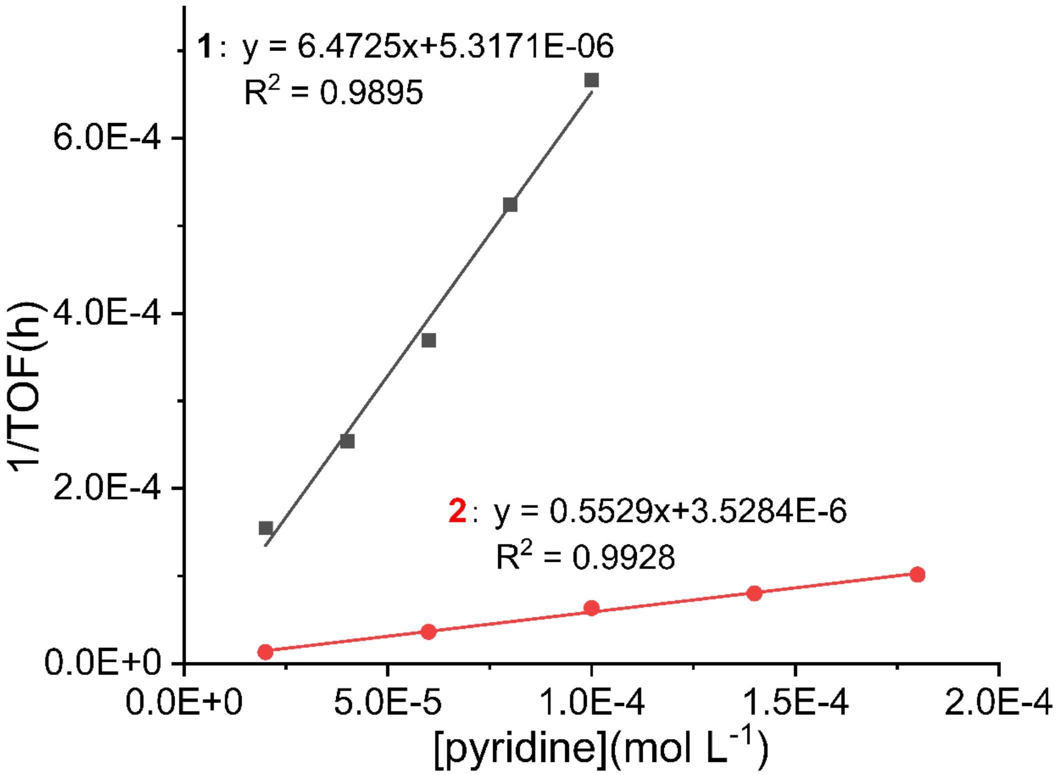

Figure S76. Plot of reciprocal polymerization activity (TOF) of complex **1** and **2** in toluene with variable amounts of added pyridine.

6 Computational Details

6.1 Selected Intermediates and Transition States

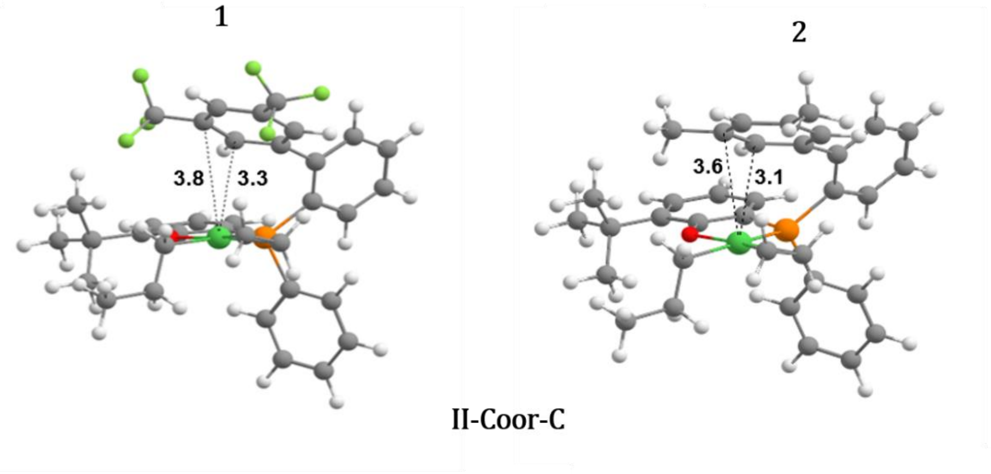

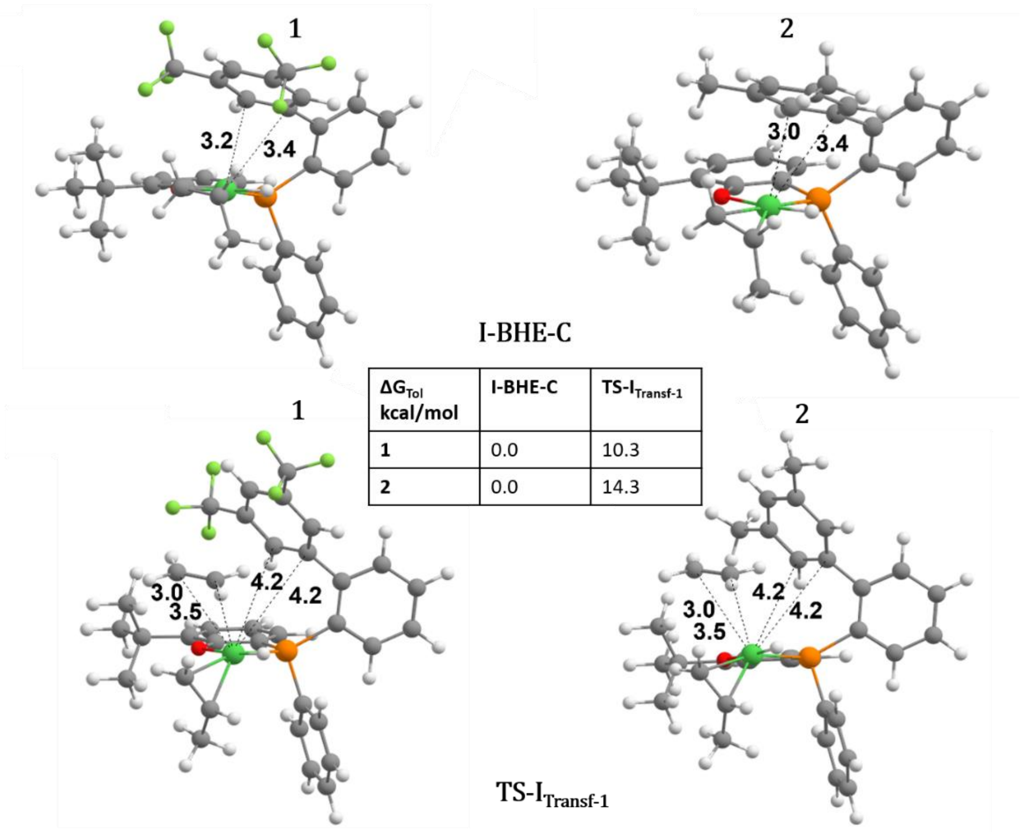

ΔG_{Tol} kcal/mol	I-BHE-C	TS-I$_{Transf-1}$
**1**	0.0	10.3
**2**	0.0	14.3

Figure S77. Optimized geometries of **II-Coor-C** for **1** and **2** (top), **I-BHE-C** for **1** and **2** (middle) and **TS-I$_{\mathbf{Transf-1}}$** for **1** and **2** (bottom) are shown, relevant distances are included.

6.2 General method

All DFT geometry optimizations were performed at the GGA BP86[19-21] level with the Gaussian09 package.[22] The electronic configuration of the systems was described with the 6-31G(d) basis set for H, C, N, F, and O while for Ni the quasi-relativistic LANL2DZ ECP effective core potential was adopted.[23] All geometries were characterized as minimum or transition state through frequency calculations. The reported Gibbs energies were built through single point energy calculations on the BP86/6-31G(d) geometries using the M06 functional and the triple-ζ TZVP[24-26] basis set on main group atoms. Solvent effects (Toluene) were included with the PCM method.[27,28] To this M06/TZVP electronic energy in solvent, thermal corrections were included from the gas-phase frequency calculations at the BP86/6- 31G(d).

6.3 Gibbs energies of competitive species for catalyst **2** with different computational protocols.

Table S10. Gibbs energies in Toluene (kcal/mol) of competitive species for catalyst **2** calculated with different computational methods. Solvent single point energy calculations (Toluene as solvent) on the BP86 optimized geometries using a) PBEO/D3 functional,[29, 30] b) B3PW91/D3[31] and a) SMD solvent model[32]

Catalyst **2**	I-β-T	TS-$I_{Transf-1}$	TS-III_{Decoor}
PBE0/D3	0.0	21.7	19.5
B3PW91/D3	0.0	20.4	17.3
M06/TZVP (SMD)	0.0	21.4	20.4

6.4 Gibbs energies of key species for catalysts **1-5**.

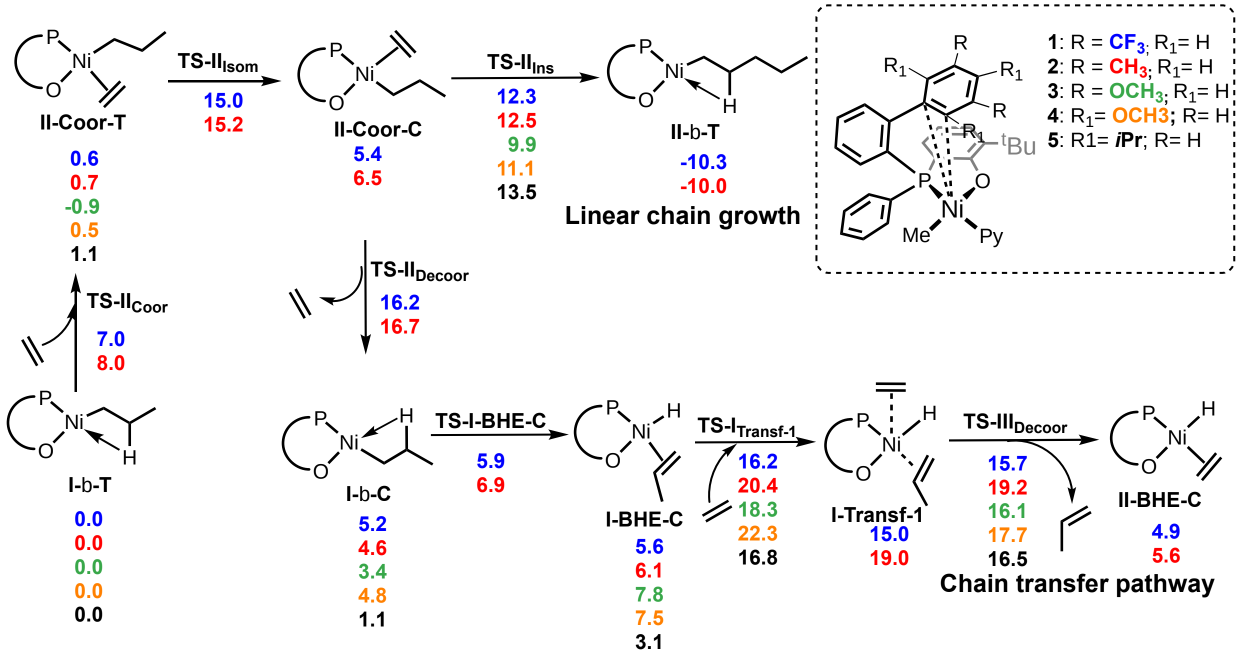

Scheme S1. Gibbs energies (ΔG_{Tol} in kcal/mol) of key species of linear chain growth and chain transfer reactions for catalysts **1-5**.

6.5 Supplementary tables

Table S11. Comparison of the computed $\Delta\Delta G^{\ddagger}$ with the experimental data.

Catalyst	$\Delta\Delta G^{‡}_{DFT}$ (kcal/mol)	M_n ($\times 10^3$ g mol^{-1})	DP_n	Chains per nickel at 50 ℃
**1**	3.9	49	1750	50
**2**	7.9	430	15357	12
**3**	8.4	329	11750	14
**4**	11.2	1235	44107	5
**5**	3.3	16	571	175

Table S12. Comparison of the $\Delta\Delta H^{‡}$ and $\Delta\Delta G^{‡}$ in Toluene for catalysts **1-5** with the M_n.

	**1**	**2**	**3**	**4**	**5**
**$\Delta\Delta H^{\neq}$ solv** (kcal/mol) **(TS-I$_{Transf-1}$)-(TS-II$_{ins}$)**	8.7	14.2	13.8	16.0	9.4
**$\Delta\Delta G^{\neq}$ solv** (kcal/mol) **(TS-I$_{Transf-1}$)-(TS-II$_{ins}$)**	3.9	7.9	8.4	11.2	3.3
**M_n** ($\times$ 10^3 g mol^{-1})	49	430	329	1235	16

The extent of the Ni-arene interaction on the reactivity of the systems was further elucidated by calculation of the $\Delta H^{‡}$ for the two key TSs, namely TS-II$_{ins}$ and TS-I$_{Transf-1}$ for catalysts **1**,**2**,**3**,**4** and **5**. Given that the breaking and the forming bonds are exactly the same for all the catalysts, and no conformational differences are present among the systems except for the extent of the Ni-arene interaction, the enthalpic contribution to the barriers can give an indication of the strength and the weight of the Ni-arene interaction on the catalyst performance.

The comparison between **1** and **2** highlights the effect of the arene on the reactivity. These two catalysts feature a very similar steric environment and the difference in the groups on the arene is essentially electronic. The $\Delta\Delta H^{‡}$ between the transfer and the insertion TS increases meaningfully moving from **1** to **2** (Table S12) demonstrating that the loss of the Ni-arene interaction in chain transfer disfavours to a greater extent the system with a more electron rich arene. As for **3**, **4** and **5**, their distal ring on the ligand varies both in sterics and electronics and both factors will affect the strength of the interaction with the metal. In any case, the trend in the calculated $\Delta\Delta H^{‡}$ between

the insertion TS and the chain transfer TS qualitatively agrees well with the computed $\Delta\Delta G^{\ddagger}$ and with the trend in the experimentally determined M_n, i.e. the more the TS-$I_{Transf-1}$ is near in energy to the TS-II_{ins} (smaller $\Delta\Delta H^{\ddagger}$ and $\Delta\Delta G^{\ddagger}$ in Table S12) the lower M_n is observed for the obtained polymer product.

Table S13. Relevant distances in Å for the intermediates I-β-T, II-Coor-T, I-β-C and I-BHE-C.

I-β-T	Ni-O	Ni-P	Ni-C_{ipso}	Ni-C_{arene1}	Ni-C_{arene2}	Ni-C_{arene3}	Ni-C_{arene4}	Ni-C_{arene5}
1	1.91	2.12	3.39	2.90	3.45	4.27	4.59	4.22
2	1.92	2.12	3.33	2.82	3.32	4.12	4.50	4.15
3	1.92	2.12	3.50	3.09	3.61	4.36	4.66	4.28
4	1.91	2.12	3.44	3.63	4.10	4.38	4.26	3.80
5	1.90	2.12	3.75	3.72	4.19	4.67	4.70	4.29

II-coord-T	Ni-O	Ni-P	Ni-C_{ipso}	Ni-C_{arene1}	Ni-C_{arene2}	Ni-C_{arene3}	Ni-C_{arene4}	Ni-C_{arene5}
1	1.98	2.20	3.66	3.29	3.78	4.50	4.76	4.40
2	1.98	2.22	3.52	3.26	3.80	4.43	4.63	4.21
3	1.99	2.23	3.60	3.37	3.89	4.50	4.66	4.26
4	1.97	2.21	3.70	3.89	4.33	4.59	4.48	4.04
5	1.96	2.23	3.83	4.12	4.66	4.95	4.71	4.18

I-β-C	Ni-O	Ni-P	Ni-C_{ipso}	Ni-C_{arene1}	Ni-C_{arene2}	Ni-C_{arene3}	Ni-C_{arene4}	Ni-C_{arene5}
1	1.87	2.22	3.43	3.10	3.57	4.20	4.40	4.08
2	1.87	2.22	3.47	2.99	3.45	4.19	4.55	4.22
3	1.88	2.22	3.51	3.03	3.49	4.26	4.60	4.28
4	1.87	2.22	3.52	3.38	3.80	4.30	4.43	4.05
5	1.87	2.22	3.63	3.69	4.10	4.46	4.40	4.02

I-BHE-C	Ni-O	Ni-P	Ni-C_{ipso}	Ni-C_{arene1}	Ni-C_{arene2}	Ni-C_{arene3}	Ni-C_{arene4}	Ni-C_{arene5}
1	1.93	2.15	3.37	3.14	3.62	4.19	4.33	3.98
2	1.94	2.15	3.43	3.05	3.55	4.26	4.56	4.18
3	1.96	2.16	3.39	3.01	3.50	4.19	4.45	4.11
4	1.94	2.16	3.38	3.67	4.11	4.27	4.04	3.58
5	1.93	2.15	3.52	3.85	4.29	4.48	4.19	3.73

6.6 Non-covalent interaction analysis.

A non-covalent interaction (NCI) analysis[33, 34] was performed on species **I-β-T** for catalysts **1** and **2** in Figure S78 and on species **TS-I$_{\mathbf{Transf\text{-}1}}$** for catalysts **1** and **2** in Figure S79.

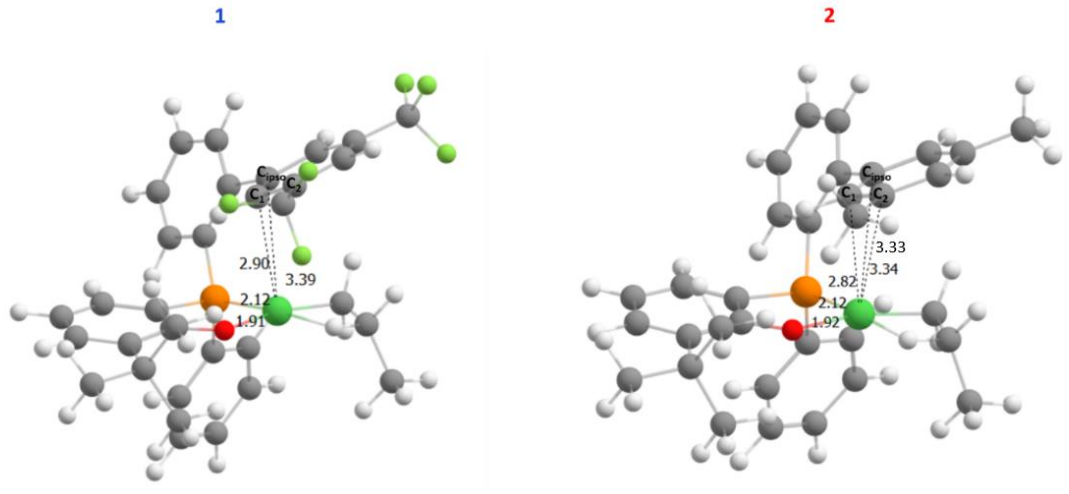

I-β-T

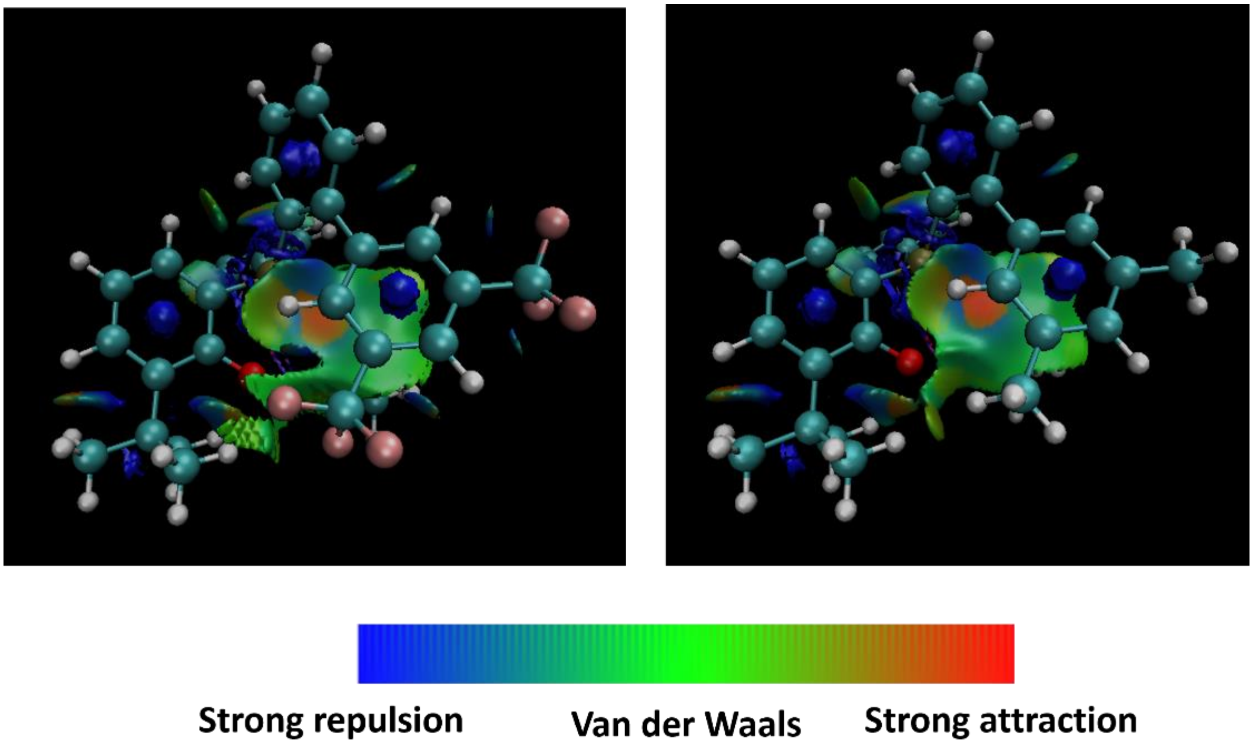

Figure S78. NCI analysis for catalysts **1** (left) and **2** (right) related to the optimized geometries of **I-β-T** species for **1** (top left) and **2** (top right). NCI surfaces correspond to s = 0.45 au and a colour scale of $-3 < \rho < 3$ au for SCF densities.

The maps clearly show that the interaction between Ni and the arene is stronger in system **2** respect to **1**, i.e. the red area corresponding to the region localized on Ni atom is more intense in the map of **2**. Instead, the weaker C-H/π interactions between the aryl ring and the chain give rise to the green portion of the NCI map.

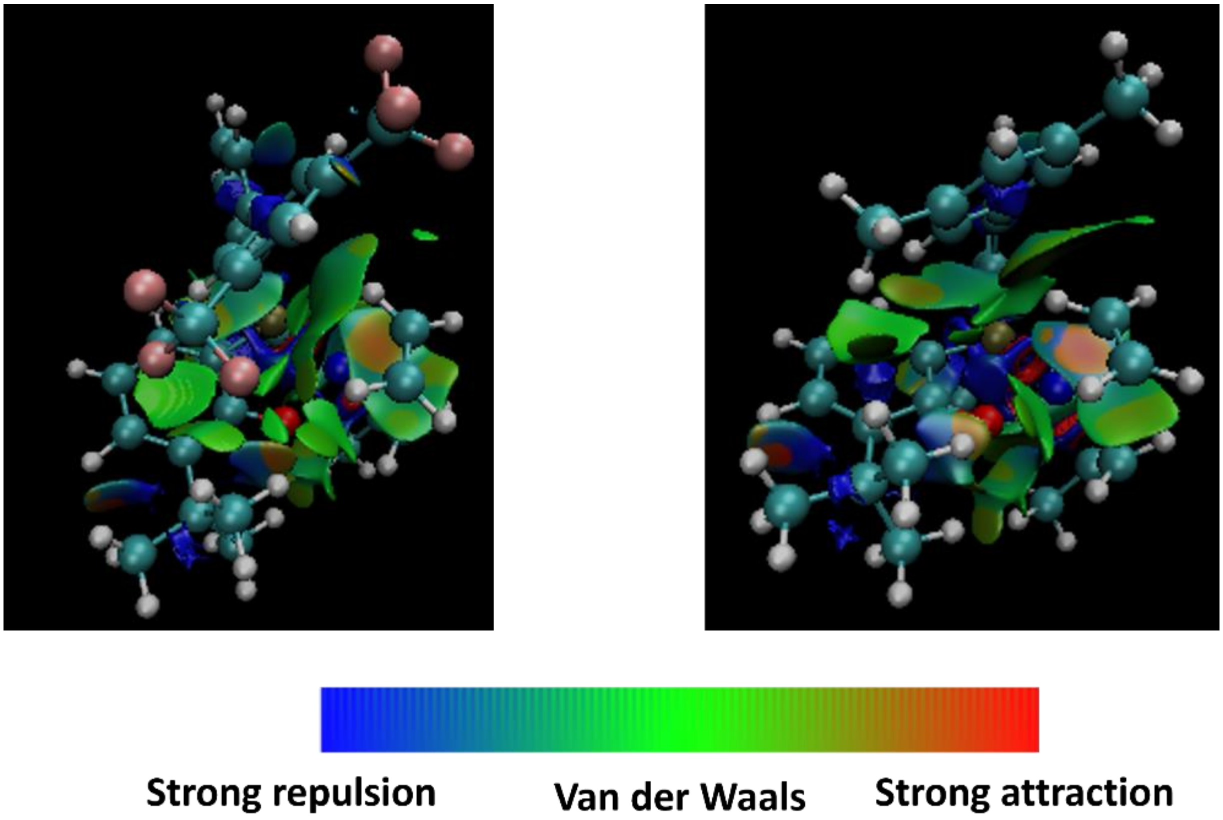

Figure S79. NCI analysis for **TS-I**$_{\mathbf{Transf\text{-}1}}$ for systems **1** and **2**. NCI surfaces correspond to s = 0.45 au and a colour scale of $-3 < \rho < 3$ au for SCF densities.

From the NCI map for **TS-I**$_{\mathbf{Tranf\text{-}1}}$ it emerges that the C-H/π interactions between the monomer and the biaryl (green areas) are weaker than the interaction occurring with the metal, i.e. between ethylene and Ni in this case (red area).

Table S14. NBO analysis on I-β-T for **1** and **2**.

	Charge Transfer (kcal/mol)<br>σC-H→LP*Ni	Mayer atomic Bond order
**1**	3.50	0.100991
**2**	2.86	0.110994

6.7 Cartesian Coordinates

[1]I-β-T

Zero-point correction= 0.547565 (Hartree/Particle)

Thermal correction to Energy= 0.589268

Thermal correction to Enthalpy= 0.590212

Thermal correction to Gibbs Free Energy= 0.469967

Sum of electronic and zero-point Energies= -2460.713408

Sum of electronic and thermal Energies= -2460.671705

Sum of electronic and thermal Enthalpies= -2460.670761

Sum of electronic and thermal Free Energies=-2460.791007

E solvent= -2460.64360792

C	1.978582	2.652942	0.766476
C	3.376821	2.576188	0.936566
H	3.930383	1.739746	0.496535
C	4.054816	3.571263	1.660397
H	5.141764	3.503281	1.784088
C	3.347983	4.647905	2.218801
H	3.880085	5.422621	2.782373
C	1.954148	4.729204	2.052869
H	1.396201	5.567795	2.485279
C	1.271105	3.736697	1.335390
C	0.061712	2.187424	-1.367999
C	0.650053	3.321827	-1.974918
H	1.634212	3.659064	-1.632501
C	-0.005635	4.037638	-2.985248
H	0.479937	4.907210	-3.441861
C	-1.284141	3.634898	-3.398596
H	-1.811072	4.181003	-4.188898
C	-1.888728	2.525352	-2.796242
H	-2.882558	2.203311	-3.127509
C	-1.239549	1.778874	-1.783307
C	-1.992862	0.633821	-1.195183
C	-3.325522	0.843298	-0.782525
C	-4.096857	-0.210130	-0.266010
C	-3.555957	-1.502183	-0.173662
C	-2.234082	-1.722680	-0.585491
C	-1.439240	-0.662292	-1.068478
C	2.273282	0.253431	-0.948148
C	3.079576	0.651697	-2.035160
H	2.951605	1.643581	-2.485564
C	4.035389	-0.235681	-2.528456
H	4.670849	0.041825	-3.376423
C	4.188654	-1.498640	-1.912625
H	4.964541	-2.162174	-2.307899
C	3.407806	-1.941448	-0.834190
C	2.375752	-1.049244	-0.352038
Ni	0.246420	-0.146118	1.231674
O	1.541151	-1.404421	0.618191
P	1.058935	1.297763	-0.085891
H	0.184297	3.809833	1.210144

C	-1.030489	0.821706	2.261879
C	-1.020923	-0.464246	3.061767
H	-0.613572	1.699360	2.782729
H	-1.968708	1.055797	1.736893
H	-2.009052	-0.960617	3.049834
H	-0.433833	-0.872695	-1.445590
H	-3.761177	1.844560	-0.855884
C	3.641728	-3.315566	-0.167973
C	2.372694	-4.198795	-0.299816
H	2.544304	-5.178417	0.186246
H	1.505844	-3.717082	0.174735
H	2.130749	-4.384029	-1.362325
C	3.972797	-3.106389	1.335959
H	4.112471	-4.085176	1.833165
H	4.907192	-2.526977	1.455246
H	3.156263	-2.567408	1.842312
C	4.822335	-4.081587	-0.809662
H	4.639562	-4.311607	-1.875703
H	5.773575	-3.522893	-0.732781
H	4.959387	-5.043727	-0.283255
H	-0.379566	-1.273517	2.521436
C	-0.439298	-0.369818	4.483505
H	-0.326560	-1.366710	4.945265
H	0.552588	0.114859	4.467905
H	-1.105569	0.234249	5.124550
C	-5.492142	0.056867	0.249120
F	-6.310823	-1.017952	0.074081
F	-5.481994	0.335105	1.587374
F	-6.069264	1.123174	-0.373738
C	-1.650757	-3.113552	-0.472614
F	-2.616310	-4.072306	-0.571299
F	-0.727058	-3.356428	-1.437222
F	-1.037375	-3.299927	0.738056
H	-4.166006	-2.332479	0.191796

[1]TS-II$_{Coor}$

Zero-point correction= 0.599041 (Hartree/Particle)

Thermal correction to Energy= 0.644449

Thermal correction to Enthalpy= 0.645393

Thermal correction to Gibbs Free Energy= 0.517583

Sum of electronic and zero-point Energies= -2539.241145

Sum of electronic and thermal Energies= -2539.195737

Sum of electronic and thermal Enthalpies= -2539.194793

Sum of electronic and thermal Free Energies= -2539.322603

E solvent= -2539.19656325

C	0.686219	2.725150	0.442334
C	1.972076	3.180567	0.084905
H	2.566965	2.608887	-0.635501
C	2.486018	4.361838	0.647879
H	3.486975	4.705651	0.362337
C	1.725000	5.097561	1.569168

H	2.128737	6.017412	2.007255	C	1.922104	-0.705765	-0.738936
C	0.442659	4.647246	1.932258	Ni	-0.031249	-0.370537	1.266522
H	-0.156024	5.215054	2.654043	O	1.506894	-1.187627	0.427098
C	-0.072078	3.466694	1.377935	P	0.002217	1.134517	-0.214307
C	-1.439488	1.689547	-1.241889	H	-1.068082	3.115854	1.673495
C	-1.375663	2.996643	-1.781179	C	-1.584600	0.060462	2.282000
H	-0.508704	3.626234	-1.555827	C	-1.466680	-1.373910	2.764837
C	-2.402079	3.515666	-2.581279	H	-1.358060	0.824796	3.046719
H	-2.317277	4.529402	-2.988567	H	-2.499480	0.293619	1.718798
C	-3.531946	2.728957	-2.846453	H	-2.178970	-2.025568	2.229745
H	-4.346346	3.115723	-3.469631	H	-0.836127	-1.265641	-1.635992
C	-3.611892	1.435174	-2.318784	H	-4.804329	-0.099838	-0.346138
H	-4.482086	0.809700	-2.547020	C	3.872949	-2.429352	-0.832710
C	-2.582460	0.879432	-1.519430	C	2.973446	-3.665023	-0.563814
C	-2.786693	-0.517188	-1.035148	H	3.571479	-4.481100	-0.114604
C	-4.038602	-0.874389	-0.479306	H	2.158567	-3.403443	0.129278
C	-4.313339	-2.191285	-0.073350	H	2.532942	-4.043570	-1.504590
C	-3.305553	-3.166207	-0.234686	C	4.493865	-1.947683	0.507046
C	-2.051344	-2.847749	-0.785004	H	5.085873	-2.761413	0.968185
C	-1.796783	-1.512433	-1.169474	H	5.167194	-1.086504	0.341715
C	1.255842	0.423841	-1.328040	H	3.703696	-1.646110	1.213006
C	1.598283	0.922604	-2.601687	C	5.029838	-2.880752	-1.754856
H	1.040599	1.764207	-3.031329	H	4.662726	-3.274288	-2.720746
C	2.645828	0.325203	-3.304869	H	5.742409	-2.061169	-1.962426
H	2.927793	0.684456	-4.300549	H	5.593179	-3.692399	-1.259354
C	3.349706	-0.747032	-2.713433	H	-0.438782	-1.804217	2.464700
H	4.180726	-1.179549	-3.279605	C	-1.544547	-1.580608	4.290579
C	3.042025	-1.281942	-1.452233	H	-1.375051	-2.637105	4.567477

H	-0.786922	-0.964443	4.805271
H	-2.541411	-1.281998	4.660650
H	-3.506980	-4.201824	0.071521
C	-0.990581	-3.910488	-0.979188
H	-0.839291	-4.134195	-2.052606
H	-0.018242	-3.573575	-0.579704
H	-1.266095	-4.854494	-0.477501
C	-5.665473	-2.563404	0.503422
H	-6.211461	-1.673963	0.863103
H	-6.303434	-3.058050	-0.254762
H	-5.566319	-3.267483	1.349216
C	1.864842	-0.244839	4.263201
C	1.881531	0.549625	3.174847
H	2.314413	-1.245099	4.251563
H	1.409281	0.073870	5.208977
H	2.370416	0.233104	2.247179
H	1.457494	1.560399	3.189451

[1]II-Coor-T

Zero-point correction= 0.601433 (Hartree/Particle)

Thermal correction to Energy= 0.646459

Thermal correction to Enthalpy= 0.647403

Thermal correction to Gibbs Free Energy= 0.520321

Sum of electronic and zero-point Energies= -2539.260031

Sum of electronic and thermal Energies= -2539.215005

Sum of electronic and thermal Enthalpies= -2539.214061

Sum of electronic and thermal Free Energies= -2539.341143

E solvent= -2.538.689237

C	-1.957239	2.694217	-0.284409
C	-3.364818	2.700314	-0.192268
H	-3.878457	1.868831	0.302087
C	-4.102621	3.770411	-0.726361
H	-5.195888	3.765009	-0.647830
C	-3.446352	4.841910	-1.351703
H	-4.024656	5.675468	-1.766153
C	-2.043175	4.842544	-1.445003
H	-1.524085	5.676384	-1.931566
C	-1.302058	3.774035	-0.920878
C	0.083408	2.005114	1.670729
C	-0.412583	3.143300	2.348404
H	-1.376535	3.566980	2.047557
C	0.307352	3.753743	3.384728
H	-0.108858	4.628930	3.895846
C	1.558584	3.239849	3.754424
H	2.135336	3.704669	4.561735
C	2.069423	2.119154	3.088772
H	3.038578	1.704369	3.388763
C	1.353206	1.477148	2.050825
C	1.998176	0.295716	1.406529
C	3.342790	0.393379	0.990416
C	3.998438	-0.702812	0.408678
C	3.330781	-1.926348	0.252825

C	1.995744	-2.034515	0.671791	H	-4.655940	-3.399398	-2.231116
C	1.323958	-0.933166	1.230963	H	-5.256581	-1.924724	-1.414632
C	-2.120360	0.110371	1.124845	H	-3.584037	-1.972337	-2.057710
C	-2.735972	0.287244	2.381235	C	-5.001801	-3.952102	0.422189
H	-2.471523	1.146812	3.009316	H	-4.701580	-4.421565	1.377123
C	-3.680261	-0.647386	2.805574	H	-5.907663	-3.344046	0.602185
H	-4.170959	-0.544736	3.779439	H	-5.280792	-4.765833	-0.271839
C	-4.012260	-1.726544	1.955958	H	0.570995	2.657109	-3.261292
H	-4.773457	-2.429883	2.308055	C	2.744373	2.716664	-3.212517
C	-3.425842	-1.943457	0.698952	H	2.853703	3.221281	-4.190678
C	-2.403062	-1.010306	0.273974	H	2.810442	3.489902	-2.424247
Ni	-0.323421	0.102252	-1.421837	H	3.605166	2.035720	-3.081129
O	-1.733691	-1.162743	-0.861267	C	5.403458	-0.535201	-0.123806
P	-0.965851	1.262790	0.335711	F	6.079075	-1.716910	-0.158099
H	-0.209114	3.780441	-1.000888	F	5.392549	-0.042433	-1.398657
C	1.261492	1.208916	-1.788500	F	6.130294	0.336276	0.631621
C	1.408867	1.944320	-3.123129	C	1.282439	-3.355772	0.486206
H	1.440771	1.920836	-0.960465	F	2.106020	-4.406514	0.770338
H	2.018547	0.400740	-1.688709	F	0.186730	-3.463421	1.279128
H	1.356736	1.236359	-3.972808	F	0.874252	-3.521655	-0.809343
H	0.294335	-1.051406	1.583372	H	3.849140	-2.788519	-0.175667
H	3.875101	1.343246	1.101331	C	-0.041854	-1.098505	-3.153160
C	-3.856915	-3.121143	-0.204195	C	-1.020773	-0.145502	-3.389830
C	-2.657587	-4.075277	-0.447374	H	0.984386	-0.965260	-3.513104
H	-2.955686	-4.890001	-1.134726	H	-0.308969	-2.087218	-2.767101
H	-1.805541	-3.535951	-0.886363	H	-0.807834	0.773722	-3.945096
H	-2.322200	-4.533190	0.500491	H	-2.073692	-0.378107	-3.199163
C	-4.368022	-2.566256	-1.562212				

[1]TS-II$_{Isom}$

Zero-point correction= 0.600518 (Hartree/Particle)

Thermal correction to Energy= 0.644941

Thermal correction to Enthalpy= 0.645885

Thermal correction to Gibbs Free Energy= 0.520502

Sum of electronic and zero-point Energies= -2539.245513

Sum of electronic and thermal Energies= -2539.201090

Sum of electronic and thermal Enthalpies= -2539.200146

Sum of electronic and thermal Free Energies= -2539.325529

E solvent= -2539.18673195

C	-2.124407	-2.495360	0.044494
C	-3.501809	-2.336492	-0.215400
H	-3.850883	-1.455141	-0.764237
C	-4.422351	-3.308115	0.216030
H	-5.489674	-3.171818	0.005862
C	-3.980490	-4.448525	0.904162
H	-4.699894	-5.205136	1.237227
C	-2.608543	-4.615061	1.166468
H	-2.254928	-5.502740	1.703909
C	-1.688519	-3.643221	0.748036
C	0.175273	-2.112700	-1.657563
C	-0.393438	-3.168216	-2.407600
H	-1.445853	-3.427541	-2.249935
C	0.361507	-3.905690	-3.330323
H	-0.111988	-4.712487	-3.901097
C	1.719641	-3.606585	-3.513085
H	2.323721	-4.172894	-4.230570
C	2.303568	-2.572094	-2.772065
H	3.360233	-2.323672	-2.924778
C	1.555648	-1.808868	-1.844280
C	2.282545	-0.736109	-1.104181
C	3.512214	-1.044636	-0.483809
C	4.261117	-0.052071	0.169905
C	3.803629	1.272552	0.203082
C	2.581541	1.589581	-0.412527
C	1.817527	0.598277	-1.053063
C	-1.786712	0.050762	-1.379004
C	-2.281722	-0.145504	-2.685397
H	-2.088723	-1.091014	-3.206917
C	-3.007846	0.869671	-3.309250
H	-3.393334	0.736609	-4.326045
C	-3.251797	2.070888	-2.611373
H	-3.840281	2.842108	-3.117734
C	-2.785950	2.320686	-1.310744
C	-1.997606	1.286412	-0.674126
Ni	-0.463644	0.176491	1.490267
O	-1.472877	1.491410	0.531602
P	-0.874097	-1.206835	-0.417331
H	-0.621422	-3.778747	0.963531
C	-2.006321	0.076831	2.712931
H	-2.088263	1.084075	3.153855
C	-1.964804	-1.049521	3.730348

H	-1.750833	-2.011483	3.228561
H	-2.795564	-0.068773	1.956231
H	-1.167954	-0.886009	4.480504
H	0.885355	0.874133	-1.556932
H	3.875825	-2.077324	-0.493025
C	-3.325723	-1.162379	4.462671
H	-4.142907	-1.375438	3.750775
H	-3.298262	-1.981043	5.205223
H	-3.573411	-0.226224	4.995055
H	4.392900	2.049133	0.697770
C	-3.108308	3.643977	-0.578138
C	-1.798814	4.404784	-0.238388
H	-2.037582	5.336794	0.309197
H	-1.133123	3.789947	0.384934
H	-1.255311	4.680765	-1.159880
C	-3.885161	3.336143	0.732138
H	-4.109631	4.277521	1.269003
H	-4.844856	2.832801	0.511340
H	-3.289711	2.687665	1.392948
C	-3.989474	4.585442	-1.433430
H	-3.487008	4.890343	-2.369975
H	-4.962053	4.127543	-1.692936
H	-4.199739	5.504954	-0.857482
C	0.730444	0.661099	3.019421
C	1.031999	-0.647338	2.574941
H	1.283207	1.523823	2.624996
H	0.195959	0.838764	3.958946
H	1.861297	-0.818746	1.877671
H	0.714519	-1.524137	3.150306
C	2.094664	3.021706	-0.359188
F	1.192275	3.291518	-1.334961
F	1.500131	3.302370	0.842626
F	3.129159	3.900929	-0.493260
C	5.594346	-0.415221	0.786239
F	5.977539	0.480585	1.736509
F	5.554882	-1.647274	1.369442
F	6.581731	-0.453436	-0.155271

[1]II-Coor-C

Zero-point correction= 0.602382 (Hartree/Particle)

Thermal correction to Energy= 0.647173

Thermal correction to Enthalpy= 0.648118

Thermal correction to Gibbs Free Energy= 0.521877

Sum of electronic and zero-point Energies= -2539.247344

Sum of electronic and thermal Energies= -2539.202553

Sum of electronic and thermal Enthalpies= -2539.201609

Sum of electronic and thermal Free Energies= -2539.327849

E solvent= -2539.19936000

C	2.141483	2.636978	0.663200
C	3.511309	2.307021	0.748174

H	3.880028	1.399089	0.258660
C	4.400210	3.140105	1.446787
H	5.461501	2.871731	1.501056
C	3.936681	4.311324	2.066806
H	4.632747	4.960103	2.610350
C	2.575843	4.650980	1.982322
H	2.206348	5.568191	2.455375
C	1.682694	3.819588	1.289327
C	-0.080692	2.521256	-1.225541
C	0.477506	3.713501	-1.742290
H	1.506579	3.978772	-1.479197
C	-0.263055	4.576267	-2.562764
H	0.200908	5.489539	-2.951655
C	-1.595218	4.266453	-2.872252
H	-2.189421	4.933816	-3.506249
C	-2.163664	3.087279	-2.374021
H	-3.196187	2.827058	-2.634245
C	-1.426327	2.193323	-1.563536
C	-2.119366	0.946960	-1.118104
C	-3.361418	1.041047	-0.456869
C	-4.060557	-0.117443	-0.078887
C	-3.552543	-1.385980	-0.393489
C	-2.323221	-1.485341	-1.063637
C	-1.596165	-0.333563	-1.404797
C	1.961587	0.392731	-1.209170
C	2.600443	0.797022	-2.399451
H	2.441181	1.806842	-2.796925
C	3.427879	-0.110250	-3.059217
H	3.929043	0.165164	-3.993356
C	3.626247	-1.392671	-2.500308
H	4.299482	-2.074686	-3.028732
C	3.016486	-1.836880	-1.316515
C	2.112343	-0.917435	-0.655088
Ni	0.201874	-0.076800	1.366097
O	1.444471	-1.261782	0.441561
P	0.963241	1.445269	-0.128346
H	0.627416	4.107424	1.217262
C	0.043947	-1.705028	2.588112
C	1.178012	-1.623904	3.605419
H	-0.923090	-1.933233	3.065965
H	0.239598	-2.439052	1.791629
H	2.120197	-1.351686	3.094292
H	-0.645990	-0.429981	-1.938793
H	-3.778426	2.025740	-0.222562
C	3.309690	-3.238702	-0.734865
C	2.010217	-4.084904	-0.698612
H	2.216274	-5.073882	-0.246433
H	1.226823	-3.585609	-0.110507
H	1.620937	-4.250343	-1.719122
C	3.880709	-3.090931	0.701928
H	4.073481	-4.089095	1.138843
H	4.836990	-2.535263	0.688846
H	3.171263	-2.553423	1.349958
C	4.351817	-4.016082	-1.573583
H	3.999255	-4.207490	-2.603921
H	5.321485	-3.487486	-1.629345

H	4.535749	-4.997832	-1.100520
H	0.980842	-0.825738	4.350739
C	1.378856	-2.963261	4.353889
H	2.199402	-2.888095	5.091810
H	0.461088	-3.259538	4.895162
H	1.630050	-3.774778	3.647488
C	-5.321714	-0.003960	0.746010
F	-6.206644	-0.998899	0.463113
F	-5.038441	-0.091251	2.082706
F	-5.956763	1.185009	0.550416
C	-1.798982	-2.864340	-1.399659
F	-2.768896	-3.623979	-1.990206
F	-0.738332	-2.820858	-2.245255
F	-1.411551	-3.534259	-0.275349
H	-4.113469	-2.286939	-0.128337
C	-1.283070	0.395023	2.731594
C	-0.819322	1.556904	2.100769
H	-2.219535	-0.073302	2.407233
H	-0.972483	0.179866	3.757865
H	-1.407822	2.022506	1.302587
H	-0.095321	2.206866	2.607839

[1]TS-II$_{Ins}$

Zero-point correction= 0.602738 (Hartree/Particle)

Thermal correction to Energy= 0.646658

Thermal correction to Enthalpy= 0.647602

Thermal correction to Gibbs Free Energy= 0.523623

Sum of electronic and zero-point Energies= -2539.242568

Sum of electronic and thermal Energies= -2539.198648

Sum of electronic and thermal Enthalpies= -2539.197704

Sum of electronic and thermal Free Energies= -2539.321683

E solvent= -2539.1938199

C	2.030211	2.709813	0.646999
C	3.424437	2.491301	0.646533
H	3.836984	1.633236	0.104531
C	4.279697	3.370579	1.330662
H	5.360746	3.189977	1.319986
C	3.756577	4.476554	2.019229
H	4.426210	5.161780	2.551211
C	2.369594	4.703809	2.020143
H	1.953614	5.568722	2.549791
C	1.510273	3.825464	1.343103
C	-0.240076	2.485394	-1.173132
C	0.255565	3.704241	-1.692796
H	1.273611	4.016746	-1.439006
C	-0.529677	4.534116	-2.504826
H	-0.111574	5.468756	-2.894911
C	-1.846518	4.159970	-2.807134
H	-2.475537	4.795541	-3.440237
C	-2.354941	2.957389	-2.301897
H	-3.375389	2.650392	-2.558117

C	-1.575276	2.097458	-1.491735	H	-1.663639	1.558631	1.547442
C	-2.226936	0.836648	-1.029010	H	-0.711610	0.024421	4.016440
C	-3.515912	0.901340	-0.458612	H	-1.950995	-0.550991	2.790369
C	-4.196832	-0.267777	-0.081454	H	1.417114	-0.703561	4.361965
C	-3.613195	-1.525255	-0.293268	H	-0.646520	-0.511709	-1.683318
C	-2.331925	-1.598137	-0.861790	H	-3.989158	1.875994	-0.301907
C	-1.628800	-0.430931	-1.207899	C	2.069939	-2.765456	4.134118
C	1.921882	0.477318	-1.266787	H	2.280518	-3.501113	3.337627
C	2.478460	0.932020	-2.479687	H	3.001532	-2.613098	4.709549
H	2.243853	1.936719	-2.852360	H	1.319501	-3.208776	4.815180
C	3.321931	0.082078	-3.193475	H	-4.157608	-2.437980	-0.035887
H	3.762984	0.398806	-4.144713	C	-5.537123	-0.162390	0.609562
C	3.615314	-1.196120	-2.667639	F	-5.386436	-0.025390	1.961013
H	4.298396	-1.831366	-3.240183	F	-6.245027	0.920151	0.178426
C	3.086648	-1.691741	-1.465266	F	-6.304734	-1.267940	0.403434
C	2.167961	-0.835722	-0.742339	C	-1.709005	-2.960122	-1.075164
Ni	0.324038	-0.035838	1.327787	F	-2.640635	-3.871948	-1.480705
O	1.565786	-1.223062	0.375703	F	-0.725036	-2.931974	-2.009102
P	0.905777	1.460852	-0.132936	F	-1.167441	-3.447938	0.082536
H	0.431502	4.020562	1.342098	C	3.482257	-3.085055	-0.925034
C	0.261259	-1.621804	2.763442	C	2.233213	-4.000997	-0.840157
H	-0.508932	-2.099135	3.388754	H	2.513870	-4.985475	-0.418682
C	1.563990	-1.432672	3.539005	H	1.455619	-3.554427	-0.203883
H	2.339322	-1.010430	2.874193	H	1.801599	-4.170209	-1.842978
H	0.381556	-2.240866	1.858974	C	4.112812	-2.928844	0.485521
C	-0.860875	1.235504	2.222076	H	4.375004	-3.922090	0.897198
C	-1.027513	0.014666	2.965890	H	5.038356	-2.325291	0.437415
H	-0.280182	2.050165	2.676849	H	3.407528	-2.436546	1.173163

C	4.522382	-3.790457	-1.827298
H	4.131921	-3.980907	-2.844039
H	5.458750	-3.209191	-1.917315
H	4.780034	-4.769851	-1.384547

[1]2-β-T

Zero-point correction= 0.602895 (Hartree/Particle)

Thermal correction to Energy= 0.646467

Thermal correction to Enthalpy= 0.647411

Thermal correction to Gibbs Free Energy= 0.523290

Sum of electronic and zero-point Energies= -2539.280193

Sum of electronic and thermal Energies= -2539.236621

Sum of electronic and thermal Enthalpies= -2539.235677

Sum of electronic and thermal Free Energies=-2539.359798

E solvent= -2539.22991673

C	1.964979	2.692297	0.454960
C	3.351541	2.660419	0.712477
H	3.948157	1.813624	0.356209
C	3.962322	3.712255	1.415514
H	5.040835	3.679046	1.608031
C	3.199107	4.800843	1.866267
H	3.678697	5.619941	2.414005
C	1.816447	4.837314	1.613054
H	1.215429	5.685295	1.961258
C	1.200231	3.788508	0.915443
C	0.213074	2.056878	-1.775151
C	0.825587	3.160235	-2.414300
H	1.775959	3.541149	-2.025122
C	0.234439	3.791051	-3.516819
H	0.737252	4.638409	-3.996005
C	-1.002135	3.331811	-3.994275
H	-1.477350	3.810241	-4.857838
C	-1.631645	2.253716	-3.361074
H	-2.592881	1.888355	-3.740076
C	-1.047843	1.594220	-2.252527
C	-1.828637	0.484903	-1.633018
C	-3.186708	0.709903	-1.326665
C	-3.981732	-0.306029	-0.770572
C	-3.438388	-1.578156	-0.536244
C	-2.091370	-1.816629	-0.845989
C	-1.274074	-0.790100	-1.364144
C	2.415763	0.198838	-1.082884
C	3.290994	0.547085	-2.132870
H	3.181920	1.509222	-2.648079
C	4.289520	-0.351724	-2.506725
H	4.979389	-0.113120	-3.323407
C	4.414822	-1.574426	-1.808648
H	5.224607	-2.247059	-2.108474
C	3.565571	-1.967466	-0.763299
C	2.489896	-1.067070	-0.408270
Ni	0.238334	-0.111466	0.964568

O	1.589850	-1.382373	0.515943
P	1.128380	1.269318	-0.371941
H	0.121816	3.826185	0.720545
C	-1.121753	0.893946	1.846259
C	-1.145596	-0.341234	2.720133
H	-0.746774	1.808954	2.333954
H	-2.025904	1.082850	1.247857
H	-2.115959	-0.870081	2.656006
H	-0.246068	-1.017863	-1.661975
H	-3.619943	1.700368	-1.498853
C	3.768201	-3.296819	-0.002078
C	2.527952	-4.211601	-0.183314
H	2.672210	-5.156572	0.375015
H	1.616980	-3.719814	0.186710
H	2.379866	-4.465516	-1.249000
C	3.975523	-2.996269	1.508537
H	4.091934	-3.942066	2.071273
H	4.887774	-2.391360	1.666518
H	3.113133	-2.446434	1.917992
C	5.008294	-4.073843	-0.503862
H	4.913934	-4.368354	-1.565492
H	5.941355	-3.492183	-0.385720
H	5.117778	-5.001227	0.087326
H	-0.434860	-1.159044	2.288842
C	-0.693617	-0.154102	4.184855
H	-0.492898	-1.142670	4.643049
H	0.267377	0.397415	4.193747
C	-1.737146	0.600343	5.030232
H	-4.061048	-2.378610	-0.128613
C	-1.510756	-3.189402	-0.588651
F	-0.538917	-3.503203	-1.483031
F	-0.959046	-3.276117	0.662278
F	-2.467906	-4.159294	-0.658663
C	-5.443277	-0.042361	-0.490766
F	-5.930154	-0.865455	0.479968
F	-5.652898	1.243665	-0.086113
F	-6.210269	-0.237301	-1.603296
C	-1.285682	0.811832	6.484598
H	-1.949862	1.579278	4.557651
H	-2.692019	0.037136	5.014850
H	-2.051937	1.351791	7.069075
H	-1.096615	-0.153410	6.990004
H	-0.350844	1.400744	6.531025

^{1}TS-II$_{\mathbf{Decoor}}$

Zero-point correction= 0.598447 (Hartree/Particle)

Thermal correction to Energy= 0.643464

Thermal correction to Enthalpy= 0.644408

Thermal correction to Gibbs Free Energy= 0.518712

Sum of electronic and zero-point Energies= -2539.229662

Sum of electronic and thermal Energies= -2539.184645

Sum of electronic and thermal Enthalpies=

-2539.183701

Sum of electronic and thermal Free Energies= -2539.309397

E solvent= -2539.18306456

C	-2.699151	-2.294055	0.211619
C	-4.047306	-1.899092	0.086787
H	-4.286524	-0.923483	-0.350174
C	-5.081478	-2.754472	0.507865
H	-6.124190	-2.432812	0.403520
C	-4.783998	-4.012055	1.053879
H	-5.591581	-4.677315	1.379687
C	-3.442299	-4.414225	1.184088
H	-3.200556	-5.394781	1.610552
C	-2.408632	-3.559458	0.774983
C	-0.443816	-2.132988	-1.572677
C	-1.194181	-3.087169	-2.298669
H	-2.251035	-3.231721	-2.053280
C	-0.618150	-3.871403	-3.308008
H	-1.233894	-4.596223	-3.852323
C	0.743236	-3.724672	-3.606960
H	1.212655	-4.331948	-4.388880
C	1.502653	-2.780906	-2.904637
H	2.560120	-2.639395	-3.156292
C	0.935162	-1.967028	-1.895538
C	1.846881	-0.967081	-1.262157
C	3.102632	-1.386351	-0.776974
C	4.035753	-0.452568	-0.293590
C	3.739361	0.917558	-0.308780
C	2.490193	1.342342	-0.790897
C	1.539294	0.412998	-1.243308
C	-2.020114	0.302908	-1.013342
C	-2.748692	0.277734	-2.220964
H	-2.856046	-0.661768	-2.777622
C	-3.325717	1.454836	-2.697749
H	-3.893336	1.464526	-3.634484
C	-3.170888	2.638160	-1.947444
H	-3.646112	3.548714	-2.332293
C	-2.446954	2.728377	-0.745996
C	-1.810357	1.519175	-0.267636
Ni	-0.324277	-0.094573	1.499235
O	-1.048132	1.530571	0.816527
P	-1.293654	-1.164547	-0.223111
H	-1.365245	-3.880880	0.885448
C	0.898682	0.802609	2.718418
H	1.350948	1.633589	2.165733
C	1.535300	-0.535051	2.648332
H	0.792610	-1.288521	2.065648
H	0.428232	1.092048	3.669559
C	-2.268006	-0.073914	3.059692
C	-1.865043	-1.252933	3.591907
H	-2.011300	0.889014	3.513271
H	-2.980663	-0.038419	2.228558
H	-1.222588	-1.291235	4.478919
H	-2.181106	-2.211546	3.166469
H	2.419215	-0.552950	1.989457
H	0.583316	0.763937	-1.642744

H	3.353423	-2.452095	-0.776231
C	1.812116	-1.273488	3.970859
H	0.929571	-1.256716	4.632041
H	2.096666	-2.326865	3.799634
H	2.643170	-0.775018	4.499667
H	4.478022	1.646122	0.037068
C	-2.398813	4.116622	-0.044375
C	-1.576250	4.177271	1.266320
H	-1.635813	5.208361	1.666728
H	-1.965006	3.486206	2.030940
H	-0.517872	3.929296	1.098158
C	-3.852800	4.544110	0.304181
H	-3.851347	5.543491	0.779370
H	-4.500817	4.598389	-0.588836
H	-4.313247	3.830895	1.012156
C	-1.781973	5.160418	-1.018116
H	-0.746172	4.883512	-1.278427
H	-2.356453	5.250788	-1.957162
H	-1.762753	6.158448	-0.540530
C	5.324979	-0.939323	0.324245
F	6.304758	0.003879	0.278137
F	5.142814	-1.263299	1.642893
F	5.796375	-2.058893	-0.293478
C	2.195562	2.825764	-0.806304
F	3.251732	3.529956	-1.309347
F	1.104656	3.128267	-1.554813
F	1.978801	3.310328	0.456413

[1]1-β-C

Zero-point correction= 0.546659 (Hartree/Particle)

Thermal correction to Energy= 0.588320

Thermal correction to Enthalpy= 0.589264

Thermal correction to Gibbs Free Energy= 0.469653

Sum of electronic and zero-point Energies= -2460.706478

Sum of electronic and thermal Energies= -2460.664816

Sum of electronic and thermal Enthalpies= -2460.663872

Sum of electronic and thermal Free Energies= -2460.783484

E solvent= -2460.63494088

C	2.083419	2.642058	0.650895
C	3.478333	2.506639	0.808963
H	3.985963	1.626969	0.398922
C	4.213775	3.496434	1.482917
H	5.297659	3.379825	1.597600
C	3.568290	4.629031	2.002832
H	4.144459	5.399704	2.527364
C	2.177680	4.770309	1.849716
H	1.666163	5.651986	2.253147
C	1.438814	3.781618	1.184485
C	0.184548	2.190547	-1.489744
C	0.820150	3.275890	-2.134884

H	1.827542	3.567576	-1.818398
C	0.178414	4.003384	-3.146873
H	0.698168	4.835480	-3.634889
C	-1.131554	3.666210	-3.519383
H	-1.648268	4.229370	-4.304527
C	-1.780564	2.599628	-2.883891
H	-2.798624	2.323967	-3.182708
C	-1.140867	1.840986	-1.876553
C	-1.910840	0.713711	-1.270732
C	-3.187039	0.959081	-0.725553
C	-3.959608	-0.094410	-0.206420
C	-3.489996	-1.414620	-0.264223
C	-2.223575	-1.667607	-0.815090
C	-1.422272	-0.613731	-1.286402
C	2.233322	0.127903	-0.880980
C	3.057751	0.388982	-1.995832
H	2.976404	1.345899	-2.525955
C	3.965320	-0.582519	-2.415767
H	4.609369	-0.409468	-3.284746
C	4.054990	-1.795935	-1.698215
H	4.790109	-2.532394	-2.037337
C	3.258765	-2.105165	-0.584785
C	2.281230	-1.120431	-0.175622
Ni	0.169712	-0.106759	1.330359
O	1.452820	-1.375959	0.838454
P	1.072034	1.301276	-0.126897
H	0.354251	3.901449	1.071078
C	-0.611377	-1.126011	2.773696
H	-1.102455	-2.021373	2.373791
C	-1.367527	0.141951	2.830995
H	-1.031940	0.876422	1.899339
H	0.139941	-1.318174	3.552701
H	-2.431424	0.036552	2.551161
H	-0.445541	-0.831337	-1.728790
H	-3.574892	1.982396	-0.694709
C	-1.165010	1.037807	4.064176
H	-0.092296	1.139601	4.301777
H	-1.582921	2.048551	3.911689
H	-1.670445	0.585265	4.935325
H	-4.109559	-2.237541	0.103518
C	3.421636	-3.433797	0.188056
C	2.104473	-4.252565	0.128221
H	2.219841	-5.193778	0.699460
H	1.265450	-3.682616	0.553452
H	1.852740	-4.515957	-0.915056
C	3.778253	-3.127278	1.669141
H	3.876971	-4.070863	2.238901
H	4.740647	-2.586578	1.736933
H	2.994801	-2.511881	2.138991
C	4.551696	-4.313960	-0.396392
H	4.348695	-4.612887	-1.441471
H	5.534466	-3.808082	-0.363712
H	4.634567	-5.239712	0.201558
C	-5.264706	0.206503	0.491528
F	-6.143401	-0.828582	0.405715
F	-5.059706	0.444425	1.827086

F	-5.873940	1.313739	-0.015814
C	-1.738199	-3.099436	-0.877556
F	-2.710289	-3.922153	-1.370906
F	-0.641257	-3.239112	-1.662265
F	-1.423763	-3.575853	0.365854

^{1}TS-I$_{BHE-C}$

Zero-point correction= 0.543552 (Hartree/Particle)

Thermal correction to Energy= 0.585140

Thermal correction to Enthalpy= 0.586084

Thermal correction to Gibbs Free Energy= 0.466149

Sum of electronic and zero-point Energies= -2460.703479

Sum of electronic and thermal Energies= -2460.661891

Sum of electronic and thermal Enthalpies= -2460.660947

Sum of electronic and thermal Free Energies= -2460.780882

E solvent= -2460.63043801

C	2.162688	2.568296	0.648166
C	3.569790	2.495826	0.592196
H	4.048257	1.675115	0.046924
C	4.352690	3.471198	1.233405
H	5.445692	3.404774	1.184639
C	3.741227	4.523402	1.932269
H	4.354320	5.281774	2.432316
C	2.338240	4.598745	1.994349
H	1.854808	5.415332	2.543059
C	1.553043	3.624595	1.362310
C	0.207919	2.207750	-1.462109
C	0.863537	3.297370	-2.079020
H	1.875028	3.564771	-1.754160
C	0.235941	4.058842	-3.074955
H	0.770518	4.893764	-3.541551
C	-1.078836	3.753216	-3.456753
H	-1.584824	4.343825	-4.228566
C	-1.746917	2.683086	-2.847261
H	-2.769645	2.432541	-3.152104
C	-1.121916	1.889024	-1.858861
C	-1.909653	0.762996	-1.276392
C	-3.172589	1.021359	-0.708559
C	-3.951344	-0.026490	-0.187814
C	-3.501792	-1.352119	-0.266846
C	-2.250200	-1.617244	-0.846138
C	-1.443856	-0.570887	-1.322598
C	2.190986	0.058038	-0.913979
C	2.986773	0.268206	-2.060041
H	2.914943	1.211696	-2.615588
C	3.854589	-0.741912	-2.474711
H	4.477418	-0.613869	-3.366608
C	3.932489	-1.935579	-1.721944
H	4.639032	-2.700722	-2.058635
C	3.158859	-2.191869	-0.579531
C	2.213882	-1.172787	-0.176531

Ni	0.134790	-0.014903	1.326139
O	1.389810	-1.366384	0.848828
P	1.097339	1.273921	-0.132788
H	0.459415	3.682582	1.423549
C	-0.635933	-1.186293	2.761961
H	-1.105215	-2.013593	2.219010
C	-1.293074	0.057861	2.905784
H	-0.822048	1.103119	1.612179
H	0.213998	-1.443386	3.406547
H	-2.323232	0.128065	2.526792
H	-0.475705	-0.797160	-1.779213
H	-3.541253	2.050663	-0.653535
C	-0.964037	1.003837	4.051883
H	0.105049	0.953234	4.319307
H	-1.210966	2.051800	3.809925
H	-1.554775	0.714926	4.942311
H	-4.123927	-2.169182	0.109752
C	3.309456	-3.498573	0.231561
C	1.975637	-4.292319	0.221078
H	2.079460	-5.211131	0.829767
H	1.153501	-3.687559	0.631155
H	1.706038	-4.593595	-0.807311
C	3.697780	-3.150437	1.695289
H	3.786320	-4.075864	2.295724
H	4.672131	-2.628303	1.729021
H	2.935540	-2.502041	2.155568
C	4.411642	-4.419366	-0.342564
H	4.183960	-4.748692	-1.373351
H	5.403941	-3.931366	-0.344153
H	4.488255	-5.326468	0.284181
C	-5.234839	0.290074	0.542639
F	-6.120562	-0.741762	0.496707
F	-4.990388	0.547004	1.867459
F	-5.853118	1.392803	0.036053
C	-1.791570	-3.055730	-0.937032
F	-2.745512	-3.836234	-1.524792
F	-0.648100	-3.191917	-1.651934
F	-1.572586	-3.587470	0.306159

[1]I-BHE-C

Zero-point correction= 0.544442 (Hartree/Particle)

Thermal correction to Energy= 0.586445

Thermal correction to Enthalpy= 0.587389

Thermal correction to Gibbs Free Energy= 0.466987

Sum of electronic and zero-point Energies= -2460.703393

Sum of electronic and thermal Energies= -2460.661390

Sum of electronic and thermal Enthalpies= -2460.660446

Sum of electronic and thermal Free Energies= -2460.780848

E solvent= -2460.63174634

C	-2.192749	-2.537426	0.640833
C	-3.597497	-2.456666	0.547400

H	-4.056464	-1.642068	-0.023242
C	-4.402514	-3.415696	1.185763
H	-5.493489	-3.343223	1.109064
C	-3.815181	-4.458867	1.918045
H	-4.445470	-5.204196	2.416302
C	-2.414687	-4.541789	2.016883
H	-1.950773	-5.350947	2.592702
C	-1.607027	-3.584151	1.388110
C	-0.210281	-2.221729	-1.456490
C	-0.873326	-3.309432	-2.068454
H	-1.889902	-3.564003	-1.749156
C	-0.245476	-4.086223	-3.052431
H	-0.784709	-4.920050	-3.515540
C	1.075651	-3.797988	-3.425382
H	1.582011	-4.401333	-4.187010
C	1.749892	-2.728944	-2.820524
H	2.777779	-2.492031	-3.118728
C	1.125272	-1.918747	-1.845379
C	1.917457	-0.793424	-1.267865
C	3.169564	-1.057044	-0.679428
C	3.950281	-0.010544	-0.159560
C	3.514013	1.317922	-0.259821
C	2.273590	1.587831	-0.860833
C	1.465326	0.543659	-1.338079
C	-2.167520	-0.034935	-0.940570
C	-2.948408	-0.226511	-2.100117
H	-2.879874	-1.165543	-2.663549
C	-3.797731	0.798883	-2.515679
H	-4.409965	0.687774	-3.417088
C	-3.871320	1.985929	-1.751453
H	-4.563765	2.762887	-2.090449
C	-3.110425	2.222235	-0.596298
C	-2.181508	1.188669	-0.191510
Ni	-0.119974	-0.039873	1.314845
O	-1.362826	1.357834	0.840395
P	-1.104959	-1.268573	-0.147435
H	-0.515892	-3.645011	1.479572
C	0.604210	1.255369	2.737985
H	1.109524	2.015663	2.133243
C	1.205941	0.029780	3.015668
H	0.720357	-1.264607	1.428102
H	-0.300901	1.574873	3.268418
H	2.226436	-0.139853	2.646465
H	0.505239	0.773545	-1.809590
H	3.526388	-2.089225	-0.605047
C	0.749961	-0.881910	4.137352
H	-0.316461	-0.730050	4.376151
H	0.902729	-1.946468	3.891747
H	1.338599	-0.663193	5.050578
H	4.137243	2.133256	0.118733
C	-3.255675	3.522657	0.225382
C	-1.912467	4.300478	0.238468
H	-2.011582	5.212956	0.857300
H	-1.101763	3.680731	0.649037
H	-1.628642	4.610781	-0.783336
C	-3.666071	3.164146	1.680484

H	-3.746274	4.083643	2.291075
H	-4.648992	2.657512	1.697337
H	-2.919979	2.497679	2.141463
C	-4.339902	4.462012	-0.352629
H	-4.095307	4.799377	-1.376903
H	-5.337549	3.985430	-0.371834
H	-4.414277	5.363421	0.282552
C	5.220310	-0.331702	0.592376
F	6.110773	0.696975	0.559924
F	4.953950	-0.587940	1.911979
F	5.842592	-1.436611	0.094636
C	1.830862	3.029180	-0.977038
F	2.781725	3.784460	-1.601359
F	0.674748	3.163863	-1.671811
F	1.643612	3.591977	0.258084

[1]TS-I$_{Transf-1}$

Zero-point correction= 0.596192 (Hartree/Particle)

Thermal correction to Energy= 0.641661

Thermal correction to Enthalpy= 0.642605

Thermal correction to Gibbs Free Energy= 0.515549

Sum of electronic and zero-point Energies= -2539.221907

Sum of electronic and thermal Energies= -2539.176437

Sum of electronic and thermal Enthalpies= -2539.175493

Sum of electronic and thermal Free Energies= -2539.302549

E solvent= -2539.17985154

C	-2.925536	-1.979088	-0.036465
C	-4.045931	-1.566257	-0.787926
H	-3.940595	-0.751992	-1.513315
C	-5.291635	-2.190314	-0.600698
H	-6.156660	-1.858384	-1.186487
C	-5.428883	-3.232471	0.330187
H	-6.401410	-3.716565	0.474874
C	-4.314906	-3.649272	1.081415
H	-4.416941	-4.458801	1.813486
C	-3.072777	-3.022396	0.906289
C	-0.315193	-2.388028	-1.231695
C	-1.045000	-3.431763	-1.848156
H	-2.131137	-3.469095	-1.727112
C	-0.418762	-4.433816	-2.602923
H	-1.023484	-5.220024	-3.068431
C	0.973633	-4.421394	-2.749062
H	1.483930	-5.197625	-3.330014
C	1.714302	-3.393769	-2.152800
H	2.801326	-3.360896	-2.288755
C	1.101866	-2.362751	-1.400927
C	2.031179	-1.319597	-0.867549
C	3.169223	-1.729032	-0.141284
C	4.146336	-0.799531	0.254168
C	4.009193	0.553848	-0.077856
C	2.878089	0.968906	-0.801948

C	1.892626	0.049233	-1.191657
C	-1.533614	0.323900	-1.249261
C	-1.641549	0.331117	-2.656454
H	-1.558260	-0.607523	-3.218616
C	-1.848109	1.545320	-3.312315
H	-1.934919	1.584144	-4.403517
C	-1.941593	2.732591	-2.552026
H	-2.095698	3.669754	-3.095768
C	-1.835583	2.775968	-1.153282
C	-1.613032	1.523193	-0.462174
Ni	-1.073021	-0.200475	1.750691
O	-1.467221	1.470827	0.855720
P	-1.278464	-1.147159	-0.218910
H	-2.210764	-3.330927	1.509301
C	-1.574514	0.578472	3.628867
H	-0.804561	0.190661	4.302303
C	-2.638746	-0.226947	3.230669
H	-0.662415	-1.520623	2.308095
H	-1.601209	1.661685	3.463657
H	-2.659291	-1.265506	3.587626
H	1.031819	0.390821	-1.773367
H	3.290121	-2.785745	0.119160
C	-3.928068	0.294092	2.646091
H	-3.790233	1.285558	2.187700
H	-4.342347	-0.392634	1.888647
H	-4.682528	0.380488	3.455638
H	4.772972	1.276736	0.221530
C	-1.931361	4.107359	-0.373702
C	-0.623579	4.347821	0.428949
H	-0.698593	5.294088	0.998215
H	-0.444289	3.522177	1.135463
H	0.245468	4.421363	-0.247279
C	-3.135007	4.052229	0.605516
H	-3.202343	4.995421	1.180598
H	-4.085509	3.918244	0.056576
H	-3.017249	3.218220	1.315153
C	-2.140649	5.321295	-1.309021
H	-1.302225	5.449162	-2.018242
H	-3.078974	5.244841	-1.889090
H	-2.202511	6.241467	-0.699999
C	1.692341	1.504147	2.897335
C	1.668042	0.163015	2.791810
H	1.548283	2.009282	3.860480
H	1.852385	2.148605	2.026877
H	1.526581	-0.486238	3.663635
H	1.829889	-0.345255	1.835956
C	5.314432	-1.275085	1.088874
F	6.322439	-0.361855	1.128185
F	4.936417	-1.521182	2.376010
F	5.831554	-2.440316	0.600252
C	2.775740	2.428050	-1.189076
F	2.835100	3.237014	-0.085008
F	3.814860	2.798384	-1.993179
F	1.624387	2.715166	-1.846029

^{1}I-Transf-1

Zero-point correction= 0.597023 (Hartree/Particle)

Thermal correction to Energy= 0.642535

Thermal correction to Enthalpy= 0.643479

Thermal correction to Gibbs Free Energy= 0.516755

Sum of electronic and zero-point Energies= -2.539.1027786

Sum of electronic and thermal Energies= -2.539.0572666

Sum of electronic and thermal Enthalpies= -2539.0563226

Sum of electronic and thermal Free Energies= -2539.1830466

E solvent= -2538.66629

C	-2.902195	-2.009458	-0.102068
C	-3.984095	-1.620466	-0.919596
H	-3.858330	-0.791638	-1.625121
C	-5.219513	-2.285512	-0.824611
H	-6.054093	-1.970365	-1.461641
C	-5.385768	-3.345002	0.081309
H	-6.350117	-3.860416	0.155214
C	-4.311286	-3.738154	0.899459
H	-4.435357	-4.561695	1.612293
C	-3.080760	-3.070710	0.813925
C	-0.257771	-2.335447	-1.204753
C	-0.959717	-3.384743	-1.845776
H	-2.043358	-3.455281	-1.723704
C	-0.315462	-4.352056	-2.630215
H	-0.905892	-5.140747	-3.109790
C	1.073771	-4.300411	-2.789132
H	1.599962	-5.046626	-3.394699
C	1.789651	-3.268906	-2.170613
H	2.874202	-3.203194	-2.314071
C	1.158158	-2.274083	-1.384978
C	2.085423	-1.236379	-0.833430
C	3.227421	-1.664889	-0.123033
C	4.215834	-0.750235	0.278893
C	4.088292	0.608020	-0.035260
C	2.954102	1.042335	-0.743090
C	1.953382	0.138436	-1.133426
C	-1.498474	0.358448	-1.168509
C	-1.616410	0.365233	-2.575927
H	-1.527936	-0.573486	-3.137272
C	-1.835685	1.574864	-3.234652
H	-1.924162	1.610946	-4.325799
C	-1.937434	2.761551	-2.474877
H	-2.104975	3.696047	-3.018954
C	-1.821956	2.808928	-1.077608
C	-1.575799	1.559043	-0.381698
Ni	-0.988409	-0.182081	1.825894
O	-1.420167	1.521753	0.932449
P	-1.264201	-1.136300	-0.162758
H	-2.249624	-3.367305	1.465439
C	-1.876159	0.395794	3.655321
H	-1.252843	-0.086317	4.416910
C	-2.866479	-0.319093	2.986460

H	-0.771354	-1.567438	2.320977
H	-1.880646	1.492262	3.629785
H	-2.963829	-1.391199	3.203402
H	1.088937	0.493887	-1.701896
H	3.345457	-2.727791	0.113131
C	-4.047833	0.324538	2.304755
H	-3.812892	1.346800	1.970709
H	-4.395189	-0.260496	1.437666
H	-4.893918	0.376225	3.020903
H	4.865087	1.319704	0.257308
C	-1.930939	4.145447	-0.306667
C	-0.617759	4.417068	0.475183
H	-0.696270	5.373981	1.025983
H	-0.424444	3.609028	1.197581
H	0.243581	4.484529	-0.211128
C	-3.117548	4.081998	0.691946
H	-3.198671	5.035315	1.248347
H	-4.073447	3.912961	0.162611
H	-2.966746	3.267421	1.417475
C	-2.174845	5.348153	-1.248686
H	-1.348040	5.487621	-1.969329
H	-3.118626	5.249482	-1.816356
H	-2.246906	6.271269	-0.645261
C	0.946795	0.937878	2.467821
C	1.188244	-0.389704	2.685739
H	0.584984	1.590737	3.268570
H	1.204800	1.427487	1.525979
H	1.048102	-0.840399	3.674220
H	1.675391	-1.013175	1.931649
C	5.388591	-1.246586	1.094392
F	6.416412	-0.355175	1.110197
F	5.027511	-1.474072	2.391053
F	5.871178	-2.426275	0.607085
C	2.833246	2.518145	-1.053129
F	2.583405	3.233668	0.087643
F	3.993607	3.012333	-1.574298
F	1.835978	2.789117	-1.931148

[1]TS-III$_{Decoor}$

Zero-point correction= 0.595935 (Hartree/Particle)

Thermal correction to Energy= 0.641222

Thermal correction to Enthalpy= 0.642166

Thermal correction to Gibbs Free Energy= 0.515793

Sum of electronic and zero-point Energies= -2539.224199

Sum of electronic and thermal Energies= -2539.178913

Sum of electronic and thermal Enthalpies= -2539.177968

Sum of electronic and thermal Free Energies= -2539.30434

C	-2.741881	-2.186158	0.083339
C	-3.983686	-1.870801	-0.506196
H	-4.065926	-0.999190	-1.165102
C	-5.111882	-2.672815	-0.255465

H	-6.071381	-2.415394	-0.718808	H	-2.912066	3.530826	-2.914732
C	-5.012458	-3.795368	0.580362	C	-2.139288	2.730790	-1.071235
H	-5.893057	-4.418328	0.773763	C	-1.660868	1.521973	-0.425505
C	-3.777429	-4.115318	1.173214	Ni	-0.560558	-0.123190	1.612892
H	-3.692072	-4.988507	1.830347	O	-1.182757	1.538994	0.809574
C	-2.652353	-3.314005	0.931611	P	-1.227339	-1.147871	-0.188929
C	-0.233936	-2.272158	-1.310222	H	-1.694599	-3.561905	1.405841
C	-0.941134	-3.278476	-2.010889	C	0.949160	1.067650	2.595605
H	-2.022180	-3.366997	-1.871598	H	1.295713	1.676200	1.756322
C	-0.299387	-4.182680	-2.868954	C	1.324695	-0.258011	2.729134
H	-0.888073	-4.941955	-3.395809	H	-0.224774	-1.513267	2.033588
C	1.088915	-4.110542	-3.036543	H	0.451230	1.602633	3.409881
H	1.612157	-4.811658	-3.696164	H	2.026244	-0.722808	2.030851
C	1.807129	-3.117837	-2.359645	H	1.000755	0.581575	-1.660899
H	2.889415	-3.034275	-2.510890	H	3.411184	-2.644719	-0.067007
C	1.175621	-2.181336	-1.505987	H	4.789482	1.443359	0.258629
C	2.081418	-1.152268	-0.910278	C	-2.158076	4.083923	-0.322445
C	3.250356	-1.576247	-0.244858	C	-0.713007	4.488033	0.071440
C	4.211025	-0.646468	0.190132	H	-0.723848	5.447391	0.623695
C	4.030889	0.720689	-0.053976	H	-0.255303	3.718196	0.710567
C	2.868848	1.149512	-0.718965	H	-0.081118	4.615114	-0.824476
C	1.890941	0.230523	-1.130448	C	-3.024208	3.964647	0.960141
C	-1.714805	0.296790	-1.176078	H	-3.029300	4.927054	1.506479
C	-2.136692	0.248268	-2.522954	H	-4.070201	3.709452	0.707809
H	-2.120671	-0.703412	-3.069137	H	-2.619186	3.184298	1.623222
C	-2.561739	1.422515	-3.143036	C	-2.751257	5.223067	-1.184456
H	-2.888450	1.418047	-4.188510	H	-2.152594	5.413127	-2.094329
C	-2.564259	2.627310	-2.404155	H	-3.793146	5.014405	-1.490795

H	-2.758635	6.157080	-0.593629
C	-1.837981	0.164184	3.725401
C	-2.852703	-0.374128	2.998484
H	-1.127878	-0.465249	4.271438
H	-1.783722	1.246259	3.894410
H	-2.916388	-1.466447	2.905101
C	-3.980919	0.417458	2.396007
C	5.405068	-1.135726	0.977464
F	5.907742	-2.293298	0.459615
F	6.413098	-0.222472	1.003303
F	5.063266	-1.399550	2.273913
C	2.695732	2.631286	-0.967897
F	2.406482	3.295528	0.194439
F	3.842490	3.188282	-1.454649
F	1.700288	2.896653	-1.851089
H	1.165897	-0.795963	3.670661
H	-4.109984	0.196428	1.321621
H	-4.935234	0.146449	2.890231
H	-3.823897	1.501679	2.511123

[1]II-BHE-C

Zero-point correction= 0.516823 (Hartree/Particle)

Thermal correction to Energy= 0.557263

Thermal correction to Enthalpy= 0.558208

Thermal correction to Gibbs Free Energy= 0.441758

Sum of electronic and zero-point Energies= -2421.416401

Sum of electronic and thermal Energies= -2421.375960

Sum of electronic and thermal Enthalpies= -2421.375016

Sum of electronic and thermal Free Energies= -2421.491466

E solvent= -2421.33390489

C	-2.174145	2.540713	-0.798334
C	-3.578577	2.474205	-0.689703
H	-4.038494	1.683173	-0.087600
C	-4.382057	3.416820	-1.353853
H	-5.472799	3.355456	-1.265278
C	-3.793554	4.429236	-2.127200
H	-4.422782	5.161620	-2.645552
C	-2.393517	4.497378	-2.241855
H	-1.928724	5.281657	-2.850372
C	-1.587341	3.555847	-1.587377
C	-0.177667	2.276780	1.296373
C	-0.832953	3.380607	1.887449
H	-1.851512	3.630345	1.570475
C	-0.195261	4.178583	2.847780
H	-0.728546	5.024541	3.295473
C	1.127986	3.895125	3.216981
H	1.641885	4.514265	3.960653
C	1.795003	2.811067	2.631188
H	2.824910	2.579095	2.926252
C	1.160967	1.980010	1.679765

C	1.948331	0.842925	1.118192
C	3.196917	1.096102	0.517362
C	3.975358	0.040654	0.012462
C	3.540213	-1.285932	0.139159
C	2.303057	-1.545524	0.750895
C	1.496747	-0.492972	1.214104
C	-2.148608	0.088025	0.860891
C	-2.925152	0.322168	2.015502
H	-2.853083	1.280602	2.544712
C	-3.775507	-0.685744	2.469367
H	-4.385529	-0.541349	3.367524
C	-3.853162	-1.898939	1.748029
H	-4.546590	-2.661698	2.116028
C	-3.095724	-2.178228	0.600132
C	-2.166640	-1.161183	0.156024
Ni	-0.131720	0.011781	-1.419014
O	-1.351118	-1.369859	-0.872015
P	-1.088267	1.292508	0.022886
H	-0.496727	3.604798	-1.691926
C	1.214393	-0.076555	-3.037785
H	0.894689	0.649806	-3.794702
C	0.525720	-1.278310	-2.872749
H	0.686028	1.243154	-1.604838
H	2.243768	0.038731	-2.678733
H	-0.382143	-1.495477	-3.446209
H	0.541161	-0.714824	1.698558
H	3.553262	2.126762	0.422521
H	0.960819	-2.111089	-2.311022
H	4.162786	-2.107870	-0.225812
C	-3.245734	-3.508183	-0.171838
C	-1.903877	-4.287759	-0.158028
H	-2.006185	-5.225236	-0.737743
H	-1.094145	-3.687027	-0.597346
H	-1.615963	-4.555216	0.874721
C	-3.658910	-3.204975	-1.638738
H	-3.739143	-4.146981	-2.213869
H	-4.642369	-2.700294	-1.673298
H	-2.914086	-2.556005	-2.126005
C	-4.330330	-4.422473	0.444692
H	-4.082461	-4.722837	1.479618
H	-5.326410	-3.942343	0.450389
H	-4.409790	-5.346278	-0.156771
C	5.240983	0.347747	-0.753008
F	6.133873	-0.677950	-0.701548
F	4.966241	0.572675	-2.076445
F	5.862968	1.465059	-0.283803
C	1.858127	-2.984475	0.888778
F	1.588160	-3.541516	-0.333457
F	2.841227	-3.748683	1.448731
F	0.747393	-3.113420	1.655123

Catalyst 2

^{2}1-β-T

Zero-point correction= 0.592541 (Hartree/Particle)

Thermal correction to Energy= 0.630353

Thermal correction to Enthalpy= 0.631297

Thermal correction to Gibbs Free Energy= 0.521268

Sum of electronic and zero-point Energies= -1865.225816

Sum of electronic and thermal Energies= -1865.188005

Sum of electronic and thermal Enthalpies= -1865.187061

Sum of electronic and thermal Free Energies= -1865.297089

E solvent= -1865.11254432

C	0.128736	2.698371	0.860558
C	1.410144	3.258568	1.046679
H	2.281095	2.780126	0.585931
C	1.566706	4.427214	1.810303
H	2.566868	4.855579	1.943743
C	0.450969	5.046806	2.395213
H	0.576004	5.959073	2.989569
C	-0.828450	4.492545	2.215070
H	-1.704497	4.972087	2.667173
C	-0.990361	3.324354	1.456230
C	-1.414854	1.476998	-1.275574
C	-1.429310	2.772011	-1.846434
H	-0.691440	3.511278	-1.516077
C	-2.381573	3.140606	-2.805439
H	-2.362582	4.148246	-3.235601
C	-3.357564	2.212783	-3.200296
H	-4.109726	2.483153	-3.950169
C	-3.366065	0.932938	-2.633978
H	-4.119253	0.202225	-2.949634
C	-2.405022	0.529972	-1.674690
C	-2.520543	-0.850302	-1.123367
C	-3.776519	-1.295464	-0.652213
C	-3.949009	-2.589860	-0.129343
C	-2.832426	-3.451068	-0.091767
C	-1.571802	-3.051020	-0.571298
C	-1.419034	-1.733864	-1.065642
C	1.462712	0.777784	-0.953570
C	1.979018	1.578140	-1.992912
H	1.403744	2.434089	-2.366038
C	3.224347	1.265367	-2.537957
H	3.646145	1.864807	-3.352009
C	3.949832	0.173053	-2.013062
H	4.937775	-0.029115	-2.438877
C	3.476907	-0.654924	-0.983045
C	2.157007	-0.377658	-0.457044
Ni	-0.113907	-0.629060	1.174102
O	1.592619	-1.151433	0.465049
P	-0.083654	1.101858	-0.044906
H	-1.993068	2.903208	1.317034
C	-1.638599	-0.364804	2.286324
C	-1.052030	-1.572145	2.982311
H	-1.607835	0.574185	2.863058
H	-2.600082	-0.521853	1.774773
H	-1.725638	-2.446720	2.917152
H	-0.456532	-1.433569	-1.495935

H	-4.627941	-0.603440	-0.669600
C	4.338298	-1.801596	-0.405094
C	3.631337	-3.166485	-0.616194
H	4.252347	-3.986071	-0.206192
H	2.656132	-3.176152	-0.105989
H	3.470056	-3.364785	-1.691944
C	4.554986	-1.561853	1.114852
H	5.138698	-2.394176	1.552910
H	5.116252	-0.624775	1.286454
H	3.587960	-1.493920	1.638364
C	5.730941	-1.882300	-1.073614
H	5.662734	-2.098675	-2.155886
H	6.308930	-0.949088	-0.941660
H	6.310373	-2.700270	-0.607823
H	-0.132251	-1.976648	2.383941
C	-0.530913	-1.342940	4.412152
H	0.016041	-2.222622	4.796003
H	0.150942	-0.475228	4.442750
H	-1.375815	-1.138142	5.093512
H	-2.952615	-4.467367	0.307715
C	-0.400612	-4.010813	-0.585607
H	-0.233077	-4.420481	-1.600545
H	0.528388	-3.497392	-0.283492
H	-0.569079	-4.866604	0.091692
C	-5.304845	-3.057606	0.362799
H	-5.214103	-3.683034	1.269120
H	-5.966204	-2.206393	0.600870
H	-5.819937	-3.671125	-0.402066

^{2}TS-II$_{Coor}$

Zero-point correction= 0.644067 (Hartree/Particle)

Thermal correction to Energy= 0.685667

Thermal correction to Enthalpy= 0.686611

Thermal correction to Gibbs Free Energy= 0.568507

Sum of electronic and zero-point Energies= -1943.753651

Sum of electronic and thermal Energies= -1943.712050

Sum of electronic and thermal Enthalpies= -1943.711106

Sum of electronic and thermal Free Energies= -1943.829210

E solvent= -1943.66354945

C	0.686219	2.725150	0.442334
C	1.972076	3.180567	0.084905
H	2.566965	2.608887	-0.635501
C	2.486018	4.361838	0.647879
H	3.486975	4.705651	0.362337
C	1.725000	5.097561	1.569168
H	2.128737	6.017412	2.007255
C	0.442659	4.647246	1.932258
H	-0.156024	5.215054	2.654043
C	-0.072078	3.466694	1.377935
C	-1.439488	1.689547	-1.241889
C	-1.375663	2.996643	-1.781179

H	-0.508704	3.626234	-1.555827	C	-1.466680	-1.373910	2.764837
C	-2.402079	3.515666	-2.581279	H	-1.358060	0.824796	3.046719
H	-2.317277	4.529402	-2.988567	H	-2.499480	0.293619	1.718798
C	-3.531946	2.728957	-2.846453	H	-2.178970	-2.025568	2.229745
H	-4.346346	3.115723	-3.469631	H	-0.836127	-1.265641	-1.635992
C	-3.611892	1.435174	-2.318784	H	-4.804329	-0.099838	-0.346138
H	-4.482086	0.809700	-2.547020	C	3.872949	-2.429352	-0.832710
C	-2.582460	0.879432	-1.519430	C	2.973446	-3.665023	-0.563814
C	-2.786693	-0.517188	-1.035148	H	3.571479	-4.481100	-0.114604
C	-4.038602	-0.874389	-0.479306	H	2.158567	-3.403443	0.129278
C	-4.313339	-2.191285	-0.073350	H	2.532942	-4.043570	-1.504590
C	-3.305553	-3.166207	-0.234686	C	4.493865	-1.947683	0.507046
C	-2.051344	-2.847749	-0.785004	H	5.085873	-2.761413	0.968185
C	-1.796783	-1.512433	-1.169474	H	5.167194	-1.086504	0.341715
C	1.255842	0.423841	-1.328040	H	3.703696	-1.646110	1.213006
C	1.598283	0.922604	-2.601687	C	5.029838	-2.880752	-1.754856
H	1.040599	1.764207	-3.031329	H	4.662726	-3.274288	-2.720746
C	2.645828	0.325203	-3.304869	H	5.742409	-2.061169	-1.962426
H	2.927793	0.684456	-4.300549	H	5.593179	-3.692399	-1.259354
C	3.349706	-0.747032	-2.713433	H	-0.438782	-1.804217	2.464700
H	4.180726	-1.179549	-3.279605	C	-1.544547	-1.580608	4.290579
C	3.042025	-1.281942	-1.452233	H	-1.375051	-2.637105	4.567477
C	1.922104	-0.705765	-0.738936	H	-0.786922	-0.964443	4.805271
Ni	-0.031249	-0.370537	1.266522	H	-2.541411	-1.281998	4.660650
O	1.506894	-1.187627	0.427098	H	-3.506980	-4.201824	0.071521
P	0.002217	1.134517	-0.214307	C	-0.990581	-3.910488	-0.979188
H	-1.068082	3.115854	1.673495	H	-0.839291	-4.134195	-2.052606
C	-1.584600	0.060462	2.282000	H	-0.018242	-3.573575	-0.579704

H	-1.266095	-4.854494	-0.477501
C	-5.665473	-2.563404	0.503422
H	-6.211461	-1.673963	0.863103
H	-6.303434	-3.058050	-0.254762
H	-5.566319	-3.267483	1.349216
C	1.864842	-0.244839	4.263201
C	1.881531	0.549625	3.174847
H	2.314413	-1.245099	4.251563
H	1.409281	0.073870	5.208977
H	2.370416	0.233104	2.247179
H	1.457494	1.560399	3.189451

^{2}II-Coor-T

Zero-point correction= 0.646682 (Hartree/Particle)

Thermal correction to Energy= 0.687939

Thermal correction to Enthalpy= 0.688883

Thermal correction to Gibbs Free Energy= 0.572346

Sum of electronic and zero-point Energies= -1943.771433

Sum of electronic and thermal Energies= -1943.730177

Sum of electronic and thermal Enthalpies= -1943.729233

Sum of electronic and thermal Free Energies= -1943.845770

E solvent= -1943.67903573

C -0.098434000 2.792576000 0.183632000

C 1.164920000 3.410950000 0.302859000

H 2.065982000 2.859432000 0.014063000

C 1.268220000 4.729583000 0.774431000

H 2.256052000 5.197479000 0.856194000

C 0.115761000 5.448611000 1.129575000

H 0.198564000 6.479341000 1.492620000

C -1.145714000 4.840765000 1.014533000

H -2.051448000 5.394946000 1.286738000

C -1.254047000 3.520157000 0.551880000

C -1.575551000 0.991464000 -1.574206000

C -1.825360000 2.170163000 -2.316859000

H -1.212834000 3.058715000 -2.133222000

C -2.855891000 2.237347000 -3.263968000

H -3.019886000 3.162941000 -3.827064000

C -3.673116000 1.117057000 -3.475048000

H -4.488135000 1.153106000 -

4.206870000

C -3.431859000 -0.060044000 -2.757044000

H -4.045584000 -0.948237000 -2.944649000

C -2.382439000 -0.161724000 -1.811872000

C -2.179339000 -1.489857000 -1.163319000

C -3.284737000 -2.173232000 -0.604268000

C -3.153024000 -3.467715000 -0.076102000

C -1.887016000 -4.090341000 -0.131568000

C -0.769872000 -3.443252000 -0.686819000

C -0.923962000 -2.130519000 -1.185184000

C 1.356693000 0.650568000 -1.174613000

C 1.738409000 1.148916000 -2.436217000

H 1.029671000 1.733492000 -3.035578000

C 3.023764000 0.880806000 -2.908890000

H 3.345249000 1.241620000 -3.891854000

C 3.917731000 0.149967000 -2.095506000

H 4.928302000 -0.019676000 -2.480694000

C 3.582864000 -0.362454000 -0.831653000

C 2.229154000 -0.147485000 -0.362438000

Ni -0.066431000 -0.355806000 1.415838000

O 1.790717000 -0.652592000 0.782614000

P -0.208627000 1.016235000 -0.326749000

H -2.244601000 3.060720000 0.459091000

C -1.888779000 0.039822000 2.052532000

H -2.539013000 0.274996000 1.188507000

H -2.253614000 -0.916953000 2.474890000

H -0.068991000 -1.632813000 -1.657948000

H -4.259910000 -1.671865000 -0.567428000

C 4.619631000 -1.102753000 0.044149000

C 4.145655000 -2.551548000 0.334452000

H 4.882384000 -3.074704000 0.973908000

H 3.174540000 -2.540755000 0.853705000

H 4.040318000 -3.126752000 -0.604076000

C 4.792052000 -0.337566000

1.385111000

H 5.497765000 -0.876090000 2.046115000

H 5.196719000 0.675984000 1.208040000

H 3.824529000 -0.242486000 1.903510000

C 6.007989000 -1.189317000 -0.631926000

H 5.975508000 -1.759400000 -1.578878000

H 6.430647000 -0.189867000 -0.843206000

H 6.709795000 -1.710539000 0.044682000

C -4.339608000 -4.186856000 0.535588000

H -4.536581000 -5.148314000 0.024901000

H -4.165308000 -4.420801000 1.602761000

H -5.257691000 -3.577705000 0.472804000

C 0.570340000 -4.141546000 -0.787868000

H 0.602141000 -5.052360000 -0.164529000

H 0.784057000 -4.446433000 -1.830598000

H 1.392082000 -3.474274000 -0.473528000

H -1.773425000 -5.110080000 0.260982000

C 0.143939000 -2.160697000 2.501601000

C 0.553878000 -1.105424000 3.298639000

H -0.847836000 -2.613277000 2.605420000

H 0.863198000 -2.689531000 1.868860000

H -0.094710000 -0.695410000 4.078953000

H 1.603653000 -0.792405000 3.306959000

C -1.967562000 1.163867000 3.094598000

C -3.423901000 1.468089000 3.513802000

H -1.388091000 0.903270000 4.001002000

H -1.509064000 2.090442000 2.700446000

H -3.465639000 2.274564000 4.269959000

H -4.030081000 1.786339000 2.644842000

H -3.909691000 0.572847000 3.944956000

^{2}TS-II$_{Isom}$

Zero-point correction= 0.646145 (Hartree/Particle)

Thermal correction to Energy= 0.686443

Thermal correction to Enthalpy=

0.687387

Thermal correction to Gibbs Free Energy= 0.573942

Sum of electronic and zero-point Energies= -1943.758797

Sum of electronic and thermal Energies= -1943.718499

Sum of electronic and thermal Enthalpies= -1943.717555

Sum of electronic and thermal Free Energies= -1943.831000

E solvent= -1943.6575453

C	0.378865	2.678142	-0.067507
C	1.641883	3.106544	-0.527836
H	2.240302	2.441882	-1.160298
C	2.128032	4.380644	-0.185233
H	3.111277	4.699921	-0.550511
C	1.360283	5.242268	0.613241
H	1.741441	6.235188	0.877743
C	0.098744	4.824286	1.074395
H	-0.507524	5.491474	1.698395
C	-0.384765	3.550467	0.744132
C	-1.783525	1.301405	-1.413603
C	-1.839596	2.476285	-2.199246
H	-0.987569	3.164777	-2.191998
C	-2.967409	2.791920	-2.969032
H	-2.977928	3.706494	-3.572865
C	-4.075715	1.931685	-2.956853
H	-4.964697	2.161587	-3.555199
C	-4.040334	0.769185	-2.177601
H	-4.897600	0.086351	-2.179779
C	-2.909178	0.424359	-1.398936
C	-2.977827	-0.831056	-0.596660
C	-4.127608	-1.086427	0.188378
C	-4.258702	-2.269543	0.935217
C	-3.211425	-3.214620	0.884744
C	-2.059873	-3.000628	0.106679
C	-1.948100	-1.795524	-0.623360
C	0.934094	0.187016	-1.515893
C	1.081529	0.513951	-2.879174
H	0.418384	1.258848	-3.335331
C	2.065133	-0.119689	-3.640640
H	2.189338	0.118143	-4.702833
C	2.910068	-1.064658	-3.023633
H	3.686235	-1.530605	-3.638670
C	2.808191	-1.428783	-1.670891
C	1.761642	-0.805935	-0.888538
Ni	0.250803	-0.360738	1.517546
O	1.569154	-1.165104	0.380814
P	-0.271830	0.971941	-0.386762
H	-1.366489	3.229974	1.114224
C	1.769143	0.256774	2.613448
H	2.234555	-0.648426	3.039049
C	1.435054	1.327109	3.639568
H	0.879422	2.153936	3.158546
H	2.392703	0.647213	1.791933
H	0.789658	0.925618	4.443637

H	-1.072530	-1.638783	-1.264957
H	-4.922557	-0.331451	0.230412
C	2.728041	1.895620	4.275285
H	3.383997	2.344070	3.507814
H	2.485537	2.679458	5.016465
H	3.301777	1.103657	4.790137
H	-3.301431	-4.145694	1.460550
C	3.789926	-2.440588	-1.033522
C	3.020217	-3.661561	-0.462809
H	3.729502	-4.381104	-0.010688
H	2.305701	-3.340110	0.310727
H	2.466055	-4.187034	-1.262523
C	4.574264	-1.744082	0.112721
H	5.267743	-2.461839	0.591493
H	5.172766	-0.900857	-0.278968
H	3.882758	-1.359300	0.878476
C	4.823759	-2.975660	-2.052801
H	4.342834	-3.516435	-2.888831
H	5.450601	-2.168748	-2.474850
H	5.498929	-3.686816	-1.543019
C	-0.553604	-1.193742	3.154036
C	-1.333695	-0.084253	2.757111
H	-0.827766	-2.202787	2.816381
H	0.098261	-1.153933	4.033760
H	-2.232149	-0.231912	2.146436
H	-1.272503	0.866836	3.298613
C	-0.965166	-4.044432	0.027298
H	-0.984204	-4.568579	-0.947640
H	0.031427	-3.580690	0.127318
H	-1.076183	-4.808948	0.816147
C	-5.495071	-2.531365	1.772568
H	-5.230512	-2.812049	2.808620
H	-6.148431	-1.643093	1.819243
H	-6.093059	-3.364554	1.356146

[2]II-Coor-C

Zero-point correction= 0.647699 (Hartree/Particle)

Thermal correction to Energy= 0.688563

Thermal correction to Enthalpy= 0.689508

Thermal correction to Gibbs Free Energy= 0.573765

Sum of electronic and zero-point Energies= -1943.760748

Sum of electronic and thermal Energies= -1943.719884

Sum of electronic and thermal Enthalpies= -1943.718939

Sum of electronic and thermal Free Energies= -1943.834682

E solvent= -1943.66900804

C	-0.265599	2.702237	0.991669
C	0.999609	3.325046	1.058073
H	1.824284	2.939516	0.448843
C	1.201200	4.438143	1.890011
H	2.188708	4.912418	1.927298

C	0.145674	4.946226	2.664149	C	3.066977	0.208280	-1.749846
H	0.304987	5.816681	3.310630	C	1.870684	0.208598	-0.931951
C	-1.118125	4.335518	2.601737	Ni	0.129613	-0.678012	1.218696
H	-1.950716	4.729759	3.195994	O	1.717821	-0.641716	0.080309
C	-1.322997	3.219566	1.775781	P	-0.467076	1.141807	0.012441
C	-2.070332	1.391586	-0.890539	H	-2.318639	2.763958	1.724977
C	-2.427996	2.713561	-1.248771	C	1.193360	-2.152564	2.138452
H	-1.772925	3.541712	-0.958562	C	2.094865	-1.502723	3.185587
C	-3.612452	2.993470	-1.943075	H	0.650578	-3.022309	2.545664
H	-3.857242	4.027481	-2.210655	H	1.754221	-2.461856	1.242087
C	-4.476144	1.942260	-2.284962	H	2.601715	-0.622744	2.746703
H	-5.406845	2.141337	-2.828355	H	-0.664166	-1.160119	-1.756786
C	-4.138609	0.629237	-1.937431	H	-4.657247	-1.421077	-0.095152
H	-4.799257	-0.196524	-2.224724	C	4.262855	-0.717676	-1.431090
C	-2.940569	0.318075	-1.248786	C	3.831053	-2.204737	-1.525358
C	-2.680108	-1.119448	-0.945039	H	4.687034	-2.866676	-1.292716
C	-3.707173	-1.900571	-0.362964	H	3.019868	-2.419338	-0.812772
C	-3.526018	-3.268865	-0.098088	H	3.478960	-2.447638	-2.544920
C	-2.288072	-3.858880	-0.433324	C	4.780068	-0.414468	0.001849
C	-1.249679	-3.115119	-1.020533	H	5.609406	-1.099524	0.262038
C	-1.452345	-1.737249	-1.259272	H	5.160824	0.621648	0.068391
C	0.849730	1.162629	-1.233588	H	3.973427	-0.540310	0.740995
C	0.931527	2.043200	-2.330486	C	5.443473	-0.511653	-2.409749
H	0.112758	2.742261	-2.538377	H	5.167775	-0.753862	-3.452801
C	2.061720	2.002292	-3.146129	H	5.830575	0.523599	-2.383353
H	2.150735	2.664456	-4.014113	H	6.272818	-1.183358	-2.122295
C	3.103375	1.101996	-2.832406	H	1.495058	-1.126312	4.039824
H	3.988600	1.111176	-3.476260	C	3.163564	-2.483664	3.723346

H	3.806631	-1.998420	4.481440
H	2.693507	-3.368101	4.192551
H	3.814557	-2.843221	2.905978
C	-4.635643	-4.098890	0.517763
H	-5.086880	-4.782630	-0.227050
H	-4.260449	-4.728516	1.345118
H	-5.444638	-3.462526	0.916255
C	0.060749	-3.766042	-1.406146
H	0.152703	-3.861909	-2.505179
H	0.912361	-3.155277	-1.059404
H	0.154831	-4.776979	-0.973162
H	-2.132435	-4.928042	-0.235239
C	-1.169831	-1.469525	2.614891
C	-1.596219	-0.185483	2.252529
H	-1.626013	-2.351838	2.150559
H	-0.686957	-1.625378	3.583405
H	-2.433840	-0.061066	1.557370
H	-1.369698	0.674329	2.895354

[2]TS-II$_{\mathbf{Ins}}$

Zero-point correction= 0.647924 (Hartree/Particle)

Thermal correction to Energy= 0.688011

Thermal correction to Enthalpy= 0.688955

Thermal correction to Gibbs Free Energy= 0.574953

Sum of electronic and zero-point Energies= -1943.755043

Sum of electronic and thermal Energies= -1943.714956

Sum of electronic and thermal Enthalpies= -1943.714012

Sum of electronic and thermal Free Energies= -1943.828014

E solvent= -1943.66315057

C	-0.190944	2.743161	0.853982
C	1.061191	3.390175	0.785279
H	1.841743	2.987375	0.130702
C	1.305171	4.546935	1.543683
H	2.282068	5.039856	1.477781
C	0.304738	5.073849	2.375864
H	0.496911	5.977516	2.965410
C	-0.947288	4.439043	2.445573
H	-1.737092	4.847632	3.086754
C	-1.193746	3.280168	1.694146
C	-2.072881	1.375905	-0.907614
C	-2.413497	2.693084	-1.298571
H	-1.737569	3.516925	-1.046662
C	-3.605504	2.975015	-1.978988
H	-3.836079	4.005231	-2.272639
C	-4.494289	1.930730	-2.273504
H	-5.431466	2.130791	-2.805337
C	-4.172846	0.622335	-1.894419
H	-4.853132	-0.199057	-2.146120
C	-2.967976	0.308323	-1.219369
C	-2.727934	-1.125134	-0.884570
C	-3.759131	-1.873990	-0.268783

C	-3.595741	-3.237326	0.030408
C	-2.372437	-3.855802	-0.305476
C	-1.331888	-3.144986	-0.928925
C	-1.516551	-1.771546	-1.202492
C	0.840903	1.034249	-1.302984
C	0.911810	1.825160	-2.466508
H	0.101673	2.524465	-2.706798
C	2.019666	1.697658	-3.304502
H	2.099772	2.290114	-4.222324
C	3.050909	0.801476	-2.947524
H	3.919344	0.741488	-3.611114
C	3.024501	-0.006014	-1.798866
C	1.851660	0.078904	-0.952490
Ni	0.091194	-0.524407	1.253978
O	1.695119	-0.683936	0.124060
P	-0.453709	1.137617	-0.033478
H	-2.178740	2.801926	1.747429
C	1.073978	-1.973452	2.489273
H	0.782574	-2.866490	3.064138
C	2.117384	-1.150436	3.244755
H	2.446449	-0.299476	2.621168
H	1.441133	-2.322842	1.508552
C	-1.505202	-0.250836	2.339777
C	-0.870519	-1.400303	2.933942
H	-1.452243	0.710124	2.870586
H	-2.410119	-0.405109	1.738654
H	-0.521971	-1.298063	3.969670
H	-1.323964	-2.375186	2.715659

H	1.670853	-0.719025	4.163878
H	-0.725527	-1.218911	-1.722577
H	-4.697431	-1.372170	-0.000140
C	3.344655	-2.002796	3.635675
H	3.845032	-2.411019	2.739414
H	4.085817	-1.399129	4.191212
H	3.052163	-2.854601	4.278079
H	-2.230932	-4.921851	-0.081054
C	-4.709895	-4.032282	0.683295
H	-4.332815	-4.649176	1.519414
H	-5.500731	-3.371935	1.079292
H	-5.185704	-4.724925	-0.037687
C	-0.039709	-3.829201	-1.320759
H	0.014397	-3.986500	-2.415347
H	0.830621	-3.211663	-1.038285
H	0.059101	-4.817172	-0.838053
C	4.212500	-0.926091	-1.435153
C	3.753944	-2.407449	-1.383514
H	4.606788	-3.062736	-1.121713
H	2.964283	-2.541319	-0.627910
H	3.362681	-2.733001	-2.365052
C	4.773676	-0.509728	-0.048109
H	5.600728	-1.182733	0.249236
H	5.169270	0.522460	-0.079350
H	3.984560	-0.559351	0.718761
C	5.368326	-0.828384	-2.459138
H	5.057908	-1.148815	-3.470846
H	5.776284	0.196720	-2.529220

H	6.192382	-1.492228	-2.139612

[2]II-β-T

Zero-point correction= 0.648360 (Hartree/Particle)

Thermal correction to Energy= 0.688800

Thermal correction to Enthalpy= 0.689744

Thermal correction to Gibbs Free Energy= 0.573808

Sum of electronic and zero-point Energies= -1943.792300

Sum of electronic and thermal Energies= -1943.751860

Sum of electronic and thermal Enthalpies= -1943.750916

Sum of electronic and thermal Free Energies= -1943.866852

E solvent= -1943.69908573

C	0.361124	2.256664	1.648035
C	1.613676	2.415639	2.278196
H	2.507687	1.976470	1.822432
C	1.715225	3.140600	3.476900
H	2.694661	3.261025	3.954234
C	0.572658	3.712396	4.058971
H	0.655250	4.278522	4.993745
C	-0.678385	3.556768	3.436957
H	-1.574548	4.002762	3.883972
C	-0.786292	2.830938	2.241696
C	-0.782527	2.255890	-1.025675
C	-0.537229	3.649643	-1.003471
H	0.179486	4.055376	-0.281174
C	-1.208150	4.526910	-1.864986
H	-0.991588	5.600493	-1.829681
C	-2.158937	4.018023	-2.763027
H	-2.692442	4.688824	-3.446094
C	-2.422406	2.643732	-2.788391
H	-3.153332	2.242714	-3.499530
C	-1.747783	1.733549	-1.938036
C	-2.119281	0.293446	-2.036816
C	-3.487014	-0.063872	-2.024624
C	-3.897140	-1.405233	-2.125685
C	-2.905699	-2.401597	-2.248398
C	-1.536249	-2.083199	-2.284437
C	-1.149606	-0.728005	-2.153867
C	1.863142	0.972257	-0.553079
C	2.678751	1.999700	-1.069821
H	2.294407	3.024292	-1.143434
C	3.974082	1.694848	-1.487885
H	4.626313	2.471751	-1.901419
C	4.447477	0.370821	-1.352704
H	5.479028	0.169134	-1.658504
C	3.674051	-0.685649	-0.847097
C	2.305834	-0.392413	-0.475927
Ni	-0.286653	-0.828981	0.510142
O	1.473171	-1.342957	-0.062627
P	0.193554	1.201167	0.141421
H	-1.765447	2.721196	1.761012

C	-1.983784	-0.739024	1.374883
C	-1.695721	-2.209158	1.576468
H	-1.971524	-0.124138	2.290261
H	-2.826000	-0.495799	0.709878
H	-2.420164	-2.845612	1.032987
H	-0.089431	-0.463997	-2.244264
H	-4.242853	0.722738	-1.906135
C	4.262809	-2.102453	-0.656743
C	3.483406	-3.133902	-1.514466
H	3.920833	-4.142959	-1.388737
H	2.427179	-3.168855	-1.207560
H	3.531288	-2.871943	-2.587577
C	4.162338	-2.497721	0.842696
H	4.542291	-3.526542	0.992838
H	4.768159	-1.816123	1.468084
H	3.116141	-2.453300	1.185856
C	5.752408	-2.178814	-1.067993
H	5.900750	-1.956111	-2.140967
H	6.382651	-1.486878	-0.479458
H	6.126089	-3.203225	-0.887558
H	-0.705339	-2.507122	1.028999
C	-1.493070	-2.679676	3.033243
H	-1.032595	-3.687628	3.041360
H	-0.771366	-2.000126	3.528099
C	-2.809842	-2.712656	3.832127
C	-5.367930	-1.773960	-2.122277
H	-5.723876	-2.014558	-3.142994
H	-5.562433	-2.663754	-1.496364
H	-5.992805	-0.947414	-1.741726
C	-0.488438	-3.157968	-2.483461
H	-0.888602	-4.161826	-2.256105
H	-0.128107	-3.174914	-3.530333
H	0.387875	-2.970551	-1.839277
H	-3.210871	-3.453514	-2.334749
C	-2.610328	-3.160942	5.289232
H	-3.273740	-1.707315	3.808843
H	-3.524192	-3.393881	3.327053
H	-3.567782	-3.179948	5.840044
H	-2.173512	-4.175684	5.339864
H	-1.926965	-2.477469	5.826573

[2]TS-II$_{Decoor}$

Zero-point correction= 0.643570 (Hartree/Particle)

Thermal correction to Energy= 0.684700

Thermal correction to Enthalpy= 0.685644

Thermal correction to Gibbs Free Energy= 0.570084

Sum of electronic and zero-point Energies= -1943.741974

Sum of electronic and thermal Energies= -1943.700844

Sum of electronic and thermal Enthalpies= -1943.699900

Sum of electronic and thermal Free Energies= -1943.815459

E solvent= -1943.65125721

C	0.817040	2.757593	0.451072
C	2.181703	3.065837	0.271262
H	2.818789	2.382868	-0.301402
C	2.721308	4.249404	0.806226
H	3.783378	4.475087	0.654974
C	1.907772	5.139338	1.523408
H	2.329915	6.061954	1.937703
C	0.545754	4.840675	1.708580
H	-0.098049	5.531032	2.266222
C	0.006601	3.657114	1.183858
C	-1.252502	1.761554	-1.287115
C	-1.076385	3.030057	-1.890516
H	-0.172944	3.606423	-1.666898
C	-2.036826	3.584845	-2.746878
H	-1.861429	4.567374	-3.199448
C	-3.216574	2.874925	-3.010422
H	-3.982294	3.291130	-3.675029
C	-3.405524	1.616116	-2.429172
H	-4.310260	1.042779	-2.660451
C	-2.442073	1.026430	-1.573458
C	-2.751917	-0.344990	-1.072350
C	-4.041869	-0.621874	-0.558681
C	-4.424903	-1.925204	-0.198749
C	-3.486729	-2.968120	-0.359905
C	-2.195555	-2.728349	-0.863625
C	-1.832533	-1.406227	-1.201527
C	1.364799	0.373278	-1.171730
C	1.830508	0.893477	-2.398446
H	1.356597	1.785345	-2.826804
C	2.886671	0.265468	-3.057658
H	3.256652	0.648225	-4.015072
C	3.485516	-0.869874	-2.468674
H	4.328881	-1.328672	-2.994719
C	3.058073	-1.433003	-1.256947
C	1.924060	-0.817187	-0.595812
Ni	-0.027917	-0.487254	1.406519
O	1.425986	-1.351383	0.515620
P	0.085115	1.155439	-0.137421
H	-1.057728	3.434936	1.331776
C	-0.528705	-2.051318	2.441420
H	-0.516680	-2.911045	1.760194
C	-1.775902	-1.254433	2.545327
H	-1.569718	-0.150123	2.107911
H	0.067190	-2.189868	3.355780
C	1.641115	0.360970	3.045853
C	0.718005	1.099537	3.707984
H	1.960946	-0.624397	3.402313
H	2.210597	0.778670	2.208623
H	0.209614	0.718857	4.601338
H	0.454162	2.111195	3.381235
H	-2.557984	-1.608926	1.853046
H	-0.841455	-1.216788	-1.629495
H	-4.754726	0.203058	-0.434107
C	-2.334045	-0.972127	3.951941
H	-1.543053	-0.612762	4.631696

H	-3.139112	-0.216127	3.928405
H	-2.748808	-1.903632	4.376057
H	-3.773445	-3.993874	-0.090655
C	3.776648	-2.653154	-0.635584
C	2.794447	-3.846962	-0.496509
H	3.312867	-4.717142	-0.049963
H	1.945038	-3.573405	0.148883
H	2.406077	-4.153289	-1.485515
C	4.324853	-2.270497	0.766682
H	4.816878	-3.143964	1.235897
H	5.073472	-1.460526	0.686988
H	3.507534	-1.932245	1.422936
C	4.972241	-3.128917	-1.493660
H	4.656229	-3.462798	-2.499283
H	5.738531	-2.341085	-1.614071
H	5.456455	-3.989134	-0.996293
C	-5.816221	-2.213092	0.331598
H	-5.786112	-2.884263	1.209269
H	-6.335954	-1.286137	0.630023
H	-6.442365	-2.712308	-0.433062
C	-1.212981	-3.862259	-1.069372
H	-1.128609	-4.127086	-2.141208
H	-0.202922	-3.578037	-0.726466
H	-1.524291	-4.772883	-0.528036

^{2}I-β-C

Zero-point correction= 0.591675 (Hartree/Particle)

Thermal correction to Energy= 0.629466

Thermal correction to Enthalpy= 0.630410

Thermal correction to Gibbs Free Energy= 0.520109

Sum of electronic and zero-point Energies= -1865.218078

Sum of electronic and thermal Energies= -1865.180287

Sum of electronic and thermal Enthalpies= -1865.179343

Sum of electronic and thermal Free Energies= -1865.289644

E solvent= -1865.10400363

C	0.114804	2.705234	0.845623
C	1.382693	3.315494	0.940243
H	2.236373	2.871355	0.417036
C	1.549625	4.489700	1.694251
H	2.540269	4.954953	1.757366
C	0.456676	5.067428	2.358629
H	0.589297	5.983849	2.944920
C	-0.810280	4.463884	2.269964
H	-1.669134	4.909279	2.785783
C	-0.979844	3.288993	1.523884
C	-1.451331	1.511244	-1.273813
C	-1.477822	2.810969	-1.831791
H	-0.719630	3.540005	-1.524849
C	-2.468806	3.197281	-2.744019
H	-2.459742	4.208640	-3.165893

C	-3.472324	2.283573	-3.101595	H	0.748249	-1.892815	3.351103
H	-4.256132	2.569635	-3.812277	H	-2.210127	-2.034855	2.538798
C	-3.467586	0.997255	-2.548983	H	-0.510868	-1.364392	-1.625717
H	-4.240482	0.275956	-2.838010	H	-4.627690	-0.596495	-0.520538
C	-2.466664	0.578539	-1.639198	C	-1.563501	-0.639644	4.136139
C	-2.551026	-0.815563	-1.114110	H	-0.693863	0.000806	4.362653
C	-3.776058	-1.285417	-0.587245	H	-2.463178	0.000164	4.101833
C	-3.917168	-2.606775	-0.126678	H	-1.685335	-1.360459	4.963921
C	-2.802231	-3.468331	-0.206758	H	-2.899517	-4.504803	0.143943
C	-1.571296	-3.039192	-0.736748	C	4.368676	-1.698871	-0.349350
C	-1.451317	-1.700035	-1.171878	C	3.659455	-3.077477	-0.432091
C	1.413270	0.776752	-0.962752	H	4.303099	-3.864133	0.006253
C	1.882056	1.520844	-2.064824	H	2.706689	-3.053751	0.119644
H	1.267970	2.329590	-2.478972	H	3.454334	-3.351744	-1.483597
C	3.122849	1.212108	-2.622506	C	4.662602	-1.355678	1.137421
H	3.503923	1.769270	-3.485253	H	5.273065	-2.155096	1.599609
C	3.892504	0.174179	-2.053818	H	5.227689	-0.408307	1.214847
H	4.871486	-0.033521	-2.497112	H	3.724911	-1.253397	1.706677
C	3.471015	-0.598265	-0.960177	C	5.724829	-1.822598	-1.082829
C	2.164685	-0.311564	-0.409427	H	5.599835	-2.103172	-2.145123
Ni	-0.030215	-0.690641	1.262623	H	6.309186	-0.885167	-1.037276
O	1.682264	-1.047604	0.594746	H	6.327612	-2.612963	-0.599448
P	-0.125749	1.099823	-0.046738	C	-5.242766	-3.103695	0.417604
H	-1.972750	2.826765	1.460007	H	-5.815978	-3.648585	-0.357738
C	-0.047172	-2.069563	2.613160	H	-5.100423	-3.801605	1.262201
H	-0.036734	-3.067397	2.155762	H	-5.876354	-2.269891	0.767305
C	-1.349658	-1.399487	2.816655	C	-0.397588	-3.988081	-0.860390
H	-1.518259	-0.527100	1.966648	H	-0.251263	-4.307481	-1.910337

H	0.537875	-3.501074	-0.533764
H	-0.548062	-4.900069	-0.256292

[2]TS-I_{BHE-C}

Zero-point correction= 0.588719 (Hartree/Particle)

Thermal correction to Energy= 0.626306

Thermal correction to Enthalpy= 0.627250

Thermal correction to Gibbs Free Energy= 0.518286

Sum of electronic and zero-point Energies= -1865.215087

Sum of electronic and thermal Energies= -1865.177501

Sum of electronic and thermal Enthalpies= -1865.176557

Sum of electronic and thermal Free Energies= -1865.285520

E solvent= -1865.09858473

C	0.197079	2.711509	0.763171
C	1.442583	3.361899	0.646369
H	2.222808	2.925917	0.013438
C	1.679808	4.563906	1.335382
H	2.651990	5.060649	1.236536
C	0.680010	5.127012	2.143130
H	0.867772	6.064396	2.679099
C	-0.563649	4.481855	2.265669
H	-1.348282	4.914709	2.897164
C	-0.802145	3.279183	1.586103
C	-1.528957	1.491956	-1.223029
C	-1.586668	2.795931	-1.768117
H	-0.817763	3.525538	-1.491274
C	-2.621834	3.184717	-2.628980
H	-2.637571	4.199760	-3.041501
C	-3.636601	2.268829	-2.945504
H	-4.455477	2.556816	-3.614646
C	-3.597320	0.976855	-2.407572
H	-4.377836	0.253297	-2.669005
C	-2.551909	0.553777	-1.551844
C	-2.596439	-0.847581	-1.044997
C	-3.795001	-1.347607	-0.485335
C	-3.892849	-2.674505	-0.032798
C	-2.762517	-3.511334	-0.154986
C	-1.559138	-3.050717	-0.717965
C	-1.482552	-1.707045	-1.146976
C	1.339882	0.689567	-1.053518
C	1.761639	1.349447	-2.226053
H	1.133719	2.129606	-2.674179
C	2.979471	0.991289	-2.805557
H	3.328049	1.478413	-3.722690
C	3.770824	-0.001175	-2.185915
H	4.734088	-0.242110	-2.646526
C	3.390859	-0.688641	-1.022419
C	2.099609	-0.368652	-0.452607
Ni	-0.088063	-0.578727	1.309345
O	1.627547	-1.032229	0.600042
P	-0.135149	1.094532	-0.073821

H	-1.772208	2.778040	1.691250
C	0.042163	-2.023810	2.692172
H	-0.007929	-2.965846	2.134624
C	-1.138321	-1.305846	2.998043
H	-1.443284	-0.132717	1.759879
H	0.973641	-1.825309	3.236887
H	-2.090583	-1.774926	2.710063
H	-0.562512	-1.347567	-1.623715
H	-4.657598	-0.677071	-0.382980
C	-1.212876	-0.363878	4.191164
H	-0.246127	0.138135	4.365307
H	-1.985297	0.413292	4.059691
H	-1.466835	-0.943248	5.099873
H	-2.826567	-4.552227	0.190401
C	4.316488	-1.731564	-0.355443
C	3.624818	-3.120656	-0.318484
H	4.287534	-3.864041	0.164904
H	2.683485	-3.065092	0.250163
H	3.401805	-3.476961	-1.341268
C	4.636010	-1.275537	1.095235
H	5.269722	-2.028904	1.601346
H	5.184801	-0.315560	1.092040
H	3.707823	-1.147047	1.674893
C	5.657871	-1.892252	-1.108217
H	5.514236	-2.257348	-2.142085
H	6.227354	-0.945521	-1.150777
H	6.284492	-2.633347	-0.579269
C	-5.187142	-3.205357	0.553066
H	-5.714834	-3.860928	-0.166653
H	-5.006043	-3.807798	1.461863
H	-5.877184	-2.386293	0.820363
C	-0.364091	-3.966715	-0.877782
H	-0.206570	-4.238534	-1.939344
H	0.558431	-3.469443	-0.529631
H	-0.493275	-4.905688	-0.311334

^{2}I-BHE-C

Zero-point correction= 0.589412 (Hartree/Particle)

Thermal correction to Energy= 0.627514

Thermal correction to Enthalpy= 0.628459

Thermal correction to Gibbs Free Energy= 0.517432

Sum of electronic and zero-point Energies= -1865.215182

Sum of electronic and thermal Energies= -1865.177080

Sum of electronic and thermal Enthalpies= -1865.176136

Sum of electronic and thermal Free Energies= -1865.287163

E solvent = -1865.09898489

C	0.175757	2.715903	0.703885
C	1.391238	3.394518	0.480325
H	2.131337	2.966736	-0.204264
C	1.649351	4.612745	1.132438
H	2.598017	5.131699	0.952622

C	0.700178	5.163001	2.007317	C	3.311661	-0.693312	-1.140295
H	0.904638	6.112809	2.514510	C	2.039467	-0.373000	-0.527891
C	-0.513387	4.489310	2.235031	Ni	-0.103346	-0.518204	1.330605
H	-1.257573	4.912492	2.919710	O	1.607304	-1.010692	0.555937
C	-0.772543	3.270606	1.592993	P	-0.183359	1.087566	-0.095416
C	-1.633829	1.446422	-1.182518	H	-1.715789	2.743708	1.781448
C	-1.729747	2.741713	-1.742410	C	0.171495	-2.053824	2.677750
H	-0.959570	3.484915	-1.508418	H	-0.220225	-2.939280	2.163534
C	-2.804517	3.105036	-2.565014	C	-0.691376	-1.146386	3.288306
H	-2.850933	4.113834	-2.990232	H	-1.429656	0.070072	1.658016
C	-3.818982	2.171650	-2.825915	H	1.251988	-2.021920	2.857278
H	-4.668740	2.439390	-3.464244	H	-1.765381	-1.373103	3.282777
C	-3.739960	0.887648	-2.273374	H	-0.633072	-1.375260	-1.576500
H	-4.520430	0.150719	-2.494312	H	-4.697846	-0.769829	-0.208042
C	-2.654876	0.488918	-1.456109	C	-0.239006	-0.113089	4.298780
C	-2.655932	-0.907056	-0.934559	H	0.838194	0.104438	4.200548
C	-3.827533	-1.425737	-0.335553	H	-0.793911	0.834582	4.191932
C	-3.887149	-2.750991	0.125876	H	-0.420382	-0.490545	5.325617
C	-2.746712	-3.568740	-0.028866	H	-2.782050	-4.609298	0.321728
C	-1.569810	-3.089193	-0.629443	C	4.280004	-1.701658	-0.480969
C	-1.530522	-1.746473	-1.066816	C	3.615579	-3.100421	-0.376332
C	1.248830	0.663292	-1.127411	H	4.308490	-3.816596	0.105816
C	1.611269	1.292780	-2.335744	H	2.693648	-3.043913	0.223015
H	0.956457	2.053942	-2.778046	H	3.363553	-3.492418	-1.379088
C	2.807364	0.927986	-2.955072	C	4.646438	-1.196880	0.942138
H	3.112565	1.390284	-3.900055	H	5.307894	-1.926192	1.447903
C	3.634624	-0.037089	-2.338699	H	5.181233	-0.230551	0.889660
H	4.580597	-0.281944	-2.831871	H	3.737845	-1.061877	1.550640

C	5.594677	-1.861562	-1.279979
H	5.418988	-2.259952	-2.296383
H	6.144180	-0.906551	-1.372012
H	6.254915	-2.575226	-0.754154
C	-5.149097	-3.299628	0.763161
C	-0.362860	-3.982963	-0.821433
H	-0.219461	-4.239902	-1.888623
H	0.556257	-3.472401	-0.483869
H	-0.465162	-4.930039	-0.263078
H	-4.931462	-3.818084	1.715089
H	-5.877818	-2.497819	0.973638
H	-5.646239	-4.036326	0.102904

^{2}TS-I$_{Transf-1}$

Zero-point correction= 0.640580 (Hartree/Particle)

Thermal correction to Energy= 0.682535

Thermal correction to Enthalpy= 0.683479

Thermal correction to Gibbs Free Energy= 0.565081

Sum of electronic and zero-point Energies= -1943.735395

Sum of electronic and thermal Energies= -1943.693441

Sum of electronic and thermal Enthalpies= -1943.692496

Sum of electronic and thermal Free Energies= -1943.810894

E solvent= -1943.64047027

C	1.767696	2.314732	0.011622
C	2.874305	2.234488	-0.860204
H	2.873874	1.495590	-1.669146
C	3.976021	3.090719	-0.687153
H	4.831993	3.015014	-1.367832
C	3.982046	4.037124	0.349752
H	4.843070	4.701881	0.483682
C	2.879604	4.126190	1.219143
H	2.878641	4.861070	2.032638
C	1.783035	3.267625	1.056568
C	-0.994962	2.246846	-0.884999
C	-0.581537	3.493865	-1.412484
H	0.480033	3.756304	-1.398910
C	-1.494739	4.416940	-1.940156
H	-1.133071	5.369373	-2.343620
C	-2.861700	4.111501	-1.938113
H	-3.594351	4.821327	-2.338877
C	-3.287880	2.877340	-1.433727
H	-4.351942	2.617312	-1.463869
C	-2.385187	1.918308	-0.913020
C	-2.990005	0.624120	-0.475530
C	-4.123253	0.641385	0.372239
C	-4.810159	-0.540183	0.700190
C	-4.356190	-1.755982	0.147967
C	-3.237530	-1.807497	-0.703156
C	-2.554737	-0.609723	-0.999965
C	0.800491	-0.100881	-1.360964
C	0.751909	0.059618	-2.760893

H	0.397509	1.004234	-3.192279
C	1.145882	-1.000114	-3.580421
H	1.116584	-0.904707	-4.671255
C	1.584855	-2.203734	-2.986326
H	1.885735	-3.017396	-3.654081
C	1.650261	-2.410591	-1.598638
C	1.230421	-1.322477	-0.739227
Ni	0.562602	-0.009338	1.706817
O	1.225074	-1.443560	0.582421
P	0.324858	1.158942	-0.140804
H	0.934565	3.325366	1.748349
C	1.396158	-0.861321	3.424407
H	0.626353	-0.752430	4.193911
C	2.200918	0.219248	3.068083
H	-0.076176	1.112313	2.450168
H	1.654228	-1.879605	3.111430
H	2.017751	1.181741	3.564397
H	-1.701367	-0.636468	-1.685197
H	-4.460489	1.596781	0.793578
C	3.512889	0.098495	2.332677
H	3.559297	-0.828894	1.740991
H	3.686039	0.956088	1.660883
H	4.342127	0.088763	3.070667
H	-4.890763	-2.685195	0.387498
C	2.144374	-3.748565	-1.001557
C	1.028410	-4.387843	-0.130924
H	1.400946	-5.315633	0.344255
H	0.701764	-3.690960	0.656905
H	0.153309	-4.655585	-0.751760
C	3.399823	-3.493965	-0.124413
H	3.752720	-4.442669	0.323200
H	4.224367	-3.074128	-0.729709
H	3.164583	-2.788708	0.687933
C	2.533436	-4.772713	-2.093363
H	1.678475	-5.031830	-2.744987
H	3.359038	-4.408735	-2.732481
H	2.875363	-5.706192	-1.610129
C	-1.736200	-2.317809	2.928113
C	-1.990609	-0.997887	2.980641
H	-1.344731	-2.870495	3.791326
H	-1.897943	-2.892606	2.009758
H	-1.838782	-0.415738	3.897618
H	-2.393986	-0.454279	2.120205
C	-5.987668	-0.518178	1.654019
H	-6.794947	-1.193741	1.317843
H	-5.683867	-0.852541	2.664747
H	-6.410520	0.496636	1.754316
C	-2.793004	-3.113173	-1.332327
H	-3.111203	-3.982571	-0.730317
H	-3.228157	-3.237466	-2.343196
H	-1.695419	-3.150848	-1.444004

^{2}I-Transf-1

Zero-point correction= 0.642074 (Hartree/Particle)

Thermal correction to Energy= 0.683660

Thermal correction to Enthalpy= 0.684605

Thermal correction to Gibbs Free Energy= 0.568490

Sum of electronic and zero-point Energies= -1943.572458

Sum of electronic and thermal Energies= -1943.530872

Sum of electronic and thermal Enthalpies= -1943.529928

Sum of electronic and thermal Free Energies= -1.943.646043

E solvent= -1.943.077553

C	-2.891330	-0.723979	-0.242951
C	-3.576687	-0.037830	-1.267576
H	-3.008098	0.515715	-2.022523
C	-4.981908	-0.050481	-1.315557
H	-5.500739	0.492464	-2.114207
C	-5.717541	-0.751161	-0.347243
H	-6.812850	-0.758099	-0.385362
C	-5.042491	-1.437707	0.677964
H	-5.609936	-1.982441	1.441590
C	-3.641282	-1.417073	0.734800
C	-0.569444	-2.367126	-0.806972
C	-1.606638	-3.181028	-1.322801
H	-2.627950	-2.792703	-1.351613
C	-1.372712	-4.481161	-1.792265
H	-2.206027	-5.073130	-2.187447
C	-0.077096	-5.009350	-1.745064
H	0.127755	-6.026331	-2.098637
C	0.966212	-4.214671	-1.253953
H	1.989718	-4.606466	-1.244262
C	0.759959	-2.892875	-0.791776
C	2.002544	-2.169153	-0.367637
C	2.821921	-2.733424	0.636131
C	4.074300	-2.178273	0.958901
C	4.516799	-1.055300	0.231963
C	3.734722	-0.481223	-0.788862
C	2.471958	-1.040268	-1.071207
C	-0.448152	0.593574	-1.248196
C	-0.447943	0.476113	-2.654273
H	-0.809334	-0.446897	-3.125614
C	0.035193	1.531797	-3.429174
H	0.049602	1.464718	-4.522411
C	0.510776	2.695025	-2.783408
H	0.881367	3.507929	-3.415883
C	0.536742	2.859061	-1.389507
C	0.048756	1.760831	-0.573578
Ni	-0.522780	0.284733	1.815790
O	0.068857	1.829824	0.747693
P	-1.038837	-0.693494	-0.106915
H	-3.117974	-1.936991	1.546736
C	1.677174	0.223158	2.491888
H	2.193467	0.136317	1.531523
C	1.094877	-0.862354	3.085904
H	-1.102861	-0.935980	2.437448
H	1.741118	1.189313	3.003485
H	1.147292	-1.852025	2.622528
H	1.864425	-0.613589	-1.875674

H	2.470680	-3.624819	1.172073
H	5.498586	-0.620503	0.463691
C	1.069428	4.158405	-0.739971
C	2.281882	3.839566	0.176179
H	2.624273	4.759257	0.688322
H	2.005378	3.091780	0.935543
H	3.128565	3.445843	-0.415497
C	-0.051958	4.818194	0.106992
H	0.318851	5.752565	0.570150
H	-0.925102	5.071368	-0.522255
H	-0.379843	4.136758	0.907096
C	1.531420	5.195192	-1.791114
H	2.359305	4.814339	-2.417289
H	0.706775	5.507715	-2.457951
H	1.898820	6.099008	-1.271442
C	-1.137586	1.505178	3.420560
C	-2.285823	1.265372	2.665447
H	-0.928050	0.918990	4.322057
H	-0.578962	2.440299	3.291040
H	-2.936203	0.434178	2.968807
C	-2.910798	2.272415	1.731175
C	4.912864	-2.759413	2.079771
H	4.736501	-3.843255	2.198799
H	5.991793	-2.605579	1.901285
H	4.669230	-2.280387	3.047989
C	4.249113	0.689068	-1.603333
H	3.425399	1.361392	-1.900388
H	4.993012	1.279143	-1.039485
H	4.741923	0.342568	-2.532824
H	0.693102	-0.817164	4.103936
H	-3.359218	1.790565	0.845946
H	-3.729519	2.800974	2.262056
H	-2.178229	3.019864	1.391185

^{2}TS-III$_{Decoor}$

Zero-point correction= 0.641157 (Hartree/Particle)

Thermal correction to Energy= 0.682506

Thermal correction to Enthalpy= 0.683450

Thermal correction to Gibbs Free Energy= 0.567590

Sum of electronic and zero-point Energies= -1943.736648

Sum of electronic and thermal Energies= -1943.695299

Sum of electronic and thermal Enthalpies= -1943.694355

Sum of electronic and thermal Free Energies= -1943.810215

E solvent= -1943.64479377

C	1.101575	2.680180	0.172095
C	2.311629	2.931653	-0.506996
H	2.671458	2.217042	-1.255623
C	3.050731	4.096639	-0.232209
H	3.990212	4.279168	-0.766845
C	2.589873	5.021276	0.717042

H	3.167740	5.928027	0.929092
C	1.383420	4.777383	1.398592
H	1.017607	5.493799	2.143355
C	0.647998	3.613757	1.132521
C	-1.366115	1.846031	-1.034684
C	-1.168007	3.081498	-1.698765
H	-0.188558	3.565977	-1.649796
C	-2.194522	3.722104	-2.405629
H	-1.995562	4.674787	-2.909290
C	-3.466757	3.137943	-2.451747
H	-4.286939	3.623166	-2.993112
C	-3.679885	1.912296	-1.811225
H	-4.662882	1.432273	-1.873769
C	-2.653669	1.233897	-1.107472
C	-3.034460	-0.093283	-0.537441
C	-4.228596	-0.198773	0.216637
C	-4.714668	-1.443342	0.651398
C	-3.989345	-2.602743	0.304325
C	-2.802636	-2.534546	-0.447262
C	-2.323183	-1.268741	-0.849363
C	1.029397	0.098543	-1.285932
C	1.255433	0.415541	-2.642070
H	0.808920	1.319657	-3.074862
C	2.037955	-0.438373	-3.419822
H	2.224808	-0.220361	-4.476912
C	2.584742	-1.598292	-2.827191
H	3.199963	-2.246236	-3.459569
C	2.374887	-1.966041	-1.489199
C	1.539231	-1.099713	-0.677831
Ni	0.088046	-0.201362	1.600537
O	1.244245	-1.399811	0.578146
P	0.103771	1.141385	-0.116771
H	-0.286273	3.422973	1.674969
C	-0.684416	-1.954940	2.583281
H	-0.768308	-2.612849	1.713808
C	-1.591999	-0.928073	2.785767
H	-0.728655	0.898238	2.182910
H	0.020963	-2.259323	3.362425
H	-2.442576	-0.783926	2.112606
H	-1.409892	-1.204536	-1.452061
H	-4.778645	0.715079	0.474120
H	-4.362580	-3.584648	0.625532
C	2.991649	-3.257607	-0.903204
C	1.861712	-4.227339	-0.464367
H	2.292374	-5.139537	-0.008408
H	1.206492	-3.740624	0.275822
H	1.253027	-4.538140	-1.333640
C	3.871015	-2.913435	0.328279
H	4.299255	-3.837564	0.761299
H	4.707815	-2.250104	0.041663
H	3.265966	-2.407938	1.096852
C	3.882754	-4.002069	-1.925426
H	3.313890	-4.331920	-2.814405
H	4.731129	-3.380873	-2.267480
H	4.303794	-4.906451	-1.449204
C	1.592307	-0.053195	3.605657

C	2.250690	0.859311	2.844749
H	0.746805	0.230673	4.240950
H	1.967991	-1.079036	3.698831
H	1.884954	1.894290	2.822119
C	3.518232	0.580178	2.085159
C	-5.983110	-1.544363	1.476042
H	-6.453613	-0.556073	1.617361
H	-6.726361	-2.206711	0.993935
H	-5.780462	-1.965936	2.478706
C	-2.071198	-3.796947	-0.856489
H	-2.375735	-4.659505	-0.238261
H	-2.284516	-4.055200	-1.912009
H	-0.977629	-3.677436	-0.769228
H	-1.646300	-0.411963	3.751582
H	3.409655	0.826162	1.013643
H	4.342350	1.210336	2.474287
H	3.816508	-0.477424	2.166643

²II-BHE-C

Zero-point correction= 0.561467 (Hartree/Particle)

Thermal correction to Energy= 0.598199

Thermal correction to Enthalpy= 0.599143

Thermal correction to Gibbs Free Energy= 0.491451

Sum of electronic and zero-point Energies= -1825.928731

Sum of electronic and thermal Energies= -1825.891999

Sum of electronic and thermal Enthalpies= -1825.891055

Sum of electronic and thermal Free Energies= -1825.998748

E solvent= -1825.80209063

C	0.189987	2.700459	0.864716
C	1.381106	3.410190	0.607739
H	2.102420	3.013375	-0.114623
C	1.640844	4.618571	1.276913
H	2.570617	5.161489	1.071163
C	0.718232	5.127443	2.203765
H	0.924550	6.068970	2.725426
C	-0.469667	4.421954	2.466520
H	-1.191615	4.811222	3.193749
C	-0.730580	3.213181	1.806488
C	-1.612949	1.468073	-1.054692
C	-1.693081	2.769014	-1.603803
H	-0.918009	3.503167	-1.357475
C	-2.758575	3.149029	-2.430934
H	-2.792562	4.161717	-2.847993
C	-3.780120	2.227666	-2.706663
H	-4.622483	2.508778	-3.348996
C	-3.718592	0.939310	-2.161966
H	-4.505949	0.212333	-2.391367
C	-2.643263	0.524248	-1.340242
C	-2.666743	-0.872064	-0.819167
C	-3.842921	-1.366491	-0.209977
C	-3.921617	-2.687554	0.262929

C	-2.794171	-3.523469	0.112112
C	-1.613154	-3.068197	-0.500586
C	-1.555819	-1.730902	-0.951651
C	1.260645	0.668198	-0.993641
C	1.630498	1.306466	-2.195180
H	0.980268	2.073649	-2.633734
C	2.827633	0.942132	-2.812726
H	3.138700	1.411657	-3.752188
C	3.647379	-0.033480	-2.202877
H	4.593984	-0.278582	-2.694694
C	3.316342	-0.699646	-1.012263
C	2.044546	-0.376411	-0.401341
Ni	-0.092153	-0.538459	1.446494
O	1.608386	-1.022708	0.676056
P	-0.172557	1.086921	0.037851
H	-1.652312	2.660321	2.024564
C	0.236021	-1.979996	2.860818
H	0.297996	-2.910496	2.286566
C	-0.994904	-1.382319	3.136762
H	-1.407911	0.065655	1.792442
H	1.152650	-1.656472	3.366343
H	-1.930311	-1.863758	2.828648
H	-0.655170	-1.376832	-1.467736
H	-4.701708	-0.695378	-0.083162
H	-1.084434	-0.623285	3.922844
H	-2.842670	-4.559171	0.475093
C	4.273018	-1.724424	-0.361556
C	3.596157	-3.119146	-0.284486
H	4.279688	-3.849268	0.189962
H	2.671213	-3.064716	0.310594
H	3.346732	-3.492147	-1.295115
C	4.633937	-1.246060	1.072023
H	5.288498	-1.987928	1.568363
H	5.174839	-0.282269	1.039022
H	3.722995	-1.116215	1.678033
C	5.591280	-1.882091	-1.154996
H	5.419157	-2.265115	-2.177914
H	6.147516	-0.929544	-1.230382
H	6.243496	-2.607412	-0.635224
C	-5.193215	-3.212325	0.901357
C	-0.420897	-3.982751	-0.688214
H	-0.295894	-4.266441	-1.750944
H	0.510802	-3.478598	-0.375580
H	-0.528798	-4.915280	-0.106967
H	-4.977902	-3.816746	1.801170
H	-5.867050	-2.389845	1.197882
H	-5.753911	-3.863794	0.203116

Catalyst 3

^{3}I-β-T

Zero-point correction= 0.603224 (Hartree/Particle)

Thermal correction to Energy= 0.642373

Thermal correction to Enthalpy= 0.643317

Thermal correction to Gibbs Free Energy=

0.531850

Sum of electronic and zero-point Energies= -2015.631322

Sum of electronic and thermal Energies= -2015.592173

Sum of electronic and thermal Enthalpies= -2015.591229

Sum of electronic and thermal Free Energies= -2015.702696

E solvent= -2.015.520268880

C 0.763428000 2.708820000 0.761001000

C 2.126416000 3.057009000 0.863444000

H 2.881822000 2.437149000 0.368732000

C 2.512256000 4.195659000 1.590455000

H 3.574417000 4.457377000 1.660815000

C 1.546250000 4.996665000 2.219510000

H 1.850430000 5.884468000 2.785558000

C 0.185747000 4.655926000 2.119485000

H -0.574707000 5.278275000 2.605503000

C -0.204466000 3.517965000 1.399025000

C -1.040265000 1.733797000 -1.293686000

C -0.828893000 3.001972000 -1.886793000

H 0.044361000 3.592472000 -1.589160000

C -1.724564000 3.533547000 -2.823664000

H -1.529472000 4.514703000 -3.270808000

C -2.870499000 2.802973000 -3.172114000

H -3.583057000 3.201827000 -3.903128000

C -3.101002000 1.554192000 -2.583318000

H -3.988421000 0.975165000 -2.862636000

C -2.202666000 0.987532000 -1.646262000

C -2.562831000 -0.344585000 -1.077168000

C -3.855440000 -0.505403000 -0.530040000

C -4.246209000 -1.762759000 -0.023937000

C -3.362357000 -2.853469000 -0.075707000

C -2.082383000 -2.693376000 -0.636191000

C -1.665078000 -1.431469000 -1.125502000

C 1.684112000 0.552698000 -1.031159000

C 2.233705000 1.165067000 -

2.175851000

H 1.728966000 2.021003000 -2.640324000

C 3.421499000 0.664306000 -2.711025000

H 3.862717000 1.112215000 -3.608073000

C 4.068698000 -0.412777000 -2.066264000

H 5.020155000 -0.755389000 -2.485705000

C 3.562247000 -1.054165000 -0.924406000

C 2.285429000 -0.599326000 -0.418312000

Ni 0.037516000 -0.466491000 1.261100000

O 1.674566000 -1.212932000 0.591914000

P 0.242258000 1.150841000 -0.089693000

H -1.267803000 3.261608000 1.323449000

C 4.351164000 -2.171617000 -0.202907000

C 3.544001000 -3.496730000 -0.188396000

H 4.128606000 -4.292107000 0.312299000

H 2.595100000 -3.364762000 0.353391000

H 3.323838000 -3.835262000 -1.218135000

C 4.630960000 -1.728484000 1.260132000

H 5.169358000 -2.527523000 1.804891000

H 5.259294000 -0.818881000 1.281016000

H 3.687363000 -1.515869000 1.788011000

C 5.712062000 -2.457055000 -0.880314000

H 5.592965000 -2.822963000 -1.917064000

H 6.363031000 -1.563696000 -0.900815000

H 6.243408000 -3.241826000 -0.311524000

C -1.474367000 -0.010542000 2.320383000

C -1.022818000 -1.241183000 3.073683000

H -1.365887000 0.942607000 2.864434000

H -2.434534000 -0.092378000 1.789672000

H -0.125757000 -1.749841000 2.520611000

H -1.772155000 -2.052073000 3.015983000

C -0.521900000 -1.012556000 4.511017000

H 0.237848000 -0.211898000 4.535623000

H -1.362470000 -0.706926000

5.159067000

H -0.071886000 -1.926226000 4.938669000

O -1.318872000 -3.826937000 -0.673481000

C -0.009330000 -3.720125000 -1.252852000

H -0.068914000 -3.452089000 -2.326028000

H 0.605530000 -2.978573000 -0.711900000

H 0.437679000 -4.720467000 -1.151589000

O -5.472071000 -2.025608000 0.538874000

C -6.407055000 -0.948943000 0.613029000

H -6.022173000 -0.114859000 1.232596000

H -6.667933000 -0.563397000 -0.392318000

H -7.307426000 -1.369028000 1.086909000

H -3.675700000 -3.832147000 0.298634000

H -4.524533000 0.357319000 -0.484699000

H -0.692466000 -1.306448000 -1.607576000

[3]II-Coor-T

Zero-point correction= 0.656785 (Hartree/Particle)

Thermal correction to Energy= 0.699478

Thermal correction to Enthalpy= 0.700422

Thermal correction to Gibbs Free Energy= 0.581778

Sum of electronic and zero-point Energies= -2094.178415

Sum of electronic and thermal Energies= -2094.135722

Sum of electronic and thermal Enthalpies= -2094.134778

Sum of electronic and thermal Free Energies= -2094.253422

E solvent= -2.094.088200450

C -0.788885000 2.802240000 -0.093761000

C -2.172136000 3.059914000 -0.203813000

H -2.891157000 2.277387000 0.061034000

C -2.627986000 4.315444000 -0.637567000

H -3.705519000 4.501578000 -0.713100000

C -1.712994000 5.329141000 -0.963565000

H -2.071547000 6.309405000 -1.297641000

C -0.334065000 5.081672000 -0.856042000

H 0.388101000 5.868009000 -1.104509000

C 0.126798000 3.826206000 -
0.430926000

C 1.109059000 1.431164000
1.636038000

C 0.993636000 2.603784000
2.421198000

H 0.145880000 3.276586000
2.257583000

C 1.953596000 2.939994000
3.385274000

H 1.832285000 3.851841000
3.980653000

C 3.066492000 2.106789000
3.571229000

H 3.831099000 2.357846000
4.315223000

C 3.188056000 0.936242000
2.813319000

H 4.035962000 0.262534000
2.980186000

C 2.218928000 0.561455000
1.851850000

C 2.416934000 -0.746687000
1.160559000

C 3.667000000 -1.026843000
0.567095000

C 3.893005000 -2.294188000 -
0.008134000

C 2.892765000 -3.281437000
0.030227000

C 1.654418000 -2.999006000
0.631802000

C 1.401106000 -1.725199000
1.187998000

C -1.611355000 0.302602000
1.170638000

C -2.081433000 0.581887000
2.468415000

H -1.515498000 1.252876000
3.126372000

C -3.267777000 -0.014152000
2.901305000

H -3.648713000 0.167184000
3.912132000

C -3.989127000 -0.838034000
2.009821000

H -4.934647000 -1.261293000
2.363564000

C -3.565279000 -1.134840000
0.703397000

C -2.292303000 -0.590240000
0.276875000

Ni 0.007462000 -0.163494000 -
1.455430000

O -1.758553000 -0.882207000 -
0.901176000

P -0.194533000 1.106185000
0.360547000

H 1.204602000 3.648904000 -
0.342277000

C -4.434260000 -1.973269000 -
0.262824000

C -3.682202000 -3.257407000 -
0.703710000

H -4.295329000 -3.828586000 -
1.426898000

H -2.723178000 -3.001613000 -
1.180639000

H -3.484804000 -3.914249000
0.163555000

C -4.764253000 -1.114474000 -
1.515315000

H -5.348702000 -1.708719000 -
2.243401000

H -5.366363000 -0.230209000 -
1.235649000

H -3.838942000 -0.768516000 -
2.003326000

C -5.772778000 -2.409899000
0.377813000

H -5.620138000 -3.063009000
1.257132000

H -6.386650000 -1.545350000
0.690541000

H -6.359786000 -2.983899000 -
0.362355000

C 1.652890000 0.707229000 -
2.085005000

C 1.343423000 1.821247000 -
3.094426000

H 2.235902000 1.102574000 -
1.231877000

H 2.262877000 -0.091972000 -
2.549982000

H 0.681348000 2.581835000 -
2.639482000

H 0.788328000 1.419008000 -
3.963811000

C 2.624769000 2.519240000 -
3.605727000

H 3.188663000 2.976725000 -
2.771079000

H 3.298636000 1.798620000 -
4.105490000

H 2.384788000 3.319163000 -
4.331214000

O 0.754866000 -4.033066000
0.634116000

C -0.466118000 -3.843934000
1.363754000

H -0.264210000 -3.638176000
2.432868000

H -1.074522000 -3.026039000
0.938037000

H -1.012753000 -4.794443000
1.269708000

O 5.062460000 -2.669108000 -
0.624149000

C 6.112476000 -1.703058000 -
0.685523000

H 5.804494000 -0.800267000 -
1.248653000

H 6.451320000 -1.402401000
0.325639000

H 6.941126000 -2.197980000 -
1.214703000

H 3.082707000 -4.271222000 -
0.394549000

H 4.431349000 -0.246440000
0.540100000

H 0.455230000 -1.506233000
1.689139000

C 0.375297000 -2.014072000 -2.394920000

C -0.269539000 -1.181111000 -3.294916000

H 1.458717000 -2.171109000 -2.425398000

H -0.193992000 -2.685382000 -1.744213000

H 0.285275000 -0.661489000 -4.083081000

H -1.362312000 -1.183785000 -3.371248000

^{3}TS-II$_{Ins}$

Zero-point correction= 0.657484 (Hartree/Particle)

Thermal correction to Energy= 0.699293

Thermal correction to Enthalpy= 0.700237

Thermal correction to Gibbs Free Energy= 0.582661

Sum of electronic and zero-point Energies= -2094.161770

Sum of electronic and thermal Energies= -2094.119960

Sum of electronic and thermal Enthalpies= -2094.119016

Sum of electronic and thermal Free Energies= -2094.236592

E solvent= -2.094.071800590

C 0.523197000 2.768641000 0.936799000

C 1.887053000 3.129477000 0.954114000

H 2.598377000 2.581716000 0.326352000

C 2.330847000 4.187731000 1.764170000

H 3.392952000 4.458960000 1.764720000

C 1.421751000 4.898653000 2.563626000

H 1.770236000 5.725176000 3.193348000

C 0.060827000 4.547858000 2.549277000

H -0.657189000 5.102028000 3.165096000

C -0.386374000 3.488756000 1.745182000

C -1.506644000 1.912331000 -0.962073000

C -1.483256000 3.272288000 -1.356263000

H -0.631427000 3.897262000 -1.068100000

C -2.534176000 3.847058000 -2.083195000

H -2.481693000 4.901164000 -2.377807000

C -3.647907000 3.064060000 -2.420647000

H -4.480479000 3.495700000 -2.987834000

C -3.687832000 1.718443000 -

2.037564000

H -4.545635000 1.096723000 -2.317669000

C -2.630585000 1.109843000 -1.318140000

C -2.782912000 -0.336237000 -0.979129000

C -3.978005000 -0.755116000 -0.351233000

C -4.166898000 -2.121549000 -0.056453000

C -3.182538000 -3.063798000 -0.398859000

C -2.002575000 -2.643561000 -1.038411000

C -1.787575000 -1.273865000 -1.321123000

C 1.276184000 0.947988000 -1.237455000

C 1.549046000 1.708315000 -2.391733000

H 0.881939000 2.529159000 -2.681723000

C 2.670763000 1.394969000 -3.160816000

H 2.898596000 1.957355000 -4.072784000

C 3.527236000 0.355378000 -2.737757000

H 4.418654000 0.158221000 -3.341931000

C 3.301355000 -0.424867000 -1.591594000

C 2.095476000 -0.158254000 -0.834552000

Ni 0.096718000 -0.494161000 1.226150000

O 1.739775000 -0.897047000 0.211528000

P -0.035106000 1.282850000 -0.021732000

H -1.452770000 3.234695000 1.731951000

C 4.319753000 -1.493370000 -1.129304000

C 3.669115000 -2.901609000 -1.098274000

H 4.406138000 -3.653494000 -0.756616000

H 2.807399000 -2.919013000 -0.413480000

H 3.327351000 -3.198348000 -2.107416000

C 4.823717000 -1.126946000 0.293513000

H 5.531527000 -1.895085000 0.659360000

H 5.350536000 -0.154821000 0.283759000

H 3.979673000 -1.061185000 0.998210000

C 5.552957000 -1.571486000 -2.059965000

H 5.280426000 -1.868850000 -3.089511000

H 6.099422000 -0.611745000 -

2.109267000

H 6.253771000 -2.332431000 -1.670680000

C 0.668904000 -2.211297000 2.371561000

C 1.815361000 -1.712952000 3.251102000

H 0.143487000 -3.054865000 2.845486000

H 1.011478000 -2.557191000 1.380812000

H 1.414592000 -1.284816000 4.192609000

H 2.357560000 -0.899613000 2.736098000

C 2.804114000 -2.847785000 3.596296000

H 2.295150000 -3.675250000 4.125363000

H 3.263238000 -3.263115000 2.681462000

H 3.618642000 -2.479939000 4.247194000

O -1.126994000 -3.643381000 -1.360604000

C 0.072285000 -3.269386000 -2.054792000

H -0.159623000 -2.829480000 -3.044455000

H 0.673460000 -2.558773000 -1.460611000

H 0.634152000 -4.205275000 -2.192845000

O -5.282273000 -2.634523000 0.560962000

C -6.317220000 -1.718121000 0.918658000

H -5.960921000 -0.961057000 1.644973000

H -6.731497000 -1.201761000 0.030272000

H -7.105659000 -2.327734000 1.385994000

H -3.340979000 -4.124914000 -0.186690000

H -4.728582000 -0.007803000 -0.081668000

H -0.893834000 -0.941174000 -1.854115000

C -1.460890000 0.056809000 2.270222000

C -1.133040000 -1.244533000 2.789493000

H -1.210427000 0.942490000 2.871555000

H -2.347398000 0.154979000 1.632008000

H -0.820508000 -1.302908000 3.839659000

H -1.774775000 -2.074829000 2.469537000

^{3}I-β-C

Zero-point correction= 0.601859 (Hartree/Particle)

Thermal correction to Energy= 0.641173

Thermal correction to Enthalpy= 0.642117

Thermal correction to Gibbs Free Energy= 0.529982

Sum of electronic and zero-point Energies= -2015.625511

Sum of electronic and thermal Energies= -2015.586198

Sum of electronic and thermal Enthalpies= -2015.585253

Sum of electronic and thermal Free Energies= -2015.697388

E solvent= -2.015.512944780

C 0.683535000 2.711576000 0.862372000
C 2.028321000 3.118516000 0.983265000
H 2.811065000 2.559145000 0.459695000
C 2.362736000 4.237036000 1.765677000
H 3.411922000 4.543612000 1.849436000
C 1.362491000 4.960107000 2.433836000
H 1.626234000 5.831547000 3.043965000
C 0.019339000 4.560499000 2.317832000
H -0.768192000 5.120836000 2.835381000
C -0.317807000 3.442068000 1.542381000
C -1.021156000 1.797281000 -1.287228000
C -0.810717000 3.079000000 -1.848891000
H 0.059429000 3.663266000 -1.529837000
C -1.705014000 3.631495000 -2.775083000
H -1.511284000 4.623484000 -3.198439000
C -2.849469000 2.907824000 -3.143989000
H -3.560074000 3.323220000 -3.867731000
C -3.082965000 1.646730000 -2.582701000
H -3.971079000 1.075074000 -2.875220000
C -2.185700000 1.062535000 -1.655823000
C -2.543848000 -0.279114000 -1.106045000
C -3.833160000 -0.453303000 -0.555534000
C -4.222184000 -1.723077000 -0.080086000
C -3.336691000 -2.812052000 -0.156601000
C -2.055373000 -2.636009000 -0.710122000
C -1.643791000 -1.364453000 -1.177088000
C 1.704873000 0.649778000 -0.963524000
C 2.269656000 1.311638000 -2.073450000
H 1.752596000 2.167351000 -2.524134000
C 3.482369000 0.862312000 -2.597524000
H 3.930477000 1.351848000 -3.469089000
C 4.140972000 -0.221492000 -1.977947000
H 5.107523000 -0.530508000 -2.388441000

C 3.624343000 -0.913779000 -
0.871125000
C 2.330210000 -0.499412000 -
0.375380000
Ni 0.049958000 -0.633659000
1.227281000
O 1.741348000 -1.177331000
0.613961000
P 0.214688000 1.171141000 -
0.054824000
H -1.368861000 3.139158000
1.457185000
C 4.416950000 -2.049608000 -
0.182847000
C 3.622812000 -3.382348000 -
0.230516000
H 4.208031000 -4.189713000
0.249896000
H 2.663829000 -3.279946000
0.299855000
H 3.423219000 -3.684616000 -
1.275690000
C 4.678760000 -1.661009000
1.298844000
H 5.218874000 -2.476305000
1.817171000
H 5.299828000 -0.748214000
1.360013000
H 3.729334000 -1.475955000
1.826308000
C 5.787411000 -2.297439000 -
0.855852000
H 5.682501000 -2.621999000 -
1.907800000
H 6.432491000 -1.399928000 -
0.832290000
H 6.317686000 -3.101039000 -
0.313069000
C -0.202233000 -2.080325000
2.478583000
C -1.392141000 -1.236419000
2.719862000
H -0.335426000 -3.026491000
1.936649000
H 0.595254000 -2.085153000
3.235215000
H -2.335366000 -1.720696000
2.407817000
H -1.424166000 -0.310445000
1.911532000
C -1.498627000 -0.526160000
4.078685000
H -1.741757000 -1.263321000
4.864276000
H -0.542290000 -0.043126000
4.343245000
H -2.286935000 0.247413000
4.077763000
O -1.283422000 -3.764055000 -
0.762032000
C 0.018065000 -3.645615000 -
1.359365000
H -0.059242000 -3.365010000 -
2.427951000
H 0.639144000 -2.909230000 -
0.818621000
H 0.469447000 -4.645585000 -
1.276531000
O -5.447812000 -2.000641000
0.477027000
C -6.394768000 -0.934198000
0.549187000
H -6.027567000 -0.102030000
1.182127000
H -6.645221000 -0.541212000 -
0.455787000
H -7.297066000 -1.368074000
1.006598000
H -3.648888000 -3.799441000
0.195380000
H -4.504465000 0.406625000 -
0.492582000

H -0.674267000 -1.228470000 -1.662305000

[3]I-BHE-C

Zero-point correction= 0.599439 (Hartree/Particle)

Thermal correction to Energy= 0.639092

Thermal correction to Enthalpy= 0.640036

Thermal correction to Gibbs Free Energy= 0.526970

Sum of electronic and zero-point Energies= -2015.622698

Sum of electronic and thermal Energies= -2015.583046

Sum of electronic and thermal Enthalpies= -2015.582102

Sum of electronic and thermal Free Energies= -2015.695167

E solvent= -2015.50745138

C 0.603571000 2.729710000 0.801800000

C 1.843785000 3.355393000 0.557593000

H 2.524645000 2.936843000 -0.191659000

C 2.205617000 4.507689000 1.276757000

H 3.172367000 4.985970000 1.080953000

C 1.337528000 5.042690000 2.241093000

H 1.623849000 5.939889000 2.801705000

C 0.102064000 4.419202000 2.491967000

H -0.576698000 4.828089000 3.249490000

C -0.261311000 3.266165000 1.782376000

C -1.268594000 1.783551000 -1.199326000

C -1.145749000 3.075543000 -1.761908000

H -0.266889000 3.683993000 -1.520075000

C -2.138649000 3.606499000 -2.595643000

H -2.015839000 4.607629000 -3.023809000

C -3.291049000 2.851418000 -2.862594000

H -4.079333000 3.252343000 -3.509980000

C -3.432217000 1.576787000 -2.300529000

H -4.324859000 0.979123000 -2.517022000

C -2.433951000 1.010657000 -1.471677000

C -2.680589000 -0.354399000 -0.922851000

C -3.905362000 -0.603893000 -0.268311000

C -4.183745000 -1.899357000

0.214564000

C -3.261990000 -2.942302000 0.023841000

C -2.048322000 -2.692685000 -0.640907000

C -1.736356000 -1.390252000 -1.099992000

C 1.497635000 0.654617000 -1.099952000

C 1.960020000 1.280122000 -2.275741000

H 1.393010000 2.109357000 -2.716819000

C 3.137762000 0.823538000 -2.870159000

H 3.513762000 1.280844000 -3.791764000

C 3.855697000 -0.224712000 -2.255013000

H 4.794602000 -0.537230000 -2.722700000

C 3.433038000 -0.882008000 -1.087759000

C 2.167771000 -0.473939000 -0.513349000

Ni -0.085457000 -0.490819000 1.247327000

O 1.625378000 -1.114452000 0.519370000

P 0.085556000 1.195469000 -0.090519000

H -1.215895000 2.769587000 1.993456000

C 4.300212000 -1.976012000 -0.420994000

C 3.544215000 -3.330317000 -0.380681000

H 4.176167000 -4.105463000 0.093636000

H 2.611184000 -3.235916000 0.195528000

H 3.297664000 -3.673010000 -1.402898000

C 4.641433000 -1.535139000 1.028756000

H 5.232202000 -2.319403000 1.539430000

H 5.237944000 -0.604343000 1.026548000

H 3.718590000 -1.356964000 1.603679000

C 5.633901000 -2.206814000 -1.169938000

H 5.475179000 -2.571135000 -2.201872000

H 6.249737000 -1.289917000 -1.216719000

H 6.221479000 -2.975093000 -0.635066000

C -0.222448000 -2.179102000 2.449436000

C -0.022304000 -1.091899000 3.294029000

H -1.232190000 -2.563040000 2.262318000

H 0.622920000 -2.791229000

2.114923000

H -0.901506000 -0.640253000 3.771890000

H -1.369853000 0.137788000 1.651494000

C 1.315693000 -0.675526000 3.855166000

H 1.403847000 -1.021053000 4.906203000

H 2.149567000 -1.101746000 3.274818000

H 1.422561000 0.424003000 3.867012000

O -1.242485000 -3.784338000 -0.810834000

C 0.001908000 -3.582538000 -1.496891000

H -0.166917000 -3.258727000 -2.542803000

H 0.629925000 -2.843202000 -0.968977000

H 0.499961000 -4.563732000 -1.495106000

O -5.333912000 -2.248057000 0.879890000

C -6.297790000 -1.220547000 1.112220000

H -5.880156000 -0.400239000 1.728683000

H -6.685384000 -0.800183000 0.163170000

H -7.121838000 -1.704179000 1.658853000

H -3.494499000 -3.951286000 0.376161000

H -4.606028000 0.221195000 -0.119475000

H -0.824209000 -1.196469000 -1.670287000

^{3}TS-I$_{Transf-1}$

Zero-point correction= 0.650317 (Hartree/Particle)

Thermal correction to Energy= 0.693774

Thermal correction to Enthalpy= 0.694718

Thermal correction to Gibbs Free Energy= 0.573926

Sum of electronic and zero-point Energies= -2094.142293

Sum of electronic and thermal Energies= -2094.098835

Sum of electronic and thermal Enthalpies= -2094.097891

Sum of electronic and thermal Free Energies= -2094.218684

E solvent= -2094.04975789

C -1.994159000 -2.320298000 -0.153368000

C -3.123954000 -2.138238000 -0.978352000

H -3.140563000 -1.315570000 -1.701317000

C -4.228026000 -3.001806000 -0.869151000

H -5.101798000 -2.846398000 -1.512665000

C -4.213891000 -4.056629000 0.056949000

H -5.076703000 -4.727130000 0.141442000

C -3.089061000 -4.246366000 0.880344000

H -3.072472000 -5.065408000 1.608829000

C -1.990128000 -3.381025000 0.782085000

C 0.729754000 -2.187551000 -1.137369000

C 0.285450000 -3.375423000 -1.766552000

H -0.778125000 -3.628638000 -1.744861000

C 1.172615000 -4.252129000 -2.406095000

H 0.788370000 -5.159204000 -2.886070000

C 2.543075000 -3.962442000 -2.414437000

H 3.255419000 -4.640576000 -2.898111000

C 2.998301000 -2.782757000 -1.812851000

H 4.063799000 -2.529056000 -1.845986000

C 2.119787000 -1.868377000 -1.184232000

C 2.751666000 -0.611468000 -0.671344000

C 3.818982000 -0.713024000 0.248964000

C 4.542550000 0.445012000 0.601772000

C 4.215511000 1.688235000 0.035026000

C 3.152593000 1.783232000 -0.879287000

C 2.407561000 0.635302000 -1.233638000

C -1.048211000 0.229265000 -1.264633000

C -1.079300000 0.225759000 -2.673309000

H -0.743708000 -0.661640000 -3.225174000

C -1.536940000 1.361554000 -3.346076000

H -1.575404000 1.387384000 -4.440546000

C -1.962360000 2.479813000 -2.595206000

H -2.322126000 3.353118000 -3.148621000

C -1.942648000 2.532662000 -1.190824000

C -1.452356000 1.366164000 -0.482902000

Ni -0.648903000 -0.229344000 1.754092000

O -1.366995000 1.336798000 0.840479000

P -0.538653000 -1.170302000 -0.223962000

H -1.124456000 -3.515905000 1.441237000

C -2.420886000 3.786652000 -0.422758000

C -1.260025000 4.355661000 0.438054000

H -1.612385000 5.224473000 1.026303000

H -0.880608000 3.588496000 1.131125000

H -0.424707000 4.696222000 -0.201157000

C -3.609812000 3.407257000 0.500794000

H -3.949583000 4.292486000 1.071554000

H -4.465096000 3.033352000 -0.091817000

H -3.305993000 2.624548000 1.213201000

C -2.900142000 4.910713000 -1.370965000

H -2.092649000 5.262933000 -2.039120000

H -3.755059000 4.593482000 -1.996389000

H -3.232793000 5.775579000 -0.768630000

C -1.395121000 0.383798000 3.614566000

C -2.229364000 -0.631041000 3.151114000

H -0.582571000 0.164660000 4.312515000

H -1.661537000 1.436763000 3.462624000

H -2.036774000 -1.655017000 3.498423000

H 0.001948000 -1.447390000 2.307375000

C -3.576448000 -0.399194000 2.511414000

H -4.367088000 -0.520700000 3.281036000

H -3.655149000 0.614015000 2.087647000

H -3.784663000 -1.132436000 1.714276000

C 1.494518000 1.362157000 2.391724000

C 2.079337000 0.854910000 3.493268000

H 0.889817000 2.275090000 2.424063000

H 1.658982000 0.920613000 1.401901000

H 1.962284000 1.317221000 4.481535000

H 2.692444000 -0.052371000 3.445905000

O 2.928346000 3.034976000 -1.388675000

C 1.886707000 3.173912000 -2.362890000

H 2.092183000 2.561717000 -3.262480000

H 0.897952000 2.898020000 -1.951850000

H 1.885490000 4.239396000 -2.639740000

O 5.595495000 0.462308000 1.484629000

C 5.985326000 -0.780348000 2.068203000

H 5.167831000 -1.226422000 2.668623000

H 6.308773000 -1.510408000 1.299926000

H 6.834403000 -0.545828000 2.728432000

H 4.786587000 2.580621000 0.305568000

H 4.066267000 -1.690482000 0.670296000

H 1.598359000 0.690237000 -1.964143000

^{3}TS-III$_{\mathbf{Decoor}}$

Zero-point correction= 0.650825 (Hartree/Particle)

Thermal correction to Energy= 0.693834

Thermal correction to Enthalpy= 0.694778

Thermal correction to Gibbs Free Energy= 0.575733

Sum of electronic and zero-point Energies= -2094.143399

Sum of electronic and thermal Energies= -2094.100391

Sum of electronic and thermal Enthalpies= -2094.099447

Sum of electronic and thermal Free Energies= -2094.218491

E solvent= -2094.05507474

C	-2.723643	-1.570375	-0.199187
C	-3.729447	-1.019886	-1.021306
H	-3.482929	-0.203209	-1.708588
C	-5.046191	-1.510103	-0.959591
H	-5.817245	-1.069418	-1.602226
C	-5.373558	-2.554913	-0.082162
H	-6.400498	-2.934685	-0.035236
C	-4.377662	-3.108093	0.742889
H	-4.625365	-3.921484	1.434842
C	-3.065874	-2.615061	0.690634
C	-0.083972	-2.291491	-1.137399
C	-0.856850	-3.287946	-1.779748
H	-1.947953	-3.219229	-1.758141
C	-0.265078	-4.378364	-2.433290
H	-0.898687	-5.127323	-2.921818
C	1.129825	-4.503236	-2.446027
H	1.610962	-5.354177	-2.941693
C	1.911664	-3.516366	-1.831623
H	3.004912	-3.586455	-1.863897
C	1.338344	-2.395848	-1.186670
C	2.308388	-1.386736	-0.650880

C	3.248935	-1.790757	0.323051
C	4.255376	-0.889390	0.730387
C	4.343168	0.387791	0.149926
C	3.426671	0.768031	-0.846707
C	2.392921	-0.110382	-1.241115
C	-0.960465	0.581797	-1.209902
C	-1.198529	0.648634	-2.598282
H	-1.361068	-0.273028	-3.171839
C	-1.210332	1.895149	-3.229029
H	-1.390681	1.976319	-4.306473
C	-0.984746	3.058164	-2.457955
H	-1.008409	4.021751	-2.977292
C	-0.720987	3.041187	-1.078449
C	-0.687463	1.749231	-0.418899
Ni	-0.303770	-0.113222	1.678868
O	-0.407301	1.645467	0.869645
P	-0.978838	-0.938862	-0.210765
H	-2.296501	-3.041633	1.345812
C	-0.461949	4.342177	-0.283195
C	0.956499	4.306026	0.349005
H	1.124056	5.217398	0.954473
H	1.068163	3.423544	0.997375
H	1.740008	4.267989	-0.429249
C	-1.518721	4.483209	0.845607
H	-1.328330	5.401852	1.432748
H	-2.539128	4.551802	0.424994
H	-1.471900	3.615740	1.522589
C	-0.553091	5.603619	-1.173097
H	0.202441	5.599410	-1.980448
H	-1.552324	5.719418	-1.632292
H	-0.367598	6.499047	-0.552245
C	-1.822662	0.594333	3.640575
C	-2.871974	0.270184	2.845878
H	-1.352434	-0.146367	4.295490
H	-1.484797	1.633423	3.733933
H	-3.209860	-0.774907	2.814777
H	-0.416781	-1.539422	2.094954
C	-3.673298	1.253107	2.037911
H	-4.710829	1.308350	2.424019
H	-3.233560	2.262875	2.072923
H	-3.747659	0.940376	0.980801
C	1.328457	0.622189	2.822040
C	1.219536	-0.733965	3.093354
H	0.915337	1.378126	3.497137
H	1.988052	0.979390	2.024127
H	0.755006	-1.093974	4.018521
H	1.836267	-1.455687	2.548405
O	3.637835	2.005102	-1.397243
C	2.856696	2.353734	-2.547908
H	2.992535	1.613742	-3.360690
H	1.781933	2.443440	-2.305255
H	3.240525	3.332198	-2.875869
O	5.213757	-1.170638	1.675272
C	5.199218	-2.471825	2.261223
H	4.256521	-2.664439	2.811047
H	5.343439	-3.265112	1.501097

H 6.041397 -2.488531 2.969926

H 5.143472 1.071042 0.447783

H 3.181647 -2.798200 0.742172

H 1.676441 0.170773 -2.015293

Catalyst 4

^{4}I-β-T

Zero-point correction= 0.634514 (Hartree/Particle)

Thermal correction to Energy= 0.676503

Thermal correction to Enthalpy= 0.677447

Thermal correction to Gibbs Free Energy= 0.559425

Sum of electronic and zero-point Energies= -2130.118393

Sum of electronic and thermal Energies= -2130.076404

Sum of electronic and thermal Enthalpies= -2130.075460

Sum of electronic and thermal Free Energies= -2130.193482

E solvent = -2.130.019416

C 1.580552000 2.656588000 0.589741000

C 2.988714000 2.657224000 0.672601000

H 3.553628000 1.810153000 0.268817000

C 3.663178000 3.739102000 1.263343000

H 4.757964000 3.727657000 1.318925000

C 2.943182000 4.829912000 1.775003000

H 3.471994000 5.673111000 2.233962000

C 1.539338000 4.836503000 1.695100000

H 0.969684000 5.685856000 2.090172000

C 0.861145000 3.756973000 1.111233000

C -0.444705000 1.925984000 -1.367832000

C -0.016670000 3.100377000 -2.030861000

H 0.942873000 3.553674000 -1.760365000

C -0.807280000 3.709611000 -3.014434000

H -0.451585000 4.616854000 -3.516009000

C -2.053865000 3.155290000 -3.340598000

H -2.688399000 3.625375000 -4.100863000

C -2.483890000 1.988428000 -2.696100000

H -3.450574000 1.543982000 -2.956899000

C -1.696455000 1.344315000 -1.715698000

C -2.224030000 0.071612000 -

1.130404000

C -3.350110000 0.064373000 -0.276597000

C -3.907602000 -1.135219000 0.216057000

C -3.333771000 -2.357461000 -0.182893000

C -2.233017000 -2.393528000 -1.058028000

C -1.693262000 -1.186160000 -1.530291000

C 1.899951000 0.107196000 -0.865296000

C 2.638253000 0.401521000 -2.026681000

H 2.458505000 1.337686000 -2.569710000

C 3.591642000 -0.515281000 -2.477543000

H 4.181360000 -0.312110000 -3.378249000

C 3.801997000 -1.706064000 -1.746666000

H 4.570289000 -2.395866000 -2.111486000

C 3.082429000 -2.045955000 -0.589300000

C 2.063166000 -1.121779000 -0.141631000

Ni 0.024907000 -0.083126000 1.468657000

O 1.293163000 -1.389801000 0.907130000

P 0.667929000 1.195714000 -0.082842000

H -0.233291000 3.772947000 1.050315000

C -1.221678000 0.982484000 2.426542000

H -0.751220000 1.869218000 2.884814000

C -1.258212000 -0.251049000 3.303038000

H -2.236792000 -0.762592000 3.247469000

H -2.144002000 1.230697000 1.881931000

H -0.557193000 -1.079185000 2.869398000

C -0.784547000 -0.064952000 4.756732000

H -1.497779000 0.573662000 5.307716000

H -0.701143000 -1.029371000 5.289328000

H 0.203242000 0.427579000 4.782622000

C 3.361580000 -3.347738000 0.195619000

C 2.090188000 -4.239760000 0.217769000

H 2.267851000 -5.143692000 0.831675000

H 1.236041000 -3.687022000 0.640597000

H 1.829328000 -4.574517000 -

0.804168000

C 3.766812000 -2.990901000 1.652518000

H 3.942512000 -3.914197000 2.237436000

H 4.699810000 -2.397452000 1.663718000

H 2.971878000 -2.406464000 2.142314000

C 4.511110000 -4.173779000 -0.427332000

H 4.281050000 -4.496614000 -1.459836000

H 5.463595000 -3.612434000 -0.444561000

H 4.673295000 -5.085873000 0.175897000

O -3.846947000 1.308380000 0.052776000

C -5.038789000 1.364745000 0.836530000

H -5.877692000 0.837092000 0.341647000

H -4.885891000 0.939642000 1.848430000

H -5.281781000 2.434491000 0.927968000

O -0.662738000 -1.114930000 -2.423588000

C -0.025261000 -2.336248000 -2.812647000

H 0.361957000 -2.882595000 -1.933885000

H -0.718880000 -2.984029000 -3.384429000

H 0.818918000 -2.033012000 -3.448732000

H -4.767314000 -1.113627000 0.885627000

H -1.832864000 -3.365085000 -1.352562000

O -3.790653000 -3.588152000 0.226046000

C -4.913518000 -3.619918000 1.105073000

H -4.698243000 -3.107450000 2.064033000

H -5.809986000 -3.161857000 0.641739000

H -5.109490000 -4.685468000 1.300110000

^{4}II-Coor-T

Zero-point correction= 0.687773 (Hartree/Particle)

Thermal correction to Energy= 0.733317

Thermal correction to Enthalpy= 0.734261

Thermal correction to Gibbs Free Energy= 0.608104

Sum of electronic and zero-point Energies= -2208.665263

Sum of electronic and thermal Energies= -2208.619719

Sum of electronic and thermal Enthalpies= -2208.618774

Sum of electronic and thermal Free Energies= -2208.744931

E solvent= -2208.58282446

C -0.719731000 2.954787000 -0.038332000

C -2.067844000 3.360875000 -0.136306000

H -2.863341000 2.654950000 0.125461000

C -2.389131000 4.661794000 -0.557748000

H -3.440993000 4.963238000 -0.625074000

C -1.372450000 5.573693000 -0.882674000

H -1.625327000 6.589143000 -1.208512000

C -0.026816000 5.178581000 -0.784117000

H 0.773947000 5.885534000 -1.030824000

C 0.299126000 3.878700000 -0.370151000

C 0.933698000 1.365301000 1.766694000

C 0.883948000 2.518697000 2.584278000

H 0.142304000 3.296080000 2.372928000

C 1.778915000 2.695904000 3.647908000

H 1.717123000 3.597071000 4.268596000

C 2.751946000 1.718021000 3.902474000

H 3.464632000 1.845737000 4.725519000

C 2.805526000 0.568477000 3.104424000

H 3.556771000 -0.202617000 3.306553000

C 1.903013000 0.357310000 2.037826000

C 2.016872000 -0.924687000 1.274261000

C 3.138179000 -1.189836000 0.453512000

C 3.299201000 -2.423052000 -0.213872000

C 2.324252000 -3.421471000 -0.030722000

C 1.217979000 -3.212299000 0.812683000

C 1.079956000 -1.977578000 1.467444000

C -1.813545000 0.442733000 1.024595000

C -2.406066000 0.712590000 2.272480000

H -1.929561000 1.414988000 2.967182000

C -3.598560000 0.068678000 2.612374000

H -4.086314000 0.266322000 3.573325000

C -4.180660000 -0.834640000

1.695498000

H -5.120190000 -1.316960000 1.985028000

C -3.619078000 -1.143616000 0.445520000

C -2.374257000 -0.491795000 0.093855000

Ni -0.090298000 0.173997000 -1.555423000

O -1.753101000 -0.745623000 -1.050983000

P -0.291359000 1.205515000 0.387957000

H 1.351619000 3.583609000 -0.291978000

C 1.714627000 0.864086000 -1.929336000

H 2.176485000 1.258597000 -1.006551000

C 1.927740000 1.855877000 -3.075458000

H 1.597890000 1.423943000 -4.040527000

H 2.201190000 -0.113880000 -2.140560000

H 1.309067000 2.760741000 -2.909956000

C 3.404716000 2.286110000 -3.219024000

H 4.054315000 1.413292000 -3.419598000

H 3.538224000 3.003957000 -4.050299000

H 3.771242000 2.767198000 -2.293131000

C -4.294177000 -2.138213000 -0.526556000

C -3.339313000 -3.329437000 -0.812024000

H -3.785221000 -4.007178000 -1.565207000

H -2.369232000 -2.968238000 -1.188754000

H -3.165447000 -3.919137000 0.107795000

C -4.631634000 -1.414153000 -1.858539000

H -5.096977000 -2.120837000 -2.571950000

H -5.342691000 -0.585405000 -1.684881000

H -3.717523000 -1.003551000 -2.314788000

C -5.611346000 -2.716164000 0.041395000

H -5.447725000 -3.279344000 0.978893000

H -6.362498000 -1.928752000 0.237069000

H -6.048604000 -3.416576000 -0.693541000

O 4.050109000 -0.163867000 0.354892000

C 5.196274000 -0.367264000 -0.469672000

H 5.812244000 -1.216336000 -

0.112212000

H 4.912050000 -0.538600000 -1.526222000

H 5.781096000 0.562414000 -0.398022000

O 0.083662000 -1.703707000 2.361311000

C -0.912117000 -2.706950000 2.583237000

H -1.435499000 -2.966643000 1.645263000

H -0.467923000 -3.617430000 3.031662000

H -1.630884000 -2.252662000 3.280272000

H 4.162227000 -2.595399000 -0.856992000

H 0.501119000 -4.024619000 0.943115000

O 2.372175000 -4.658318000 -0.628431000

C 3.477320000 -4.935998000 -1.486889000

H 3.511899000 -4.238944000 -2.347622000

H 4.441036000 -4.889001000 -0.941731000

H 3.318095000 -5.960893000 -1.855721000

C -0.272246000 -0.716182000 -3.472648000

C -0.965397000 0.486066000 -3.432318000

H 0.718229000 -0.787905000 -3.935953000

H -0.778216000 -1.656667000 -3.228031000

H -0.550979000 1.405611000 -3.857643000

H -2.025220000 0.502960000 -3.158629000

^{4}TS-II$_{Ins}$

Zero-point correction= 0.689012 (Hartree/Particle)

Thermal correction to Energy= 0.733446

Thermal correction to Enthalpy= 0.734390

Thermal correction to Gibbs Free Energy= 0.611309

Sum of electronic and zero-point Energies= -2208.649202

Sum of electronic and thermal Energies= -2208.604768

Sum of electronic and thermal Enthalpies= -2208.603824

Sum of electronic and thermal Free Energies= -2208.726905

E solvent= -2.208.570153
C 1.008825000 2.890700000 0.808510000
C 2.407438000 3.074215000 0.845386000
H 3.055792000 2.383454000 0.295403000
C 2.966445000 4.137984000 1.572680000
H 4.054783000 4.268602000

1.588606000
C 2.138928000 5.032679000
2.269626000
H 2.576809000 5.863464000
2.834735000
C 0.744605000 4.859771000
2.235256000
H 0.089961000 5.557648000
2.770492000
C 0.182299000 3.795760000
1.513987000
C -1.103267000 2.094825000 -
1.037086000
C -0.981754000 3.421820000 -
1.513407000
H -0.092777000 4.006362000 -
1.253735000
C -1.985242000 4.013725000 -
2.291842000
H -1.862915000 5.042434000 -
2.649634000
C -3.142285000 3.282811000 -
2.599127000
H -3.940727000 3.733065000 -
3.200241000
C -3.270425000 1.965667000 -
2.141452000
H -4.165768000 1.385678000 -
2.390399000
C -2.263864000 1.340404000 -
1.370716000
C -2.481647000 -0.090100000 -
0.987720000
C -3.498344000 -0.460831000 -
0.078922000
C -3.778799000 -1.811202000
0.222120000
C -3.034153000 -2.813352000 -
0.429125000
C -2.036714000 -2.486268000 -
1.365830000
C -1.773798000 -1.135060000 -
1.643793000
C 1.574581000 0.785752000 -
1.159831000
C 2.016385000 1.424251000 -
2.333210000
H 1.547029000 2.359924000 -
2.660957000
C 3.046318000 0.842330000 -
3.076602000
H 3.409994000 1.315358000 -
3.995410000
C 3.627639000 -0.361700000 -
2.621337000
H 4.444995000 -0.785939000 -
3.213371000
C 3.215846000 -1.036239000 -
1.460138000
C 2.123581000 -0.457397000 -
0.704952000
Ni 0.145684000 -0.245393000
1.377104000
O 1.630600000 -1.043745000
0.379483000
P 0.290517000 1.401662000 -
0.030240000
H -0.907174000 3.679888000
1.484154000
C 3.904226000 -2.339181000 -
0.991986000
C 2.878713000 -3.504708000 -
0.957648000
H 3.353384000 -4.420999000 -
0.556618000
H 2.015796000 -3.247924000 -
0.323149000
H 2.514480000 -3.735061000 -
1.976748000
C 4.496792000 -2.124921000
0.427292000
H 4.966163000 -3.058839000

0.791839000
H 5.272963000 -1.337421000 0.411248000
H 3.707995000 -1.824353000 1.134420000
C 5.062221000 -2.763813000 -1.925203000
H 4.712902000 -2.972033000 -2.953499000
H 5.857465000 -1.997547000 -1.976859000
H 5.519766000 -3.692008000 -1.536498000
C 0.423165000 -1.924772000 2.683133000
C 1.657817000 -1.551288000 3.502663000
H -0.228127000 -2.616432000 3.240456000
H 0.678511000 -2.414796000 1.727335000
H 1.360238000 -0.957422000 4.391307000
H 2.325732000 -0.909607000 2.899893000
C 2.434851000 -2.800357000 3.972834000
H 1.796354000 -3.458812000 4.591715000
H 2.793973000 -3.390763000 3.111045000
H 3.315089000 -2.516321000 4.578702000
C -1.244898000 0.678033000 2.387935000
C -1.159883000 -0.619266000 3.009454000
H -0.807714000 1.537990000 2.915826000
H -2.114176000 0.911031000 1.761023000
H -0.855170000 -0.650198000 4.063665000
H -1.952057000 -1.336192000 2.756954000
O -4.179387000 0.590530000 0.500044000
C -5.224715000 0.282796000 1.422236000
H -6.026043000 -0.315973000 0.946310000
H -4.840883000 -0.258366000 2.309274000
H -5.634573000 1.254016000 1.738953000
O -0.869029000 -0.714632000 -2.575457000
C -0.072595000 -1.704951000 -3.234725000
H 0.507021000 -2.300201000 -2.506467000
H -0.701171000 -2.373987000 -3.854981000
H 0.622169000 -1.140455000 -3.873258000
O -3.218050000 -4.159955000 -0.223351000
C -4.226400000 -4.555956000 0.704781000
H -4.014746000 -4.177662000 1.724751000
H -5.231091000 -4.210290000 0.389889000
H -4.206998000 -5.656515000 0.714876000
H -1.498096000 -3.297581000 -1.858419000
H -4.560485000 -2.069684000 0.936851000

[4]I-β-C

Zero-point correction= 0.633230 (Hartree/Particle)

Thermal correction to Energy= 0.675338

Thermal correction to Enthalpy= 0.676283

Thermal correction to Gibbs Free Energy= 0.557633

Sum of electronic and zero-point Energies= -2130.112081

Sum of electronic and thermal Energies= -2130.069972

Sum of electronic and thermal Enthalpies= -2130.069028

Sum of electronic and thermal Free Energies= -2130.187678

E solvent = -2130.0099075

C 1.009014000 2.693033000 0.895346000

C 2.371662000 2.955877000 1.147793000

H 3.136555000 2.298883000 0.719760000

C 2.748228000 4.058573000 1.933242000

H 3.811394000 4.252534000 2.117801000

C 1.772865000 4.910916000 2.473942000

H 2.069421000 5.771298000 3.084841000

C 0.412472000 4.656369000 2.225728000

H -0.355993000 5.319541000 2.640358000

C 0.032367000 3.554122000 1.446579000

C -0.654708000 1.883427000 -1.321480000

C -0.311750000 3.119365000 -1.918547000

H 0.593249000 3.641353000 -1.588292000

C -1.119439000 3.701701000 -2.903917000

H -0.829613000 4.657521000 -3.355126000

C -2.300968000 3.054775000 -3.298291000

H -2.946129000 3.498463000 -4.065529000

C -2.657244000 1.836879000 -2.706776000

H -3.579879000 1.330844000 -3.011009000

C -1.851734000 1.225186000 -1.718567000

C -2.317711000 -0.079236000 -1.152609000

C -3.512788000 -0.161457000 -0.400311000

C -4.022254000 -1.395087000 0.063319000

C -3.315718000 -2.573810000 -0.242993000

C -2.121477000 -2.533583000 -

0.986146000

C -1.636755000 -1.294882000 -1.439174000

C 1.966231000 0.524201000 -0.838428000

C 2.649714000 1.116967000 -1.917043000

H 2.256138000 2.030248000 -2.378674000

C 3.817651000 0.524003000 -2.400970000

H 4.360225000 0.963525000 -3.245134000

C 4.302666000 -0.646745000 -1.779728000

H 5.232221000 -1.079465000 -2.163331000

C 3.657612000 -1.277717000 -0.703153000

C 2.421781000 -0.692132000 -0.233964000

Ni 0.127818000 -0.529684000 1.335377000

O 1.727743000 -1.287486000 0.740491000

P 0.471084000 1.181271000 -0.032054000

H -1.031283000 3.368320000 1.252538000

C -0.258899000 -1.839412000 2.697856000

H -0.470225000 -2.814223000 2.238418000

C -1.369135000 -0.873871000 2.844327000

H -1.296429000 -0.000607000 1.979730000

H 0.524202000 -1.850502000 3.469290000

H -2.347984000 -1.286182000 2.538010000

C -1.439253000 -0.060895000 4.147668000

H -0.447814000 0.350007000 4.404238000

H -2.150289000 0.780864000 4.071142000

H -1.765673000 -0.714671000 4.975881000

C 4.244348000 -2.538777000 -0.027619000

C 3.249534000 -3.725679000 -0.144192000

H 3.660518000 -4.619097000 0.364246000

H 2.284738000 -3.467919000 0.320670000

H 3.077825000 -3.989764000 -1.204673000

C 4.515513000 -2.237050000 1.472417000

H 4.900534000 -3.142134000 1.980290000

H 5.272286000 -1.437965000 1.579486000

H 3.590496000 -1.915073000

1.976827000

C 5.580394000 -2.980373000 -0.670344000

H 5.461437000 -3.250984000 -1.736029000

H 6.356486000 -2.196425000 -0.596639000

H 5.959769000 -3.873883000 -0.141616000

O -4.127465000 1.043013000 -0.136384000

C -5.367304000 1.020117000 0.568188000

H -6.137162000 0.436660000 0.025360000

H -5.252531000 0.606893000 1.590293000

H -5.687911000 2.070996000 0.637141000

O -0.516838000 -1.158265000 -2.209272000

C 0.280257000 -2.325442000 -2.445261000

H 0.601262000 -2.781718000 -1.491845000

H -0.269948000 -3.066706000 -3.057511000

H 1.163836000 -1.967971000 -2.993174000

H -4.946995000 -1.432601000 0.639039000

H -1.612800000 -3.473495000 -1.207231000

O -3.718397000 -3.831001000 0.141906000

C -4.939189000 -3.941638000 0.872251000

H -4.888009000 -3.405266000 1.840938000

H -5.800323000 -3.558440000 0.289653000

H -5.077025000 -5.017216000 1.061320000

4I-BHE-C arrivata fin qui da controllare

Zero-point correction= 0.630862 (Hartree/Particle)

Thermal correction to Energy= 0.673397

Thermal correction to Enthalpy= 0.674342

Thermal correction to Gibbs Free Energy= 0.554317

Sum of electronic and zero-point Energies= -2130.110059

Sum of electronic and thermal Energies= -2130.067524

Sum of electronic and thermal Enthalpies= -2130.066579

Sum of electronic and thermal Free Energies= -2130.186604

E solvent= -2.130.006313

C 1.826681000 2.488210000 0.623622000

C 3.210659000 2.470267000 0.353303000

H 3.623348000 1.697867000 -

0.304668000
C 4.054924000 3.436918000
0.926449000
H 5.129391000 3.412803000
0.710414000
C 3.527536000 4.427511000
1.769083000
H 4.188156000 5.179726000
2.215319000
C 2.148184000 4.448745000
2.043859000
H 1.731065000 5.217025000
2.705401000
C 1.302715000 3.482395000
1.481470000
C -0.409658000 2.163431000 -
1.210545000
C 0.074714000 3.350851000 -
1.803478000
H 1.086659000 3.698376000 -
1.568490000
C -0.728829000 4.101961000 -
2.672604000
H -0.335125000 5.019606000 -
3.124450000
C -2.036717000 3.675979000 -
2.948536000
H -2.678520000 4.258458000 -
3.619709000
C -2.523603000 2.498137000 -
2.365059000
H -3.542900000 2.159671000 -
2.581610000
C -1.727978000 1.717105000 -
1.499559000
C -2.312190000 0.455246000 -
0.944834000
C -3.285121000 0.489852000
0.081192000
C -3.919748000 -0.683946000
0.543478000
C -3.572862000 -1.914269000 -
0.046667000
C -2.617608000 -1.986965000 -
1.077304000
C -2.003194000 -0.805700000 -
1.523925000
C 1.688583000 0.028846000 -
1.014830000
C 2.313401000 0.281788000 -
2.250622000
H 2.178813000 1.251332000 -
2.746061000
C 3.091590000 -0.719540000 -
2.837644000
H 3.589838000 -0.550252000 -
3.798574000
C 3.236954000 -1.956099000 -
2.171823000
H 3.861013000 -2.718484000 -
2.649444000
C 2.623099000 -2.256872000 -
0.944238000
C 1.791272000 -1.235218000 -
0.343591000
Ni -0.041709000 -0.081815000
1.496794000
O 1.131887000 -1.452830000
0.790565000
P 0.684012000 1.213415000 -
0.071454000
H 0.230575000 3.491118000
1.710466000
C 2.819698000 -3.624571000 -
0.252084000
C 1.451358000 -4.345422000 -
0.107970000
H 1.574010000 -5.294591000
0.448295000
H 0.731539000 -3.708158000
0.429462000
H 1.032632000 -4.590857000 -

1.102301000
C 3.443110000 -3.405277000
1.153522000
H 3.557450000 -4.373035000
1.678345000
H 4.443546000 -2.942066000
1.069167000
H 2.801706000 -2.745702000
1.759054000
C 3.764189000 -4.558448000 -
1.044598000
H 3.370724000 -4.794194000 -
2.050862000
H 4.775652000 -4.127033000 -
1.160115000
H 3.871168000 -5.514258000 -
0.499627000
O -3.564938000 1.739897000
0.578428000
C -4.493177000 1.834629000
1.656698000
H -5.499666000 1.472579000
1.365923000
H -4.143785000 1.271479000
2.544340000
H -4.551335000 2.905606000
1.904523000
O -1.100632000 -0.759905000 -
2.550089000
C -0.670584000 -2.000600000 -
3.118381000
H -0.213603000 -2.654173000 -
2.352890000
H -1.509760000 -2.528982000 -
3.612465000
H 0.092501000 -1.730733000 -
3.862717000
H -4.666682000 -0.635609000
1.336044000
H -2.388309000 -2.963153000 -
1.507830000
O -4.127564000 -3.119825000
0.314960000
C -5.108973000 -3.112528000
1.350065000
H -4.693835000 -2.732742000
2.304972000
H -5.993428000 -2.505537000
1.071727000
H -5.414179000 -4.162271000
1.479467000
C -0.783667000 -1.440639000
2.883611000
C -0.087902000 -0.471880000
3.597617000
H -1.870033000 -1.369361000
2.751574000
H -0.300500000 -2.378555000
2.588202000
H -0.655631000 0.354656000
4.044682000
C 1.341906000 -0.612747000
4.060980000
H -0.921179000 1.065799000
1.851232000
H 1.364738000 -0.866617000
5.141181000
H 1.872248000 -1.400772000
3.502562000
H 1.896749000 0.335023000
3.940168000

^{4}TS-I$_{Transf-1}$

Zero-point correction= 0.682361 (Hartree/Particle)

Thermal correction to Energy= 0.728269

Thermal correction to Enthalpy= 0.729213

Thermal correction to Gibbs Free Energy=

0.603495

Sum of electronic and zero-point Energies= -2208.627707

Sum of electronic and thermal Energies= -2208.581799

Sum of electronic and thermal Enthalpies= -2208.580854

E solvent= -2208.54452454

C -1.889813000 2.430638000 -0.287412000

C -3.156513000 2.471283000 0.332942000

H -3.410243000 1.724331000 1.093226000

C -4.090181000 3.460847000 -0.022950000

H -5.071461000 3.479417000 0.465755000

C -3.770150000 4.419626000 -0.996969000

H -4.500108000 5.188807000 -1.274133000

C -2.508217000 4.386692000 -1.617593000

H -2.250958000 5.131007000 -2.380397000

C -1.576794000 3.397775000 -1.269791000

C 0.584006000 2.102269000 1.144170000

C 0.085408000 3.256195000 1.797759000

H -0.965407000 3.534298000 1.672482000

C 0.905252000 4.066115000 2.594046000

H 0.483193000 4.948870000 3.087821000

C 2.260690000 3.741722000 2.741671000

H 2.921896000 4.367685000 3.352088000

C 2.765563000 2.601471000 2.107084000

H 3.820978000 2.333593000 2.227204000

C 1.953863000 1.756664000 1.313716000

C 2.633103000 0.547129000 0.750301000

C 3.656358000 0.681359000 -0.218932000

C 4.451687000 -0.412684000 -0.621777000

C 4.220170000 -1.668689000 -0.031203000

C 3.218558000 -1.841484000 0.942160000

C 2.443930000 -0.736742000 1.333210000

C -1.469768000 -0.058698000 1.230096000

C -1.715581000 0.151682000 2.600754000

H -1.370843000 1.074653000 3.082742000

C -2.389996000 -0.828547000 3.332732000

H -2.606686000 -0.682953000 4.396917000

C -2.791407000 -2.016631000 2.683042000

H -3.310945000 -2.771542000 3.282104000

C -2.553767000 -2.279003000 1.324334000

C -1.865946000 -1.262215000 0.552929000

Ni -0.652066000 -0.058550000 -1.731683000

O -1.595442000 -1.432289000 -0.735444000

P -0.635556000 1.123798000 0.126909000

H -0.600566000 3.366009000 -1.768127000

C -2.988931000 -3.611727000 0.672329000

C -1.741802000 -4.350886000 0.114645000

H -2.044135000 -5.288665000 -0.389887000

H -1.210340000 -3.713158000 -0.609601000

H -1.045717000 -4.616424000 0.932608000

C -3.984473000 -3.334314000 -0.485774000

H -4.288943000 -4.283815000 -0.966423000

H -4.895883000 -2.835016000 -0.108088000

H -3.518002000 -2.688565000 -1.245754000

C -3.688671000 -4.558110000 1.675802000

H -3.027846000 -4.833621000 2.518432000

H -4.612802000 -4.114729000 2.090392000

H -3.971997000 -5.492097000 1.157001000

O 3.811274000 1.947388000 -0.734293000

C 4.843162000 2.155986000 -1.697037000

H 5.843926000 1.921057000 -1.283208000

H 4.677281000 1.555038000 -2.612869000

H 4.796559000 3.225695000 -1.952521000

O 1.490452000 -0.791983000 2.310535000

C 1.177873000 -2.070613000 2.871741000

H 0.862090000 -2.785317000 2.089100000

H 2.039952000 -2.485240000 3.430441000

H 0.334652000 -1.888455000 3.553470000

H 5.220537000 -0.288109000 -1.383776000

H 3.082947000 -2.832636000 1.378353000

O 4.928198000 -2.803747000 -0.341003000

C 5.919125000 -2.707610000 -1.363576000

H 5.476518000 -2.392952000 -2.328953000

H 6.728990000 -2.004086000 -1.085153000

H 6.338810000 -3.720002000 -1.467432000

C -1.340825000 -0.718931000 -3.601169000

C -2.123888000 0.375126000 -3.239348000

H -0.478993000 -0.595344000 -4.264146000

H -1.695205000 -1.740467000 -3.422041000

H -1.827300000 1.367563000 -3.604041000

H 0.166058000 1.020913000 -2.352399000

C -3.527629000 0.267075000 -2.697593000

H -4.247402000 0.392974000 -3.533210000

H -3.704586000 -0.712841000 -2.227961000

H -3.745576000 1.057752000 -1.960234000

C 1.199697000 -2.658319000 -2.485663000

C 1.627534000 -1.387037000 -2.364297000

H 1.097654000 -3.148159000 -3.462177000

H 0.914548000 -3.248736000 -1.608452000

H 1.920613000 -0.789835000 -3.235614000

H 1.760298000 -0.913877000 -1.384700000

[4]TS-III$_{Decoor}$

Zero-point correction= 0.682383 (Hartree/Particle)

Thermal correction to Energy= 0.728106

Thermal correction to Enthalpy= 0.729050

Thermal correction to Gibbs Free Energy= 0.604109

Sum of electronic and zero-point Energies= -2208.630082

Sum of electronic and thermal Energies= -2208.584359

Sum of electronic and thermal Enthalpies= -2208.583415

Sum of electronic and thermal Free Energies= -2208.708356

E solvent= -2208.5525096

C 2.876888 -1.508027 -0.090241

C	3.997289	-0.944946	0.555075
H	3.862435	-0.094466	1.232181
C	5.284329	-1.467699	0.333775
H	6.145185	-1.016213	0.840681
C	5.467712	-2.558984	-0.528473
H	6.471366	-2.964554	-0.699957
C	4.355912	-3.126567	-1.177346
H	4.489996	-3.976792	-1.856332
C	3.073285	-2.601400	-0.966001
C	0.353605	-2.172111	1.138770
C	1.196326	-3.094665	1.803894
H	2.281146	-3.010249	1.693144
C	0.678632	-4.126017	2.599382
H	1.362300	-4.820293	3.101398
C	-0.708708	-4.260607	2.740425
H	-1.132827	-5.065369	3.352121
C	-1.554288	-3.346766	2.099596
H	-2.640348	-3.432814	2.216913
C	-1.055592	-2.290286	1.304203
C	-2.082401	-1.358562	0.734255
C	-2.933480	-1.767554	-0.316647
C	-4.024107	-0.982970	-0.748970
C	-4.273690	0.239059	-0.097092
C	-3.465933	0.669756	0.971906
C	-2.396392	-0.137923	1.393757
C	1.275317	0.670801	1.135707
C	1.722132	0.707439	2.472031
H	1.991432	-0.224721	2.984448
C	1.802358	1.934247	3.134240
H	2.158916	1.990575	4.168764
C	1.402908	3.107932	2.456089
H	1.466507	4.055429	3.000824
C	0.917979	3.118988	1.138974
C	0.848658	1.850083	0.437128
Ni	0.367472	0.058832	-1.702190
O	0.401510	1.781349	-0.807429
P	1.159938	-0.831694	0.117136
H	2.212536	-3.039055	-1.486418
C	0.446163	4.426283	0.460411
C	-1.058162	4.307235	0.091696
H	-1.391839	5.210546	-0.454527
H	-1.231800	3.423787	-0.542563
H	-1.679989	4.216513	1.002235
C	1.267547	4.686844	-0.831234
H	0.920802	5.616640	-1.321568
H	2.341618	4.804804	-0.596291
H	1.149277	3.848662	-1.535358
C	0.613169	5.659670	1.378534
H	0.020152	5.571291	2.307687
H	1.669429	5.831157	1.657066
H	0.259702	6.560911	0.844915
O	-2.614477	-2.979755	-0.895621
C	-3.554166	-3.548998	-1.808312
H	-4.557438	-3.647406	-1.349777
H	-3.636359	-2.955110	-2.740576
H	-3.160615	-4.547601	-2.052707

O	-1.618530	0.147085	2.479485
C	-1.875490	1.364877	3.184110
H	-1.738358	2.243647	2.527581
H	-2.895693	1.371183	3.616104
H	-1.124924	1.399686	3.986550
H	-4.649881	-1.313265	-1.578108
H	-3.712336	1.613948	1.460347
O	-5.301761	1.089312	-0.428862
C	-6.154635	0.705636	-1.506745
H	-5.593015	0.606425	-2.456595
H	-6.681012	-0.246003	-1.293359
H	-6.893847	1.515487	-1.604403
C	1.736593	0.867996	-3.665354
C	2.772839	0.610068	-2.826107
H	1.330603	0.094177	-4.324359
H	1.355150	1.888214	-3.794894
H	3.165421	-0.414041	-2.767165
H	0.524611	-1.343851	-2.180899
C	3.501604	1.651895	-2.022206
H	4.565774	1.692108	-2.328139
H	3.060188	2.652626	-2.155137
H	3.489140	1.411278	-0.944126
C	-1.383503	0.740054	-2.713406
C	-1.184984	-0.578194	-3.093238
H	-1.056203	1.574574	-3.341558
H	-2.021470	0.985162	-1.858309
H	-0.729882	-0.827400	-4.058686
H	-1.702342	-1.394965	-2.582955

Catalyst 5

^{5}I-β-T

Zero-point correction= 0.786363 (Hartree/Particle)

Thermal correction to Energy= 0.833924

Thermal correction to Enthalpy= 0.834868

Thermal correction to Gibbs Free Energy= 0.704528

Sum of electronic and zero-point Energies= -2140.197069

Sum of electronic and thermal Energies= -2140.149508

Sum of electronic and thermal Enthalpies= -2140.148564

Sum of electronic and thermal Free Energies= -2140.278904

E solvent= -2140.16574911

C	1.548678000	2.632837000	0.734809000
C	2.945588000	2.767121000	0.877491000
H	3.607705000	2.004383000	0.452973000
C	3.483644000	3.876031000	1.551415000
H	4.570921000	3.971371000	1.653239000
C	2.636889000	4.860158000	2.086176000
H	3.059401000	5.725328000	2.609740000

C 1.243893000 4.733786000 1.943521000

H 0.576817000 5.501312000 2.353146000

C 0.701461000 3.626363000 1.275288000

C -0.331184000 1.832389000 -1.324110000

C 0.159773000 2.957268000 -2.033245000

H 1.098385000 3.423394000 -1.714285000

C -0.531817000 3.498083000 -3.125084000

H -0.123745000 4.366363000 -3.654468000

C -1.742350000 2.916933000 -3.528697000

H -2.294046000 3.317716000 -4.386649000

C -2.255141000 1.825701000 -2.815230000

H -3.214802000 1.386061000 -3.109951000

C -1.584279000 1.268657000 -1.699556000

C -2.292683000 0.165154000 -0.957707000

C -3.386159000 0.497050000 -0.106743000

C -4.081276000 -0.544806000 0.540471000

C -3.742103000 -1.896177000 0.366888000

C -2.676257000 -2.199653000 -0.500535000

C -1.952900000 -1.201192000 -1.179246000

C 2.170629000 0.298347000 -0.968872000

C 2.757259000 0.751757000 -2.167663000

H 2.358322000 1.635811000 -2.678232000

C 3.841480000 0.054604000 -2.702629000

H 4.308836000 0.382508000 -3.637540000

C 4.341876000 -1.075525000 -2.019452000

H 5.205774000 -1.589730000 -2.452473000

C 3.789836000 -1.568893000 -0.827074000

C 2.633430000 -0.882828000 -0.295669000

Ni 0.523917000 -0.335842000 1.463564000

O 2.003577000 -1.322305000 0.788774000

P 0.821151000 1.121879000 -0.049945000

H -0.385881000 3.538734000 1.163764000

C -0.877216000 0.316235000 2.563603000

C -0.400654000 -0.818990000 3.441818000

H -0.710336000 1.328196000 2.968710000

H -1.872449000 0.189570000 2.114094000

H -1.182285000 -1.591480000 3.571471000

C -0.899153000 -1.601539000 -2.219268000

C -3.869352000 1.941315000 0.095874000

C 4.390265000 -2.787677000 -0.089402000

C 3.341671000 -3.928520000 0.010395000

H 3.771692000 -4.792076000 0.553349000

H 2.443744000 -3.585023000 0.547461000

H 3.039626000 -4.274240000 -0.995217000

C 4.823784000 -2.357276000 1.339425000

H 5.223965000 -3.227095000 1.894780000

H 5.617324000 -1.588456000 1.292653000

H 3.967470000 -1.945644000 1.897311000

C 5.636336000 -3.352297000 -0.812098000

H 5.397922000 -3.718915000 -1.827775000

H 6.445305000 -2.603007000 -0.894971000

H 6.033937000 -4.207652000 -0.235890000

H 0.415112000 -1.445189000 2.887043000

C 0.242266000 -0.419584000 4.781866000

H 0.694191000 -1.287056000 5.295087000

H 1.031763000 0.336050000 4.625569000

H -0.521452000 0.016860000 5.449861000

H -4.923923000 -0.294778000 1.198463000

H -2.409472000 -3.249375000 -0.669362000

C -4.533373000 -2.997099000 1.073647000

C -5.231664000 2.163691000 -0.605267000

C -3.964290000 2.351254000 1.583602000

H -3.138515000 2.617409000 -0.382121000

H -4.283935000 3.406640000 1.666142000

H -4.703401000 1.740468000 2.133912000

H -2.993227000 2.246631000 2.097278000

H -5.560318000 3.213510000 -0.493154000

H -5.173811000 1.936771000 -1.684414000

H -6.013405000 1.515066000 -0.168618000

C -0.049283000 -2.821048000 -1.807990000

C -1.580298000 -1.850034000 -3.588377000

H -0.204081000 -0.752192000 -2.349144000

H -0.826024000 -2.115102000 -4.352015000

H -2.306166000 -2.681688000 -3.519493000

H -2.122730000 -0.954610000 -3.940472000

H 0.764386000 -2.970141000 -2.539852000

H 0.409846000 -2.673768000 -0.815856000

H -0.643599000 -3.753843000 -1.787893000

C -3.632810000 -3.872577000 1.974304000

H -5.276897000 -2.497488000 1.726734000

C -5.313030000 -3.869526000 0.061686000

H -5.925156000 -4.625426000 0.587644000

H -5.985443000 -3.254367000 -0.562045000

H -4.623555000 -4.406596000 -0.615205000

H -4.232314000 -4.638355000 2.500076000

H -2.862450000 -4.398188000 1.380929000

H -3.113486000 -3.261805000 2.734682000

[5]II-Coor-T

Zero-point correction= 0.840066 (Hartree/Particle)

Thermal correction to Energy= 0.890934

Thermal correction to Enthalpy= 0.891878

Thermal correction to Gibbs Free Energy= 0.755874

Sum of electronic and zero-point Energies= -2218.743373

Sum of electronic and thermal Energies= -2218.692506

Sum of electronic and thermal Enthalpies= -2218.691562

Sum of electronic and thermal Free Energies= -2218.827565

E solvent= -2218.73289256

C 2.365334000 -2.220964000 0.203479000

C 3.729661000 -1.872561000 0.102621000

H 4.032462000 -0.829526000 0.246099000

C 4.695651000 -2.854969000 -0.169538000

H 5.751630000 -2.569238000 -0.238763000

C 4.314989000 -4.195211000 -0.345193000

H 5.070826000 -4.960285000 -0.555723000

C 2.959470000 -4.551131000 -0.245421000

H 2.653272000 -5.595744000 -0.374683000

C 1.989962000 -3.572472000 0.023134000

C -0.035025000 -1.573292000 1.711885000

C 0.585612000 -2.378140000 2.700946000

H 1.636555000 -2.658869000 2.578868000

C -0.109038000 -2.831038000 3.829788000

H 0.404691000 -3.449520000 4.574307000

C -1.454761000 -2.478092000 3.995568000

H -2.016311000 -2.812389000 4.875314000

C -2.082933000 -1.691158000 3.021684000

H -3.134940000 -1.412238000 3.147460000

C -1.409773000 -1.228292000 1.864093000

C -2.249047000 -0.411687000 0.909907000

C -3.112484000 -1.074389000 -0.011758000

C -4.015773000 -0.302473000 -0.769086000

C -4.114540000 1.092572000 -0.631873000

C -3.265819000 1.721428000 0.296254000

C -2.340828000 1.001182000 1.076483000

C 1.949700000 0.529857000 1.132080000

C 2.481619000 0.569358000 2.436870000

H 2.314854000 -0.267986000 3.125190000

C 3.211335000 1.687961000 2.841396000

H 3.627852000 1.747668000 3.852776000

C 3.420786000 2.742284000 1.925610000

H 4.016391000 3.596416000 2.263515000

C 2.907487000 2.750845000 0.618915000

C 2.103735000 1.618134000 0.210911000

Ni 0.608233000 -0.036287000 -1.614594000

O 1.520555000 1.573634000 -0.981404000

P 1.085891000 -0.894641000 0.388573000

H 0.937728000 -3.866074000 0.111582000

C 0.147553000 -1.739913000 -2.445819000

C 1.406639000 -2.134167000 -3.230862000

H -0.123262000 -2.522688000 -1.713853000

H -0.718088000 -1.587112000 -3.118262000

H 1.676995000 -1.339644000 -3.954241000

C -1.524536000 1.742337000 2.142145000

C -3.145943000 -2.603926000 -0.143951000

C 3.195879000 3.912420000 -0.359675000

C 1.870601000 4.596414000 -0.791726000

H 2.080174000 5.410695000 -1.511758000

H 1.194418000 3.868833000 -1.266660000

H 1.354960000 5.038579000 0.080635000

C 3.927482000 3.355918000 -1.612255000

H 4.106198000 4.167283000 -2.343587000

H 4.908237000 2.927546000 -1.333566000

H 3.326573000 2.569537000 -2.096423000

C 4.099951000 4.998541000 0.269436000

H 3.627802000 5.476283000 1.147877000

H 5.081543000 4.594798000 0.579132000

H 4.287698000 5.791596000 -0.477275000

H 2.269506000 -2.239404000 -2.545588000

C 1.226256000 -3.465882000 -3.998586000

H 2.138910000 -3.723500000 -4.567689000

H 1.015906000 -4.296305000 -3.300394000

H 0.384659000 -3.403840000 -4.713059000

H -4.684681000 -0.805882000 -1.478623000

H -3.336814000 2.807045000 0.434319000

C -5.139875000 1.883998000 -1.444694000

C -4.303012000 -3.201333000 0.695766000

C -3.266516000 -3.096721000 -1.603604000

H -2.197415000 -3.000047000 0.264376000

H -3.167825000 -4.197306000 -1.637295000

H -4.251392000 -2.848123000 -2.040007000

H -2.485508000 -2.662629000 -2.249149000

H -4.319708000 -4.303368000 0.603812000

H -4.205300000 -2.950745000 1.765551000

H -5.277724000 -2.815087000 0.344231000

C -0.913438000 3.062862000 1.627622000

C -2.385486000 1.999900000 3.402969000

H -0.682626000 1.093021000 2.442125000

H -1.788909000 2.515926000 4.177848000

H -3.256543000 2.637404000 3.161829000

H -2.765399000 1.057741000 3.836172000

H -0.230811000 3.481226000 2.388541000

H -0.332574000 2.901838000 0.704514000

H -1.687496000 3.826450000 1.424091000

C -4.483733000 2.993055000 -2.297397000

H -5.624980000 1.169333000 -2.139561000

C -6.244345000 2.470976000 -0.534054000

H -7.006887000 2.999330000 -1.135610000

H -6.750232000 1.677679000 0.044364000

H -5.822081000 3.195373000 0.186542000

H -5.243025000 3.510062000 -2.912662000

H -3.996864000 3.754208000 -1.660539000

H -3.715624000 2.579239000 -2.974485000

C -0.922861000 1.061188000 -2.486470000

C 0.135683000 1.037625000 -3.386356000

H -1.786801000 0.395362000 -2.586019000

H -1.025778000 1.876910000 -1.762619000

H 0.135787000 0.352472000 -4.241463000

H 0.882903000 1.838022000 -3.387700000

[5]TS-II$_{Ins}$

Zero-point correction= 0.841488 (Hartree/Particle)

Thermal correction to Energy=

0.891313

Thermal correction to Enthalpy= 0.892257

Thermal correction to Gibbs Free Energy= 0.758030

Sum of electronic and zero-point Energies= -2218.725215

Sum of electronic and thermal Energies= -2218.675390

Sum of electronic and thermal Enthalpies= -2218.674446

Sum of electronic and thermal Free Energies= -2218.808673

E solvent= -2218.71422260

C 1.336399000 2.731936000 0.805976000

C 2.739822000 2.870552000 0.851862000

H 3.370356000 2.164932000 0.299469000

C 3.326320000 3.911269000 1.590447000

H 4.417889000 4.009309000 1.613330000

C 2.521848000 4.825709000 2.289669000

H 2.981691000 5.638309000 2.863551000

C 1.123168000 4.698210000 2.243742000

H 0.487041000 5.413654000 2.777978000

C 0.533143000 3.658095000 1.509313000

C -0.789678000 2.055769000 -1.055370000

C -0.473498000 3.311201000 -1.634361000

H 0.460197000 3.808930000 -1.351973000

C -1.324104000 3.937323000 -2.554890000

H -1.045507000 4.906282000 -2.984106000

C -2.521161000 3.307739000 -2.922562000

H -3.194228000 3.770824000 -3.653054000

C -2.860673000 2.081705000 -2.337007000

H -3.804397000 1.595022000 -2.607405000

C -2.031104000 1.437065000 -1.387073000

C -2.572938000 0.172221000 -0.770727000

C -3.612825000 0.264568000 0.202689000

C -4.149127000 -0.925343000 0.734968000

C -3.709816000 -2.195967000 0.331140000

C -2.709969000 -2.261194000 -0.656823000

C -2.141790000 -1.107052000 -1.227669000

C 1.800913000 0.701432000 -
1.277042000

C 2.113779000 1.334304000 -
2.496725000

H 1.561078000 2.225977000 -
2.813763000

C 3.120214000 0.801653000 -
3.302285000

H 3.376429000 1.269838000 -
4.258877000

C 3.812331000 -0.349849000 -
2.869301000

H 4.604159000 -0.738479000 -
3.517494000

C 3.537267000 -1.017341000 -
1.665439000

C 2.470243000 -0.489355000 -
0.840169000

Ni 0.660309000 -0.360779000
1.397337000

O 2.106132000 -1.076966000
0.294007000

P 0.576239000 1.273273000 -
0.054059000

H -0.559681000 3.579533000
1.466648000

C 1.331745000 -1.840365000
2.800911000

C 2.569079000 -1.192815000
3.422105000

H 0.859510000 -2.543435000
3.504692000

H 1.557378000 -2.399961000
1.876970000

H 2.271174000 -0.515545000
4.248126000

C -1.164889000 -1.246148000 -
2.401010000

C -4.211879000 1.606839000
0.654915000

C 4.345968000 -2.261158000 -
1.230309000

C 3.406068000 -3.487244000 -
1.078403000

H 3.979266000 -4.364843000 -
0.722705000

H 2.600360000 -3.276592000 -
0.358249000

H 2.947147000 -3.751627000 -
2.048748000

C 5.046466000 -1.968738000
0.124275000

H 5.606679000 -2.859931000
0.466599000

H 5.764684000 -1.134003000
0.021806000

H 4.304930000 -1.700424000
0.892985000

C 5.442451000 -2.638320000 -
2.254889000

H 5.017941000 -2.893939000 -
3.243331000

H 6.181333000 -1.827557000 -
2.393659000

H 5.989146000 -3.526879000 -
1.889699000

H 3.082461000 -0.568352000 2.669032000

C 3.551967000 -2.250482000 3.972024000

H 4.434402000 -1.769896000 4.433202000

H 3.068865000 -2.881503000 4.741600000

H 3.911551000 -2.915062000 3.166352000

H -4.944397000 -0.860162000 1.488949000

H -2.370373000 -3.242898000 -1.006841000

C -4.322794000 -3.459986000 0.933113000

C -5.608956000 1.817112000 0.019941000

C -4.316249000 1.756705000 2.190725000

H -3.554269000 2.415973000 0.289566000

H -4.715058000 2.756627000 2.442823000

H -5.002736000 1.011300000 2.632112000

H -3.336197000 1.644974000 2.684817000

H -6.026994000 2.796769000 0.317223000

H -5.566802000 1.783662000 -1.082450000

H -6.313229000 1.032002000 0.352003000

C -0.188992000 -2.431709000 -2.258311000

C -1.950765000 -1.349858000 -3.732266000

H -0.552920000 -0.327517000 -2.451167000

H -1.252729000 -1.422001000 -4.586612000

H -2.594101000 -2.249548000 -3.737752000

H -2.597458000 -0.469093000 -3.893601000

H 0.572626000 -2.383647000 -3.056300000

H 0.334395000 -2.405603000 -1.287859000

H -0.703900000 -3.406218000 -2.354827000

C -3.268900000 -4.315759000 1.672451000

H -5.073542000 -3.134348000 1.681100000

C -5.063079000 -4.299311000 -0.134166000

H -5.551701000 -5.176173000 0.329431000

H -5.839108000 -3.702811000 -0.645620000

H -4.363744000 -4.673773000 -0.904196000

H -3.741721000 -5.197288000 2.143592000

H -2.491277000 -4.682696000 0.977760000

H -2.764758000 -3.732851000 2.464143000

C -0.794676000 0.404813000 2.441889000

C -0.416125000 -0.763258000 3.187891000

H -0.518361000 1.394896000 2.830989000

H -1.711095000 0.365110000 1.843463000

H -0.002833000 -0.608696000 4.192521000

H -1.082095000 -1.631957000 3.111337000

^{5}I-β-C

Zero-point correction= 0.785407 (Hartree/Particle)

Thermal correction to Energy= 0.833025

Thermal correction to Enthalpy= 0.833969

Thermal correction to Gibbs Free Energy= 0.703237

Sum of electronic and zero-point Energies= -2140.193251

Sum of electronic and thermal Energies= -2140.145633

Sum of electronic and thermal Enthalpies= -2140.144689

Sum of electronic and thermal Free Energies= -2140.275421

E solvent = -2140.16268212

C -1.644323000 2.667051000 -0.703344000

C -3.046438000 2.781599000 -0.803320000

H -3.683648000 2.008886000 -0.359551000

C -3.623016000 3.882417000 -1.459582000

H -4.714393000 3.960037000 -1.527702000

C -2.809722000 4.879420000 -2.020473000

H -3.261738000 5.737620000 -2.530773000

C -1.411028000 4.772796000 -1.923902000

H -0.768784000 5.548764000 -2.356849000

C -0.831941000 3.672820000 -1.275024000

C 0.219237000 1.913127000 1.377980000

C -0.284826000 3.025834000 2.093687000

H -1.239685000 3.468823000 1.789495000

C 0.419029000 3.586135000 3.168056000

H 0.003079000 4.445580000 3.705885000

C 1.655435000 3.039584000 3.542525000

H 2.218919000 3.462283000
4.382181000

C 2.177802000 1.954233000
2.825957000

H 3.154645000 1.539151000
3.100150000

C 1.488495000 1.374805000
1.734022000

C 2.184579000 0.259953000
0.994875000

C 3.207204000 0.579964000
0.057470000

C 3.916148000 -0.469437000 -
0.562174000

C 3.657313000 -1.820334000 -
0.277541000

C 2.648020000 -2.111665000
0.658889000

C 1.911563000 -1.103731000
1.308847000

C -2.179558000 0.254940000
0.891099000

C -2.853218000 0.657860000
2.063045000

H -2.538351000 1.564963000
2.592360000

C -3.909954000 -0.113254000
2.548758000

H -4.438335000 0.176177000
3.463710000

C -4.300997000 -1.270249000
1.840891000

H -5.145851000 -1.845490000
2.232594000

C -3.664699000 -1.716224000
0.672033000

C -2.537178000 -0.946592000
0.195859000

Ni -0.389825000 -0.372781000 -
1.477759000

O -1.851903000 -1.361686000 -
0.871486000

P -0.847798000 1.173512000
0.051799000

H 0.260496000 3.600233000 -
1.204287000

C 0.031465000 -1.543881000 -
2.948269000

C 1.002764000 -0.444647000 -
3.123586000

H 0.389455000 -2.506978000 -
2.560245000

H -0.811047000 -1.613422000 -
3.650786000

H 0.914384000 0.351411000 -
2.187597000

C 0.896023000 -1.484056000
2.392795000

C 3.592933000 2.028252000 -
0.271792000

C -4.150151000 -2.969727000 -
0.092085000

C -3.024301000 -4.037643000 -
0.153960000

H -3.379067000 -4.927952000 -
0.708190000

H -2.134380000 -3.635905000 -0.662900000

H -2.733894000 -4.362488000 0.862093000

C -4.558512000 -2.564202000 -1.535586000

H -4.875734000 -3.458103000 -2.106070000

H -5.405215000 -1.853136000 -1.517316000

H -3.713063000 -2.091517000 -2.060338000

C -5.380567000 -3.623617000 0.579501000

H -5.157500000 -3.977669000 1.603073000

H -6.243537000 -2.934227000 0.630731000

H -5.692983000 -4.502204000 -0.013889000

H 2.049326000 -0.747607000 -2.936849000

C 0.847484000 0.444855000 -4.367580000

H 1.458070000 1.362291000 -4.298290000

H 1.169857000 -0.114603000 -5.263545000

H -0.206139000 0.741560000 -4.507453000

H 4.713172000 -0.225595000 -1.277600000

H 2.434972000 -3.158700000 0.903866000

C 4.472232000 -2.930238000 -0.943300000

C 4.997440000 2.368052000 0.282273000

C 3.521877000 2.335680000 -1.784919000

H 2.871215000 2.696364000 0.230988000

H 3.779025000 3.394330000 -1.973950000

H 4.228642000 1.715199000 -2.365938000

H 2.507039000 2.155527000 -2.182707000

H 5.253820000 3.423424000 0.073647000

H 5.046153000 2.213270000 1.374561000

H 5.774828000 1.732884000 -0.181221000

C 0.069899000 -2.739812000 2.044096000

C 1.607371000 -1.662627000 3.756800000

H 0.183025000 -0.646240000 2.498441000

H 0.874146000 -1.921912000 4.542638000

H 2.355311000 -2.475761000 3.704647000

H 2.129052000 -0.740280000 4.067918000

H -0.747934000 -2.862668000 2.776178000

H -0.379466000 -2.657809000 1.040192000

H 0.681335000 -3.661118000 2.080453000

C 3.581709000 -3.909284000 -1.742082000

H 5.162793000 -2.442676000 -1.660635000

C 5.334545000 -3.692225000 0.090412000

H 5.957934000 -4.456582000 -0.409310000

H 6.003091000 -3.005180000 0.638564000

H 4.699592000 -4.208908000 0.833352000

H 4.198585000 -4.682684000 -2.235633000

H 2.861595000 -4.425966000 -1.081806000

H 3.003636000 -3.382319000 -2.522222000

[5]I-BHE-C

Zero-point correction= 0.782831 (Hartree/Particle)

Thermal correction to Energy= 0.830919

Thermal correction to Enthalpy= 0.831863

Thermal correction to Gibbs Free Energy= 0.699794

Sum of electronic and zero-point Energies= -2140.190858

Sum of electronic and thermal Energies= -2140.142770

Sum of electronic and thermal Enthalpies= -2140.141826

Sum of electronic and thermal Free Energies= -2140.273895

E solvent= -2140.15601179

C -2.140866000 2.457124000 -0.512998000

C -3.509138000 2.453495000 -0.171211000

H -3.899501000 1.673865000 0.491962000

C -4.366660000 3.443629000 -0.681328000

H -5.428764000 3.430664000 -0.410513000

C -3.868011000 4.443119000 -1.530909000

H -4.538949000 5.213652000 -1.927561000

C -2.504630000 4.450052000 -1.876398000

H -2.110226000 5.225120000 -2.543801000

C -1.646529000 3.460522000 -1.377043000

C 0.140146000 2.074540000 1.251402000

C -0.412197000 3.153793000 1.981113000

H -1.449050000 3.454278000 1.792950000

C 0.344058000 3.856059000 2.929299000

H -0.108052000 4.686474000 3.483161000

C 1.678119000 3.488485000 3.155115000

H 2.285825000 4.026193000 3.891823000

C 2.238229000 2.428713000 2.427835000

H 3.282964000 2.144975000 2.598009000

C 1.497710000 1.700472000 1.466971000

C 2.218154000 0.594027000 0.736394000

C 3.028844000 0.904973000 -0.393863000

C 3.781919000 -0.123478000 -0.992676000

C 3.770610000 -1.442847000 -0.509214000

C 2.971550000 -1.723531000 0.613591000

C 2.202348000 -0.733890000 1.254417000

C -1.954718000 -0.032400000 1.074753000

C -2.510513000 0.195779000 2.349864000

H -2.313342000 1.138174000 2.876120000

C -3.303304000 -0.795051000 2.932385000

H -3.742421000 -0.648246000 3.925126000

C -3.539168000 -1.993233000 2.223253000

H -4.173567000 -2.747204000 2.700226000

C -3.000453000 -2.268570000 0.956092000

C -2.145275000 -1.263857000 0.360762000

Ni -0.376749000 -0.104220000 -1.533052000

O -1.540761000 -1.467335000 -0.806244000

P -0.982356000 1.156792000 0.104516000

H -0.588539000 3.457930000 -1.664112000

C 0.324821000 -1.454220000 -2.949898000

C -0.376357000 -0.479286000 -3.647709000

H 1.412929000 -1.384088000 -2.831231000

H -0.157750000 -2.392439000 -2.654063000

H 0.489599000 1.041972000 -1.905220000

C 1.441267000 -1.084940000 2.539294000

C 3.148688000 2.335433000 -0.935664000

C -3.297219000 -3.593663000 0.218499000

C -1.975656000 -4.372437000 -0.024668000

H -2.178279000 -5.297920000 -0.597342000

H -1.260311000 -3.754210000 -0.589546000

H -1.509363000 -4.661193000 0.935191000

C -3.977023000 -3.282378000 -1.142907000

H -4.154168000 -4.219234000 -1.705106000

H -4.952929000 -2.786026000 -0.988741000

H -3.339246000 -2.619848000 -1.749282000

C -4.247288000 -4.515063000 1.018874000

H -3.815814000 -4.814785000 1.991837000

H -5.228657000 -4.040878000 1.205361000

H -4.429971000 -5.438502000 0.439500000

H 0.189209000 0.348309000 -4.095835000

C -1.813235000 -0.609470000 -4.090746000

H -2.364780000 0.337371000 -3.948407000

H -1.852129000 -0.846118000 -5.174122000

H -2.337666000 -1.405327000 -3.537720000

H 4.410262000 0.111595000 -1.861381000

H 2.956441000 -2.742729000 1.017174000

C 4.633521000 -2.520753000 -1.166755000

C 4.450745000 3.006537000 -0.433033000

C 3.076782000 2.416257000 -2.477044000

H 2.298115000 2.917832000 -0.535969000

H 3.055928000 3.473859000 -2.799060000

H 3.954723000 1.947430000 -2.958303000

H 2.167762000 1.917294000 -2.857016000

H 4.524721000 4.043796000 -0.809885000

H 4.492120000 3.040840000 0.669514000

H 5.339281000 2.451521000 -0.787212000

C 0.729860000 -2.453669000 2.479196000

C 2.393804000 -1.032981000 3.759794000

H 0.659969000 -0.318312000 2.691814000

H 1.845121000 -1.273602000 4.689243000

H 3.212483000 -1.767835000 3.646029000

H 2.849400000 -0.034606000 3.881690000

H 0.081109000 -2.576640000 3.364648000

H 0.098537000 -2.542650000 1.580058000

H 1.450601000 -3.292958000 2.481530000

C 3.802505000 -3.735621000 -1.636288000

H 5.096498000 -2.064506000 -2.065034000

C 5.780653000 -2.968236000 -0.229882000

H 6.430926000 -3.706725000 -0.734324000

H 6.405256000 -2.110372000 0.076215000

H 5.383274000 -3.439891000 0.687637000

H 4.447834000 -4.474089000 -2.146941000

H 3.319146000 -4.247293000 -0.784140000

H 3.007267000 -3.430558000 -2.339311000

^{5}TS-I$_{Transf-1}$

Zero-point correction= 0.834412 (Hartree/Particle)

Thermal correction to Energy= 0.885803

Thermal correction to Enthalpy= 0.886747

Thermal correction to Gibbs Free Energy= 0.750322

Sum of electronic and zero-point Energies= -2218.712046

Sum of electronic and thermal Energies= -2218.660655

Sum of electronic and thermal Enthalpies= -2218.659711

Sum of electronic and thermal Free Energies= -2218.796135

E solvent = -2218.70125490

C 2.403124000 -2.217457000 -0.008116000

C 3.619782000 -2.069611000 0.690687000

H 3.739326000 -1.241753000 1.398610000

C 4.673659000 -2.976022000 0.478098000

H 5.615159000 -2.849226000 1.025199000

C 4.522935000 -4.037270000 -0.428580000

H 5.346381000 -4.741249000 -0.594408000

C 3.311391000 -4.191352000 -

1.126550000

H 3.187868000 -5.016288000 -1.837966000

C 2.260474000 -3.285474000 -0.922730000

C -0.173655000 -2.022827000 1.266956000

C 0.414002000 -3.032862000 2.069258000

H 1.488989000 -3.222123000 1.991749000

C -0.341365000 -3.805010000 2.961728000

H 0.151463000 -4.573668000 3.567644000

C -1.720934000 -3.585633000 3.064973000

H -2.332961000 -4.181079000 3.752029000

C -2.316944000 -2.594406000 2.275116000

H -3.396401000 -2.418899000 2.347177000

C -1.575833000 -1.793656000 1.371282000

C -2.392748000 -0.783036000 0.604351000

C -3.035901000 -1.171864000 -0.605654000

C -3.969800000 -0.293987000 -1.189546000

C -4.299106000 0.945914000 -0.615753000

C -3.646413000 1.310717000 0.574948000

C -2.702714000 0.474038000 1.201047000

C 1.614921000 0.327522000 1.260463000

C 1.722261000 0.274603000 2.665024000

H 1.387235000 -0.619089000 3.205747000

C 2.256158000 1.367573000 3.349294000

H 2.350465000 1.353460000 4.440581000

C 2.678926000 2.497020000 2.615831000

H 3.093740000 3.338705000 3.179403000

C 2.591690000 2.598939000 1.217896000

C 2.024246000 1.476029000 0.497552000

Ni 1.025257000 -0.005341000 -1.737884000

O 1.875589000 1.494226000 -0.821360000

P 0.998287000 -1.022457000 0.210026000

H 1.324973000 -3.396661000 -1.483648000

C 1.712054000 0.595213000 -3.629233000

H 0.871987000 0.395934000 -

4.300546000

C 2.541320000 -0.439630000 -3.201826000

H 0.304867000 -1.178448000 -2.294198000

H 2.009132000 1.641680000 -3.489828000

H 2.307509000 -1.458679000 -3.538068000

C -2.079266000 0.916871000 2.530205000

C -2.794403000 -2.544922000 -1.244173000

C 3.924128000 -0.237923000 -2.632872000

H 4.034869000 0.761026000 -2.183648000

H 4.172390000 -0.997541000 -1.873116000

H 4.666960000 -0.340139000 -3.451432000

C -5.345443000 1.851549000 -1.265674000

C 3.074366000 3.866342000 0.474278000

C 1.889946000 4.512550000 -0.294041000

H 2.233839000 5.411114000 -0.841297000

H 1.467816000 3.799395000 -1.019097000

H 1.090424000 4.821936000 0.403889000

C 4.196033000 3.489682000 -0.530977000

H 4.532919000 4.387724000 -1.083605000

H 5.070395000 3.065593000 -0.003535000

H 3.828438000 2.748057000 -1.257063000

C 3.647064000 4.932259000 1.437654000

H 2.891121000 5.289292000 2.161022000

H 4.519418000 4.555563000 2.002834000

H 3.983943000 5.806270000 0.851069000

C -0.994988000 1.787069000 -1.967824000

C -1.447520000 1.579336000 -3.220594000

H -0.270411000 2.574681000 -1.738938000

H -1.384759000 1.214425000 -1.119628000

H -1.093578000 2.176235000 -4.070636000

H -2.182055000 0.796037000 -3.439554000

H -4.475426000 -0.594216000 -2.116660000

H -3.885400000 2.274611000 1.040587000

C -3.976468000 -3.500911000 -

0.952363000

C -2.525440000 -2.465365000 -2.763307000

H -1.892608000 -2.979083000 -0.775330000

H -2.278688000 -3.469229000 -3.156518000

H -3.409715000 -2.105908000 -3.321832000

H -1.677721000 -1.791114000 -2.979841000

H -3.788169000 -4.497890000 -1.392509000

H -4.132007000 -3.630435000 0.133172000

H -4.916108000 -3.109849000 -1.384828000

C -4.788296000 3.259835000 -1.572957000

H -5.624836000 1.383771000 -2.231144000

C -6.625560000 1.938565000 -0.401489000

H -7.392332000 2.559849000 -0.900282000

H -7.056590000 0.937909000 -0.219975000

H -6.408915000 2.395216000 0.581812000

H -5.536215000 3.861080000 -2.122156000

H -4.541812000 3.805040000 -0.643182000

H -3.869118000 3.199707000 -2.181194000

C -1.467256000 2.333265000 2.456419000

C -3.111584000 0.825477000 3.679561000

H -1.254158000 0.221330000 2.767016000

H -0.964836000 2.576800000 3.409545000

H -0.713226000 2.402641000 1.654058000

H -2.236799000 3.106828000 2.277288000

H -2.648961000 1.119525000 4.639839000

H -3.970389000 1.497831000 3.496890000

H -3.504701000 -0.200988000 3.789563000

[5]TS-III$_{Decoor}$

Zero-point correction= 0.835235 (Hartree/Particle)

Thermal correction to Energy= 0.886175

Thermal correction to Enthalpy= 0.887119

Thermal correction to Gibbs Free Energy= 0.751421

Sum of electronic and zero-point Energies= -2218.619089

Sum of electronic and thermal Energies=

-2218.567205
Sum of electronic and thermal Enthalpies= -2218.652566

Sum of electronic and thermal Free Energies= -2218.702903
E solvent= -2217.951482

C	-2.190666	2.390648	-0.204801
C	-3.475212	2.372858	0.377876
H	-3.749281	1.558517	1.057766
C	-4.397186	3.397850	0.099486
H	-5.391441	3.370272	0.560505
C	-4.048469	4.450366	-0.760212
H	-4.768370	5.247911	-0.976495
C	-2.769875	4.475040	-1.346096
H	-2.489224	5.293000	-2.019926
C	-1.849976	3.451814	-1.075016
C	0.273314	1.950008	1.190482
C	-0.292294	2.951647	2.019955
H	-1.353338	3.197452	1.913817
C	0.462498	3.652711	2.969727
H	-0.018390	4.416852	3.590758
C	1.824499	3.361994	3.113938
H	2.435821	3.887876	3.856129
C	2.403459	2.386700	2.292169
H	3.468043	2.151680	2.399640
C	1.667162	1.674251	1.312772
C	2.477793	0.672610	0.526569
C	3.357045	1.093748	-0.516049
C	4.233935	0.139316	-1.078762
C	4.294041	-1.195970	-0.650775
C	3.421241	-1.583279	0.380010
C	2.526832	-0.679722	0.983120
C	-1.766279	-0.188026	1.182489
C	-2.112877	0.027042	2.533273
H	-1.841705	0.968604	3.026192
C	-2.784396	-0.974526	3.235223
H	-3.058148	-0.833435	4.286454
C	-3.101134	-2.182700	2.575414
H	-3.628741	-2.950319	3.150453
C	-2.765138	-2.452487	1.240283
C	-2.048970	-1.424474	0.508590
Ni	-0.643451	-0.232238	-1.649769
O	-1.654433	-1.621050	-0.740583
P	-0.952861	1.036571	0.099827
H	-0.859155	3.474438	-1.544308
C	0.469949	-1.856790	-2.524365
H	0.720743	-2.429068	-1.626226
C	1.146615	-0.689665	-2.830975
H	0.026535	0.986810	-2.176675
H	-0.203974	-2.341095	-3.237637
H	1.979178	-0.343945	-2.211560
C	1.693071	-1.156944	2.178418
C	3.491771	2.516319	-1.098550
C	5.285714	-2.175439	-1.277138
C	-3.116582	-3.805731	0.578120
C	-1.817564	-4.541191	0.150257

H	-2.068903	-5.494342	-0.353650
H	-1.232246	-3.918775	-0.544572
H	-1.190622	-4.775768	1.029855
C	-4.003431	-3.566476	-0.673205
H	-4.247559	-4.530918	-1.158533
H	-4.953576	-3.074274	-0.394560
H	-3.474746	-2.928979	-1.398775
C	-3.893568	-4.742558	1.532974
H	-3.302367	-5.008534	2.428665
H	-4.849055	-4.297243	1.866725
H	-4.132258	-5.682736	1.003073
C	-2.003564	-0.235986	-3.763768
C	-2.853080	0.526318	-3.026889
H	-1.183391	0.210382	-4.335080
H	-2.193658	-1.306092	-3.911892
H	-2.670046	1.606925	-2.955581
C	-4.104513	0.013283	-2.369373
H	4.903299	0.462813	-1.888426
H	3.447432	-2.617013	0.744473
H	1.056950	-0.230485	-3.822306
H	-4.119586	0.246164	-1.289866
H	-4.993970	0.503209	-2.812861
H	-4.208617	-1.077098	-2.489313
H	3.957826	2.360944	-2.092407
C	2.185633	3.292208	-1.364883
C	4.491789	3.386772	-0.295975
H	2.406731	4.173896	-1.995348
H	1.451141	2.663435	-1.900179
H	1.716629	3.656270	-0.436607
H	4.713986	4.317562	-0.850848
H	4.078424	3.673611	0.686152
H	5.444149	2.852738	-0.128418
C	1.041074	-2.536496	1.947223
H	0.871883	-0.436628	2.336377
C	2.553282	-1.170652	3.465763
H	2.960434	-0.169247	3.691950
H	1.946922	-1.496920	4.330789
H	3.405066	-1.868464	3.362395
H	0.324832	-2.752581	2.759562
H	0.485289	-2.560872	0.994974
H	1.788137	-3.352499	1.936481
C	4.582056	-3.412775	-1.879202
H	5.791847	-1.641165	-2.106400
C	6.374538	-2.597463	-0.262646
H	7.117650	-3.261742	-0.741391
H	6.907418	-1.718825	0.142044
H	5.932811	-3.145632	0.589841
H	5.315547	-4.071366	-2.379740
H	4.078532	-4.009103	-1.096321
H	3.818898	-3.118597	-2.621088

7 Supplementary References

(1) Göttker-Schnetmann, I.; Mecking, S. A Practical Synthesis of [(tmeda)Ni(CH_3)$_2$], Isotopically Labeled [(tmeda)Ni($^{13}CH_3$)$_2$], and Neutral Chelated-Nickel Methyl Complexes. *Organometallics* **2020**, *39*, 3433-3440.

(2) Jang, K. P.; Hutson, G. E.; Johnston, R. C.; McCusker, E. O.; Cheong, P. H. Y.; Scheidt, K. A. Asymmetric Homoenolate Additions to Acyl Phosphonates through Rational Design of a Tailored N-Heterocyclic Carbene Catalyst. *J. Am. Chem. Soc.* **2014**, *136*, 76-79.

(3) Roberti, M.; Pizzirani, D.; Recanatini, M.; Simoni, D.; Grimaudo, S.; Di Cristina, A.; Abbadessa, V.; Gebbia, N.; Tolomeo, M. Identification of a terphenyl derivative that blocks the cell cycle in the G0-G1 phase and induces differentiation in leukemia cells. *Journal of medicinal chemistry* **2006**, *49*, 3012-3018.

(4) Becht, J.-M.; Ngouela, S.; Wagner, A.; Mioskowski, C. A straightforward anionic coupling for the synthesis of ortho-bromobiaryls. *Tetrahedron* **2004**, *60*, 6853-6857.

(5) Hicks, J. D.; Hyde, A. M.; Cuezva, A. M.; Buchwald, S. L. Pd-Catalyzed N-Arylation of Secondary Acyclic Amides: Catalyst Development, Scope, and Computational Study. *J. Am. Chem. Soc.* **2009**, *131*, 16720-16734.

(6) Rezai, N.; Meybodi, F. A.; Salehi, P. Protection of Alcohols and Phenols with Dihydropyran and Detetrahydropyranylation by $ZrCl_4$. *Synth. Commun.* **2000**, *30*, 1799 - 1805.

(7) He, L.-P.; Liu, J.-Y.; Pan, L.; Li, Y.-S. Ethylene polymerization of the new titanium complexes bearing a phosphine oxide-bridged bisphenolato ligand. *J. Polym. Sci. A: Polym. Chem.* **2008**, *46*, 7062-7073.

(8) X-RED version 1.31 (2005) Stoe Data Reduction Program.

(9) Dolomanov, O. V.; Bourhis, L. J.; Gildea, R. J.; Howard, J. A. K.; Puschmann, H. OLEX2: a complete structure solution, refinement and analysis program. *J. Appl. Crystallogr.* **2009**, *42*, 339-341.

(10) Sheldrick, G. SHELXT - Integrated space-group and crystal-structure determination. *Acta Crystallogr. A* **2015**, *71*, 3-8.

(11) Bourhis, L. J.; Dolomanov, O. V.; Gildea, R. J.; Howard, J. A.; Puschmann, H. The anatomy of a comprehensive constrained, restrained refinement program for the modern computing environment - Olex2 dissected. *Acta. Crystallogr. A* **2015**, *71*, 59-75.

(12) Spek, A. Single-crystal structure validation with the program PLATON. *J. Appl. Crystallogr.* **2003**, *36*, 7-13.

(13) Ragone, F.; Poater, A.; Cavallo, L. Flexibility of N-Heterocyclic Carbene Ligands in Ruthenium Complexes Relevant to Olefin Metathesis and Their Impact in the First Coordination

Sphere of the Metal. *J. Am. Chem. Soc.* **2010**, *132*, 4249-4258.

(14) Falivene, L.; Credendino, R.; Poater, A.; Petta, A.; Serra, L.; Oliva, R.; Scarano, V.; Cavallo, L. SambVca 2. A Web Tool for Analyzing Catalytic Pockets with Topographic Steric Maps. *Organometallics* **2016**, *35*, 2286-2293.

(15) Falivene, L.; Cao, Z.; Petta, A.; Serra, L.; Poater, A.; Oliva, R.; Scarano, V.; Cavallo, L. Towards the online computer-aided design of catalytic pockets. *Nat. Chem.* **2019**, *11*, 872-879.

(16) Zhang, Y.; Mu, H.; Pan, L.; Wang, X.; Li, Y. Robust Bulky [P,O] Neutral Nickel Catalysts for Copolymerization of Ethylene with Polar Vinyl Monomers. *ACS Cat.* **2018**, *8*, 5963-5976.

(17) Berkefeld, A.; Möller, H. M.; Mecking, S. Unusual Reactivity of N,N,N′,N′-Tetramethylethylenediamine-Coordinated Neutral Nickel(II) Polymerization Catalysts. *Organometallics* **2009**, *28*, 4048-4055.

(18) Xin, B. S.; Sato, N.; Tanna, A.; Oishi, Y.; Konishi, Y.; Shimizu, F. Nickel Catalyzed Copolymerization of Ethylene and Alkyl Acrylates. *J. Am. Chem. Soc.* **2017**, *139*, 3611-3614.

(19) Perdew, J. P. Density-functional approximation for the correlation energy of the inhomogeneous electron gas. *Phys. Rev. B* **1986**, *33*, 8822-8824.

(20) Perdew, J. P. Erratum: Density-functional approximation for the correlation energy of the inhomogeneous electron gas. *Phys. Rev. B* **1986**, *34*, 7406-7406.

(21) Becke, A. D. Density-functional exchange-energy approximation with correct asymptotic behavior. *Phys. Rev. A* **1988**, *38*, 3098-3100.

(22) Frisch, M. J.; Trucks, G. W.; Schlegel, H. B.; Scuseria, G. E.; Robb, M. A.; Cheeseman, J. R.; Scalmani, G.; Barone, V.; Mennucci, B.; Petersson, G. A.; Nakatsuji, H.; Caricato, M.; Li, X.; Hratchian, H. P.; Izmaylov, A. F.; Bloino, J.; Zheng, G.;. Sonnenberg, J. L; Hada, M.; Ehara, M.; Toyota, K.; Fukuda, R.; Hasegawa, J.; Ishida, M.; Nakajima, T.; Honda, Y.; Kitao, O.; Nakai, H.; Vreven, T.; Montgomery, J. A.; Peralta, J. E.; Ogliaro, F.; Bearpark, M.; Heyd, J. J.; Brothers, E.; Kudin, K. N.; Staroverov, V. N.; Kobayashi, R.; Normand, J.; Raghavachari, K.; Rendell, A.; Burant, J. C.; Iyengar, S. S.; Tomasi, J.; Cossi, M;. Rega, N.; Millam, J. M.; Klene, M.; Knox, J. E.; Cross, J. B.; Bakken, V.; Adamo, C.; Jaramillo, J.; Gomperts, R.; Stratmann, R. E.; Yazyev, O.; Austin, A. J.; Cammi, R.; Pomelli, C.; Ochterski, J. W. R.; Martin, L.; Morokuma, K.; Zakrzewski, V. G.; Voth, G. A.; Salvador, P.; Dannenberg, J. J.; Dapprich, S.; Daniels, A. D.; Farkas, Ö.; S163 Foresman, J. B.; Ortiz, J. V.; Cioslowski, J.; Fox D. J. Gaussian 09 Revision A.1, Gaussian, Inc., Wallingford, CT, 2009.

(23) Weigend, F.; Ahlrichs, R. Balanced basis sets of split valence, triple zeta valence and quadruple zeta valence quality for H to Rn: Design and assessment of accuracy. *Phys. Chem. Chem. Phys* **2005**, *7*, 3297-3305.

(24) Häussermann, U.; Dolg, M.; Stoll, H.; Preuss, H.; Schwerdtfeger, P.; Pitzer, R. M. Accuracy

of energy-adjusted quasirelativistic ab initio pseudopotentials. *Mol. Phys.* **1993**, *78*, 1211-1224.

(25) Küchle, W.; Dolg, M.; Stoll, H.; Preuss, H. Energy-adjusted pseudopotentials for the actinides. Parameter sets and test calculations for thorium and thorium monoxide. *J. Chem. Phys.* **1994**, *100*, 7535-7542.

(26) Leininger, T.; Nicklass, A.; Stoll, H.; Dolg, M.; Schwerdtfeger, P. The accuracy of the pseudopotential approximation. II. A comparison of various core sizes for indium pseudopotentials in calculations for spectroscopic constants of InH, InF, and InCl. *J. Chem. Phys.* **1996**, *105*, 1052-1059.

(27) Tomasi, J.; Persico, M. Molecular Interactions in Solution: An Overview of Methods Based on Continuous Distributions of the Solvent. *Chem. Rev.* **1994**, *94*, 2027-2094.

(28) Barone, V.; Cossi, M. Quantum Calculation of Molecular Energies and Energy Gradients in Solution by a Conductor Solvent Model. *J. Phys. Chem. A* **1998**, *102*, 1995-2001.

(29) Adamo, C.; Barone, V. Toward reliable density functional methods without adjustable parameters: The PBE0 model. *J. Chem. Phys.* **1999**, *110*, 6158-6170.

(30) Grimme, S.; Antony, J.; Ehrlich, S.; Krieg, H. A consistent and accurate ab initio parametrization of density functional dispersion correction (DFT-D) for the 94 elements H-Pu. *J. Chem. Phys.* **2010**, *132*, 154104.

(31) Perdew, J. P.; Wang, Y. Accurate and simple analytic representation of the electron-gas correlation energy. *Phys. Rev. B* **1992**, *45*, 13244-13249.

(32) Marenich, A. V.; Cramer, C. J.; Truhlar, D. G. Universal solvation model based on solute electron density and on a continuum model of the solvent defined by the bulk dielectric constant and atomic surface tensions. *J. Phys. Chem. B* **2009**, *113*, 6378-6396.

(33) Contreras-García, J; Johnson, E.R.; Keinan, S.; Chaudret, R.; Piquemal, J-P.; Beratan, D. N.; Yang, W. *J. Am. Chem. Soc.* **2010**, *132*, 18, 6498–6506.

(34) Johnson, E.R.; Keinan, S.; Mori-Sanchez, P.; Contreras-García, J; Cohen, A.J.; Yang, W. *J. Chem. Theory Comput*. **2011**, *7*, 3, 625–632
